# The Society for Immunotherapy of Cancer consensus statement on immunotherapy for the treatment of hematologic malignancies: multiple myeloma, lymphoma, and acute leukemia

**DOI:** 10.1186/s40425-016-0188-z

**Published:** 2016-12-20

**Authors:** Michael Boyiadzis, Michael R. Bishop, Rafat Abonour, Kenneth C. Anderson, Stephen M. Ansell, David Avigan, Lisa Barbarotta, Austin John Barrett, Koen Van Besien, P. Leif Bergsagel, Ivan Borrello, Joshua Brody, Jill Brufsky, Mitchell Cairo, Ajai Chari, Adam Cohen, Jorge Cortes, Stephen J. Forman, Jonathan W. Friedberg, Ephraim J. Fuchs, Steven D. Gore, Sundar Jagannath, Brad S. Kahl, Justin Kline, James N. Kochenderfer, Larry W. Kwak, Ronald Levy, Marcos de Lima, Mark R. Litzow, Anuj Mahindra, Jeffrey Miller, Nikhil C. Munshi, Robert Z. Orlowski, John M. Pagel, David L. Porter, Stephen J. Russell, Karl Schwartz, Margaret A. Shipp, David Siegel, Richard M. Stone, Martin S. Tallman, John M. Timmerman, Frits Van Rhee, Edmund K. Waller, Ann Welsh, Michael Werner, Peter H. Wiernik, Madhav V. Dhodapkar

**Affiliations:** 1 Division of Hematology-Oncology, Department of Medicine, University of Pittsburgh School of Medicine, University of Pittsburgh Cancer Institute, University of Pittsburgh Medical Center, 5150 Centre Avenue, Suite 564, Pittsburg, PA 15232 USA; 2 Hematopoietic Cellular Therapy Program, University of Chicago, 5841 S. Maryland Avenue, Chicago, IL 60637 USA; 3 Indiana University School of Medicine, 980 W. Walnut St., Walther Hall-R3, C400, Indianapolis, IN 46202 USA; 4 Dana-Farber Cancer Institute, 450 Brookline Avenue, Boston, MA 02215 USA; 5 Mayo Clinic, 200 First Street SW, Rochester, MN 55905 USA; 6 Beth Israel Deaconess Medical Center, 330 Brookline Avenue, Boston, MA 02215 USA; 7 Smilow Cancer Hospital at Yale New Haven, 35 Park Street, New Haven, CT 06519 USA; 8 National Institutes of Health, Building 10-CRC Room 3-5330, Bethesda, MD 20814 USA; 9 Weill Cornell Medical College, 407 E 71st St, New York, NY 10065 USA; 10 Mayo Clinic, 13400 E. Shea Blvd., Scottsdale, AZ 85259 USA; 11 Johns Hopkins School of Medicine, 1650 Orleans St, Baltimore, MD 21231 USA; 12 Icahn School of Medicine at Mount Sinai, One Gustave L Levy Place, New York, NY 10029 USA; 13 University of Pittsburgh Cancer Institute, 5150 Centre Avenue, Pittsburgh, PA 15232 USA; 14 New York Medical College at Maria Fareri Children’s Hospital, 100 Woods Road, Valhalla, New York 10595 USA; 15 Abramson Cancer Center at the University of Pennsylvania, 3400 Civic Center Blvd, Philadelphia, PA 19104 USA; 16 MD Anderson Cancer Center, 1515 Holcombe Blvd, Houston, TX 77030 USA; 17 City of Hope National Medical Center, 1500 East Duarte Road, Duarte, CA 91010 USA; 18 Wilmot Cancer Institute, University of Rochester, 601 Elmwood Avenue, Box 704, Rochester, NY 14642 USA; 19 Johns Hopkins University School of Medicine, 401 N. Broadway, Baltimore, MD 21231 USA; 20 Yale Cancer Center, 333 Cedar Street, New Haven, CT 06511 USA; 21 Washington University School of Medicine, 660 S. Euclid Ave., St. Louis, MO 63110 USA; 22 The University of Chicago, 5841 S. Maryland Ave, Chicago, IL 60637 USA; 23 National Institutes of Health, National Cancer Institute, 8500 Roseweood Drive, Bethesda, MD 20814 USA; 24 City of Hope National Medical Center, 1500 E. Duarte Road, Beckman Bldg., Room 4117, Duarte, CA 91010 USA; 25 Division of Medical Oncology, Stanford University School of Medicine, 269 Campus Drive, Stanford, CA 94305 USA; 26 Department of Medicine-Hematology and Oncology, Case Western Reserve University, 11100 Euclid Ave., Cleveland, OH 44106 USA; 27 Department of Hematology, Mayo Clinic Cancer Center, 200 First Street SW, Rochester, MN 55905 USA; 28 Helen Diller Family Comprehensive Cancer Center, University of California San Francisco, Box 0324, San Francisco, CA 94143 USA; 29 Division of Hematology/Oncology, University of Minnesota, 420 Delaware St SE, Minneapolis, MN 55455 USA; 30 Dana-Farber Cancer Institute, 450 Brookline Avenue, Dana B106, Boston, MA 02215 USA; 31 The University of Texas MD Anderson Cancer Center, 1515 Holcombe Blvd., Unit 429, Houston, TX 77030 USA; 32 Swedish Cancer Institute, 1221 Madison Street, Suite 1020, Seattle, WA 98104 USA; 33 University of Pennsylvania, 3400 Civic Center Blvd, PCAM 12 South Pavilion, Philadelphia, PA 19104 USA; 34 Patients Against Lymphoma, 3774 Buckwampum Road, Riegelsville, PA 18077 USA; 35 Dana-Farber Cancer Institute, 450 Brookline Ave, Mayer 513, Boston, MA 02215 USA; 36 Hackensack University Medical Center, 92 2nd St., Hackensack, NJ 07601 USA; 37 Memorial Sloan Kettering Cancer Center, 1275 York Avenue, New York, NY 10065 USA; 38 University of California, Los Angeles, 10833 LeConte Ave., Los Angeles, CA 90095 USA; 39 University of Arkansas for Medical Sciences, Myeloma Institute, 4301 W Markham #816, Little Rock, AR 72205 USA; 40 Winship Cancer Institute, Emory University, 1365B Clifton Road NE, Atlanta, GA 30322 USA; 41 University of Pittsburgh Medical Center, 200 Lothrop St., Pittsburgh, PA 15213 USA; 42 Patient Advocate, 33 East Bellevue Place, Chicago, IL 60611 USA; 43 Cancer Research Foundation of New York, 43 Longview Lane, Chappaqua, NY 10514 USA; 44 Department of Hematology & Immunobiology, Yale University, 333 Cedar Street, Box 208021, New Haven, CT 06510 USA

**Keywords:** Cancer immunotherapy, Hematologic malignancies, Acute leukemia, Lymphoma, Multiple myeloma, Immunotherapy

## Abstract

Increasing knowledge concerning the biology of hematologic malignancies as well as the role of the immune system in the control of these diseases has led to the development and approval of immunotherapies that are resulting in impressive clinical responses. Therefore, the Society for Immunotherapy of Cancer (SITC) convened a hematologic malignancy Cancer Immunotherapy Guidelines panel consisting of physicians, nurses, patient advocates, and patients to develop consensus recommendations for the clinical application of immunotherapy for patients with multiple myeloma, lymphoma, and acute leukemia. These recommendations were developed following the previously established process based on the Institute of Medicine’s clinical practice guidelines. In doing so, a systematic literature search was performed for high-impact studies from 2004 to 2014 and was supplemented with further literature as identified by the panel. The consensus panel met in December of 2014 with the goal to generate consensus recommendations for the clinical use of immunotherapy in patients with hematologic malignancies. During this meeting, consensus panel voting along with discussion were used to rate and review the strength of the supporting evidence from the literature search. These consensus recommendations focus on issues related to patient selection, toxicity management, clinical endpoints, and the sequencing or combination of therapies. Overall, immunotherapy is rapidly emerging as an effective therapeutic strategy for the management of hematologic malignances. Evidence-based consensus recommendations for its clinical application are provided and will be updated as the field evolves.

**Electronic supplementary material:**

The online version of this article (doi:10.1186/s40425-016-0188-z) contains supplementary material, which is available to authorized users.

## Introduction

The incidence of hematologic malignancies has steadily increased over the past 30 years. Over this period of time, there have been significant advancements in the understanding of the biology of these diseases, including the important role that the immune system plays in their development, maintenance, and eradication. As a result of these discoveries, there has been concurrent advancement in immunotherapies specifically developed for the treatment of hematologic malignancies. Probably the most remarkable example of the success of immunotherapy for hematologic malignancies is the anti-CD20 monoclonal antibody rituximab, which has been incorporated into almost all aspects in the treatment of B cell malignancies.

An understanding of the basic mechanisms of the immune system as it relates to hematologic malignancies has been increasing rapidly. This understanding has accelerated the translation of this research and has led to the development of several novel immunotherapeutic approaches. A major recent example is research related to tumor immune evasion mechanisms. The programmed cell death-1 (PD-1) pathway has emerged as a highly relevant immune checkpoint pathway in a number of hematologic malignancies, particularly Hodgkin’s lymphoma [1]. This work has led to the development of several antibodies that disrupt the interactions between negative regulatory receptors on tumor-specific T cells and their ligands on tumor cells or antigen-presenting cells.

**Fig. 1:**
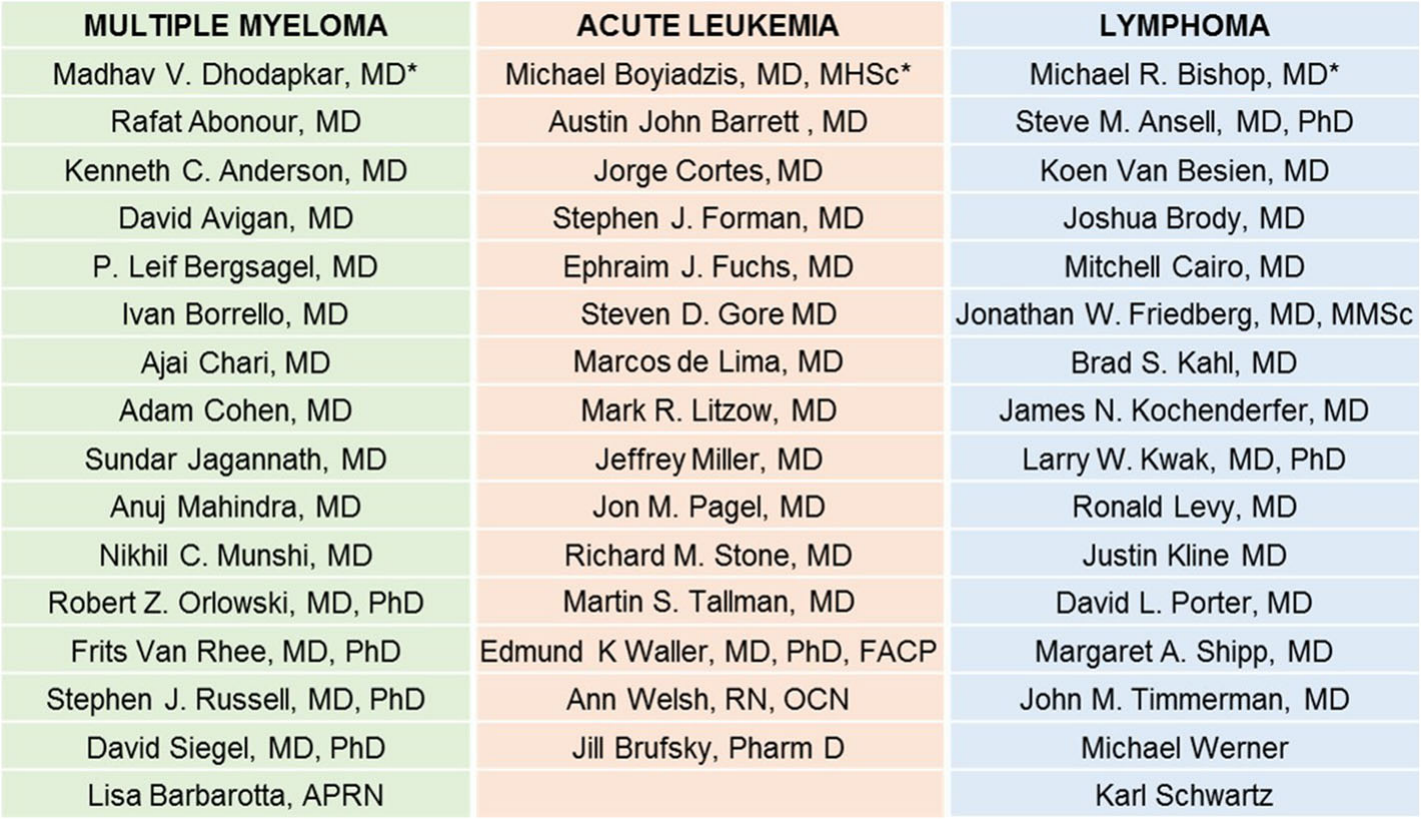
Table of the Cancer Immunotherapy Guidelines for Hematologic Malignancy participants. Asterisks (*) indicate panel chair and steering committee member

In response to the growing number of immunotherapeutic agents that have been approved and are in final stages of clinical investigation in the treatment of hematologic malignances, SITC formed a hematologic malignancy Cancer Immunotherapy Guidelines panel to provide guidance to practicing clinicians caring for patients with multiple myeloma, lymphoma, and acute leukemia. SITC is a nonprofit professional organization dedicated to the basic understanding and clinical applications of cancer immunotherapy. The panel consisted of experts in hematologic malignancies, including physicians, nurses, patient advocates, and patients (Additional file 1). This panel met to consider issues related to patient selection, toxicity management, treatment cessation guidelines and current recommendations for treatment sequencing with the goal of preparing a consensus statement on clinical use of immunotherapy for patients with hematologic malignancies. The hematologic malignancy panel was comprised of three separate disease-specific panels focused on multiple myeloma, lymphoma, and acute leukemia (Fig. 1). The consensus panels were charged to provide evidence-based guidelines and recommendations with a major emphasis on US Food and Drug Administration (FDA)-approved agents. While the members of the panel agreed that allogeneic hematopoietic stem cell transplantation (HSCT) is an important and effective therapeutic option in the management of hematologic malignancies, it was not included in the current consensus statement at the recommendation of the Steering Committee. Although the major emphasis of this report is to provide summaries and recommendations relative to approved agents, the panel felt it was also important to addresses biological principles and treatment that would be relevant to clinical oncologists in regard to the future of immunotherapy research for hematologic malignancies.

## Methods

### Consensus statement development

This consensus statement was developed using the standards delineated by the SITC consensus statement on tumor immunotherapy for the treatment of cutaneous melanoma as described previously [2]. These standards were originally developed based on the Institute of Medicine’s Standards for Developing Trustworthy Clinical Practice Guidelines, and include key components such as establishing a transparent process for guideline development and funding, managing and reporting conflicts of interest, including a multidisciplinary and balanced panel, establishing an evidence-based foundation and rating system for the strength of the evidence, reporting the results through a publicly available website and publication, and having a plan to update the recommendations [2, 3].

In December 2014, SITC convened a hematologic malignancy Cancer Immunotherapy Guidelines panel charged with developing clinical practice guidelines for the use of immunotherapy in multiple myeloma, lymphoma, and acute leukemia. To do so, these Steering Committee-led panels considered patient selection, toxicity management, assessment of response, and sequencing as well as the combination of therapies for immunotherapies in current clinical practice. Due to differences in the regulation and availability of immunotherapy agents world-wide, the consensus panel focused on drugs currently approved by the US FDA. These consensus guidelines are not intended to be a substitute for the professional judgment of treating physicians. The full consensus recommendations as well as any future updates can be found on the SITC website [4].

### Consensus panel and conflicts of interest

Potential consensus panel members including physicians, nurses, patient advocates, and patients were solicited from SITC members and non-members. Panel members were screened using the SITC conflicts of interest disclosure form. This form requires the disclosure of any financial as well as non-financial conflicts of interests that may have direct implications resulting from the publication of this statement. In addition, no commercial funding was used to support the consensus panel meeting, literature review, or preparation of this manuscript.

The hematologic malignancy panel, consisting of three separate disease-specific panels for multiple myeloma, lymphoma, and acute leukemia, met in December, 2014, to review and discuss results from a previously distributed questionnaire collecting information on the panel member’s role in patient care, primary clinical focus, experience with FDA-approved agents, and current clinical practices concerning the use of, or recommended use of immunotherapy agents. The final version of this consensus statement was made available to the entire SITC membership for an open comment period. These comments were collected and considered in the final version of this manuscript (Additional file 2).

### Literature review

The MEDLINE database was used to perform a systematic search of scientific literature from 2004 to 2014. The search was limited to “humans” and “clinical trials or controlled clinical trials or randomized controlled clinical trials.” The results from the literature search are listed according to each disease type as follows. These bibliographies were supplemented with additional literature as identified by the panel.

#### Multiple myeloma

The search terms included “myeloma and lenalidomide,” “myeloma and pomalidomide,” “myeloma and thalidomide,” “myeloma and monoclonal antibody,” “myeloma and checkpoint blockade or PD-1 or PD-L1 or B7-H1,” “myeloma and oncolytic virus,” “myeloma and virotherapy,” and “myeloma and dendritic cell vaccine or idiotype vaccine.” After duplicates and irrelevant citations were removed, this search resulted in a 173-item bibliography (Additional file 3: Bibliography I).

#### Lymphoma

The search terms included “lymphoma and rituximab or ofatumumab,” “lymphoma and checkpoint blockade,” “lymphoma and chimeric antigen receptor,” “lymphoma and idiotype vaccine,” “lymphoma and denileukin diftitox,” “lymphoma and interferon alfa-2b,” “mantle cell lymphoma and lenalidomide,” and “mantle cell lymphoma and bortezomib.” After duplicates and irrelevant citations were removed, this search resulted in a 138-item bibliography (Additional file 3: Bibliography II).

#### Acute leukemia

The search terms included “AML and epigenetic therapy,” “AML and hypomethylating agents or 5-azacytidine or decitabine,” “AML and monoclonal antibody,” “ALL and monoclonal antibody or rituximab or blinatumomab,” “AML and checkpoint blockade,” “AML and CAR or CART,” and “ALL and CAR or CART.” After duplicates and irrelevant citations were removed, this search resulted in a 56-item bibliography (Additional file 3: Bibliography III).

The literature was reviewed and graded according to the previously established rating system [2]. In summary, Level A was defined as strong supporting evidence-based data from prospective, randomized clinical trials, and meta-analyses; Level B was defined as moderate supporting data from uncontrolled, prospective clinical trials; and Level C represented weak supporting data from retrospective reviews and case reports.

## Multiple myeloma

Immune-based therapies in multiple myeloma (MM) can be classified as current or emerging therapies, based largely on the level of clinical evidence. The panel therefore first considered the status of current therapies, followed by considerations for the current status and optimal evaluation of emerging therapies.

### Current immunotherapies in myeloma

Two broad categories of current immune/immune-modulating therapies in MM are immune-modulating drugs (IMiDs) and anti-tumor monoclonal antibodies (mAbs). Thalidomide, lenalidomide, and pomalidomide are already FDA-approved for use in MM [5, 6]. While non-immune effects of IMiDs are recognized, the myeloma panel voted to include these agents among the list of immune therapies for these guidelines. Although anti-tumor antibodies were not yet FDA-approved at the time of the panel review, the level of evidence supporting the clinical activity of some agents (anti-CD38 mAb (daratumumab) and anti-SLAMF7 mAb (elotuzumab)) was felt to be high, and therefore, they were included among current immune therapies [7, 8]. Both elotuzumab and daratumumab recently received FDA approval for relapsed myeloma.

### IMiDs: thalidomide, lenalidomide, and pomalidomide

Over the past 15 years, the use of IMiDs together with proteasome inhibitors has transformed the therapeutic landscape and outcome of patients with MM. Lenalidomide plus dexamethasone (Rd) was superior to dexamethasone alone in two phase III trials involving patients with relapsed/refractory MM (RRMM) [9, 10]. Rd was also superior to dexamethasone in the setting of induction therapy [11]. Use of a lower dose of dexamethasone led to an improved safety profile, and accordingly, Rd has been commonly adopted in the US [12]. In a clinical trial involving elderly patients with previously untreated MM, continuous Rd was superior to fixed duration Rd and to melphalan, prednisolone, and thalidomide (MPT) [13].

The Rd regimen has also been combined with several agents, most notably proteasome inhibitors. Data comparing the addition of carfilzomib to Rd (KRd) in RRMM demonstrated improved progression-free survival (PFS) [14]. In a phase III trial, the addition of elotuzumab to Rd led to improved PFS in patients with RRMM [15]. Recently, the addition of ixazomib to the Rd backbone also led to improved PFS in RRMM [16]. It should be noted that these phase III studies were performed in patients with lenalidomide-sensitive disease, though differences in patient populations preclude across study comparisons.

In the front-line setting, results from trials comparing Rd to triplets such as those in combination with bortezomib (VRd), carfilzomib, and elotuzumab are currently awaited. Initial data from SWOG 0777 have demonstrated superiority of VRd over Rd in front-line therapy of myeloma [17]. Data from randomized clinical trials evaluating the timing of stem cell transplantation in the era of novel agents is also awaited. Initial data from a phase III trial demonstrated improvement in PFS in patients receiving early stem cell transplantation [18]. Lenalidomide has also been utilized in the setting of maintenance therapy following autologous HSCT as demonstrated in clinical trials Cancer and Leukemia Group B (CALGB) 100104 and IFM 2005–02 or as continuous therapy for transplant ineligible patients (MM-015) [19–21]. All three trials reported significant differences in PFS, and the CALGB trial reported improved 3 year overall survival (OS).

Pomalidomide plus dexamethasone demonstrated remarkable activity in patients with RRMM refractory to lenalidomide, and was the last immunotherapy agent approved for therapy of MM [22–24]. Two dosing schedules (2 mg daily or 4 mg on 21/28 day schedule) of pomalidomide (in combination with dexamethasone) have been explored with comparable results [25–27]. Pomalidomide is also active in patients with high-risk cytogenetics such as deletion 17 [28].

In recent years, the E3 ubiquitin ligase cereblon has been identified as a key target of IMiDs [29, 30]. Binding of the drug to cereblon leads to degradation of Ikaros family zinc finger proteins IKZF1 and IKZF3, which then leads to inhibition of tumor cell growth and immune activation [31–33]. In preclinical and early clinical studies, immune activation by IMiDs provides the basis for synergy in combination with vaccines, antibodies and checkpoint inhibitors [34–37]. IMiD therapy leads to activation of both T and natural killer (NK) cells in vivo [27, 38, 39]. IMiD-mediated immune activation is rapid and correlates with clinical response to therapy [27].

Myeloma Panel Recommendations:

- The panel recommends the use of combination therapies with lenalidomide in both front-line and relapsed MM setting based on level A evidence. Data directly comparing regimens commonly utilized in front-line setting is awaited and enrollment in well-designed clinical trials is recommended. In a recent Southwestern Oncology Group (SWOG) study, combination therapy with VRd led to improved outcome compared to Rd [17].
- The front-line regimen for transplant-eligible patients (outside of a clinical trial) preferred by the majority (53.3%) of the panel was VRd, followed by Rd (26.7%) and cyclophosphamide, bortezomib, and dexamethasone (CyBorD) (13.3%) based on level B evidence.
- The front-line regimen for transplant-ineligible patients (outside of a clinical trial) preferred by the panel were Rd (46.6%), VRd (40%), followed by CyBorD (6.7%) based on level B evidence.
- Based on the results of SWOG S0777 (not available at the time of the panel review), VRd is now expected to become the preferred front-line regimen for most patients with newly diagnosed MM based on level A evidence. Participation in ongoing clinical trials comparing this regimen with others is strongly encouraged.
- All panelists recommend the use of a proteasome inhibitor-based regimen in patients with t(4:14), del17p, and plasma cell leukemia based on level B evidence.
- The panel recognizes the lack of level A evidence regarding the timing of stem cell transplant in the era of novel agents. While the results of studies addressing these questions are awaited, most panelists (66.7%) favor consideration of early autologous HSCT. The outcome of the French cohort Intergroupe Francophone Du Myeloma trial has been recently presented and demonstrated improved PFS with early transplant. These data were not available at the time of the panel review [18].
- The majority of the panel (80%) recommends the use of maintenance therapy following autologous HSCT based on level A evidence. The preferred duration of maintenance therapy is until progression (50% of panelists) or for 2 years (28.6% of panelists). Patients on lenalidomide maintenance after prior melphalan exposure should also be monitored for secondary malignancies.
- Preclinical and clinical data support the design of clinical studies combining IMiDs with several immune therapies including monoclonal antibodies, vaccines, and immune checkpoint inhibitors based on level B evidence.
- Nearly all of the clinical data with IMiDs is in combination with concurrent steroids, including that in the setting of current combinations with monoclonal antibodies. Although steroids have the potential to dampen immune activation, recent data suggests that IMiDs may be able to activate immunity even in the setting of concurrent steroids [27, 40]. The impact of concurrent steroids on IMiD-based immune therapies was debated, and the panel agreed that minimizing (or eventually eliminating) steroids would be highly desirable. However, there is a lack of consensus and currently no data to support the need to eliminate steroids, particularly in light of their synergistic direct anti-tumor effects.

### Anti-tumor monoclonal antibodies

In recent years, several anti-tumor mAbs have entered clinical testing in MM. Of these, elotuzumab and daratumumab have entered phase III testing. Elotuzumab is a fully humanized mAb against the glycoprotein SLAMF-7 expressed on myeloma and NK cells [41]. In preclinical models, elotuzumab illustrated anti-tumor effects via NK activation and enhanced antibody-dependent cytotoxicity [41]. In a phase II trial, elotuzumab plus Rd (Elo-Rd) achieved a 92% objective response rate (ORR) in patients with RRMM [42]. In a recent phase III trial, Elo-Rd led to an improvement in PFS compared to Rd in patients with RRMM, including those with high-risk features [15]. In this study, median PFS was 19.4 months in the Elo-Rd group vs. 14.9 months in the Rd group alone, with a hazard ration of .70 (95% CI: .57-.85, *P* < .001).

Daratumumab targets CD38 expressed on MM cells as well as hematopoietic progenitor cells, endothelial cells, and activated immune cells [43]. Anti-myeloma effects of daratumumab involve several mechanisms including direct as well as immune-mediated effects [44]. Preliminary studies with daratumumab showed promising single agent activity with 31% objective responses in heavily pretreated RRMM, including those refractory to both proteasome inhibitors and IMiDs [45]. These results were confirmed in a phase I-II study, illustrating a 36% response rate and median PFS of 5.6 months in heavily pretreated RRMM patients who received daratumumab monotherapy (16 mg/kg) [46]. In addition, in a phase II, multicenter trial daratumumab showed a 29.2% response rate and median PFS of 3.7 months in MMRR patients who had received a median of 5 previous lines of therapy [47]. Moreover, the addition of daratumumab to the Rd backbone led to an improved ORR of 75% in RRMM. Daratumumab has also been combined with pomalidomide in therapy of patients with RRMM [48]. Similar results have been observed with another anti-CD38 mAb, SAR650984 (isatuximab) in patients with RRMM.

Two antibody-drug conjugates (ADCs) are in active clinical testing in RRMM. Indatuximab ravtansine (BT062) is comprised of an anti-CD138 mAb conjugated to the maytansinoid DM4 toxin. In a phase II trial, indatuximab ravtansine plus Rd led to a 78% ORR in patients with RRMM. J6MO-mcMMAF (GSK2857916) is an ADC targeting B cell maturation antigen currently in phase I testing in RRMM. In addition, mAbs targeting several other molecules (e.g., CD40, CD56, CD54) are also in preclinical/early clinical testing. mAbs may be of particular interest in populations at higher risk with current therapies, including those with genetic high risk disease and comorbidities such as renal failure.

Myeloma Panel Recommendations:

- mAbs targeting SLAMF-7 (elotuzumab) or CD38 (daratumumab and SAR650984) in combination with Rd or VRd have demonstrated promising clinical activity in RRMM, including those with high-risk disease. Eligible patients with RRMM or NDMM and particularly those with high-risk features should be encouraged to participate in ongoing clinical trials with these agents based on level B evidence. After the panel meeting, on November 16, 2015, daratumumab received approval to treat patients with relapsed MM who have received at least three prior lines of therapy or are refractory to both a proteasome inhibitor and an IMiD. On November 30, 2015, the FDA approved elotuzumab in combination with lenalidomide and dexamethasone for therapy of relapsed MM who have received one to three prior medications.
- IMiDs often show synergy with mAbs likely in part related to their effects on antibody-dependent cell mediated cytotoxicity (ADCC) and are emerging as important agents for combination with mAbs, although proteasome inhibitors are also being combined with monoclonal antibodies.

### Emerging immunotherapies in myeloma

For the evaluation of emerging therapies, the panel considered both early phase clinical as well as key preclinical findings from the literature in its recommendations. It is recognized that this is an area of active ongoing preclinical and clinical investigation with several new approaches showing promise. Therefore, periodic updates to these recommendations are strongly recommended.

### Immune checkpoint blockade

Several studies have shown that PD-L1 is commonly overexpressed by myeloma tumor cells [49]. In preclinical models, targeting PD-L1 led to anti-tumor effects in murine myeloma [50]. Blockade of the PD-L1 axis leads to activation of antigen-specific T and NK cells in culture [36, 51, 52]. Expression of PD-L1 in MM tumor cells is enriched in minimal residual disease and correlates with risk of progression from monoclonal gammopathy of undetermined significance (MGUS) to MM [53, 54]. In phase II clinical studies with the anti-PD-1 antibody nivolumab, stable disease (but no objective regressions) were observed in RRMM patients [55]. The impact of targeting this axis on survival of MM patients is currently unknown. Early data combining anti-PD-1 antibody pembrolizumab with IMiDs (lenalidomide and pomalidomide) have been reported and suggest promising clinical activity. Limited single agent activity with PD-1 blockade in early myeloma studies suggests the need to consider combination with other agents or approaches that stimulate and expand tumor specific lymphocytes [56, 57].

Myeloma Panel Recommendations:

- There was a consensus among the panel for a strong preclinical rationale for consideration of clinical trials of immune-checkpoint blockade in myeloma.
- The panel identified the following top clinical settings for evaluation of immune checkpoint blockade as single agents: high-risk MM, post-autologous HSCT, and minimal residual disease (MRD).
- The panel identified the following top clinical settings for evaluation of immune checkpoint-based combination therapies: relapsed MM, high-risk MM, and post-autologous HSCT.
- The panel identified the following as the top three agents for combination with immune checkpoint blockade in clinical trials: lenalidomide/IMiDs, vaccine, and other immune checkpoint inhibitors. Update added after the panel meeting: initial reports of studies testing combination of IMiDs and immune checkpoint blockade have shown promising clinical activity. Tumor-directed mAbs are also attractive agents for combination with immune checkpoint blockade. Thus, participation in phase II/III trials testing these combinations is strongly encouraged.

### Immune activating antibodies

There are preclinical data to support targeting co-stimulation via activating antibodies in MM. One example is targeting CD137, which leads to antitumor effects in mouse models [58, 59]. Targeting CD137 has also been shown to synergize with anti-tumor antibodies in preclinical models [60–62].

Myeloma Panel Recommendations:

- There is preclinical rationale to consider clinical evaluation of immune activating antibodies in MM.
- The panel identified the following top clinical settings for evaluation of immune activating antibodies as single agents: relapsed MM, MRD, and post-autologous HSCT.
- The panel identified the following top clinical settings for evaluation of immune activating antibody-based combination therapies: high-risk MM, MRD, and post-autologous HSCT.
- The panel identified the following as the top agents for combination with immune-activating antibodies in clinical trials: lenalidomide/IMiDs and vaccines. With the emergence of anti-tumor antibodies, there is interest in combining these with immune activating antibodies as well.

### Vaccines

Vaccines against tumor-specific antigens represent an attractive strategy to boost tumor immunity and may be particularly relevant with the emergence of checkpoint blockade strategies. Most of the early vaccine studies in MM targeted idiotypic determinants on clonal immunoglobulin (Ig) [63–65]. Ongoing vaccine studies are targeting peptides derived from defined antigens, in combination with lenalidomide and with anti-PD-1 [66]. Several vaccine approaches are in early phase testing. The PVX-410 vaccine consists of a cocktail of HLA-A2 derived peptides from X-box binding protein1 (XBP-1), CD138, and SLAM-F7 antigens that can trigger activation of MM-specific T cells and is currently under evaluation in combination with lenalidomide and anti-PD-1 (NCT01718899). One particular approach to boost immunity to multiple tumor-associated antigens involves fusion of tumor cells and dendritic cells (DCs) [67–69]. In a phase II trial, MM-DC vaccination following autologous HSCT led to a 78% very good partial response (VGPR) rate, and a 47% complete response (CR)/near complete response (nCR) rate, with responses improving from PR to CR/nCR after 100 days in 24% of patients [70]. This approach is now being tested in a randomized multicenter clinical trial. DC vaccines targeting innate lymphocytes such as NKT cells in combination with low-dose lenalidomide also led to tumor regression in asymptomatic MM in a small clinical trial [71]. Another approach has been to use an allogeneic myeloma vaccine in combination with a GM-CSF secreting cell line (myeloma GVAX) [72]. When administered in combination with lenalidomide in patients in a near complete remission (with a detectable immunofixation of their monoclonal protein), patients have shown evidence of priming and persistence of a tumor-specific immune response that correlated with an ongoing disease remission [73]. These data have led to a randomized trial comparing lenalidomide maintenance to lenalidomide + GVAX.

Myeloma Panel Recommendations:

- Vaccines represent an attractive strategy to boost tumor-specific immunity, particularly in the setting of early phase or MRD [70, 71, 74].
- The panel identified MRD and high-risk asymptomatic MM as the top clinical settings for clinical evaluation of vaccine strategies.
- Clinical evaluation of vaccines is strongly recommended in combination with approaches that modify the immune suppressive factors in the tumor microenvironment. The panel identified lenalidomide and immune checkpoint blockade as the top strategies for combination with vaccines.

### Adoptive cellular therapies, including chimeric antigen receptor (CAR) T cells

Adoptive transfer of activated tumor-infiltrating T cells led to tumor regression in patients with melanoma. In a similar fashion, marrow infiltrating T cells have been infused following ex vivo activation in MM patients following autologous HSCT. In a recent study with 25 patients treated using this approach, the presence of central memory a CD8+ T cell phenotype at baseline and persistence of myeloma-specific T cells at 1 year post adoptive T cell therapy was predictive of improved outcome [75, 76]. One strategy involved combining vaccination against tumor antigens with adoptive transfer of anti-CD3-stimulated and vaccine-primed T cells following autologous HSCT in patients with RRMM [77–79]. Antigens targeted via this approach included h-TERT and survivin in one study and MAGE in another study [77, 78]. The combination approach led to enhanced reconstitution of cellular and humoral immunity post-ASCT, including tumor-specific T cells.

CAR T cells against CD19 have shown remarkable clinical activity in acute lymphoblastic leukemia (ALL) [80]. CART-19 cells are currently being evaluated in the setting of MM following autologous HSCT, based on the premise that a subset of drug resistant and possibly clonogenic subset of tumor cells express CD19 [81] and have shown early signs of activity [81]. Another antigen being targeted in early phase clinical trials by this approach is B cell maturation antigen [82], and NY-ESO-1 has been targeted with TCR-engineered T-cells [83]. Other approaches testing CAR-modified T or NK cells are targeting diverse antigens such as kappa light chain, NKG2D, CD38 and SLAMF-7. In addition to cell-based therapies, virotherapy approaches such as measles virus have also been evaluated in patients with RRMM and impressive clinical responses have been observed in some patients with this approach [84]. Virus-induced death of tumor cells is thought to activate anti-tumor immunity, which sets the stage for combination approaches [85].

Myeloma Panel Recommendations:

- Adoptive transfer of costimulated/vaccine-primed T cells as well as marrow infiltrating T cells is a promising strategy for immunotherapy of MM.
- Several CAR-modified T/NK cell approaches are also being developed and in preclinical/early phase testing.
- Virotherapy approaches such as measles virus have led to impressive clinical responses in some patients with RRMM.
- The panel identified patients with high-risk MM or RRMM as well as post-autologous HSCT as preferred clinical settings for clinical evaluation of adoptive cellular therapies.
- The panel also identified combination approaches with lenalidomide and immune checkpoint blockade as preferred combination approaches with these strategies.

### Issues related to immunotherapy research in myeloma

Emergence of effective immune therapies in cancer has led to a reassessment of trial designs and endpoints for evaluating clinical efficacy of such therapies, particularly in the setting of some solid tumors. Traditional criteria such as response rates and PFS did not correlate with OS or clinical benefit for some immune therapies in the setting of solid tumors. Novel immune related response criteria have been proposed in the setting of some solid tumors [86].

Prior preclinical studies have shown that tumor-specific T cells are enriched in the bone marrow in preneoplastic gammopathy and even in the setting of clinical MM, T cells from the bone marrow can be activated to kill autologous tumor cells [76, 87, 88]. Antigen-specific T cells have been detected in both blood and bone marrow of myeloma patients [89, 90]. The phenotypic and functional profile of immune cells in the bone marrow differs from that in circulation, such as with accumulation of IL17-producing T cells [91–94]. MM patients may have significant immune paresis in terms of both humoral and cellular immunity, which may also be impacted by prior therapies [95]. Detection of MRD is emerging as an important parameter and further research is needed to fully integrate MRD testing in the management of myeloma.

Myeloma Panel Recommendations:

- The panel strongly recommends incorporation of detailed immune monitoring in ongoing clinical trials of immune therapies including IMiDs, mAbs and other emerging immune therapies based on level A evidence.
- The panel recommends that immune monitoring should include serial analysis of the bone marrow microenvironment in all studies, as this may differ from the findings in circulating immune cells based on level A evidence.
- Immune monitoring should include both phenotypic and also functional studies including analyses of antigen-specific T cell responses. Guidelines for optimal monitoring of tissue-based immune responses, including those in the bone marrow are currently under development through SITC. Collection, initial processing, transport and storage of tissue aspirates or biopsies may have an impact on results of immune monitoring approaches, and these details should be included in the clinical protocols as well as publication of results.
- Timing of immune monitoring may depend on the nature of the specific therapy. For example, mid-cycle measurements may be needed to fully evaluate the effect of IMiDs [71].
- The nature of preexisting immune paresis may impact the response to immune therapies and should be considered in trial design [95].
- The panel concluded that there are insufficient data to evaluate whether current criteria for clinical response/progression are inadequate for the evaluation of response to immune therapies and whether immune related response criteria as in the setting of solid tumors will be useful in MM. Nonetheless, repeat tumor biopsies should be strongly considered to confirm disease progression and avoid the potential caveat of pseudoprogression due to a transient increase in M protein or the possibility that progression by imaging may reflect immune infiltration as opposed to true progression.
- The panel concluded that there were insufficient data at present to recommend a change in preferred endpoints for MM clinical trials in immunotherapy. However, the panel did note that PFS has not been a consistent or reliable predictor of eventual improvement in OS following immune therapies in solid tumors. It is possible that PFS at a defined time-point (e.g., 2 or 3 years) may be a better correlate of clinical benefit with immune therapies, but this has not been validated.

## Lymphoma

The overall goal of the lymphoma consensus panel was to provide guidance on the use of immunotherapeutics to practicing physicians caring for patients with lymphoma. The specific goal was to provide evidence-based guidelines and recommendations with a major emphasis on FDA-approved agents. In addition, the panel was charged to provide consensus opinions relative to: 1) defining optimal selection of lymphoma patients for immunotherapy; 2) improving management of immunotherapy side effects; 3) how best to monitor responses to immunotherapy; and 4) developing a rationale for sequencing (or combining) immunotherapy with other agents for patients with high-risk and advanced disease.

### Definition of an immunotherapeutic agent

For the purpose of their review, the panel initially addressed how to define whether an agent or therapy was a form of immunotherapy. In the broad sense, several therapeutic agents may have effects upon the immune system, but it may not be their major mechanism of action in the treatment of lymphoma. It was the consensus opinion that the major mechanism of action of a lymphoma immunotherapeutic agent was augmenting anti-tumor responses of immune cells. For example, if an agent directly inhibits tumor escape mechanisms, it would be classified as immunotherapy. In contrast, agents that target a tumor cell directly and mediate cell death mostly through non-immunological pathways (e.g., targeted agents to B cell receptor) were not considered immunotherapeutics. Based on this definition, the list of FDA-approved agents which the panel did not consider as a “true” form of immunotherapy for lymphoma included bortezomib, denileukin diftitox, brentuximab vedotin, temsirolimus and the radio-immunoconjugates Y-90 ibritumomab tiuxetan as well as tositumomab and iodine I-131 tositumomab.

It was thoroughly recognized by the lymphoma panel that allogeneic HSCT is an important and efficacious form of immunotherapy in the treatment of lymphoma [96]. However, it was the recommendation of the steering committee overseeing the hematologic malignancies panels to not include this topic in the first set of guidelines. It is the intent to review in a future update how to incorporate new immunotherapies into both allogeneic and autologous HSCT and how these agents may challenge standard uses of allogeneic transplant.

## Current immunotherapies in lymphoma

### Monoclonal antibodies

#### Rituximab

Rituximab is a chimeric anti-CD20 mAb and is the most commonly used and most clearly defined immunotherapy in lymphoma. Rituximab is FDA-approved for the treatment of non-Hodgkin lymphoma (NHL) and chronic lymphocytic leukemia (CLL). Specifically, rituximab is indicated for the treatment of NHL patients with: 1) relapsed or refractory, low-grade or follicular, CD20-positive, B cell NHL as a single agent; 2) previously untreated follicular, CD20-positive, B cell NHL in combination with cyclophosphamide, vincristine, and prednisone (CVP) chemotherapy; 3) non-progressing (including stable disease), low-grade, CD20-positive, B cell NHL, as a single agent, after first-line CVP chemotherapy; and 4) previously untreated diffuse large B cell, CD20-positive NHL in combination with cyclophosphamide, adriamycin, vincristine, prednisone (CHOP) or other anthracycline-based chemotherapy regimens. Rituximab is also indicated, in combination with fludarabine and cyclophosphamide, for the treatment of patients with previously untreated and previously treated CD20-positive CLL. Although it is well recognized that rituximab may have several mechanisms of action, the primary effect is on normal anti-tumor immune response [97]. It has been demonstrated that the Fab domain of rituximab binds to the CD20 antigen on lymphocytes, and the Fc domain recruits immune effector functions to mediate B cell lysis. Mechanisms of action include direct anti-proliferative effects, complement-dependent cytotoxicity (CDC), and ADCC, with the latter believed to be dominant in vivo [98].

Lymphoma Panel Recommendations:

- Rituximab is FDA-approved as maintenance therapy for previously untreated follicular, CD20-positive B cell NHL and in non-progressing, low-grade, CD20-positive, B cell NHL after first-line CVP chemotherapy. However, the clinical benefit of maintenance rituximab in these two clinical settings remains controversial, based on endpoints that fail to clearly demonstrate a survival benefit. It was the consensus opinion based on level B evidence that maintenance rituximab is not recommended in low burden (as generally defined Groupe D’Etude de Lymphomes Folliculaires), low-grade NHL, and patients should be carefully counseled relative to clinical benefits based on specific endpoints [99, 100].
- Maintenance rituximab is not recommended in diffuse large B cell lymphoma (DLBCL) based on level A evidence.
- The panel further emphasized there are several unresolved issues with endpoints used to assess the clinical utility of maintenance rituximab, as selected endpoints may have differing relevance in different histologies (e.g., mantle cell lymphoma). Future trials addressing the role of maintenance rituximab should clearly define and emphasize endpoints based on histology.
- The panel could not make any recommendations relative to dose, frequency, and duration of rituximab as maintenance therapy.

### Ofatumumab

Ofatumumab is a fully human anti-CD20 antibody that is FDA-approved in combination with chlorambucil, for the treatment of previously untreated patients with CLL for whom fludarabine-based therapy is considered inappropriate. The approval was based on the results of a multicenter randomized open-label trial that demonstrated improved PFS with ofatumumab in combination with chlorambucil as compared to single-agent chlorambucil [101].

Lymphoma Panel Recommendations:

- The panel had no specific recommendation for ofatumumab as the results were not viewed as providing any significant clinical advantages over rituximab. Ofatumumab is currently approved in combination with chlorambucil for front-line therapy of CLL.

### Obinutuzumab

Obinutuzumab is a humanized, glyco-engineered type 2, anti-CD20 antibody that is FDA-approved for use in combination with chlorambucil for the treatment of patients with previously untreated CLL. The approval was based on demonstration of an improvement in PFS in a randomized, open-label, multicenter trial comparing obinutuzumab in combination with chlorambucil to chlorambucil alone in patients with previously untreated CD20-positive CLL. The study also included a rituximab in combination with chlorambucil arm [102].

Lymphoma Panel Recommendations:

- The panel had no specific recommendation for obinutuzumab for lymphoma as the results in this disease, as opposed to CLL, were not viewed as providing any significant clinical advantages over rituximab.

### Alemtuzumab

Alemtuzumab is a recombinant DNA-derived humanized IgG1 kappa anti-CD52 monoclonal antibody indicated as a single agent for the treatment of B cell CLL. Alemtuzumab was initially FDA-approved in 2001 under accelerated approval and subsequently to regular approval based on an international, multicenter trial in 297 previously untreated CLL patients randomized to either alemtuzumab or chlorambucil [103]. The PFS was significantly longer in the alemtuzumab arm; no differences in survival were observed.

Lymphoma Panel Recommendations:

- Alemtuzumab significantly impairs most important immunologic effectors and potentially impairs the utility of other immunotherapeutics.
- CD52 is expressed by approximately half of all peripheral T cell lymphomas, and alemtuzumab has been used alone and in combination with conventional chemotherapy in their treatment. However, as with CLL, there is significant concern over toxicity and immunosuppression.

## Other lymphoma immunotherapies

### Lenalidomide

Lenalidomide, a thalidomide analogue, is an immunomodulatory agent with antiangiogenic and antineoplastic properties. Lenalidomide is FDA-approved for the treatment of mantle cell lymphoma (MCL) that has relapsed or progressed after two prior therapies, one of which included bortezomib. The approval of lenalidomide for MCL was based on a multicenter, single-arm, open-label trial of single-agent lenalidomide in 134 patients whose MCL had relapsed after or were refractory to bortezomib or a bortezomib-containing regimen [104]. Treatment with lenalidomide resulted in an ORR of 26%; the median duration of response was 16.6 months. The combination of lenalidomide plus rituximab (LR) has been investigated as initial therapy in MCL [105]. In a single-group, multicenter, phase 2 study, 38 patients with untreated MCL received lenalidomide (20 mg/day x 21 days of a 28-day cycle) as induction therapy for 12 cycles. Rituximab was administered once weekly for the first 4 weeks and then once every other cycle until disease progression. The most common grade 3 or 4 adverse events were neutropenia (50%), rash (29%), thrombocytopenia (13%), an inflammatory syndrome ( 11%), anemia (in 11%), serum sickness (in 8%), and fatigue (in 8%). At the median follow-up of 30 months, the overall response rate in evaluable patients was 92%, and the CR rate was 64%. Median PFS had not been reached at the time of this report. The 2-year PFS and OS were estimated to be 85% and 97%, respectively. A response to treatment was associated with improvement in quality of life [105].

In a multicenter phase II/III study, DLBCL patients were stratified by germinal center B cell-like (GCB) versus non-GCB subtype, then randomized 1:1 to receive lenalidomide or investigator’s choice (IC) chemotherapy until progressive disease, unacceptable toxicity, or voluntary withdrawal [106]. Patients with GCB or non-GCB DLBCL treated with lenalidomide had similar ORR, but the data suggested greater improvements in PFS and OS with lenalidomide versus IC in the non-GCB patients, particularly the ABC subtype. In the Alliance phase II trial, patients with relapsed follicular lymphoma (FL) were randomized to rituximab alone or lenalidomide alone or LR [107]. The rituximab-alone arm was discontinued as a result of poor accrual. The ORR was 53% (CR = 20%) and 76% (CR = 39%) for lenalidomide alone and LR, respectively (*P* = 0.029). Patients were treated until time of progression. At the median follow-up of 2.5 years, median time to progression was 1.1 years for lenalidomide alone and 2 years for LR (*P* = 0.0023).

Lymphoma Panel Recommendations:

- It was the consensus opinion that lenalidomide as a single agent has clinical activity in relapsed MCL and that LR was an option as initial therapy in untreated MCL based on level B evidence.
- It was the consensus opinion that lenalidomide has clinical activity in DLBCL based on level B evidence.
- The lenalidomide dose of 25 mg used in DLBCL is higher than clinicians are accustomed to using in CLL; however, the risk of toxicity and clots/thrombosis is decreased for lymphoma patients. For patients without standard risk factors for deep vein thrombosis, the panel suggested giving low-dose aspirin.
- The panel felt that clinical endpoints were needed to better define the duration of therapy for LR in FL.

### Interferon (IFN)-α-2b

IFN-α-2b belongs to family of interferons, which are naturally occurring small proteins and glycoproteins produced and secreted by cells in response to viral infections and to synthetic or biological inducers. Interferons exert their effects through a complex sequence of intracellular events including the induction of certain enzymes, suppression of cell proliferation, and augmentation of the specific cytotoxicity of lymphocytes for target cells [108]. IFN-α-2b is FDA-approved for the initial treatment of clinically aggressive follicular NHL in conjunction with anthracycline-containing combination chemotherapy in patients 18 years of age or older. This approval was based on a randomized, controlled trial that evaluated the safety and efficacy of IFN-α-2b in conjunction with a combination of cyclophosphamide, doxorubicin, and teniposide (CHVP) as initial treatment in patients with clinically aggressive, large tumor burden, stage III/IV follicular NHL [109]. Patients were randomized to CHVP alone or CHVP plus IFN-α-2b at 5 million IU subcutaneously three times weekly for the duration of 18 months. The group receiving the combination of IFN-α-2b plus CHVP had a significantly longer PFS (2.9 years versus 1.5 years, *P* = 0.0001). After a median follow-up of 6.1 years, the median survival for patients treated with CHVP alone was 5.5 years while median survival for patients treated with CHVP plus IFN-α-2b had not been reached (*P* = 0.004). IFN-α also has documented single agent activity against multiple subtypes of relapsed NHL [110–112]. Direct injection of IFN-α into lymphoma lesions can often lead to their regression, suggesting that efficient delivery of IFN-α to tumors might be a useful approach to treating lymphomas [113, 114]. To enable delivery of IFN-α to lymphoma cells, with anti-CD20 antibody-IFN-α fusion proteins have been developed which show potent anti-lymphoma effects in pre-clinical models [115, 116].

Recent evidence has also indicated that spontaneous activation of the stimulator of IFN genes (STING) pathway within tumor-resident DCs leads to type I IFN production and adaptive immune responses against tumors [117].

Lymphoma Panel Recommendations:

- The panel commented that IFN-α-2b is currently not commonly used in the treatment of NHL, and its indication came prior to the introduction of rituximab. As such, its use should follow label indications strictly or in the context of a clinical trial. However, other novel means of targeting IFN-α activities to tumor sites to treat lymphomas and other cancers are important areas of investigation.

### Emerging immunotherapies in lymphoma

There have been recent reports of several forms of immunotherapy under clinical investigation for the treatment of lymphoma that have demonstrated clinical efficacy. As many of these treatments are likely to receive FDA approval in the coming years, the panel unanimously agreed that a brief overview of these modalities and clinical data related to them would be of value to the practicing oncologist. During the preparation of this manuscript, nivolumab received FDA approval for the treatment of classical Hodgkin lymphoma (HL) that has relapsed or progressed after autologous HSCT and post-transplantation treatment with brentuximab vedotin. The subsequent section concerning checkpoint blockade therapy was updated to reflect this approval.

### Vaccines

There have been several trials evaluating the use of vaccines in the treatment of lymphoma with one study validating the vaccine approach by demonstrating improvement of disease-free survival in a randomized, controlled clinical trial [118], while others have reported varying levels of success [119, 120]. As T cell activation is critical to a clinically relevant immune response, there is a potentially a significant role for vaccines in the treatment of lymphoma, particularly in combination with other modalities. For vaccines to have a more significant role, there is great need for new antigens, but unfortunately very few true tumor specific antigens in lymphoma are known. Genome sequencing in context of HLA binding permits the identification of large numbers of neoantigens to which vaccines may be developed [121]. The failure of vaccines may be due in large part to an immunosuppressive microenvironment, which may be secondary to past treatments or the inherent biology of the lymphoma. As such, there is a need to further understand vaccine efficacy in association with the microenvironment and develop biomarkers which will permit us to identify subsets of patients or specific lymphomas that more likely to benefit from immunotherapy in general.

### Cellular therapy

There are a variety of cellular therapies that have recently demonstrated clinical efficacy in lymphomas. These therapies include partially HLA-matched third-party Epstein-Barr virus (EBV)-specific cytotoxic T lymphocytes (CTLs), marrow and tumor-infiltrating lymphocytes (MIL/TIL), NK cells, and most prominently genetically-engineered T cells, particularly CAR T cells targeting CD19 [122–125].

### Third-party EBV-specific CTLs

- There is an increasing number of studies demonstrating that allogenic donor or “off-the-shelf” third-party CTLs specific for EBV can be used safely and successfully to treat EBV-associated lymphomas [122, 126].
- One donor can be used to generate antigen-specific T cells that can be infused into multiple recipients making them readily and immediately available to treat patients.

### CAR T cell therapy

- In contrast to the relatively large numbers and successes in ALL and CLL, the use of CAR T cell therapy for the treatment of lymphoma is limited and has short follow-up times. However, the available data are encouraging with anecdotal data demonstrating responses in refractory and relapsed FL, DLBCL, and MCL [125].
- There have been two major categories of toxicities associated with this therapy: cytokine release syndrome (CRS) and neurologic toxicities, which may be related. Classical CRS is associated with high fever, tachycardia, hypotension, tachypnea and hypoxia, and it can be life-threatening [127]. CRS is associated with elevated circulating levels of several cytokines including IL-6 and IFN-γ, and uncontrolled studies demonstrate that immunosuppression using tocilizumab, an anti-IL-6 receptor antibody, with or without corticosteroids can reverse the syndrome. Neurologic toxicities observed with CAR-T cell therapy have included aphasia, dysphasia, tremor, and seizure. These have generally been transient, lasting up to 2 weeks, but they also can be life threatening.
- A significant practical obstacle in making this technology more broadly accessible is that the screening and production process requires several weeks. However, improving culture techniques have reduced production times to less than two weeks. There has also been increased standardization and automation in manufacturing in preparation to provide CAR T cells to large numbers of patients as commercial products.
- A key scientific question for this field is why the response rates for lymphomas are so variable and not as high as those observed in ALL. One hypothesis is that it may relate to host T cell function. A highly related question is what is the optimal T cell phenotype for response and persistence, which appears to correlate with duration of response [128].
- The majority of trials have targeted CD19, but CAR T cells targeting a number of other lymphoma antigens (e.g., CD22, CD28, CD30, ROR1) are in early clinical trials or in development [129].
- This technology is very promising as a salvage regimen. However, the immediate question is its role and timing among the many emerging choices for refractory and relapsed lymphomas. There will be increased utilization of this therapy and earlier consideration for it as a treatment option, as long as it proves to be safe (see toxicities), and especially if it is shown to be a “once and done” option, which has been observed in ALL.

### Bispecific T cell Engager (BiTE) molecules

- Blinatumomab is FDA approved for the treatment of relapsed or refractory B cell precursor ALL. It recruits cytotoxic T cells to target tumor B cells by linking the CD3 and CD19 antigens.
- In a phase II clinical trial, treatment of heavily pretreated patients with relapsed/refractory DLBCL with blinatumomab showed an acceptable safety profile and resulted in objective (ORR = 43%) and durable responses [130].
- CRS and neurotoxicity have been observed with blinatumomab.

### Checkpoint blockade

Tumor immune evasion pathways have been most thoroughly studied in solid tumors; however, emerging data have demonstrated that malignancies of hematopoietic origin are also able to co-opt their local environment in order to escape immune attack. Activated T cells upregulate negative costimulatory receptors, such as PD-1 and cytotoxic lymphocyte antigen-4 (CTLA-4) [131]. Engagement of PD-1 or CTLA-4 with ligands expressed on tumor cells or professional antigen presenting cells results in down-regulation of effector T cell function and represents a potent mechanism of immune evasion across a number of human cancers. Antibodies which block PD-1/PD-L1 interactions have demonstrated that in select subtypes of HL and NHL, the PD-1 ligands are over-expressed due to a genetic amplification of the loci encoding them [132–134]. Other mechanisms of PD-L1 over-expression in lymphomas have also been elucidated. Reports from early-phase clinical trials of PD-1 blockade have demonstrated remarkable effectiveness in HL and also appear active against some NHLs.

- Preclinical studies suggested that Reed-Sternberg cells exploit the PD-1 pathway to evade immune detection. In classic HL, alterations in chromosome 9p24.1 increase the abundance of the PD-1 ligands, PD-L1 and PD-L2, and promote their induction through Janus kinase (JAK)-signal transducer and activator of transcription (STAT) signaling [133]. Based on these observations, nivolumab, a PD-1-blocking antibody, was investigated in 23 patients with relapsed or refractory HL [1]. An objective response was reported in 20 patients (87%) per investigator assessment, including 17% with a CR and 70% with a PR; the remaining 3 patients (13%) had stable disease. The rate of PFS at 24 weeks was 86%. In a subsequent phase II study, nivolumab was investigated in relapsed/refractory classical HL patients. Results from this study illustrated an ORR of 66% per independent review; CR and PR rates were 8.8% and 57.5%, respectively. At the time of the database lock for this study, 62% of responders remained in response with a median follow-up time of 8.9 months [135]. Based on results from these studies, nivolumab was granted accelerated approval by the FDA on May 17, 2016 for patients with classical HL that has progressed following autologous HSCT and brentuximab vedotin.
- In trials with small numbers of patients, responses have been observed with CTLA-4 or PD-L1 blockade in FL and DLBCL [136–138].
- With virally-associated lymphoid tumors (e.g., EBV+ DLBCL), most all have increased PD-L1 on tumor cells [132, 139]. Therefore, determining biological heterogeneity may allow for the identification of subsets susceptible to PD-1 blockade.
- Trials of PD-1 blockade in lymphoma show toxicities similar to those reported in solid tumors.
- Although results are very preliminary, the efficacy of PD-1 blockade as a single agent rivals that of chemotherapy in heavily pretreated patients, and consideration should be given to studying these agents earlier in the disease course and in combination with conventional agents as well as other forms of immune therapy, particularly vaccines.

### Issues related to immunotherapy research in lymphoma

- The panel thought it was essential to try to learn as much as possible from every patient who enters a trial. Specifically, it is important to obtain tumor and blood samples from every patient. Patient samples are critical for evaluation of:
  - ▪ Tumor microenvironment
  - ▪ Systemic immune responses
  - ▪ Tumor and host mutational burden
  - ▪ Tumor antigens
  - ▪ T cell receptor (TCR) repertoire (locally and systemically) and clonal T cell expansion within tumors
- The panel suggested that pretreatment biopsies should be mandatory for participation in clinical trials and strongly suggested that follow-up biopsies be obtained at the time of relapse in order to understand mechanisms of resistance. In order to do so, there is a need for funding for sample banks.
- One of the major problems that will need to be addressed is how to design and prioritize immunotherapy trials with so many competing agents and modalities. The panel suggested that a profile/portfolio of collaborative immune studies with uniform approaches to immune monitoring be established in order to develop a large dataset.
- It was emphasized that the majority of trials will be developed and conducted with pharmaceutical companies. Thus, it is imperative for industry to share the biologic data that result from these studies. A collaborative effort is needed to bring together different interests and strengths in order to develop important trial(s) and generate robust data. There is a strategic advantage to a pharma-academia partnership. Such a partnership will result in faster completion of trials with greater scientific depth and would be a “win-win” situation for both entities.
- In thinking about developing immunotherapeutic trials in lymphoma, the extraordinary heterogeneity of diseases, as well as within disease heterogeneity, must be recognized. Therefore, it is essential to study the quality and pathologic evidence of immune infiltration, which is the genetic basis for the perturbation and modulation of regulators. This understanding of the biology and heterogeneity must be linked to specific treatments for diseases

## Acute leukemia

Acute myeloid leukemia (AML) and ALL remain formidable clinical challenges largely due to resistance of leukemia to current therapies and leukemia relapse [140, 141]. Negative immune regulatory mechanisms present in acute leukemia may contribute to the development of a suppressive microenvironment that protects leukemic cells from immune destruction. Furthermore, immune cell abnormalities including impaired NK cell activity and increased frequency and immunosuppressive functions of regulatory T cells have been described in patients with acute leukemia [142, 143].

During the past four decades, allogeneic HSCT following both myeloablative and non-myeloablative (reduced intensity) conditioning regimens has been established as a standard and curative treatment option for acute leukemia [144–146]. The anti-leukemic activity of allogeneic HSCT relies not only on the effects of high dose chemotherapy or irradiation given during the conditioning regimen, but also on the immune-mediated graft-versus-leukemia effect [147–149]. The use of cytokines or pharmacologic agents to restore immune cell effector functions and, by extension, anti-leukemic effects represent other immunotherapeutic approaches that have been used in leukemia treatment [150–153].

Several non-transplant immunotherapeutic strategies are currently being evaluated in numerous clinical trials. These include among others the use antibody based therapies, immune checkpoint inhibitors, CAR T cells, NK cells, and vaccine based therapies.

### Current immunotherapies in acute leukemia

#### Blinatumomab

Blinatumomab is a bispecific CD19-directed CD3 T cell engager that activates endogenous T cells when bound to the CD19-expressing target cell. Blinatumomab was studied in patients with MRD-positive B-lineage ALL after intensive chemotherapy and in follow-up studies in patients with relapsed and refractory Philadelphia chromosome-negative B cell ALL [154–157]. The role of blinatumomab in is currently being evaluated in a Phase III clinical trial (ECOG-ACRIN Cancer Research Group, NCT02003222) in patients with newly diagnosed BCR-ABL-Negative B Lineage ALL.

Blinatumomab was granted accelerated approval by the FDA on December 3, 2014 for the treatment of Philadelphia chromosome-negative relapsed or refractory B cell precursor ALL [155, 158]. The basis of the approval was a single arm trial with 185 evaluable adults. Blinatumomab was administered in patients with refractory/relapsed ALL by continuous infusion for 4 weeks of a 6-week cycle. Up to two cycles were used for induction and three cycles for consolidation. The complete remission rate was 33% (95% CI: 27%–41%) with 2 cycles of treatment with blinatumomab, and the median duration of response was 6.7 months (range, 0.46–16.5 months). Median OS was 6.1 months (95% CI: 4.2–7.5 months). A minimal residual response was achieved by 31% (95% CI: 25%–39%) of all patients.

Safety was evaluated in 212 patients with relapsed or refractory ALL treated with blinatumomab [158]. The most common adverse reactions (≥20%) were pyrexia, headache, peripheral edema, febrile neutropenia, nausea, rash and tremor. Elevated transaminases were the most common (>10%) laboratory abnormalities related to blinatumomab. A neurological toxicity occurred in approximately 50% of patients. CRS was reported in 12% of the patients (grade 3 ≥ CRS syndrome in 2%). Blinatumomab administration was interrupted in 32% of the patients and discontinued in 17%. The most common reasons for interruption were neurologic toxicity and CRS. The most common reasons for permanent withdrawal included neurologic toxicity and sepsis.

Leukemia Panel Recommendations:

- The panel recommended the use of blinatumomab for patients with relapsed or refractory ALL based on level B evidence.

### Emerging therapies

#### Monoclonal antibodies in acute leukemia

Engagement of mAb with leukemia target antigens can lead to direct apoptosis, CDC, and ADCC [159]. Antigens expressed on leukemia blasts or preferentially expressed on leukemia stem cells including CD33, CD45, CD96, CD123, CD135, CLL-1 and T cell immunoglobulin mucin-3 (TIM-3) represent potential targets for antibody-based therapy in AML [160, 161]. In ALL, CD19, CD20, CD22 and CD52 (among others) represent potential targets [162–164]. A number of monoclonal antibodies are currently being evaluated (Table 1). These include unconjugated monoclonal antibodies and monoclonal antibodies conjugated with cytotoxins.

**Table 1:** Selected monoclonal antibodies in ALL and AML

Selected monoclonal antibodies in ALL		Selected monoclonal antibodies in AML	
Rituximab	anti-CD20 antibody	SGN-CD33A	anti-CD33 pyrrolobenzodiazepine dimer
Ofatumumab	anti-CD20 antibody	AMG 330	anti-CD33 and CD3, bi-specific T cell engager antibody
Epratuzumab	anti-CD22 antibody	MGD006	anti-CD123 and CD3, dual affinity retargeting molecule
Alemtuzumab	anti-CD52 antibody	CSL362	anti-CD123 antibody
Inotuzumab ozogamicin	Monoclonal anti-CD22 immunotoxin	SL-401	diphtheria toxin interleukin-3 fusion protein against CD123
Blinatumomab	bi-specific T cell engager antibody		
Moxetunomab pasudotox	conjugated immunotoxin targeting CD22		

An approach to enhance the efficacy of antibody therapy is the use of BiTE antibodies like blinatumomab mentioned on the previous page. By bridging tumor antigens with T cell receptors, these can direct effector T cells to leukemia blasts target antigens. In recent years, different T cell engaging antibody constructs have been developed. The use of bispecific antibodies that contain CD16 and blast-specific antigens can enhance NK cell mediated ADCC. Furthermore, anti-KIR antibodies to block inhibitory KIR receptors can be used to enhance NK cell cytotoxicity [165, 166].

Several phase I and phase II antigen-specific antibody clinical trials are currently in development for the treatment of acute leukemia. Epratuzumab, an unconjugated humanized monoclonal antibody, binds to the third extracellular domain of CD22. Epratuzumab was evaluated by the Children’s Oncology Group as single agent and as part of a chemotherapy backbone in 114 relapsed ALL patients either weekly or twice weekly [167, 168]. The CR rates were similar to both arms (65% and 66%) but were not significantly higher than those observed historically without epratuzumab. The addition of epratuzumab was well tolerated, with a similar toxicity profile to that observed with the re-induction chemotherapy platform regimen alone. While CR rates were not improved compared to historical controls treated with chemotherapy alone, there was a non-significant trend towards improvement in MRD response with the addition of epratuzumab to re-induction chemotherapy.

In a recent SWOG study (31 patients, median age: 41 years old), the addition of epratuzumab to the combination of clofarabine and cytarabine in adults with relapsed/refractory pre-B ALL was evaluated [169]. The response rate (CR plus CR without count recovery) was 52%, significantly higher than the previous trial with clofarabine/cytarabine alone, where the response rate was 17%. The median OS was 5 months (95% CI: 3–9 months).

Rituximab, a chimeric anti-CD20 antibody, has been evaluated with combination chemotherapy for patients with B cell ALL demonstrating event-free survival (EFS) as well as OS benefit and molecular CR rates [170–172]. A multicenter randomized trial compared a pediatric-inspired protocol to the same regimen plus rituximab in patients newly diagnosed with CD20-positive Ph-negative B-Cell precursor ALL (105 in the rituximab arm and 104 in the control arm) [172]. Median age was 40 years. Both randomization arms were well balanced for pretreatment characteristics. CR rate was 92% and 91% in rituximab and control arm, respectively. With a median follow-up of 30 months, patients treated in the rituximab arm had a lower cumulative incidence of relapse (CIR) (2-year CIR, 18% [95% CI: 10–26] vs. 30.5% [95% CI: 21–40] in control arm; *P* = 0.02), while no significant difference was observed regarding non-relapse mortality between both arms. This translated into longer EFS in patients treated in the rituximab arm (2-year EFS, 65% [95% CI: 56–75] vs 52% [95% CI: 43–63] in control arm; *P* = 0.038). When censoring patients who received allogeneic HSCT in first CR at transplant time, EFS and OS were longer in the rituximab arm.

Ofatumumab is an anti-CD20 antibody that targets a membrane proximal small-loop epitope on the CD20 molecule. Similar to rituximab, ofatumumab was combined with ALL chemotherapy in a phase II clinical study. The CR rate was 96%; and 96% of patients achieved MRD negativity. The one year CR duration and OS were 90% and 88% respectively [173, 174].

Alemtuzumab is a humanized monoclonal antibody directed against the CD52 antigen present on the surface of immune cells. Alemtuzumab has limited activity as single agent in patients with ALL [175]. In a phase I study by CALGB, alemtuzumab was administered post-remission for eradication of MRD. The addition of alemtuzumab resulted in reduction of MRD, but it was also associated with viral infections [176]. Based on these results an expansion phase was completed which may confirm the preliminary results.

Inotuzumab ozogamicin is a humanized anti-CD22 antibody conjugated to calicheamicin. In a recent phase 3 trial patients with relapsed or refractory ALL were randomized to inotuzumab ozogamicin or standard of care intensive chemotherapy [177]. The rate of CR was significantly higher in the inotuzumab ozogamicin group than in the standard-therapy group (80.7% vs. 29.4%, *P* < 0.001). Among the patients who had CR a higher percentage in the inotuzumab ozogamicin group had results below the threshold for minimal residual disease. The duration of remission was longer in the inotuzumab ozogamicin group (median, 4.6 months vs. 3.1 months; *P* = 0.03). In the survival analysis, which included all 326 patients, PFS was significantly longer in the inotuzumab ozogamicin group (median, 5.0 months vs. 1.8 months; *P* < 0.001); the median OS was 7.7 months (95% CI: 6.0 to 9.2) versus 6.7 months (95% CI: 4.9 to 8.3), and the hazard ratio was 0.77 (97.5% CI, 0.58 to 1.03) (*P* = 0.04). Inotuzumab ozogamicin has already received FDA Breakthrough Therapy Designation for patients with relapsed or refractory ALL.

CD33 is a myeloid differentiation antigen that is broadly expressed on AML blasts. Antibody-based therapeutics in AML have targeted CD33 for many years. Gemtuzumab ozogamicin is a targeted antineoplastic agent consisting of a recombinant anti-CD33 humanized antibody linked to N-acetyl-γ-calicheamicin. Gemtuzumab ozogamicin was approved in 2000 by the FDA for use in patients age 60 or older with CD33 + AML in first relapse [178, 179]. However, in 2010 gemtuzumab ozogamicin was voluntary withdrawn after a phase 3 trial (SWOG S0106) in newly diagnosed AML based showed a trend toward an increased mortality in the gemtuzumab ozogamicin arm [180]. A recent meta-analysis from five randomized controlled trials incorporating gemtuzumab ozogamicin demonstrated a significant survival benefit for patients with favorable and intermediate cytogenetic characteristics suggesting of reassessing the status of gemtuzumab ozogamicin [181]. Given the potential of targeting CD33, new CD33 monoclonal antibodies are in development in clinical trials and CD33 has been incorporated in bi-specific antibodies such as CD33/CD3 or CD33/CD123.

SGN‑CD33, a CD33-directed antibody conjugated to two molecules of a pyrrolobenzodiazepine dimer, has been evaluated as monotherapy in patients with CD33-positive AML with CR + CRi rates up to 60% in treatment naïve patients and in combination with hypomethylating agents [182, 183].

CSL362 is a fully humanized anti-CD123 monoclonal antibody, engineered for greater ADCC by higher affinity for NK cell CD16. An early report from a phase I clinical trial of 25 AML high-risk patients who achieved CR indicated that the antibody was safe and well tolerated [184].

Leukemia Panel Recommendations:

- The panel recommended the use of rituximab in patients with CD20-positive Ph-negative B-Cell precursor ALL based on Level A evidence.
- All panelists agreed that mAbs should be evaluated in clinical trials in the relapsed/refractory setting, in newly diagnosed acute leukemia patients with combination chemotherapy, and in high-risk patients in complete remission.

### Immune checkpoint blockade

Surface expression and inhibitory functions of checkpoint inhibitors are up-regulated in T cells present in the tumor microenvironment. While the presence of these inhibitory receptors on T cells is physiologically necessary to regulate cellular activation, their overexpression in disease leads to dysfunction of T cells and other immune effector cells [185–187]. In the setting of cancer, chronic overexpression of checkpoint molecules results in T cell dysfunction and impairs anti-tumor immunity.

The PD-1/PDL-1 pathway has been investigated in preclinical leukemia mouse models. The PD-1 receptor biology, expression of PD-1 on the surface of activated immune cells and its ligands, PD-L1 and PD-L2, on leukemic blasts and functional consequences of antibody-based or pharmacologic blockade of PD-1 are under investigation in acute leukemia [188–190]. PD-1 blockade can restore anti-leukemia T cell functions and thus may offer therapeutic advantages in acute leukemia. Given the acceptable tolerability, pre-clinical rationale, and immunological activity of PD-1/PD-L1 blockade, clinical trials of anti-PD-1 mAbs are underway in acute leukemia patients [191]. Several other checkpoint molecules are known [192, 193] and are under investigation in acute leukemia, including CTLA-4, TIM-3, lymphocyte activation gene-3 (LAG-3), and B and T cell lymphocyte attenuator (BTLA).

Leukemia Panel Recommendations:

- The panel was in consensus that there is preclinical rationale for consideration of clinical trials for immune-checkpoint blockade in acute leukemia.
- The panel identified the following clinical settings for evaluation of immune-checkpoint blockade in acute leukemia: patients with MRD, high-risk patients, and elderly patients.

### CAR T Cells for the treatment of acute leukemia

Adoptive transfer of T cells engineered to express a CAR has emerged as a powerful immunotherapy. CAR-based therapies have been studied mainly in patients with B cell ALL. As described above, CAR are synthetic molecules consisting of an extracellular antigen-binding domain fused via a spacer region to intracellular signaling domains that are capable of activating T cells. CARs engage molecular structures independent of antigen processing by the target cell and independent of MHC [194, 195]. Over the course of years, several generations of CAR-T cells with different and multiple costimulatory intracellular domains have been developed and tested in clinical trials [80]. First generation CAR include a single T cell stimulatory domain such as CD3-zeta. Second generation CAR add a co-stimulatory domain most typically derived from CD28 or CD137 (4-1BB). Third generation CAR, not yet in clinical trials, include 2 co-stimulatory signals. The later CAR generations with additional intracellular signaling domains have increased the activity by circumventing the T cell’s need for co-stimulatory molecules. The addition of a co-stimulatory domain in the new generation CARs improved the replicative capacity and persistence of modified T cells. Several gene transfer technologies are used to engineer T cells to express CARs including electroporation as well as retroviral and lentiviral vector methods.

Most studies using CARs have focused on hematologic malignancies by targeting CD19 [196]. Multiple clinical trials using other antigens are underway in ALL and AML. Reported clinical trials using CAR T cells differed in the design of the CAR, expression of the CAR on the T cells, conditions of the T cell culture, lymphodepleting strategy, cytokine support for the infused T cells, and timing of CAR T cell infusion with regard to standard therapy such as allogeneic HSCT [80].

High remission rates have been reported in patients with relapsed/refractory ALL treated with CAR T cells with CR rates of 70%-90%. Also, durable remissions were observed without additional therapy [80, 197–200]. In addition, in studies that included patients with prior history of allogeneic HSCT, no graft-versus-host disease was observed. Furthermore, among the different studies, the persistence of CAR-modified T cells varied, which could be related to different CAR design.

Twenty-one children and young adults with ALL were treated in a phase I clinical study with CD19-CAR incorporating an anti-CD19 single-chain variable fragment plus TCR zeta and CD28 signaling domains. Among 20 patients with B-ALL, the CR rate was 70% (95% CI: 45.7–88.1), with 12 of 20 patients with B-ALL achieving MRD-negative complete response (60%; 95% CI: 36.1–80.9). OS at a median follow-up of 10 months was 51.6% at 9.7 months and beyond. Leukemia-free survival of 12 patients who achieved an MRD-negative CR was 78.8% beginning at 4.8 months [201].

Sixteen patients with relapsed or refractory B cell ALL were treated in a phase I clinical study with autologous T cells expressing the 19-28z CAR specific to the CD19 antigen. The overall CR rate was 88%, which allowed transition of most of these patients to allo-HSCT. This therapy was as effective in high-risk patients with Philadelphia chromosome–positive (Ph^+^) disease as in those with relapsed disease after previous allogeneic HSCT [202].

Thirty children and adults with relapsed or refractory ALL were treated with autologous T cells transduced with a CD19-directed CAR lentiviral vector that included the 4-1BB costimulatory signal (CTL019). CR was achieved in 27 patients (90%), including 2 patients with blinatumomab-refractory disease and 15 who had undergone stem cell transplantation. At 6 months, the probability that a patient would have persistence of CTL019 was 68% (95% CI: 50 to 92), and the probability that a patient would have relapse-free B cell aplasia was 73% (95% CI: 57 to 94) [199]. Sustained remission was achieved with a 6-month EFS rate of 67% (95% CI: 51–88%) and an OS rate of 78% (95% CI: 65–95%).

CAR T cell therapies are associated with neurologic toxicities including encephalopathy and seizures, which could be reversible. CRS is a common and potentially life-threatening toxicity associated with CAR T cell therapy [199, 201, 202]. It is associated with elevation of cytokines including interleukin-2 receptor ɑ (IL-2Rɑ), IL-6, IL-10 and IFN.

IL-6 inhibitors have been used to ameliorate CRS. The development of CRS may correlate with response to therapy; patients who develop CRS often respond to therapy. Moreover, the severity of CRS may correlate with the tumor burden. Furthermore, B cell aplasia is an expected on-target result of CD19-directed therapies and has served as a useful surrogate to determine the persistence and effectiveness of CD19-directed CAR-T cells.

The FDA has granted breakthrough therapy status to CTL019, an investigational CAR therapy for the treatment of pediatric and adult patients with relapsed/refractory ALL. CTL019 -engineered T cells express a CAR in which the T cell activation signal is provided by the CD3-zeta domain, and the costimulatory signal is provided by the CD137 (4-1BB) domain.

Leukemia Panel Recommendations:

- The panel recognizes the lack of level A evidence regarding the use of CAR T cells. However, all panelists agreed that there are very encouraging clinical data regarding the use of CAR T cells. With current-ongoing studies using CAR T cells, the panel concluded that this treatment modality is among the most promising immunotherapies for the treatment of ALL.
- The panel recommended the development of guidelines for management of complications with CAR T cells.

### Natural killer cells for the treatment of acute leukemia

NK cells contribute to anti-leukemic immune responses both by exerting direct cytotoxic activity and activating other immune cells [203, 204]. In patients with acute leukemia, there is impairment of NK cell activity mediated by several mechanisms including reduced expression of activating receptors and reduced NK cell cytokine secretion capacity [205–208]. Furthermore, low expression of NK ligands on leukemia blasts and production of soluble immunosuppressive factors contribute to impaired NK cell activity [209, 210]. Different strategies for NK cell-based cancer immunotherapy have been studied including the administration of anti-KIR antibodies that can block NK cell inhibitory recognition, the use of cytokines including IL-15 and IL-2, adoptively transferred HLA-haploidentical NK cells and the use of NK cell lines [203, 204, 211–213].

In particular, adoptive therapy using haploidentical NK cells in combination with cyclophosphamide and fludarabine to lymphodeplete the recipient and facilitate expansion of the allogeneic NK cells with interleukin (IL)-2 administration induced CR in patients with relapsed and refractory AML. Furthermore, the use of IL-2-diphtheria fusion protein (IL2DT) to deplete regulatory T cells, led to improvements in rates of in vivo NK-cell expansion and AML remissions (CR rate 53%) compared with patients that did not received IL2DT (CR rate 21%, *P* = 0.02) [212, 214].

Leukemia Panel Recommendations:

- The panel recognizes the lack of level A evidence regarding the use of NK cells. However, all panelists agreed that there are very encouraging clinical data regarding the use of NK cells.
- The panel identified the following clinical settings as high priority for evaluation of NK cell based therapies in acute leukemia: refractory patients, patients with MRD, and high-risk patients.

### Vaccines

To stimulate anti-leukemic responses active immunizations through vaccination has been explored for patients with acute leukemia. Several types of vaccines have been used for patients with acute leukemia; peptide vaccines, granulocytes macrophage-colony stimulating factor vaccines, and DC vaccines. Moreover, different maturation protocols have been applied for the generation of these vaccines. Pre-clinical and clinical trials involving vaccination with peptides derived from different leukemia antigens, including Wilms’s tumor 1 (WT1), PR1, or hyalunoric-acid-mediated motility (RHAMM), demonstrated immunogenicity and clinical safety [215–221].

Leukemia Panel Recommendations:

- The panel recognizes the lack of level A evidence regarding the use of vaccines in acute leukemia.
- The panel agreed that clinical trials using vaccines in acute leukemia have established their safety and immunogenicity.
- The panel identified the following clinical settings as high priority for evaluation of vaccines in acute leukemia: patients with MRD and high-risk patients.
- More studies are needed to investigate the effects of chemotherapy on the immune system as intensive chemotherapy may impair the immune responses to vaccination.

### Issues related to immunotherapy research in acute leukemia

The methods and timing needed to evaluate response to immune therapies in acute leukemia are not established, and, similar to solid tumors treated with immune therapies, endpoints such as CR rates and PFS may need to be re-evaluated.

Leukemia Panel Recommendations:

- There are insufficient data to evaluate whether current criteria for clinical response in acute leukemia are adequate for evaluation of response to immune therapies and whether immune related response criteria as in the setting of solid tumors will be useful in acute leukemia.
- The panel recommended detailed immune monitoring in ongoing clinical trials that are tailored to specific immune therapies.
- The timing of the evaluation of response is not known and should be tailored to the specific immune therapy.
- Standards of immune monitoring in acute leukemia should be established and include among others analysis of bone marrow microenvironment and circulating immune cells.
- Information gained from all on-going clinical trials may provide the foundation for the generation of new response criteria and the proper timing of response evaluation.

## Additional files

Additional file 1: Cancer Immunotherapy Guidelines for Hematologic Malignancies Roster. (DOCX 13 kb)
Additional file 2: Comments Recieved during Review. (DOCX 14 kb)
Additional file 3: Annotated Bibliography (Myeloma), Annotated Bibliography (Lymphoma), and Annotated Bibliography (Leukemia). (ZIP 1812 kb)

## Abbreviations

ADCC: Antibody-dependent cell mediated cytotoxicity
ADCs: Antibody-drug conjugates
ALL: Acute lymphoblastic leukemia
AML: Acute myeloid leukemia
BiTE: Bispecific T cell engager
BTLA: B and T cell lymphocyte attenuator
CALGB: Cancer and leukemia group B
CAR: Chimeric antigen receptor
CDC: Complement-dependent cytotoxicity
CHOP: Cyclophosphamide, adriamycin, vincristine, prednisone
CHVP: Cyclophosphamide, doxorubicin, and teniposide
CIR: Cumulative incidence of relapse
CLL: Chronic lymphocytic leukemia
CR: Complete response
CRS: Cytokine release syndrome
CTL: Cytotoxic T lymphocyte
CTLA-4: Cytotoxic lymphocyte antigen-4
CVP: Cyclophosphamide, vincristine, and prednisone
CyBorD: Cyclophosphamide, bortezomib, and dexamethasone
DC: Dendritic cell
DLBCL: Diffuse large B cell lymphoma
EBV: Epstein-barr virus
EFS: Event-free survival
Elo-Rd: Elotuzumab with lenalidomide plus dexamethasone
FDA: Food and drug administration
GCB: Germinal center b cell-like
HL: Hodgkin lymphoma
HSCT: Hematopoietic stem cell transplantation
IC: Investigator’s choice
IFN: Interferon
Ig: Immunoglobulin
IL: Interleukin
IL-2Rɑ: Interleukin-2 receptor ɑ
IMiDs: Immune-modulating drugs
JAK: Janus kinase
KRd: Carfilzomib with lenalidomide plus dexamethasone
LAG-3: Lymphocyte activation gene-3
LR: Lenalidomide plus rituximab
mAbs: Monoclonal antibodies
MCL: Mantle cell lymphoma
MGUS: Monoclonal gammopathy of undetermined significance
MIL/TIL: Marrow and tumor-infiltrating lymphocytes
MM: Multiple myeloma
MPT: Melphalan, prednisolone, and thalidomide
MRD: Minimal residual disease
nCR: Near complete response
NHL: Non-hodgkin lymphoma
NK: Natural killer
ORR: Objective response rate
OS: Overall survival
PD-1: Programmed cell death-1
PFS: Progression-free survival
Rd: Lenalidomide plus dexamethasone
RRMM: Relapsed/refractory multiple myeloma
SITC: Society for immunotherapy of cancer
STING: Stimulator of interferon genes
SWOG: Southwestern oncology group
TCR: T cell receptor
TIM-3: T cell immunoglobulin mucin-3
VRd: Bortezomib with lenalidomide plus dexamethasone
XBP-1: X-box binding protein 1
