# Supplementary material for: The Society for Immunotherapy of Cancer consensus statement on immunotherapy for the treatment of hematologic malignancies: multiple myeloma, lymphoma, and acute leukemia
Source: J Immunother Cancer. 2016 Dec 20;4:90. doi: 10.1186/s40425-016-0188-z (PMC5168808; doi:10.1186/s40425-016-0188-z)
Supplement: Additional file 1: — Cancer Immunotherapy Guidelines for Hematologic Malignancies Roster. (DOCX 13 kb) [file 40425_2016_188_MOESM1_ESM.docx]

# APPENDIX I: TASK FORCE PARTICIPANT LIST

**Steering Committee:**

Madhav V. Dhodapkar, MD Yale University

Michael R. Bishop, MD University of Chicago

Michael Boyiadzis, MD, MHSc University of Pittsburgh

**Task Force Participants:**

Rafat Abonour, MD Indiana University School of Medicine

Kenneth C. Anderson, MD Dana-Farber Cancer Institute

Steve M. Ansell, MD, PhD Mayo Clinic Cancer Center

David Avigan, MD Beth Israel Deaconess Medical Center

Lisa Barbarotta, APRN Simlow Cancer Hospital at Yale New Haven

Austin John Barrett, MD National Institutes of Health

Koen Van Besien, MD Weill Cornell Medical College

P. Leif Bergsagel, MD Mayo Clinic

Ivan Borrello, MD Johns Hopkins University

Joshua Brody, MD Icahn School of Medicine at Mount Sinai

Jill Brufsky, Pharm D University of Pittsburgh Cancer Institute

Mitchell Cairo, MD New York Medical College at Maria Fareri Children's Hospital

Ajai Chari, MD Icahn School of Medicine at Mount Sinai

Adam Cohen, MD University of Pennsylvania

Jorge Cortes, MD University of Texas MD Anderson Cancer Center

Stephen J. Forman, MD City of Hope National Medical Center

Jonathan W. Friedberg, MD, MMSc University of Rochester Medical Center

Ephraim J. Fuchs, MD Johns Hopkins University

Steven D. Gore MD Yale University

Sundar Jagannath, MD Icahn School of Medicine at Mount Sinai

Brad S. Kahl, MD University of Wisconsin, Madison

Justin Kline MD University of Chicago

James N. Kochenderfer, MD National Institutes of Health, National Cancer Institute

Larry W. Kwak, MD, PhD University of Texas MD Anderson Cancer Center

Ronald Levy, MD Stanford University

Marcos de Lima, MD Case Western Reserve University

Mark R. Litzow, MD Mayo Clinic

Anuj Mahindra, MD University of California, San Francisco

Jeffrey Miller, MD University of Minnesota

Nikhil C. Munshi, MD Dana-Farber Cancer Institute

Robert Z. Orlowski, MD, PhD University of Texas MD Anderson Cancer Center

Jon M. Pagel, MD Swedish Medical Center

David L. Porter, MD University of Pennsylvania

Stephen J. Russell, MD, PhD Mayo Clinic

Margaret A. Shipp, MD Dana-Farber Cancer Institute

Karl Schwartz Patients Against Lymphoma

David Siegel, MD, PhD Hackensack University Medical Center

Richard M. Stone, MD Dana-Farber Cancer Institute

Martin S. Tallman, MD Memorial Sloan Kettering Cancer Center

John M. Timmerman, MD University of California, Los Angeles

Frits Van Rhee, MD, PhD University of Arkansas for Medical Sciences Myeloma Institute

Edmund K. Waller, MD, PhD, FACP Emory University

Ann Welsh, RN, OCN University of Pittsburgh Medical Center

Michael Werner Patient Advocate
