# Supplementary material for: The Society for Immunotherapy of Cancer consensus statement on immunotherapy for the treatment of hematologic malignancies: multiple myeloma, lymphoma, and acute leukemia
Source: J Immunother Cancer. 2016 Dec 20;4:90. doi: 10.1186/s40425-016-0188-z (PMC5168808; doi:10.1186/s40425-016-0188-z)
Supplement: Additional file 2: — Comments Recieved during Review. (DOCX 14 kb) [file 40425_2016_188_MOESM2_ESM.docx]

# APPENDIX III: COMMENTS

Comments from the open review of this consensus statement.

| **Comment Date** | **Comment** |
| --- | --- |
| **6/21/2016** | Page 19, line 5 should read: reported varying levels of success [117, 118]…. New reference 118 should be added: Freedman A, Neelapu SS, Nichols C, Robertson MJ, Djulbegovic B, Winter JN,Bender JF, Gold DP, Ghalie RG, Stewart ME, Esquibel V, Hamlin P. Placebo-controlled phase III trial of patient-specific immunotherapy with mitumprotimut-T and granulocyte-macrophage colony-stimulating factor after rituximab in patients with follicular lymphoma. J Clin Oncol. 2009 Jun 20;27(18):3036-43. doi: 10.1200/JCO.2008.19.8903. Epub 2009 May 4. PubMed PMID: 19414675; PubMed Central PMCID: PMC3646306. |
| **7/20/2016** | P. 9: Please consider clarifying the sentence regarding antitumor effects of elotuzumab, to note that the effect on enhancing NK activation is a direct effect on the NK cell. P. 9: Please consider further quantifying the statement “improvement in PFS” in the Elo-Rd Phase 3 trial; for example by stating the primary endpoint result (PFS hazard ratio, confidence intervals, p-value). P. 10: Please consider clarifying the rationale for recommendation of top clinical settings for evaluation of ICP-based combinations (currently seems narrow in scope). Please also consider whether maintenance should be included as a specific “top clinical settings for evaluation” of ICP. P 10: Please consider whether tumor-directed mAbs should be included as one of the “top agents” for evaluation in combination with ICP in clinical trials. P. 13: Please consider including a discussion on the need for further research in MRD in the era of I-O therapies. P. 21: Please consider clarifying that the ORR of 87% for the 23 HL patients in the nivolumab Phase 1 nivolumab is per investigator assessment, and the ORR of 66% for the 8 HL patients in the Phase 2 study is per independent review assessment. P. 22: Please consider modifying to “...imperative for industry and academia to share...” P. 26: Please consider a discussion of recent data regarding CTLA-4 blockade post-allogeneic SCT (Davids et al NEJM 2016, 375;2:143). References: Please consider updating reference 55 to the fully published manuscript: Leoshkin et al JCO 2016, 34 (epub ahead of print). |
| **7/20/2016** | The Consensus Statement on Immunotherapy for the treatment of Hematologic Malignancies: Multiple Myeloma, Lymphoma and Acute leukemia perfectly fulfilled the original purpose of the responsible experts of the Society for Immunotherapy of Cancer. The importance of this initiative is huge, as it summarises the state of art of this new era of cancer therapeutics and provides the necessary guidelines to use new cancer treatment modalities involving the immune system of the patient with cancer. The Consensus statement will be well used by the physicians when introducing the new „Immunotherapeutics” in various types and different cases of multiple myelomas, lymphomas and acute leukemias. The Consensus statement serves with up to date information about these diseases and gives a perfect summary of the experiences by now with previously approved agents and related to the novel immunotherapeutics and potential prognostic outcomes. In order to develop the consensus statement, special standards were used that include key components, like transparent process for guideline development and funding, dealing with conflicts of interest, a multidisciplinary panel, rating system for the strength of the evidence, publications and publicity, just to mention the most important ones. There is a detailed list of myeloma panel recommendations, when using the various therapeutic modalities like IMiDs, anti-tumor monoclonal antibodies, emerging immunotherapies, immune activating antibodies, vaccines, adoptive cellular therapies. The issues related to immunotherapy research in myeloma are also informative and helpful. There is a special recommendation for detailed immune monitoring in ongoing clinical trials. The guidelines on lymphoma fit well both into the overall goal and the specific goals. Additionally, there is valuable information on the selection of patients, immunotherapy side effects, monitoring possibilities and combined therapies. The definition of an immunotherapeutic agent is a very useful point of this section. *Although the consensus lists the various forms of anti CD20 antibodies, available for treatment, it would be of importance to bring up more data on the various experiences with these agents, and what is the potential immunological, biochemical explanation for different efficacy. The detailed list of other lymphoma immunotherapies is broad and well explained. The issues related to the immunotherapy research in lymphoma are essential. Acute myeloid leukemia and acute lymphoblastic leukemia remain formidable clinical challenges due to resistance of leukemia to current therapies and leukemia relapse. *Although there is a perfect description on emerging therapies, which is informative and useful, I could not find any outlook for potential handling of relapse mechanisms and the cancer stem cell issue. In summary I congratulate to this highly important work that certainly will help cancerous patient care. |
