# Supplementary material for: The Society for Immunotherapy of Cancer consensus statement on immunotherapy for the treatment of hematologic malignancies: multiple myeloma, lymphoma, and acute leukemia
Source: J Immunother Cancer. 2016 Dec 20;4:90. doi: 10.1186/s40425-016-0188-z (PMC5168808; doi:10.1186/s40425-016-0188-z)
Supplement: Additional file 3: — Annotated Bibliography (Myeloma), Annotated Bibliography (Lymphoma), and Annotated Bibliography (Leukemia). (ZIP 1812 kb) [file 40425_2016_188_MOESM3_ESM.zip › Add/APPENDIX II-Supplementary Biobliography I Cancer Immunotherapy Guidelines Multiple Myeloma.pdf]

Cancer Immunotherapy Guidelines (Myeloma)

An Annotated Bibliography of the Literature (in order of topic)

Society for Immunotherapy of Cancer

November 26, 2014

Table of Contents

TOPIC: Myeloma + Lenalidomide (OR) Pomalidomide (OR) Thalidomide 6

TOPIC: Myeloma + monoclonal antibod* (y or ies) 68

TOPIC: Myeloma + checkpoint blockade or programmed death-1 (PD-1) or PD-L1 or B7-H1 87

TOPIC: Myeloma + oncolytic virus 98

TOPIC: Myeloma + virotherapy 111

TOPIC: Myeloma + dendritic cell vaccine or idiotype vaccine 112

Note: The bibliography is organized in topic order and then in alphabetical order by author. Duplicates and selected references have been removed in this bibliography.

Note: The bibliography is organized in topic order and then in alphabetical order by author. Duplicates and selected references (by Dr. Dhodapkar) have been removed in this bibliography. The searches were conducted on 11/17/14 and 11/21/14 in the sequence and with the limits as follows:

Myeloma Immunotherapy Literature Searches Conducted November 17 and 21, 2014

Search Terms	Date Limits	Query Translation	Date Search Completed	Total Refs Identified	EndNote record numbers	total # dupes	Resulting # of records in bibliography	total # drops	Resulting # of records in bibliography
Myeloma + Lenalidomide (OR) Pomalidomide (OR) Thalidomide	2004-2014	((("multiple myeloma"[MeSH Terms] OR ("multiple"[All Fields] AND "myeloma"[All Fields]) OR "multiple myeloma"[All Fields] OR "myeloma"[All Fields]) AND "lenalidomide"[Supplementary Concept]) OR "pomalidomide"[Supplementary Concept]) OR "thalidomide"[MeSH Terms] AND (Randomized Controlled Trial[ptyp] AND ("2004/01/01"[PDAT] : "2014/12/31"[PDAT]) AND "humans"[MeSH Terms])	11/17/2014	199	1-199	0	199	113	86
Myeloma + monoclonal antibod* (y or ies)	2004-2014	("multiple myeloma"[MeSH Terms] OR ("multiple"[All Fields] AND "myeloma"[All Fields]) OR "multiple myeloma"[All Fields] OR "myeloma"[All Fields]) AND "antibodies, monoclonal"[MeSH Terms] AND (Clinical Trial[ptyp] AND ("2004/01/01"[PDAT] : "2014/12/31"[PDAT]) AND "humans"[MeSH Terms])	11/17/2014	105	200-304	1	104	72	32
Myeloma + checkpoint blockade	2004-2014	("multiple myeloma"[MeSH Terms] OR ("multiple"[All Fields] AND "myeloma"[All Fields]) OR "multiple myeloma"[All Fields] OR "myeloma"[All Fields]) AND (("cell cycle checkpoints"[MeSH Terms] OR ("cell"[All Fields] AND "cycle"[All Fields] AND "checkpoints"[All Fields]) OR "cell cycle checkpoints"[All Fields] OR "checkpoint"[All Fields]) AND blockade[All Fields]) AND (Clinical Trial[ptyp] AND ("2004/01/01"[PDAT] : "2014/12/31"[PDAT]) AND "humans"[MeSH Terms])	11/17/2014	1	305	1	0	0	0

Myeloma + checkpoint blockade or programmed death1 (PD-1) or PD-L1 or B7-H1	**2004-2014**	**((((("multiple myeloma"[MeSH Terms] OR ("multiple"[All Fields] AND "myeloma"[All Fields]) OR "multiple myeloma"[All Fields] OR "myeloma"[All Fields]) AND (("cell cycle checkpoints"[MeSH Terms] OR ("cell"[All Fields] AND "cycle"[All Fields] AND "checkpoints"[All Fields]) OR "cell cycle checkpoints"[All Fields] OR "checkpoint"[All Fields]) AND blockade[All Fields])) OR (programmed[All Fields] AND death-1[All Fields])) OR PD-1[All Fields]) OR ("antigens, cd274"[MeSH Terms] OR ("antigens"[All Fields] AND "cd274"[All Fields]) OR "cd274 antigens"[All Fields] OR ("pd"[All Fields] AND "l1"[All Fields]) OR "pd l1"[All Fields])) OR ("antigens, cd274"[MeSH Terms] OR ("antigens"[All Fields] AND "cd274"[All Fields]) OR "cd274 antigens"[All Fields] OR ("b7"[All Fields] AND "h1"[All Fields]) OR "b7 h1"[All Fields]) AND (Clinical Trial[ptyp] AND ("2004/01/01"[PDAT] : "2014/12/31"[PDAT]) AND "humans"[MeSH Terms])**	11/21/2014	81	306-386	1	80	65	15

Myeloma + oncolytic virus	**2004-2014**	**("multiple myeloma"[MeSH Terms] OR ("multiple"[All Fields] AND "myeloma"[All Fields]) OR "multiple myeloma"[All Fields] OR "myeloma"[All Fields]) AND ("oncolytic viruses"[MeSH Terms] OR ("oncolytic"[All Fields] AND "viruses"[All Fields]) OR "oncolytic viruses"[All Fields] OR ("oncolytic"[All Fields] AND "virus"[All Fields]) OR "oncolytic virus"[All Fields]) AND (Clinical Trial[ptyp] AND ("2004/01/01"[PDAT] : "2014/12/31"[PDAT]) AND "humans"[MeSH Terms])**	11/17/2014	0			0	0	0
Myeloma + oncolytic virus	**2004-2014**	**("multiple myeloma"[MeSH Terms] OR ("multiple"[All Fields] AND "myeloma"[All Fields]) OR "multiple myeloma"[All Fields] OR "myeloma"[All Fields]) AND ("oncolytic viruses"[MeSH Terms] OR ("oncolytic"[All Fields] AND "viruses"[All Fields]) OR "oncolytic viruses"[All Fields] OR ("oncolytic"[All Fields] AND "virus"[All Fields]) OR "oncolytic virus"[All Fields]) AND (("2004/01/01"[PDAT] : "2014/12/31"[PDAT]) AND "humans"[MeSH Terms])**	11/21/2014	44	387-430		44	20	24
Myeloma + virotherapy	**2004-2014**	**("multiple myeloma"[MeSH Terms] OR ("multiple"[All Fields] AND "myeloma"[All Fields]) OR "multiple myeloma"[All Fields] OR "myeloma"[All Fields]) AND "oncolytic virotherapy"[MeSH Major Topic] AND (("2004/01/01"[PDAT] : "2014/12/31"[PDAT]) AND "humans"[MeSH Terms])**	11/21/2014	34	431-464	32	2	1	1

Myeloma + dendritic cell vaccine or idiotype vaccine	**2004-2014**	**(("multiple myeloma"[MeSH Terms] OR ("multiple"[All Fields] AND "myeloma"[All Fields]) OR "multiple myeloma"[All Fields] OR "myeloma"[All Fields]) AND (("dendritic cells"[MeSH Terms] OR ("dendritic"[All Fields] AND "cells"[All Fields]) OR "dendritic cells"[All Fields] OR ("dendritic"[All Fields] AND "cell"[All Fields]) OR "dendritic cell"[All Fields] OR "antigen-presenting cells"[MeSH Terms] OR ("antigen-presenting"[All Fields] AND "cells"[All Fields]) OR "antigen-presenting cells"[All Fields] OR ("dendritic"[All Fields] AND "cell"[All Fields])) AND ("vaccines"[MeSH Terms] OR "vaccines"[All Fields] OR "vaccine"[All Fields]))) OR (("immunoglobulin idiotypes"[MeSH Terms] OR ("immunoglobulin"[All Fields] AND "idiotypes"[All Fields]) OR "immunoglobulin idiotypes"[All Fields] OR "idiotype"[All Fields]) AND ("vaccines"[MeSH Terms] OR "vaccines"[All Fields] OR "vaccine"[All Fields])) AND (Clinical Trial[ptyp] AND ("2004/01/01"[PDAT] : "2014/12/31"[PDAT]) AND "humans"[MeSH Terms])**	11/21/2014	33	465-497	1	32	17	15
				497	Totals	36	461	288	173

NOTE: IN THE BIBLIOGRAPHY, THE NUMBER IN BRACKETS IS THE RECORD NUMBER IN THE ENDNOTE DATABASE (e.g., 156 is the record number for the first item in this bibliography). This is the correct number to use for identifying references in the manuscript during the manuscript draft stages and for any other purposes.

TOPIC: Myeloma + Lenalidomide (OR) Pomalidomide (OR) Thalidomide

1 [156]. Abdelkefi, A., S. Ladeb, et al. (2008). "Single autologous stem-cell transplantation followed by maintenance therapy with thalidomide is superior to double autologous transplantation in multiple myeloma: results of a multicenter randomized clinical trial." Blood **111**(4): 1805-1810.

From April 2003 to December 2006, 195 patients with de novo symptomatic myeloma and younger than 60 years of age were randomly assigned to receive either tandem transplantation up front (arm A, n = 97) or one autologous stem-cell transplantation followed by a maintenance therapy with thalidomide (day + 90, 100 mg per day during 6 months) (arm B, n = 98). Patients included in arm B received a second transplant at disease progression. In both arms, autologous stem-cell transplantation was preceded by first-line therapy with thalidomide-dexamethasone and subsequent collection of peripheral blood stem cells with high-dose cyclophosphamide (4 g/m(2)) and granulocyte colony stimulating factor. Data were analyzed on an intent-to-treat basis. With a median follow-up of
33 months (range, 6-46 months), the 3-year overall survival was 65% in arm A and 85% in arm B (P = .04). The 3-year progression-free survival was 57% in arm A and 85% in arm B (P = .02). Up-front single autologous transplantation followed by 6 months of maintenance therapy with thalidomide (with second transplant in reserve for relapse or progression) is an effective therapeutic strategy to treat multiple myeloma patients and appears superior to tandem transplant in this setting. This study was registered at www.ClinicalTrials.gov as (NCT 00207805).

2 [171]. Attal, M., J. L. Harousseau, et al. (2006). "Maintenance therapy with thalidomide improves survival in patients with multiple myeloma." Blood **108**(10): 3289-3294.

Newer chemotherapeutic protocols as well as high-dose chemotherapy have increased the response rate in myeloma. However, these treatments are not curative. Effective maintenance strategies are now required to prolong the duration of response. We conducted a randomized trial of maintenance treatment with thalidomide and pamidronate. Two months

after high-dose therapy, 597 patients younger than age 65 years were randomly assigned to receive no maintenance (arm A), pamidronate (arm B), or pamidronate plus thalidomide (arm C). A complete or very good
partial response was achieved by 55% of patients in arm A, 57% in arm B, and 67% in arm C (P = .03). The 3-year postrandomization probability of event-free survival was 36% in arm A, 37% in arm B, and 52% in arm C (P < .009). The 4-year postdiagnosis probability of survival was 77% in arm A, 74% in arm B, and 87% in arm C (P < .04). The proportion of patients who had skeletal events was 24% in arm A, 21% in arm B, and 18% in arm C (P = .4). Thalidomide is an effective maintenance therapy in patients with multiple myeloma. Maintenance treatment with pamidronate does not decrease the incidence of bone events.

3 [52]. Attal, M., V. Lauwers-Cances, et al. (2012). "Lenalidomide maintenance after stem-cell transplantation for multiple myeloma." N Engl J Med **366**(19):
1782-1791.

BACKGROUND: High-dose chemotherapy with autologous stem-cell transplantation is a standard treatment for young patients with multiple myeloma. Residual disease is almost always present after transplantation and is responsible for relapse. This phase 3, placebo-controlled trial investigated the efficacy of lenalidomide maintenance therapy after transplantation. METHODS: We randomly assigned 614 patients younger than 65 years of age who had nonprogressive disease after first-line transplantation to maintenance treatment with either lenalidomide (10 mg per day for the first 3 months, increased to 15 mg if tolerated) or placebo until relapse. The primary end point was progression-free survival. RESULTS: Lenalidomide maintenance therapy improved median progression-free survival (41 months, vs. 23 months with placebo; hazard ratio, 0.50; P<0.001). This benefit was observed across all patient subgroups, including those based on the beta(2)-microglobulin level, cytogenetic profile, and response after transplantation. With a median follow-up period of 45 months, more than 70% of patients in both groups were alive at 4 years. The rates of grade 3 or 4 peripheral neuropathy were similar in the two groups. The incidence of second primary cancers was 3.1 per 100 patient-years in the lenalidomide group versus 1.2 per 100 patient-years in the placebo group (P=0.002). Median event-free survival (with events that included second primary cancers) was significantly improved with lenalidomide (40 months, vs. 23 months with placebo; P<0.001). CONCLUSIONS:

Lenalidomide maintenance after transplantation significantly prolonged progression-free and event-free survival among patients with multiple myeloma. Four years after randomization, overall survival was similar in the two study groups. (Funded by the Programme Hospitalier de Recherche Clinique and others; ClinicalTrials.gov number, NCT00430365.).

4 [104]. Barlogie, B., E. Anaissie, et al. (2010). "Reiterative survival analyses of total therapy 2 for multiple myeloma elucidate follow-up time dependency of prognostic variables and treatment arms." J Clin Oncol **28**(18): 3023-3027.

PURPOSE: In Total Therapy 2, after randomly assigning 323 patients with myeloma to thalidomide and 345 to a control arm, no difference was observed in overall survival, with a median follow-up of 42 months, although at 72 months, survival was superior on the thalidomide arm in the one third exhibiting cytogenetic abnormalities (CA). After further follow-up of 87 months, we examined, in reiterative analyses, the effect of increasing time intervals on clinical outcomes relevant to baseline prognostic variables and treatment randomization. PATIENTS AND METHODS: We investigated clinical trial end points as a function of increasing time intervals from protocol enrollment to determine consistencies of results by treatment and prognostic variables. RESULTS: The complete congruence of serial survival plots for both study arms combined attested to stable patient characteristics over the time of accrual and the quality of follow-up management. Presence of CA was associated with consistently inferior survival curves from year 3 onward. Although 80% of patients randomly assigned to thalidomide discontinued study drug after 2 years because of toxicity, its clinical benefit did not reach statistical significance until year 10. The relative ranking order in multivariate models of prognostic factors remained stable over time. Decline in initially high hazard ratio values of gene array-defined high risk is consistent with an initial crisis phase that is time limited. CONCLUSION: Reporting potentially time-sensitive features as a part of clinical trial results will enable the critical reader to judge the robustness of prognostic factors and the time sensitivity of outcome predictors, with important implications for future trial designs.

5 [140]. Barlogie, B., M. Pineda-Roman, et al. (2008). "Thalidomide arm of Total Therapy 2 improves complete remission duration and survival in myeloma patients with metaphase cytogenetic abnormalities." Blood **112**(8): 3115-3121.

Total Therapy 2 examined the clinical benefit of adding thalidomide up-front to a tandem transplant regimen for newly diagnosed patients with multiple myeloma. When initially reported with a median follow-up of 42

months, complete response rate and event-free survival were superior among the 323 patients randomized to thalidomide, whereas overall survival was indistinguishable from that of the 345 patients treated on the control arm. With further follow-up currently at a median of 72 months, survival plots segregated 5 years after initiation of therapy in favor of thalidomide (P = .09), reaching statistical significance for the one third of patients exhibiting cytogenetic abnormalities (CAs; P = .02), a well-recognized adverse prognostic feature. The duration of complete remission was also superior in the cohort presenting with CAs such that, at 7 years from onset of complete remission, 45% remained relapse-free as opposed to 20% on the control arm (P = .05). These observations were confirmed when examined by multivariate analysis demonstrating that thalidomide reduced the hazard of death by 41% among patients with CA-positive disease (P = .008). Because two thirds of patients without CAs have remained alive at 7 years, the presently emerging separation in favor of thalidomide may eventually reach statistical significance as well.

6 [178]. Barlogie, B., G. Tricot, et al. (2006). "Thalidomide and hematopoietic-cell transplantation for multiple myeloma." N Engl J Med **354**(10): 1021-1030.

BACKGROUND: High-dose therapy with melphalan can prolong survival among patients with multiple myeloma. We assessed whether the addition of thalidomide, which has activity against advanced and refractory myeloma, would further improve survival. METHODS: Between October 1998 and February 2004, 668 patients with newly diagnosed multiple myeloma received two cycles of intensive melphalan-based chemotherapy, each supported by autologous hematopoietic stem-cell transplantation. A total of 323 were randomly assigned to receive thalidomide from the outset until disease progression or undue adverse effects, and 345 did not receive thalidomide. The primary end point was the five-year event-free survival rate. Secondary end points were complete response and overall survival. RESULTS: After a median follow-up of 42 months among survivors, the thalidomide and control groups had rates of complete response of 62 percent and 43 percent, respectively (P<0.001), and five-year event-free survival rates of 56 percent and 44 percent (P=0.01). The five-year rate of overall survival was approximately 65 percent in both groups (P=0.90). Median survival after relapse was 1.1 years in the thalidomide group and 2.7 years in the control group (P=0.001). Severe peripheral neuropathy and deep-vein thrombosis occurred more frequently in the thalidomide group than in the control group. CONCLUSIONS: When incorporated into high-dose therapy for

myeloma, thalidomide increased the frequency of complete responses and extended event-free survival at the expense of added adverse effects without improving overall survival. (ClinicalTrials.gov number, NCT00083551.).

7 [93]. Beksac, M., R. Haznedar, et al. (2011). "Addition of thalidomide to oral melphalan/prednisone in patients with multiple myeloma not eligible for transplantation: results of a randomized trial from the Turkish Myeloma Study Group." Eur J Haematol **86**(1): 16-22.

The combination of melphalan-prednisone-thalidomide (MPT) has been investigated in several clinical studies that differed significantly with regard to patient characteristics and treatment schedules. This prospective trial differs from previous melphalan-prednisone (MP) vs. MPT trials by treatment dosing, duration, routine anticoagulation, and permission for a crossover. Newly diagnosed patients with multiple myeloma (MM) (n=122) aged greater than 55 yr, not eligible for transplantation were randomized to receive 8 cycles of M (9 mg/m(2) /d) and P (60 mg/m(2) /d) for 4d every 6 wk (n=62) or MP and thalidomide (100 mg/d) continuously (n=60). Primary endpoint was treatment response and toxicities following 4 and 8 cycles of therapy. Secondary endpoints were disease-free (DFS) and overall survival (OS). Overall, MPT-treated patients were younger (median 69 yr vs. 72 yr; P=0.016) and had a higher incidence of renal impairment (RI, 19% vs. 7%, respectively; P=0.057). After 4 cycles of treatment (n=115), there were more partial responses or better in the MPT arm than in the MP arm (57.9% vs. 37.5%; P=0.030). However, DFS and OS were not significantly different between the arms after a median of 23 months follow-up (median OS 26.0 vs. 28.0 months, P=0.655; DFS 21.0 vs. 14.0 months, P=0.342, respectively). Crossover to MPT was required in 11 patients, 57% of whom responded to treatment. A higher rate of grade 3-4 infections was observed in the MPT arm compared with the MP arm (22.4% vs. 7.0%; P=0.033). However, none of these infections were associated with febrile neutropenia. Death within the first 3 months was observed more frequently in the MP arm (n=8, 14.0%) than in the MPT arm (n=2, 3.4%; P=0.053). Long-term discontinuation and dose reduction rates were also analyzed (MPT: 15.5% vs. MP: 5.3%; P=0.072). Although patients treated with MPT were relatively younger and had more frequent RI, better responses and less early mortality were observed in all age groups despite more frequent discontinuation.

8 [1]. Benboubker, L., M. A. Dimopoulos, et al. (2014). "Lenalidomide and dexamethasone in transplant-ineligible patients with myeloma." N Engl J Med **371**(10): 906-917.

BACKGROUND: The combination melphalan-prednisone-thalidomide (MPT) is considered a standard therapy for patients with myeloma who are ineligible for stem-cell transplantation. However, emerging data on the use of lenalidomide and low-dose dexamethasone warrant a prospective comparison of the two approaches. METHODS: We randomly assigned 1623 patients to lenalidomide and dexamethasone in 28-day cycles until disease progression (535 patients), to the same combination for 72 weeks (18 cycles; 541 patients), or to MPT for 72 weeks (547 patients). The primary end point was progression-free survival with continuous lenalidomide-dexamethasone versus MPT. RESULTS: The median progression-free survival was 25.5 months with continuous lenalidomide-dexamethasone, 20.7 months with 18 cycles of lenalidomide-dexamethasone, and 21.2 months with MPT (hazard ratio for the risk of progression or death, 0.72 for continuous lenalidomide-dexamethasone vs. MPT and 0.70 for continuous lenalidomide-dexamethasone vs. 18 cycles of lenalidomide-dexamethasone; P<0.001 for both comparisons). Continuous lenalidomide-dexamethasone was superior to MPT for all secondary efficacy end points, including overall survival (at the interim analysis). Overall survival at 4 years was 59% with continuous lenalidomide-dexamethasone, 56% with 18 cycles of lenalidomide-dexamethasone, and 51% with MPT. Grade 3 or 4 adverse events were somewhat less frequent with continuous lenalidomide-dexamethasone than with MPT (70% vs. 78%). As compared with MPT, continuous lenalidomide-dexamethasone was associated with fewer hematologic and neurologic toxic events, a moderate increase in infections, and fewer second primary hematologic cancers. CONCLUSIONS: As compared with MPT, continuous lenalidomide-dexamethasone given until disease progression was associated with a significant improvement in progression-free survival, with an overall survival benefit at the interim analysis, among patients with newly diagnosed multiple myeloma who were ineligible for stem-cell transplantation. (Funded by Intergroupe, Francophone du Myelome and Celgene; FIRST ClinicalTrials.gov number, NCT00689936; European Union Drug Regulating Authorities Clinical Trials number, 2007-004823-39.).

9 [69]. Boyd, K. D., F. M. Ross, et al. (2011). "The clinical impact and molecular biology of del(17p) in multiple myeloma treated with conventional or thalidomide-based therapy." Genes Chromosomes Cancer **50**(10): 765-774.

Hemizygous deletion of 17p (del(17p)) has been identified as a variable associated with poor prognosis in myeloma, although its impact in the context of thalidomide therapy is not well described. The clinical outcome of 85 myeloma patients with del(17p) treated in a clinical trial incorporating both conventional and thalidomide-based induction therapies was examined. The clinical impact of deletion, low expression, and mutation of TP53 was also determined. Patients with del(17p) did not have inferior response rates compared to patients without del(17p), but, despite this, del(17p) was associated with impaired overall survival (OS) (median OS 26.6 vs. 48.5 months, $P < 0.001$). Within the del(17p) group, thalidomide induction therapy was associated with improved response rates compared to conventional therapy, but there was no impact on OS. Thalidomide maintenance was associated with impaired OS, although our analysis suggests that this effect may have been due to confounding variables. A minimally deleted region on 17p13.1 involving 17 genes was identified, of which only TP53 and SAT2 were underexpressed. TP53 was mutated in <1% in patients without del(17p) and in 27% of patients with del(17p). The higher TP53 mutation rate in samples with del(17p) suggests a role for TP53 in these clinical outcomes. In conclusion, del(17p) defined a patient group associated with short survival in myeloma, and although thalidomide induction therapy was associated with improved response rates, it did not impact OS, suggesting that alternative therapeutic strategies are required for this group.

10 [97]. Bringhen, S., A. Larocca, et al. (2010). "Efficacy and safety of once-weekly bortezomib in multiple myeloma patients." Blood **116**(23): 4745-4753.

In a recent phase 3 trial, bortezomib-melphalan-prednisone-thalidomide followed by maintenance treatment with bortezomib-thalidomide demonstrated superior efficacy compared with bortezomib-melphalan-prednisone. To decrease neurologic toxicities, the protocol was amended and patients in both arms received once-weekly instead of the initial twice-weekly bortezomib infusions: 372 patients received once-weekly and 139 twice-weekly bortezomib. In this post-hoc analysis we assessed the impact of the schedule change on clinical outcomes and safety. Long-term outcomes appeared similar: 3-year progression-free survival rate was 50% in the once-weekly and 47% in the twice-weekly group ($P > .999$), and 3-year overall survival rate was 88%

and 89%, respectively (P = .54). The complete response rate was 30% in the once-weekly and 35% in the twice-weekly group (P = .27). Nonhematologic grade 3/4 adverse events were reported in 35% of once-weekly patients and 51% of twice-weekly patients (P = .003). The incidence of grade 3/4 peripheral neuropathy was 8% in the once-weekly and 28% in the twice-weekly group (P < .001); 5% of patients in the once-weekly and 15% in the twice-weekly group discontinued therapy because of peripheral neuropathy (P < .001). This improvement in safety did not appear to affect efficacy. This study is registered at http://www.clinicaltrials.gov as NCT01063179.

11 [115]. Brown, R. D., A. Spencer, et al. (2009). "Prognostically significant cytotoxic T cell clones are stimulated after thalidomide therapy in patients with multiple myeloma." Leuk Lymphoma **50**(11): 1860-1864.

The expanded T cell clones are associated with a prolonged survival in patients with multiple myeloma. We sought to confirm this prognostic significance in a multicenter patient cohort and investigate the effect of thalidomide on clones and T regulatory cells (T(regs)). Blood was collected from 120 patients enrolled in a Phase III trial of maintenance therapy +/- thalidomide after autologous stem cell transplantation. TCR Vbeta repertoire analysis identified T cell expansions in 48% of patients pre-transplant and 68% after 8-month maintenance. T cell expansions, previously shown to be clonal, were predominantly CD8+ (93%) and all 24 TCR Vbeta families tested were represented. Thalidomide therapy was associated with a significant increase in the incidence of patients with multiple expansions (49% vs. 23%; chi2 = 6.8; p = 0.01). The presence of expansions regardless of therapy was associated with a significantly longer median progression free survival (PFS) (32.1 vs. 17.6 months; chi2 = 5.6; p = 0.02) and overall survival (OS) (chi2 = 3.9; p < 0.05). Median PFS in the thalidomide arm was 50.9 months for patients with expansions and 28.3 months for patients without expansions (chi2 = 19.4; p = 0.0002). Thalidomide did not appear to modulate T(reg) numbers. Expanded T cell clones are prognostically significant and have an impact on progression after thalidomide therapy in a proportion of patients.

12 [29]. Broyl, A., R. Kuiper, et al. (2013). "High cereblon expression is associated with better survival in patients with newly diagnosed multiple myeloma treated with thalidomide maintenance." Blood **121**(4): 624-627.

Recently, cereblon (CRBN) expression was found to be essential for the activity of thalidomide and lenalidomide. In the present study, we

investigated whether the clinical efficacy of thalidomide in multiple myeloma is associated with CRBN expression in myeloma cells. Patients with newly diagnosed multiple myeloma were included in the HOVON-65/GMMG-HD4 trial, in which postintensification treatment in 1 arm consisted of daily thalidomide (50 mg) for 2 years. Gene-expression profiling, determined at the start of the trial, was available for 96 patients who started thalidomide maintenance. In this patient set, increase of CRBN gene expression was significantly associated with longerprogression-free survival (P = .005). In contrast, no association between CRBN expression and survival was observed in the arm with bortezomib maintenance. We conclude that CRBN expression may be associated with the clinical efficacy of thalidomide. This trial has been registered at the Nederlands Trial Register (www.trialregister.nl) as NTR213; at the European Union Drug Regulating Authorities Clinical Trials (EudraCT) as 2004-000944-26; and at the International Standard Randomized Controlled Trial Number (ISRCTN) as 64455289.

13 [22]. Buikhuisen, W. A., J. A. Burgers, et al. (2013). "Thalidomide versus active supportive care for maintenance in patients with malignant mesothelioma after first-line chemotherapy (NVALT 5): an open-label, multicentre, randomised phase 3 study." Lancet Oncol **14**(6): 543-551.

BACKGROUND: Standard chemotherapy does not lead to long-term survival in patients with malignant pleural mesothelioma. Malignant pleural mesothelioma is strongly dependent on vasculature with high vessel counts and high concentrations of serum vascular growth factors. Thalidomide has shown antiangiogenic activity, and we hypothesised that its use in the maintenance setting could improve outcomes. METHODS: In this open-label, multicentre, randomised phase 3 study, eligible patients had proven malignant pleural or peritoneal mesothelioma and had received a minimum of four cycles of first-line treatment containing at least pemetrexed, with or without cisplatin or carboplatin, and had not progressed on this treatment. Patients were randomly assigned (in a 1:1 ratio, stratified by previous first-line chemotherapy, histological subtype, and recruiting hospital) to receive thalidomide 200 mg per day (including a 2 week run in of 100 mg per day) plus active supportive care or active supportive care alone until disease progression. Patients were required to be registered and to start treatment with thalidomide within 10 weeks after the end of the first-line chemotherapy. Thalidomide was given for a maximum of 1 year or until unacceptable toxicity. The primary endpoint was time to progression. The primary analyses were by intention to treat. The study is registered, ISRCTN13632914. FINDINGS: Between May 11, 2004,

and Dec 23, 2009, we randomly assigned 222 patients, 111 in each group (one patient on active supportive care later withdrew consent and was excluded from analyses). At the time of this final analysis, median follow-up was 33.1 months (IQR 22.3-66.8), and physician-reported disease progression had occurred in 104 patients in the thalidomide group and 107 in the active supportive care group; 92 patients in the thalidomide group and 93 in the active supportive care group had died. Median time to progression in the thalidomide group was 3.6 months (95% CI 3.2-4.1) compared with 3.5 months (2.3-4.8) in the active supportive care group (hazard ratio 0.95, 95% CI 0.73-1.20, p=0.72). 43 (39%) grade 3 or 4 adverse events were reported in the thalidomide group and 31 (28%) in the active supportive care group; neurosensory events were reported by two (2%) patients on thalidomide and none on active supportive care, cardiac events by two (2%) patients on thalidomide and three (3%) on active supportive care, and thromboembolic events by three (3%) patients on thalidomide and none on active supportive care. INTERPRETATION: No benefit was noted in time to progression with the addition of thalidomide maintenance to first-line chemotherapy. Different treatment strategies are needed to improve outcomes in patients with malignant mesothelioma. FUNDING: Dutch Cancer Society (KWF), Eli Lilly, NSW Dust Disease Compensation Board, University of Sydney, and Cancer Australia.

14 [9]. Burnette, B. L., A. Dispenzieri, et al. (2013). "Treatment trade-offs in myeloma: A survey of consecutive patients about contemporary maintenance strategies." Cancer **119**(24): 4308-4315.

BACKGROUND: Two randomized trials have demonstrated improved progression-free survival (PFS) with lenalidomide maintenance after autologous transplantation for multiple myeloma (MM). Overall survival (OS) results are conflicting, and quality-of-life (QOL) data are lacking. The authors conducted a systematic survey of patients with MM regarding what constitutes a meaningful benefit that would make burdens of maintenance treatments (toxicity and cost) acceptable. METHODS: A self-administered survey was mailed to 1159 consecutive, living patients who were evaluated at Mayo Clinic. The survey provided background information on the standard of care for MM and data on maintenance. Patients were asked to estimate the magnitude of OS benefit that would be acceptable for various degrees of toxicity and cost. RESULTS: Of 1159 surveys sent, 886 patients (83.2%) responded, and 736 patients returned a completed survey (66% raw response rate). The most worrisome potential toxicity was identified as peripheral neuropathy by 27% of patients, cytopenias by 24%, deep vein thrombosis by 20%, fatigue by 15%, nausea

by 8%, and diarrhea/constipation by 7%. If treatment was free, had no toxicity, and the OS benefit was </=1 year, then 49% of patients indicated that they would choose maintenance; with moderate toxicity, this proportion decreased to 42%. Adding a treatment cost of $25 per month decreased the proportion that would choose maintenance to 39% of patients. CONCLUSIONS: The current results indicated that willingness to receive maintenance treatment declined when actual benefits were provided in concrete numeric terms compared with a general statement of PFS benefit. The authors also observed that the magnitude of benefit required to consider maintenance was affected by cost and toxicity.

15 [55]. Cavo, M., L. Pantani, et al. (2012). "Bortezomib-thalidomide-dexamethasone is superior to thalidomide-dexamethasone as consolidation therapy after autologous hematopoietic stem cell transplantation in patients with newly diagnosed multiple myeloma." Blood **120**(1): 9-19.

In a randomized, phase 3 study, superior complete/near-complete response (CR/nCR) rates and extended progression-free survival were demonstrated with bortezomib-thalidomide-dexamethasone (VTD) versus thalidomide-dexamethasone (TD) as induction therapy before, and consolidation after, double autologous stem cell transplantation for newly diagnosed myeloma patients (intention-to-treat analysis; VTD, n = 236; TD, n = 238). This per-protocol analysis (VTD, n = 160; TD, n = 161) specifically assessed the efficacy and safety of consolidation with VTD or TD. Before starting consolidation, CR/nCR rates were not significantly different in the VTD (63.1%) and TD arms (54.7%). After consolidation, CR (60.6% vs 46.6%) and CR/nCR (73.1% vs 60.9%) rates were significantly higher for VTD-treated versus TD-treated patients. VTD consolidation significantly increased CR and CR/nCR rates, but TD did not (McNemar test). With a median follow-up of 30.4 months from start of consolidation, 3-year progression-free survival was significantly longer for the VTD group (60% vs 48% for TD). Grade 2 or 3 peripheral neuropathy (8.1% vs 2.4%) was more frequent with VTD (grade 3, 0.6%) versus TD consolidation. The superior efficacy of VTD versus TD as induction was retained despite readministration as consolidation therapy after double autologous transplantation. VTD consolidation therapy significantly contributed to improved clinical outcomes observed for patients randomly assigned to the VTD arm of the study. The study is registered at www.clinicaltrials.gov as #NCT01134484.

16 [89]. Cavo, M., P. Tacchetti, et al. (2010). "Bortezomib with thalidomide plus dexamethasone compared with thalidomide plus dexamethasone as induction therapy before, and consolidation therapy after, double autologous stem-cell transplantation in newly diagnosed multiple myeloma: a randomised phase 3 study." Lancet **376**(9758): 2075-2085.

BACKGROUND: Thalidomide plus dexamethasone (TD) is a standard induction therapy for myeloma. We aimed to assess the efficacy and safety of addition of bortezomib to TD (VTD) versus TD alone as induction therapy before, and consolidation therapy after, double autologous stem-cell transplantation in newly diagnosed multiple myeloma. METHODS: Patients (aged 18-65 years) with previously untreated symptomatic myeloma were enrolled from 73 sites in Italy between May, 2006, and April, 2008, and data collection continued until June 30, 2010. Patients were randomly allocated (1:1 ratio) by a web-based system to receive three 21-day cycles of thalidomide (100 mg daily for the first 14 days and 200 mg daily thereafter) plus dexamethasone (40 mg daily on 8 of the first 12 days, but not consecutively; total of 320 mg per cycle), either alone or with bortezomib (1.3 mg/m(2) on days 1, 4, 8, and 11). The randomisation sequence was computer generated by the study coordinating team and was stratified by disease stage. After double autologous stem-cell transplantation, patients received two 35-day cycles of their assigned drug regimen, VTD or TD, as consolidation therapy. The primary endpoint was the rate of complete or near complete response to induction therapy. Analysis was by intention to treat. Patients and treating physicians were not masked to treatment allocation. This study is still underway but is not recruiting participants, and is registered with ClinicalTrials.gov, number NCT01134484, and with EudraCT, number 2005-003723-39. FINDINGS: 480 patients were enrolled and randomly assigned to receive VTD (n=241 patients) or TD (n=239). Six patients withdrew consent before start of treatment, and 236 on VTD and 238 on TD were included in the intention-to-treat analysis. After induction therapy, complete or near complete response was achieved in 73 patients (31%, 95% CI 25.0-36.8) receiving VTD, and 27 (11%, 7.3-15.4) on TD ($p<0.0001$). Grade 3 or 4 adverse events were recorded in a significantly higher number of patients on VTD (n=132, 56%) than in those on TD (n=79, 33%; $p<0.0001$), with a higher occurrence of peripheral neuropathy in patients on VTD (n=23, 10%) than in those on TD (n=5, 2%; $p=0.0004$). Resolution or improvement of severe peripheral neuropathy was recorded in 18 of 23 patients on VTD, and in three of five patients on TD. INTERPRETATION: VTD induction therapy before double autologous stem-cell transplantation significantly improves rate of complete or near complete response, and represents a new standard of

care for patients with multiple myeloma who are eligible for transplant. FUNDING: Seragnoli Institute of Haematology at the University of Bologna, Bologna, Italy.

17 [38]. Chen, N., C. Kasserra, et al. (2012). "Single-dose pharmacokinetics of lenalidomide in healthy volunteers: dose proportionality, food effect, and racial sensitivity." Cancer Chemother Pharmacol **70**(5): 717-725.

PURPOSE: Lenalidomide is an immunomodulatory drug with efficacy in various hematological malignancies. The purpose of these studies was to evaluate the single-dose pharmacokinetics of lenalidomide, including dose proportionality, food effect, and racial sensitivity. METHODS: Three studies were conducted including a total of 58 healthy subjects: a randomized, single-blind, alternating group, single-ascending dose study; a randomized, two-way crossover food effect study; and a randomized, double-blind, two-group, within-subject, single-ascending dose study. RESULTS: Oral absorption of lenalidomide was rapid and the maximum plasma concentration (C (max)) was observed approximately 1 h post-dose. Co-administration with a high-fat meal reduced the area under the concentration-time curve (AUC) and C (max) by approximately 20 and 50 %, respectively, and delayed time to C (max) (t (max)) by 1.63 h. However, phase III trials were dosed without regard to food; therefore, clinical relevance of the food effect was minimal. The terminal elimination half-life (t ((1/2))) was 3-4 h at doses up to 50 mg and was not affected by food. The AUC and C (max) were proportional to lenalidomide single doses (5-400 mg), and total and renal clearance were dose-independent. The R- to S-lenalidomide ratio in plasma was stable over time, approximately 45-55 % of total drug. There were no differences in pharmacokinetic parameters, dose-exposure relationship, or enantiomeric ratio, between Japanese and Caucasian subjects. CONCLUSION: Lenalidomide displayed linear pharmacokinetics from doses 5-400 mg in healthy subjects. Although food reduced bioavailability, this was not considered clinically relevant. Lenalidomide was generally well tolerated in both ethnic groups.

18 [39]. Coleman, E. A., J. A. Goodwin, et al. (2012). "Effects of exercise on fatigue, sleep, and performance: a randomized trial." Oncol Nurs Forum **39**(5): 468-477.

PURPOSE/OBJECTIVES: To compare usual care with a home-based individualized exercise program (HBIEP) in patients receiving intensive treatment for multiple myeloma (MM)and epoetin alfa therapy. DESIGN: Randomized trial with repeated measures of two groups (one

experimental and one control) and an approximate 15-week experimental period. SETTING: Outpatient setting of the Myeloma Institute for Research and Therapy at the Rockfellow Cancer Center at the University of Arkansas for Medical Sciences. SAMPLE: 187 patients with newly diagnosed MM enrolled in a separate study evaluating effectiveness of the Total Therapy regimen, with or without thalidomide. METHODS: Measurements included the Profile of Mood States fatigue scale, Functional Assessment of Cancer Therapy-Fatigue, ActiGraph(R) recordings, 6-Minute Walk Test, and hemoglobin levels at baseline and before and after stem cell collection. Descriptive statistics were used to compare demographics and treatment effects, and repeated measures analysis of variance was used to determine effects of HBIEP. MAIN RESEARCH VARIABLES: Fatigue, nighttime sleep, performance (aerobic capacity) as dependent or outcome measures, and HBIEP combining strength building and aerobic exercise as the independent variable. FINDINGS: Both groups were equivalent for age, gender, race, receipt of thalidomide, hemoglobin levels, and type of treatment regimen for MM. No statistically significant differences existed among the experimental and control groups for fatigue, sleep, or performance (aerobic capacity). Statistically significant differences ($p < 0.05$) were found in each of the study outcomes for all patients as treatment progressed and patients experienced more fatigue and poorer nighttime sleep and performance (aerobic capacity). CONCLUSIONS: The effect of exercise seemed to be minimal on decreasing fatigue, improving sleep, and improving performance (aerobic capacity). IMPLICATIONS FOR NURSING: Exercise is safe and has physiologic benefits for patients undergoing MM treatment; exercise combined with epoetin alfa helped alleviate anemia.

19 [101]. Dimopoulos, M., A. Alegre, et al. (2010). "The efficacy and safety of lenalidomide plus dexamethasone in relapsed and/or refractory multiple myeloma patients with impaired renal function." Cancer **116**(16): 3807-3814.

BACKGROUND: In patients with multiple myeloma, renal impairment (RI) at the time of diagnosis is associated with poor survival. To the authors' knowledge, the current retrospective analysis presented is the first to assess the impact of various degrees of renal dysfunction on safety and efficacy outcomes in a large cohort of patients with relapsed and/or refractory multiple myeloma who received treatment with lenalidomide plus dexamethasone. METHODS: Three hundred fifty-three patients from 2 large phase 3 trials were randomized to receive lenalidomide (25 mg) plus dexamethasone (40 mg). For the purpose of this analysis, RI was defined according to the calculated creatinine clearance (CLCr) level as follows:

mild or no RI (CLCr>or=60 mL/minute), moderate RI (CLCr from >or=30 mL/minute to <60 mL/minute), and severe RI (CLCr<30 mL/minute). RESULTS: The RI subgroups did not differ significantly in terms of the overall response rate (range, 50%-64%) or response quality (very good partial response or better, 27%-37%). In all RI subgroups, the time to progression and progression-free survival did not differ significantly compared with the mild or no RI group. Patients with RI experienced an increased incidence of thrombocytopenia, required more frequent lenalidomide dose reduction or interruption, and had shorter overall survival than patients with mild or no RI (P=.006). Lenalidomide plus dexamethasone led to improvement in renal function in the majority of patients. CONCLUSIONS: The results from this study indicated that, with careful monitoring of the CLCr level and adverse events as well as appropriate dose adjustments, lenalidomide plus dexamethasone is an effective and well tolerated treatment option for patients with multiple myeloma who have RI.

20 [152]. Dimopoulos, M., A. Spencer, et al. (2007). "Lenalidomide plus dexamethasone for relapsed or refractory multiple myeloma." N Engl J Med **357**(21): 2123-2132.

BACKGROUND: Lenalidomide is a structural analogue of thalidomide with similar but more potent biologic activity. This phase 3, placebo-controlled trial investigated the efficacy of lenalidomide plus dexamethasone in the treatment of relapsed or refractory multiple myeloma. METHODS: Of 351 patients who had received at least one previous antimyeloma therapy, 176 were randomly assigned to receive 25 mg of oral lenalidomide and 175 to receive placebo on days 1 to 21 of a 28-day cycle. In addition, all patients received 40 mg of oral dexamethasone on days 1 to 4, 9 to 12, and 17 to 20 for the first four cycles and subsequently, after the fourth cycle, only on days 1 to 4. Patients continued in the study until the occurrence of disease progression or unacceptable toxic effects. The primary end point was time to progression. RESULTS: The time to progression was significantly longer in the patients who received lenalidomide plus dexamethasone (lenalidomide group) than in those who received placebo plus dexamethasone (placebo group) (median, 11.3 months vs. 4.7 months; P<0.001). A complete or partial response occurred in 106 patients in the lenalidomide group (60.2%) and in 42 patients in the placebo group (24.0%, P<0.001), with a complete response in 15.9% and 3.4% of patients, respectively (P<0.001). Overall survival was significantly improved in the lenalidomide group (hazard ratio for death, 0.66; P=0.03). Grade 3 or 4 adverse events that occurred in more than 10% of patients in the lenalidomide group were neutropenia (29.5%, vs. 2.3% in the placebo group), thrombocytopenia (11.4% vs. 5.7%), and venous

thromboembolism (11.4% vs. 4.6%). CONCLUSIONS: Lenalidomide plus dexamethasone is more effective than high-dose dexamethasone alone in relapsed or refractory multiple myeloma. (ClinicalTrials.gov number, NCT00424047 [ClinicalTrials.gov].).

21 [17]. Dimopoulos, M. A., M. Beksac, et al. (2013). "Phase II study of bortezomib-dexamethasone alone or with added cyclophosphamide or lenalidomide for sub-optimal response as second-line treatment for patients with multiple myeloma." Haematologica **98**(8): 1264-1272.

This phase II study is the first prospective evaluation of bortezomib-dexamethasone as second-line therapy for relapsed/refractory multiple myeloma. A total of 163 patients were enrolled to receive four cycles of bortezomib-dexamethasone. Patients were investigator-assessed for response at cycle 5 Day 1, then treated as follows: responding patients received another four cycles of bortezomib-dexamethasone, while patients with stable disease were subsequently randomized to sequential treatment with a further four cycles of bortezomib-dexamethasone alone or with added cyclophosphamide or lenalidomide. The primary end point was response to sequential therapy; however, this could not be evaluated because

investigator-assessed response rates to bortezomib-dexamethasone after four cycles were high, and an insufficient number of patients were randomized to sequential treatment per protocol. Among all 163 patients, validated best confirmed response rate was 66%, including 37% complete/very good partial responses; median response duration was 9.7 months. After a median follow up of 16.9 months, median time to progression and progression-free survival were 9.5 and 8.6 months, respectively; estimated 1-year overall survival was 81%. Median glomerular filtration rate improved from baseline during treatment. Among 58 patients with baseline glomerular filtration rate below 50 mL/min, 24 had renal responses. Grade 3/4 adverse events included: thrombocytopenia (17%), anemia (10%), constipation (6%), peripheral sensory neuropathy (5%), and polyneuropathy (5%). Overall, 57% of neuropathy events improved/resolved; median time to improvement was 2.1 months. These findings suggest bortezomib-dexamethasone represents an active, feasible second-line treatment option for patients with relapsed/refractory myeloma.

22 [28]. Dimopoulos, M. A., M. Delforge, et al. (2013). "Lenalidomide, melphalan, and prednisone, followed by lenalidomide maintenance, improves health-related quality of life in newly diagnosed multiple myeloma patients

aged 65 years or older: results of a randomized phase III trial." Haematologica **98**(5):
784-788.

The MM-015 trial assessed the effect of lenalidomide-based therapy on health-related quality of life. Patients (n=459) with newly diagnosed multiple myeloma aged 65 years or over were randomized 1:1:1 to nine 4-week cycles of lenalidomide, melphalan, and prednisone, followed by lenalidomide maintenance; or lenalidomide, melphalan, and prednisone, or melphalan and prednisone, with no maintenance therapy. Patients completed health-related quality of life questionnaires at baseline, after every third treatment cycle, and at treatment end. Health-related quality of life improved in all treatment groups during induction therapy. Patients receiving lenalidomide maintenance had the most pronounced improvements, Global Health Status/Quality of Life (P<0.05), Physical Functioning (P<0.01), and Side Effects of Treatment (P<0.05) out of 6 pre-selected health-related quality of life domains. More patients receiving lenalidomide maintenance achieved minimal important differences (P<0.05 for Physical Functioning). Therefore, lenalidomide, melphalan, and prednisone, followed by lenalidomide maintenance, improves health-related quality of life in patients with newly diagnosed multiple myeloma. (Clinicaltrials.gov identifier NCT00405756).

23 [153]. Facon, T., J. Y. Mary, et al. (2007). "Melphalan and prednisone plus thalidomide versus melphalan and prednisone alone or reduced-intensity autologous stem cell transplantation in elderly patients with multiple myeloma (IFM 99-06): a randomised trial." Lancet **370**(9594): 1209-1218.

BACKGROUND: In multiple myeloma, combination chemotherapy with melphalan plus prednisone is still regarded as the standard of care in elderly patients. We assessed whether the addition of thalidomide to this combination, or reduced-intensity stem cell transplantation, would improve survival. METHODS: Between May 22, 2000, and Aug 8, 2005, 447 previously untreated patients with multiple myeloma, who were aged between 65 and 75 years, were randomly assigned to receive either melphalan and prednisone (MP; n=196), melphalan and prednisone plus thalidomide (MPT; n=125), or reduced-intensity stem cell transplantation using melphalan 100 mg/m2 (MEL100; n=126). The primary endpoint was overall survival. Analysis was by intention to treat. This trial is registered with ClinicalTrials.gov, number NCT00367185. FINDINGS: After a median follow-up of 51.5 months (IQR 34.4-63.2), median overall survival times were 33.2 months (13.8-54.8) for MP, 51.6 months (26.6-not reached) for MPT, and 38.3 months (13.0-61.6) for MEL100. The MPT regimen was associated with

a significantly better overall survival than was the MP regimen (hazard ratio 0.59, 95% CI 0.46-0.81, p=0.0006) or MEL100 regimen (0.69, 0.49-0.96, p=0.027). No difference was seen for MEL100 versus MP (0.86, 0.65-1.15, p=0.32). INTERPRETATION: The results of our trial provide strong evidence to indicate that the use of thalidomide in combination with melphalan and prednisone should, at present, be the reference treatment for previously untreated elderly patients with multiple myeloma.

24 [135]. Fonseca, R. and S. V. Rajkumar (2008). "Consolidation therapy with bortezomib/lenalidomide/ dexamethasone versus bortezomib/dexamethasone after a dexamethasone-based induction regimen in patients with multiple myeloma: a randomized phase III trial." Clin Lymphoma Myeloma **8**(5): 315-317.

In recent years, we have seen tremendous progress in our ability to achieve durable responses in patients with multiple myeloma. At the center of this progress, we have the development of 2 unrelated classes of drugs: proteasome inhibitors such as bortezomib and immunomodulatory drugs such as thalidomide and lenalidomide. The depth and durability of responses attained with these agents in the first-line setting has raised the possibility that they may be considered primary therapy. The Eastern Cooperative Oncology Group E1A05 clinical trial addresses the potential role of bortezomib and dexamethasone (VD) or VD plus lenalidomide (VRD) as primary first-line therapy. This clinical trial enrolls patients who have completed a dexamethasone-based induction (excluding patients using bortezomib). Assuming that most patients entering this clinical trial have been previously treated with thalidomide or lenalidomide, the trial will test whether switching to a proteasome inhibitor (VD arm) versus adding a proteasome inhibitor (VRD) results in superior longterm disease control. Patients entering this clinical trial will have deferred stem cell transplantation until the time of relapse. The primary endpoint of the trial is progression-free survival. By using an alkylator-free consolidation and reserving stem cell transplantation until the time of relapse, we hope that these treatment strategies will further prolong the survival of patients with myeloma.

25 [51]. Garderet, L., S. Iacobelli, et al. (2012). "Superiority of the triple combination of bortezomib-thalidomide-dexamethasone over the dual combination of thalidomide-dexamethasone in patients with multiple myeloma progressing or relapsing after autologous transplantation: the MMVAR/IFM 2005-04 Randomized Phase III Trial from the Chronic Leukemia Working Party of the European Group for Blood and Marrow Transplantation." J Clin Oncol **30**(20): 2475-2482.

PURPOSE: This prospective multicenter phase III study compared the efficacy and safety of a triple combination (bortezomib-thalidomide-dexamethasone [VTD]) versus a dual combination (thalidomide-dexamethasone [TD]) in patients with multiple myeloma (MM) progressing or relapsing after autologous stem-cell transplantation (ASCT). PATIENTS AND METHODS: Overall, 269 patients were randomly assigned to receive bortezomib (1.3 mg/m(2) intravenous bolus) or no bortezomib for 1 year, in combination with thalidomide (200 mg per day orally) and dexamethasone (40 mg orally once a day on 4 days once every 3 weeks). Bortezomib was administered on days 1, 4, 8, and 11 with a 10-day rest period (day 12 to day 21) for eight cycles (6 months), and then on days 1, 8, 15, and 22 with a 20-day rest period (day 23 to day 42) for four cycles (6 months). RESULTS: Median time to progression (primary end point) was significantly longer with VTD than TD (19.5 v13.8 months; hazard ratio,
0.59; 95% CI, 0.44 to 0.80; P = .001), the complete response plus near-complete response rate was higher (45% v 21%; P 0.001), and the median duration of response was longer (17.9 v 13.4 months; P.04) [corrected].The 24-month survival rate was in favor of VTD (71% v 65%; P =
.093). Grade 3 peripheral neuropathy was more frequent with VTD (29% v 12%; P = .001) as were the rates of grades 3 and 4 infection and thrombocytopenia. CONCLUSION: VTD was more effective than TD in the treatment of patients with MM with progressive or relapsing disease post-ASCT but was associated with a higher incidence of grade 3 neurotoxicity.

26 [150]. Haessler, J., J. D. Shaughnessy, Jr., et al. (2007). "Benefit of complete response in multiple myeloma limited to high-risk subgroup identified by gene expression profiling." Clin Cancer Res **13**(23): 7073-7079.

EXPERIMENTAL DESIGN: To determine whether the clinical benefit of complete remission (CR) may depend on prognostic subgroups of patients with multiple myeloma. PATIENTS AND METHODS: Newly diagnosed patients with myeloma received a tandem autotransplant regimen. Using multivariate regression analyses, we examined the prognostic implications of time-dependent onset of CR on overall survival and event-free survival in the context of standard prognostic factors (SPF) and gene expression profiling-derived data available for 326 patients. RESULTS: CR benefited patients regardless of risk status when only SPFs were examined. With knowledge of gene array data, a survival (and event-free survival) benefit of CR only pertained to the small high-risk subgroup of 13% of patients (hazard ratio, 0.23; P = 0.001), whereas the

majority of patients with low-risk disease had similar survival expectations whether or not CR was achieved (hazard ratio, 0.68; P = 0.128). CONCLUSIONS: Access to gene expression information permitted the recognition of a small very high-risk subgroup of 13% of patients, in whom prolonged survival critically depended on achieving CR. Absence of such benefit in the remainder should lead to a reassessment of clinical trial designs that rely on this end point as a surrogate for long-term prognosis.

27 [44]. Heuck, C. J., J. Szymonifka, et al. (2012). "Thalidomide in total therapy 2 overcomes inferior prognosis of myeloma with low expression of the glucocorticoid receptor gene NR3C1." Clin Cancer Res **18**(19): 5499-5506.

PURPOSE: Because dexamethasone remains a key component of myeloma therapy, we wished to examine the impact of baseline and relapse expression levels of the glucocorticoid receptor gene NR3C1 on survival outcomes in the context of treatment with or without thalidomide. EXPERIMENTAL DESIGN: We investigated the clinical impact of gene expression profiling (GEP)-derived expression levels of NR3C1 in 351 patients with GEP data available at baseline and in 130 with data available at relapse, among 668 subjects accrued to total therapy 2 (TT2). RESULTS: Low NR3C1 expression levels had a negative impact on progression-free survival (PFS; HR, 1.47; P = 0.030) and overall survival (OS; HR, 1.90; P = 0.002) in the no-thalidomide arm. Conversely, there was a significant clinical benefit of thalidomide for patients with low receptor levels (OS: HR, 0.54; P = 0.015; PFS: HR, 0.54; P = 0.004), mediated most likely by thalidomide's upregulation of NR3C1. In the context of both baseline and relapse parameters, post-relapse survival (PRS) was adversely affected by low NR3C1 levels at relapse in a multivariate analysis (HR, 2.61; P = 0.012). CONCLUSION: These findings justify the inclusion of NR3C1 expression data in the work-up of patients with myeloma as it can significantly influence the choice of therapy and, ultimately, OS. The identification of an interaction term between thalidomide and NR3C1 underscores the importance of pharmacogenomic studies in the systematic study of new drugs.

28 [58]. Hjorth, M., O. Hjertner, et al. (2012). "Thalidomide and dexamethasone vs. bortezomib and dexamethasone for melphalan refractory myeloma: a randomized study." Eur J Haematol **88**(6): 485-496.

OBJECTIVES: Thalidomide and bortezomib have been frequently used for second-line therapy in patients with myeloma relapsing after or refractory to initial melphalan-based treatment, but no randomized trials have been published comparing these two treatment alternatives. METHODS:

Thalidomide- and bortezomib-naive patients with melphalan refractory myeloma were randomly assigned to low-dose thalidomide + dexamethasone (Thal-Dex) or bortezomib + dexamethasone (Bort-Dex). At progression on either therapy, the patients were offered crossover to the alternative drug combination. An estimated 300 patients would be needed for the trial to detect a 50% difference in median PFS between the treatment arms. RESULTS: After inclusion of 131 patients, the trial was prematurely closed because of low accrual. Sixty-seven patients were randomized to Thal-Dex and 64 to Bort-Dex. Progression-free survival was similar (median, 9.0 months for Thal-Dex and 7.2 for Bort-Dex). Response rate was similar (55% for Thal-Dex and 63% for Bort-Dex), but time to response was shorter (P < 0.05) and the VGPR rate higher (P < 0.01) for Bort-Dex. Time-to-other treatment after crossover was similar (median, 13.2 months for Thal-Dex and 11.2 months for Bort-Dex), as was overall survival (22.8 months for Thal-Dex and 19.0 for Bort-Dex). Venous thromboembolism was seen in seven patients and cerebrovascular events in four patients in the Thal-Dex group. Severe neuropathy, reactivation of herpes virus infections, and mental depression were more frequently observed in the Bort-Dex group. In the quality-of-life analysis, no difference was noted for physical function, pain, and global quality of life. Fatigue and sleep disturbances were significantly more prevalent in the Bort-Dex group. CONCLUSIONS: Thalidomide (50-100 mg daily) in combination with dexamethasone seems to have an efficacy comparable with that of bortezomib and dexamethasone in melphalan refractory myeloma. However, the statistical strength of the results in this study is limited by the low number of included patients.

29 [59]. Ho, P. J., R. D. Brown, et al. (2012). "Thalidomide consolidation improves progression-free survival in myeloma with normal but not up-regulated expression of fibroblast growth factor receptor 3: analysis from the Australasian Leukaemia and Lymphoma Group MM6 clinical trial." Leuk Lymphoma **53**(9):
1728-1734.

The translocation t(4;14) is associated with a poor prognosis in myeloma, but its effect in the setting of new drugs such as thalidomide, bortezomib and lenalidomide continues to be investigated, and the role of candidate genes such as FGFR3 (fibroblast growth factor receptor 3) is not yet clarified. In the Australasian Leukaemia and Lymphoma Group (ALLG) MM6 randomized study comparing consolidation thalidomide and prednisolone with prednisolone alone following autologous stem cell transplant, patients on consolidation thalidomide and prednisolone had

superior progression-free (PFS) and overall survival (OS). We now show that thalidomide consolidation benefited both t(4;14)-positive (PFS 29 vs. 17 months, p =0.03) and -negative (52 vs. 24 months, p =0.04) disease. PFS for patients with normal FGFR3 expression was significantly better than for those with up-regulated FGFR3 (31 vs. 21 months, p =0.02). Consolidation thalidomide conferred an improved PFS in patients with normal FGFR3 expression (41 vs. 19 months, p =0.02), but there was no improvement in patients with up-regulated FGFR3 (31 vs. 29 months, p =0.76). We conclude that consolidation thalidomide may mitigate the poor prognostic effect of t(4;14), and improves PFS in normal but not up-regulated FGFR3 expression. Thus the level of FGFR3 expression provides additional prognostic information to t(4;14) in myeloma induction and consolidation therapy.

30 [127]. Hulin, C., T. Facon, et al. (2009). "Efficacy of melphalan and prednisone plus thalidomide in patients older than 75 years with newly diagnosed multiple myeloma: IFM 01/01 trial." J Clin Oncol **27**(22): 3664-3670.

PURPOSE: Until recently, melphalan and prednisone were the standards of care in elderly patients with multiple myeloma. The addition of thalidomide to this combination demonstrated a survival benefit for patients age 65 to 75 years. This randomized, placebo-controlled, phase III trial investigated the efficacy of melphalan and prednisone plus thalidomide in patients older than 75 years with newly diagnosed myeloma. PATIENTS AND METHODS: Between April 2002 and December 2006, 232 previously untreated patients with myeloma, age 75 years or older, were enrolled and 229 were randomly assigned to treatment. All patients received melphalan (0.2 mg/kg/d) plus prednisone (2 mg/kg/d) for 12 courses (day 1 to 4) every 6 weeks. Patients were randomly assigned to receive 100 mg/d of oral thalidomide (n = 113) or placebo (n = 116), continuously for 72 weeks. The primary end point was overall survival. RESULTS: After a median follow-up of 47.5 months, overall survival was significantly longer in patients who received melphalan and prednisone plus thalidomide compared with those who received melphalan and prednisone plus placebo (median,

44.0 v 29.1 months; P = .028). Progression-free survival was significantly prolonged in the melphalan and prednisone plus thalidomide group (median, 24.1 v 18.5 months; P = .001). Two adverse events were significantly increased in the melphalan and prednisone plus thalidomide group: grade 2 to 4 peripheral neuropathy (20% v 5% in the melphalan and prednisone plus placebo group; P < .001) and grade 3 to 4 neutropenia (23% v 9%; P = .003). CONCLUSION: This trial confirms the

superiority of the combination melphalan and prednisone plus thalidomide over melphalan and prednisone alone for prolonging survival in very elderly patients with newly diagnosed myeloma. Toxicity was acceptable.

31 [68]. Krishnan, A., M. C. Pasquini, et al. (2011). "Autologous haemopoietic stem-cell transplantation followed by allogeneic or autologous haemopoietic stem-cell transplantation in patients with multiple myeloma (BMT CTN 0102): a phase 3 biological assignment trial." Lancet Oncol **12**(13): 1195-1203.

BACKGROUND: Autologous haemopoietic stem-cell transplantation (HSCT) improves survival in patients with multiple myeloma, but disease progression remains an issue. Allogeneic HSCT might reduce disease progression, but can be associated with high treatment-related mortality. Thus, we aimed to assess effectiveness of allogeneic HSCT with non-myeloablative conditioning after autologous HSCT compared with tandem autologous HSCT. METHODS: In our phase 3 biological assignment trial, we enrolled patients with multiple myeloma attending 37 transplant centres in the USA. Patients (<70 years old) with adequate organ function who had completed at least three cycles of systemic antimyeloma therapy within the past 10 months were eligible for inclusion. We assigned patients to receive an autologous HSCT followed by an allogeneic HSCT (auto-allo group) or tandem autologous HSCTs (auto-auto group) on the basis of the availability of an HLA-matched sibling donor. Patients in the auto-auto group subsequently underwent a random allocation (1:1) to maintenance therapy (thalidomide plus dexamethasone) or observation. To avoid enrolment bias, we classified patients as standard risk or high risk on the basis of cytogenetics and beta2-microglobulin concentrations. We used the Kaplan-Meier method to estimate differences in 3-year progression-free survival (PFS; primary endpoint) between patients with standard-risk disease in the auto-allo group and the best results from the auto-auto group (maintenance, observation, or pooled). This study is registered with ClinicalTrials.gov, number NCT00075829. FINDINGS: Between Dec 17, 2003, and March 30, 2007, we enrolled 710 patients, of whom 625 had standard-risk disease and received an autologous HSCT. 156 (83%) of 189 patients with standard-risk disease in the auto-allo group and 366 (84%) of 436 in the auto-auto group received a second transplant.

219 patients in the auto-auto group were randomly assigned to observation and 217 to receive maintenance treatment, of whom 168 (77%) completed this treatment. PFS and overall survival did not differ between maintenance and observation groups and pooled data were

used. Kaplan-Meier estimates of 3-year PFS were 43% (95% CI 36-51) in the auto-allo group and 46% (42-51) in the auto-auto group (p=0.671); overall survival also did not differ at 3 years (77% [95% CI 72-84] vs 80% [77-84]; p=0.191). Within 3 years, 87 (46%) of 189 patients in the auto-allo group had grade 3-5 adverse events as did 185 (42%) of 436 patients in the auto-auto group. The adverse events that differed most between groups were hyperbilirubinaemia (21 [11%] patients in the auto-allo group vs 14 [3%] in the auto-auto group) and peripheral neuropathy (11 [6%] in the auto-allo group vs 52 [12%] in the auto-auto group). INTERPRETATION: Non-myeloablative allogeneic HSCT after autologous HSCT is not more effective than tandem autologous HSCT for patients with standard-risk multiple myeloma. Further enhancement of the graft versus myeloma effect and reduction in transplant-related mortality are needed to improve the allogeneic HSCT approach. FUNDING: US National Heart, Lung, and Blood Institute and the National Cancer Institute.

32 [62]. Kropff, M., H. G. Baylon, et al. (2012). "Thalidomide versus dexamethasone for the treatment of relapsed and/or refractory multiple myeloma: results from OPTIMUM, a randomized trial." Haematologica **97**(5): 784-791.

BACKGROUND: Thalidomide has potent antimyeloma activity, but no prospective, randomized controlled trial has evaluated thalidomide monotherapy in patients with relapsed/refractory multiple myeloma. DESIGN AND METHODS: We conducted an international, randomized, open-label, four-arm, phase III trial to compare three different doses of thalidomide (100, 200, or 400 mg/day) with standard dexamethasone in patients who had received one to three prior therapies. The primary end-point was time to progression. RESULTS: In the intent-to-treat population (N=499), the median time to progression was 6.1, 7.0, 7.6, and 9.1 months in patients treated with dexamethasone, and thalidomide 100, 200, and 400 mg/day, respectively; the difference between treatment groups was not statistically significant. In the per-protocol population (n=465), the median time to progression was 6.0, 7.0, 8.0, and 9.1 months, respectively. In patients who had received two or three prior therapies, thalidomide significantly prolonged the time to progression at all dose levels compared to the result achieved with dexamethasone. Response rates and median survival were similar in all treatment groups, but the median duration of response was significantly longer in all thalidomide groups than in the dexamethasone group. Adverse events reported in the thalidomide groups, such as fatigue, constipation and neuropathy, confirmed the known safety profile of thalidomide. CONCLUSIONS: Although thalidomide

was not superior to dexamethasone in this randomized trial, thalidomide monotherapy may be considered an effective salvage therapy option for patients with relapsed/refractory multiple myeloma, particularly those with a good prognosis and those who have received two or three prior therapies. The recommended starting dose of thalidomide monotherapy is 400 mg/day, which can be rapidly reduced for patients who do not tolerate this treatment. (CLINICAL TRIAL REGISTRATION NUMBER: NCT00452569).

33 [57]. Kumar, S., I. Flinn, et al. (2012). "Randomized, multicenter, phase 2 study (EVOLUTION) of combinations of bortezomib, dexamethasone, cyclophosphamide, and lenalidomide in previously untreated multiple myeloma." Blood **119**(19): 4375-4382.

Combinations of bortezomib (V) and dexamethasone (D) with either lenalidomide (R) or cyclophosphamide (C) have shown significant efficacy. This randomized phase 2 trial evaluated VDC, VDR, and VDCR in previously untreated multiple myeloma (MM). Patients received V 1.3 mg/m2 (days 1, 4, 8, 11) and D 40 mg (days 1, 8, 15), with either C 500 mg/m2 (days 1, 8) and R 15 mg (days 1-14; VDCR), R 25 mg (days 1-14; VDR), C 500 mg/m2 (days 1, 8; VDC) or C 500 mg/m2 (days 1, 8, 15; VDC-mod) in 3-week cycles (maximum 8 cycles), followed by maintenance with V 1.3 mg/m2 (days 1, 8, 15, 22) for four 6-week cycles (all arms)>/=very good partial response was seen in 58%, 51%, 41%, and 53% (complete response rate of 25%, 24%, 22%, and 47%) of patients (VDCR, VDR, VCD, and VCD-mod, respectively); the corresponding 1-year progression-free survival was 86%, 83%, 93%, and 100%, respectively. Common adverse events included hematologic toxicities, peripheral neuropathy, fatigue, and gastrointestinal disturbances. All regimens were highly active and well tolerated in previously untreated MM, and, based on this trial, VDR and VCD-mod are preferred for clinical practice and further comparative testing. No substantial advantage was noted with VDCR over the 3-drug combinations. This trial is registered at www.clinicaltrials.gov (NCT00507442).

34 [75]. Larocca, A., F. Cavallo, et al. (2012). "Aspirin or enoxaparin thromboprophylaxis for patients with newly diagnosed multiple myeloma treated with lenalidomide." Blood **119**(4): 933-939; quiz 1093.

Lenalidomide plus dexamethasone is effective in the treatment of multiple myeloma (MM) but is associated with an increased risk of venous thromboembolism (VTE). This prospective, open-label, randomized substudy of a phase 3 trial compared the efficacy and safety of

thromboprophylaxis with low-dose aspirin (ASA) or low-molecular-weight heparin (LMWH) in patients with newly diagnosed MM, treated with lenalidomide and low-dose dexamethasone induction and melphalan-prednisone-lenalidomide consolidation. Overall, 342 patients who did not have clinical indications or contraindications to antiplatelet or anticoagulant therapy were randomly assigned to receive ASA 100 mg/d (n = 176) or LMWH enoxaparin 40 mg/d (n = 166). The incidence of VTE was
2.27% in the ASA group and 1.20% in the LMWH group. Compared with LMWH, the absolute difference in the proportion of VTE was 1.07% (95% confidence interval, -1.69-3.83; P = .452) in the ASA group. Pulmonary embolism was observed in 1.70% of patients in the ASA group and none in the LMWH group. No arterial thrombosis, acute cardiovascular events, or sudden deaths were reported. No major hemorrhagic complications were reported. In previously untreated patients with MM receiving lenalidomide with a low thromboembolic risk, ASA could be an effective and less-expensive alternative to LMWH thromboprophylaxis.

35 [25]. Leleu, X., M. Attal, et al. (2013). "Pomalidomide plus low-dose dexamethasone is active and well tolerated in bortezomib and lenalidomide-refractory multiple myeloma: Intergroupe Francophone du Myelome 2009-02." Blood **121**(11): 1968-1975.

The combination of pomalidomide and dexamethasone can be safely administered to patients with multiple myeloma (MM) and has significant efficacy, although the optimal regimen remains to be determined. Patients with MM whose disease progressed after multiple lines of therapy have limited treatment options. We designed a multicenter, phase 2 randomized study assessing two different dose regimens of pomalidomide and dexamethasone in advanced MM. Treatment response was assessed centrally. Pomalidomide (4 mg) was given orally on days 1 to 21 (arm 21/28) or continuously (arm 28/28) over a 28-day cycle, plus dexamethasone given weekly. Eighty-four patients (43, arm 21/28 and 41, arm 28/28) were randomized. The median number of prior lines was 5. Overall response rate was 35% (arm 21/28) and 34% (arm 28/28), independent of the number of prior lines and level of refractoriness. Median duration of response, time to disease progression, and progression-free survival was 7.3, 5.4, and 4.6 months, respectively, which was similar across cohorts. At 23 months follow-up, median overall survival was 14.9 months, with 44% of the patients alive at 18 months. Toxicity consisted primarily of myelosuppression, which was manageable. The efficacy and safety data presented here, along with data from other

phase 2 trials, suggest that pomalidomide 4 mg per day on days 1 to 21 of 28 with dexamethasone should be investigated in future trials. This trial is registered at ClinicalTrials.gov (No. NCT01053949).

36 [146]. Lokhorst, H. M., I. Schmidt-Wolf, et al. (2008). "Thalidomide in induction treatment increases the very good partial response rate before and after high-dose therapy in previously untreated multiple myeloma." Haematologica **93**(1): 124-127.

In the prospective phase 3 HOVON-50/GMMG-HD3 trial, patients randomized to TAD (thalidomide, doxorubicin, dexamethasone) had a significantly higher response rate (at least PR) after induction compared with patients randomized to VAD (vincristine, adriamycin, dexamethasone, 72% vs. 54%, p<0.001). Complete remission (CR) and very good partial remission (VGPR) were also higher after TAD. After High Dose melphalan 200mg/m(2) response was comparable in both arms, 76% and 79% respectively. However, CR plus VGPR were significantly higher in the patients randomized to TAD (49% vs. 32%, p<0.001). CTC grade 3-4 adverse events were similar in both arms.

37 [116]. Lokhorst, H. M., B. van der Holt, et al. (2010). "A randomized phase 3 study on the effect of thalidomide combined with adriamycin, dexamethasone, and high-dose melphalan, followed by thalidomide maintenance in patients with multiple myeloma." Blood **115**(6): 1113-1120.

The phase 3 trial HOVON-50 was designed to evaluate the effect of thalidomide during induction treatment and as maintenance in patients with multiple myeloma who were transplant candidates. A total of 556 patients was randomly assigned to arm A: 3 cycles of vincristine, adriamycin, and dexamethasone, or to arm B: thalidomide 200 mg orally, days 1 to 28 plus adriamycin and dexamethasone. After induction therapy and stem cell mobilization, patients were to receive high-dose melphalan, 200 mg/m(2), followed by maintenance with alpha-interferon (arm A) or thalidomide 50 mg daily (arm B). Thalidomide significantly improved overall response rate as well as quality of the response before and after high dose melphalan. Best overall response rate on protocol was 88% and 79% (P = .005), at least very good partial remission 66% and 54% (P = .005), and complete remission 31% and 23% (P = .04), respectively, in favor of the thalidomide arm. Thalidomide also significantly improved event-free survival from median 22 months to 34 months (P < .001), and prolonged progression free from median 25 months to 34 months (P < .001). Median survival was longer in the thalidomide arm, 73 versus 60 months; however, this difference was not significant (P = .77). Patients randomized to

thalidomide had strongly reduced survival after relapse. This trial was registered on www.controlled-trials.com as ISRCTN06413384.

38 [106]. Ludwig, H., Z. Adam, et al. (2010). "Thalidomide maintenance treatment increases progression-free but not overall survival in elderly patients with myeloma." Haematologica **95**(9): 1548-1554.

BACKGROUND: Thalidomide maintenance therapy after stem cell transplantation resulted in increased progression-free survival and overall survival in a few trials, but its role in non-transplant eligible patients with multiple myeloma remains unclear. This study assessed the impact of thalidomide-interferon in comparison to interferon maintenance therapy in elderly patients with multiple myeloma. DESIGN AND METHODS: Of 289 elderly patients with multiple myeloma who were randomized to thalidomide-dexamethasone or melphalan-prednisolone induction therapy, 137 finally completed 9 cycles of induction therapy with stable disease or better and thereby qualified for maintenance treatment. Of these, 128 have been randomized to either thalidomide-interferon or interferon alone. Primary study endpoints were progression-free survival and response rates; secondary endpoints were overall survival, toxicity and quality of life. RESULTS: Thalidomide-interferon maintenance therapy led to a significantly longer progression-free survival compared to interferon (27.7 vs. 13.2 months, P=0.0068), but overall survival was similar in both groups (52.6 vs. 51.4 months, P=0.81) and did not differ between patients aged 75 years or older, or younger patients (P=0.39). Survival after disease progression tended to be shorter in patients on thalidomide-interferon maintenance therapy (P=0.056). Progression-free survival and overall survival tended to be shorter in patients with adverse cytogenetic (FISH) findings compared to the standard risk group but differences were not significant (P=0.084 and P=0.082, respectively). Patients on thalidomide-interferon presented with more neuropathy (P=0.0015), constipation (P=0.0004), skin toxicity (P=0.0041) and elevated creatinine (P=0.026). CONCLUSIONS: Thalidomide plus interferon maintenance therapy increased progression-free survival but not overall survival and was associated with slightly more toxicity than maintenance with interferon alone. (ClinicalTrials.gov Identifier: NCT00205751).

39 [132]. Ludwig, H., R. Hajek, et al. (2009). "Thalidomide-dexamethasone compared with melphalan-prednisolone in elderly patients with multiple myeloma." Blood **113**(15): 3435-3442.

We compared thalidomide-dexamethasone (TD) with

melphalan-prednisolone (MP) in 289 elderly patients with multiple myeloma (MM). Patients received either thalidomide 200 mg plus dexamethasone 40 mg, days 1 to 4 and 15 to 18 on even cycles and days 1 to 4 on odd cycles, during a 28-day cycle or to melphalan 0.25 mg/kg and prednisolone 2 mg/kg orally on days 1 to 4 during a 28- to 42-day cycle. Patients achieving stable disease or better were randomly assigned to maintenance therapy with either thalidomide 100 mg daily and 3 MU interferon alpha-2b thrice weekly or to 3 MU interferon alpha-2b thrice weekly only. TD resulted in a higher proportion of complete and very good remissions (26% vs 13%; P= .006) and overall responses (68% vs 50%; P= .002) compared with MP. Time to progression (21.2 vs 29.1 months; P= .2), and progression-free survival was similar (16.7 vs 20.7 months; P= .1), but overall survival was significantly shorter in the TD group (41.5 vs 49.4 months; P= .024). Toxicity was higher with TD, particularly in patients older than 75 years with poor performance status. The study was registered at ClinicalTrials.gov as NCT00205751.

40 [33]. Ludwig, H., L. Viterbo, et al. (2013). "Randomized phase II study of bortezomib, thalidomide, and dexamethasone with or without cyclophosphamide as induction therapy in previously untreated multiple myeloma." J Clin Oncol **31**(2): 247-255.

PURPOSE: Bortezomib-thalidomide-dexamethasone (VTD) is an effective induction therapy in multiple myeloma (MM). This phase II, noncomparative study sought to determine whether addition of cyclophosphamide to this regimen (VTDC) could further increase efficacy without compromising safety. PATIENTS AND METHODS: Patients age 18 to 70 years with previously untreated, measurable MM, who were eligible for high-dose chemotherapy-autologous stem-cell transplantation (HDCT-ASCT), were randomly assigned to bortezomib 1.3 mg/m(2), thalidomide 100 mg, and dexamethasone 40 mg, with (n = 49) or without (n = 49) cyclophosphamide 400 mg/m(2) for four 21-day cycles, followed by HDCT-ASCT. The primary end point was postinduction combined rate of near-complete response (nCR) or better (including complete response [CR] with normalized serum kappa:lambda free light chain ratio, CR, and nCR). RESULTS: Postinduction, 51% (VTD) and 44% (VTDC) of patients achieved combined CR/nCR, with bone marrow-confirmed CR in 29% and 31%, overall response rates of 100% and 96%, respectively, and very good partial response or better rates of 69% per arm. Post-HDCT-ASCT, combined CR/nCR rates were 85% (VTD) and 77% (VTDC). In all, 35% (VTD) and 27% (VTDC) of patients were negative for minimal residual disease (MRD) during induction and postinduction. Three-year overall survival was

80% (both arms). Grade 3 to 4 adverse events (AEs) and serious AEs were observed in 47% and 22% (VTD) and 57% and 41% (VTDC) of patients, respectively. The primary health-related quality of life end point (European Organisation for Research and Treatment of Cancer Quality of Life Questionnaire C30 [EORTC QLQ-C30] Global Health score) steadily increased with VTD during induction and reached a clinically relevant difference post-transplantation versus baseline. CONCLUSION: Both VTD and VTDC are highly active induction regimens producing high combined CR/nCR and MRD-negative rates; however, VTDC was associated with increased toxicity and suggestion of transient decreases in Global Health score, without an increase in activity.

41 [49]. Maiolino, A., V. T. Hungria, et al. (2012). "Thalidomide plus dexamethasone as a maintenance therapy after autologous hematopoietic stem cell transplantation improves progression-free survival in multiple myeloma." Am J Hematol **87**(10): 948-952.

Despite the good response of stem cell transplant (SCT) in the treatment of multiple myeloma (MM), most patients relapse or do not achieve complete remission, suggesting that additional treatment is needed. We assessed the impact of thalidomide in maintenance after SCT in untreated patients with MM. A hundred and eight patients (<70 years old) were randomized to receive maintenance with dexamethasone (arm A; n = 52) or dexamethasone with thalidomide (arm B; n = 56; 200 mg daily) for 12 months or until disease progression. After a median follow-up of 27 months, an intention to treat analysis showed a 2-year progression-free survival (PFS) of 30% in arm A (95% CI 22-38) and 64% in arm B (95% CI 57-71; P = 0.002), with median PFS of 19 months and 36 months, respectively. In patients who did not achieve at least a very good partial response, the PFS at 2 years was significantly higher when in use of thalidomide (19 vs. 59%; P = 0.002). Overall survival at 2 years was not significantly improved (70 vs. 85% in arm A and arm B, respectively; P = 0.27). The addition of thalidomide to dexamethasone as maintenance improved the PFS mainly in patients who did not respond to treatment after SCT.

42 [14]. Mateos, M. V., M. T. Hernandez, et al. (2013). "Lenalidomide plus dexamethasone for high-risk smoldering multiple myeloma." N Engl J Med **369**(5):
438-447.

BACKGROUND: For patients with smoldering multiple myeloma, the standard of care is observation until symptoms develop. However, this approach does not identify high-risk patients who may benefit from early

intervention. METHODS: In this randomized, open-label, phase 3 trial, we randomly assigned 119 patients with high-risk smoldering myeloma to treatment or observation. Patients in the treatment group received an induction regimen (lenalidomide at a dose of 25 mg per day on days 1 to 21, plus dexamethasone at a dose of 20 mg per day on days 1 to 4 and days 12 to 15, at 4-week intervals for nine cycles), followed by a maintenance regimen (lenalidomide at a dose of 10 mg per day on days 1 to 21 of each 28-day cycle for 2 years). The primary end point was time to progression to symptomatic disease. Secondary end points were response rate, overall survival, and safety. RESULTS: After a median follow-up of 40 months, the median time to progression was significantly longer in the treatment group than in the observation group (median not reached vs. 21 months; hazard ratio for progression, 0.18; 95% confidence interval [CI], 0.09 to 0.32; P<0.001). The 3-year survival rate was also higher in the treatment group (94% vs. 80%; hazard ratio for death, 0.31; 95% CI, 0.10 to 0.91; P=0.03). A partial response or better was achieved in 79% of patients in the treatment group after the induction phase and in 90% during the maintenance phase. Toxic effects were mainly grade 2 or lower. CONCLUSIONS: Early treatment for patients with high-risk smoldering myeloma delays progression to active disease and increases overall survival. (Funded by Celgene; ClinicalTrials.gov number, NCT00480363.).

43 [98]. Mateos, M. V., A. Oriol, et al. (2010). "Bortezomib, melphalan, and prednisone versus bortezomib, thalidomide, and prednisone as induction therapy followed by maintenance treatment with bortezomib and thalidomide versus bortezomib and prednisone in elderly patients with untreated multiple myeloma: a randomised trial." Lancet Oncol **11**(10): 934-941.

BACKGROUND: Bortezomib plus melphalan and prednisone (VMP) is significantly better than melphalan plus prednisone alone for elderly patients with untreated multiple myeloma; however, toxic effects are high. We investigated a novel and less intensive bortezomib-based regimen to maintain efficacy and to reduce toxic effects. METHODS: Between March, 2006, and October, 2008, 260 patients with untreated multiple myeloma, 65 years and older, from 63 Spanish centres, were randomly assigned to receive six cycles of VMP (n=130) or bortezomib plus thalidomide and prednisone (VTP; n=130) as induction therapy, consisting of one cycle of bortezomib twice per week for 6 weeks (1.3 mg/m(2) on days 1, 4, 8, 11, 22, 25, 29, and 32), plus either melphalan (9 mg/m(2) on days 1-4) or daily thalidomide (100 mg), and prednisone (60 mg/m(2) on days 1-4). The first cycle was followed by five cycles of bortezomib once

per week for 5 weeks (1.3 mg/m(2) on days 1, 8, 15, and 22) plus the same doses of melphalan plus prednisone and thalidomide plus prednisone. 178 patients completed the six induction cycles and were randomly assigned to maintenance therapy with bortezomib plus prednisone (n=87) or bortezomib plus thalidomide (n=91), consisting of one conventional cycle of bortezomib for 3 weeks (1.3 mg/m(2) on days 1, 4, 8, and 11) every 3 months, plus either prednisone (50 mg every other day) or thalidomide (50 mg per day), for up to 3 years. Treatment codes were generated with a computerised random number generator, and neither participants nor study personnel were masked to treatment. The primary endpoint was response rate in induction and maintenance phases. Analysis was by intention to treat. This trial is registered with ClinicalTrials.gov, number NCT00443235. FINDINGS: In the induction phase, 105 (81%) patients in the VTP group and 104 (80%) in the VMP group achieved partial responses or better (p=0.9), including 36 (28%) and 26 (20%) complete remissions, respectively (p=0.2). Treatment with VTP resulted in more serious adverse events (40 [31%] vs 20 [15%], p=0.01) and discontinuations (22 [17%] vs 15 [12%], p=0.03) than did treatment with VMP. The most common toxicities (grade 3 or worse) were infections (one [1%] in the VTP group vs nine [7%] in the VMP group), cardiac events (11 [8%] vs 0), and peripheral neuropathy (nine [7%] vs 12 [9%]). After maintenance therapy, the complete remission rate was 42% (40 [44%] patients in complete remission in the bortezomib plus thalidomide group, 34 [39%] in the bortezomib plus prednisone group). No grade 3 or worse haematological toxicities were recorded during maintenance therapy; two (2%) patients in the bortezomib plus prednisone group and six (7%) in the bortezomib plus thalidomide group developed peripheral neuropathy. INTERPRETATION: Reduced-intensity induction with a bortezomib-based regimen, followed by maintenance, is a safe and effective treatment for elderly patients with multiple myeloma. FUNDING: Pethema (Spanish Program for the Treatment of Hematologic Diseases).

44 [42]. Mateos, M. V., A. Oriol, et al. (2012). "Maintenance therapy with bortezomib plus thalidomide or bortezomib plus prednisone in elderly multiple myeloma patients included in the GEM2005MAS65 trial." Blood **120**(13): 2581-2588.

Maintenance therapy has become a hot field in myeloma, and it may be particularly relevant in elderly patients because the major benefit results from the initial therapy. We report the results of a randomized comparison of maintenance with bortezomib plus thalidomide (VT) or prednisone (VP) in 178 elderly untreated myeloma patients who had received 6 induction

cycles with bortezomib plus either melphalan and prednisone or thalidomide and prednisone. The complete response (CR) rate increased from 24% after induction up to 42%, higher for VT versus VP (46% vs 39%). Median progression-free survival (PFS) was superior for VT (39 months) compared with VP (32 months) and overall survival (OS) was also longer in VT patients compared with VP (5-year OS of 69% and 50%, respectively) but the differences did not reach statistical significance. CR achievement was associated with a significantly longer PFS (P < .001) and 5-year OS (P < .001). The incidence of G3-4 peripheral neuropathy was 9% for VT and 3% for VP. Unfortunately, this approach was not able to overcome the adverse prognosis of cytogenetic abnormalities. In summary, these maintenance regimens result in a significant increase in CR rate, remarkably long PFS, and acceptable toxicity profile. The trial is registered at www.clinicaltrials.gov as NCT00443235.

45 [53]. McCarthy, P. L., K. Owzar, et al. (2012). "Lenalidomide after stem-cell transplantation for multiple myeloma." N Engl J Med **366**(19): 1770-1781.

BACKGROUND: Data are lacking on whether lenalidomide maintenance therapy prolongs the time to disease progression after autologous hematopoietic stem-cell transplantation in patients with multiple myeloma. METHODS: Between April 2005 and July 2009, we randomly assigned 460 patients who were younger than 71 years of age and had stable disease or a marginal, partial, or complete response 100 days after undergoing stem-cell transplantation to lenalidomide or placebo, which was administered until disease progression. The starting dose of lenalidomide was 10 mg per day (range, 5 to 15). RESULTS: The study-drug assignments were unblinded in 2009, when a planned interim analysis showed a significantly longer time to disease progression in the lenalidomide group. At unblinding, 20% of patients who received lenalidomide and 44% of patients who received placebo had progressive disease or had died (P<0.001); of the remaining 128 patients who received placebo and who
did not have progressive disease, 86 crossed over to lenalidomide. At a median follow-up of 34 months, 86 of 231 patients who received lenalidomide (37%) and 132 of 229 patients who received placebo (58%) had disease progression or had died. The median time to progression was 46 months in the lenalidomide group and 27 months in the placebo group (P<0.001). A total of 35 patients who received lenalidomide (15%) and 53 patients who received placebo (23%) died (P=0.03). More grade 3 or 4 hematologic adverse events and grade 3 nonhematologic adverse events occurred in patients who received lenalidomide (P<0.001 for both

comparisons). Second primary cancers occurred in 18 patients who received lenalidomide (8%) and 6 patients who received placebo (3%). CONCLUSIONS: Lenalidomide maintenance therapy, initiated at day 100 after hematopoietic stem-cell transplantation, was associated with more toxicity and second cancers but a significantly longer time to disease progression and significantly improved overall survival among patients with myeloma. (Funded by the National Cancer Institute; ClinicalTrials.gov number, NCT00114101.).

46 [70]. Morabito, F., M. Gentile, et al. (2011). "Safety and efficacy of bortezomib-melphalan-prednisone-thalidomide followed by bortezomib-thalidomide maintenance (VMPT-VT) versus bortezomib-melphalan-prednisone (VMP) in untreated multiple myeloma patients with renal impairment." Blood **118**(22): 5759-5766.

We assessed efficacy, safety, and reversal of renal impairment (RI) in untreated patients with multiple myeloma given bortezomib-melphalan-prednisone-thalidomide followed by bortezomib-thalidomide (VMPT-VT) maintenance or bortezomib-melphalan-prednisone (VMP). Exclusion criteria included serum creatinine >/= 2.5 mg/dL. In the VMPT-VT/VMP arms, severe RI (estimated glomerular filtration rate [eGFR] </= 30 mL/min), moderate RI (eGFR 31-50 mL/min), and normal renal function (eGFR > 50 mL/min), were 6%/7.9%, 24.1%/24.9%, and 69.8%/67.2%, respectively. Statistically significant improvements in overall response rates and progression-free survival were observed in VMPT-VT versus VMP arms across renal cohorts, except in severe RI patients. In the VMPT group, severe RI reduced overall survival (OS). RI was reversed in 16/63 (25.4%) patients receiving VMPT-VT versus 31/77 (40.3%) receiving VMP. Multivariate analysis showed male sex (P = .022) and moderate RI (P = .003) significantly predicted RI recovery. VMP patients achieving renal response showed longer OS. In both arms, greater rates of severe hematologic adverse events were associated with RI (eGFR < 50 mL/min), however, therapy discontinuation rates were unaffected. VMPT-VT was superior to VMP for cases with normal renal function and moderate RI, whereas VMPT-VT failed to outperform VMP in patients with severe RI, although the relatively low number of cases analyzed preclude drawing definitive conclusions. VMPT-VT had no advantage in terms of RI reversal over VMP.

47 [73]. Moreau, P., H. Avet-Loiseau, et al. (2011). "Bortezomib plus dexamethasone versus reduced-dose bortezomib, thalidomide plus dexamethasone as induction treatment before autologous stem cell

transplantation in newly diagnosed multiple myeloma." Blood **118**(22): 5752-5758; quiz 5982.

The Intergroupe Francophone du Myelome conducted a randomized trial to compare bortezomib-dexamethasone (VD) as induction before high-dose therapy (HDT) and autologous stem cell transplantation (ASCT) to a combination consisting of reduced doses of bortezomib and thalidomide plus dexamethasone (vtD) in patients with multiple myeloma. Overall, a total of 199 patients were centrally randomly assigned to receive VD or vtD. After 4 cycles, the complete response (CR) rate was the same in both groups (13% in the vtD arm, 12% in the VD arm, P = .74). However, the CR plus very good partial response (VGPR) rate was significantly higher in the vtD arm (49% vs 36%, P = .05). After ASCT, the CR plus VGPR rate was significantly higher in the vtD arm (74% vs 58%, P = .02). The reduced doses of bortezomib and thalidomide translated into a reduced incidence of peripheral neuropathy (PN): grade >/= 2 PN were reported in 34% in the VD arm versus 14% in the vtD arm (P = .001). vtD, including reduced doses of bortezomib and thalidomide, yields higher VGPR rates compared with VD and can be considered a new effective triplet combination before HDT/ASCT.

48 [11]. Morgan, G. J., F. E. Davies, et al. (2013). "Long-term follow-up of MRC Myeloma IX trial: Survival outcomes with bisphosphonate and thalidomide treatment." Clin Cancer Res **19**(21): 6030-6038.

PURPOSE: Medical Research Council (MRC) Myeloma IX was a phase III trial evaluating bisphosphonate and thalidomide-based therapy for newly diagnosed multiple myeloma. Results were reported previously after a median follow-up of 3.7 years (current controlled trials number: ISRCTN68454111). Survival outcomes were reanalyzed after an extended follow-up (median, 5.9 years). EXPERIMENTAL DESIGN: At first randomization, patients (N = 1,970) were assigned to bisphosphonate (clodronic acid or zoledronic acid) and induction therapies [cyclophosphamide-vincristine-doxorubicin-dexamethasone (CVAD) or cyclophosphamide-thalidomide-dexamethasone (CTD) followed by high-dose therapy plus autologous stem cell transplantation for younger/fitter patients (intensive pathway), and melphalan-prednisone (MP) or attenuated CTD (CTDa) for older/less fit patients (nonintensive pathway)]. At second randomization, patients were assigned to thalidomide maintenance therapy or no maintenance. Interphase FISH (iFISH) was used to analyze cytogenics. RESULTS: Zoledronic acid significantly improved progression-free survival (PFS; HR, 0.89; P = 0.02) and overall survival (OS; HR, 0.86; P = 0.01) compared with clodronic acid. In the

intensive pathway, CTD showed noninferior PFS and OS compared with CVAD, with a trend toward improved OS in patients with favorable cytogenics (P = 0.068). In the nonintensive pathway, CTDa significantly improved PFS (HR, 0.81; P = 0.007) compared with MP and there was an emergent survival benefit after 18 to 24 months. Thalidomide maintenance improved PFS (HR, 1.44; P < 0.0001) but not OS (HR, 0.96; P = 0.70), and was associated with shorter OS in patients with adverse cytogenics (P = 0.01). CONCLUSIONS: Long-term follow-up is essential to identify clinically meaningful treatment effects in myeloma subgroups based on cytogenetics.

49 [65]. Morgan, G. J., F. E. Davies, et al. (2012). "Cyclophosphamide, thalidomide, and dexamethasone as induction therapy for newly diagnosed multiple myeloma patients destined for autologous stem-cell transplantation: MRC Myeloma IX randomized trial results." Haematologica **97**(3): 442-450.

BACKGROUND: Thalidomide is active in multiple myeloma and is associated with minimal myelosuppression, making it a good candidate for induction therapy prior to high-dose therapy with autologous stem-cell transplantation. DESIGN AND METHODS: Oral cyclophosphamide, thalidomide, and dexamethasone was compared with infusional cyclophosphamide, vincristine, doxorubicin, and dexamethasone in patients with newly diagnosed multiple myeloma. RESULTS: The post-induction overall response rate (>/= partial response) for the intent-to-treat population was significantly higher with cyclophosphamide-thalidomide-dexamethasone (n=555) versus cyclophosphamide-vincristine-doxorubicin-dexamethasone (n=556);
82.5% versus 71.2%; odds ratio 1.91; 95% confidence interval 1.44-2.55; P<0.0001. The complete response rates were 13.0% with cyclophosphamide-thalidomide-dexamethasone and 8.1% with cyclophos-phamide-vincristine-doxorubicin-dexamethasone (P=0.0083), with this differential response being maintained in patients who received autologous stem-cell transplantation (post-transplant complete response 50.0% versus 37.2%, respectively; P=0.00052).
Cyclophosphamide-thalidomide-dexamethasone was non-inferior to cyclophosphamide-vincristine-doxorubicin-dexamethasone for progression-free and overall survival, and there was a trend toward a late survival benefit with cyclophosphamide-thalidomide-dexamethasone in responders. A trend toward an overall survival advantage for cyclophosphamide-thalidomide-dexamethasone over cyclophosphamide-vincristine-doxorubicin-dexamethasone was also

observed in a subgroup of patients with favorable interphase fluorescence in situ hybridization. Compared with cyclophosphamide-vincristine-doxorubicin-dexamethasone, cyclophosphamide-thalidomide-dexamethasone was associated with more constipation and somnolence, but a lower incidence of cytopenias. CONCLUSIONS: The cyclophosphamide-thalidomide-dexamethasone regimen showed improved response rates and was not inferior in terms of survival outcomes to the standard infusional regimen of cyclophosphamide-vincristine-doxorubicin-dexamethasone. Based on its oral administration and the reduced incidence of infection and cytopenia, cyclophosphamide-thalidomide-dexa-methasone may be considered an effective induction therapy option for patients with newly diagnosed multiple myeloma. (ISRCTN: 68454111).

50 [82]. Morgan, G. J., F. E. Davies, et al. (2011). "Cyclophosphamide, thalidomide, and dexamethasone (CTD) as initial therapy for patients with multiple myeloma unsuitable for autologous transplantation." Blood **118**(5): 1231-1238.

As part of the randomized MRC Myeloma IX trial, we compared an attenuated regimen of cyclophosphamide, thalidomide, and dexamethasone (CTDa; n = 426) with melphalan and prednisolone (MP; n = 423) in patients with newly diagnosed multiple myeloma ineligible for autologous stem-cell transplantation. The primary endpoints were overall response rate, progression-free survival, and overall survival (OS). The overall response rate was significantly higher with CTDa than MP (63.8% vs 32.6%; P < .0001), primarily because of increases in the rate of complete responses (13.1% vs 2.4%) and very good partial responses (16.9% vs 1.7%). Progression-free survival and OS were similar between groups. In this population, OS correlated with the depth of response (P < .0001) and favorable interphase fluorescence in situ hybridization profile (P < .001). CTDa was associated with higher rates of thromboembolic events, constipation, infection, and neuropathy than MP. In elderly patients with newly diagnosed multiple myeloma (median age, 73 years), CTDa produced higher response rates than MP but was not associated with improved survival outcomes. We highlight the importance of cytogenetic profiling at diagnosis and effective management of adverse events. This trial was registered at International Standard Randomized Controlled Trials Number as #68454111.

51 [56]. Morgan, G. J., F. E. Davies, et al. (2012). "Effects of induction and maintenance plus long-term bisphosphonates on bone disease in patients

with multiple myeloma: the Medical Research Council Myeloma IX Trial." Blood **119**(23): 5374-5383.

The Medical Research Council Myeloma IX Trial (ISRCTNG8454111) examined traditional and thalidomide-based induction and maintenance regimens and IV zoledronic acid (ZOL) and oral clodronate (CLO) in 1960 patients with newly diagnosed multiple myeloma. Overall survival (OS) and skeletal-related event (SRE) data have been reported for the overall trial population. The present analysis investigated optimal therapy regimens for different patient populations in Myeloma IX. Patients were assigned to intensive or nonintensive treatment pathways and randomized to induction cyclophosphamide, vincristine, doxorubicin, and dexamethasone (CVAD) versus cyclophosphamide, thalidomide, and dexamethasone (CTD; intensive) or melphalan and prednisolone versus attenuated oral CTD (CTDa; nonintensive). Patients were also randomized to ZOL or CLO. In the nonintensive pathway, CTDa produced better responses and lower SRE rates than melphalan and prednisolone. ZOL improved OS compared with CLO independently of sex, stage, or myeloma subtype, most profoundly in patients with baseline bone disease or other SREs. In patients treated for >/= 2 years, ZOL improved OS compared with CLO from randomization (median not reached for either; P = .02) and also from first on-study disease progression (median, 34 months for ZOL vs 27 months for CLO; P = .03). Thalidomide-containing regimens had better efficacy than traditional regimens, and ZOL demonstrated greater benefits than CLO.

52 [67]. Morgan, G. J., W. M. Gregory, et al. (2012). "The role of maintenance thalidomide therapy in multiple myeloma: MRC Myeloma IX results and meta-analysis." Blood **119**(1): 7-15.

Thalidomide maintenance has the potential to modulate residual multiple myeloma (MM) after an initial response. This trial compared the effect of thalidomide maintenance and no maintenance on progression-free survival (PFS) and overall survival (OS) in MM patients. After intensive or nonintensive induction therapy, 820 newly diagnosed MM patients were randomized to open-label thalidomide maintenance until progression, or no maintenance. Interphase FISH (iFISH) analysis was performed at study entry. Median PFS was significantly longer with thalidomide maintenance (log-rank P < .001). Median OS was similar between regimens (log-rank P = .40). Patients with favorable iFISH showed improved PFS (P = .004) and a trend toward a late survival benefit. Patients with adverse iFISH receiving thalidomide showed no significant PFS benefit and worse OS (P = .009). Effective relapse therapy enhanced survival after progression, translating

into a significant OS benefit. Meta-analysis of this and other studies show a significant late OS benefit (P < .001, 7-year difference hazard ratio = 12.3; 95% confidence interval, 5.5-19.0). Thalidomide maintenance significantly improves PFS and can be associated with improved OS. iFISH testing is important in assessing the clinical impact of maintenance therapy. Overview analysis demonstrated that thalidomide maintenance was associated with a significant late OS benefit. This trial was registered at www.isrctn.org as #ISRCTN68454111.

53 [112]. Nair, B., F. van Rhee, et al. (2010). "Superior results of Total Therapy 3 (2003-33) in gene expression profiling-defined low-risk multiple myeloma confirmed in subsequent trial 2006-66 with VRD maintenance." Blood **115**(21): 4168-4173.

The Total Therapy 3 trial 2003-33 enrolled 303 newly diagnosed multiple myeloma patients and was noted to provide superior clinical outcomes compared with predecessor trial Total Therapy 2, especially in gene expression profiling (GEP)-defined low-risk disease. We report here on the results of successor trial 2006-66 with 177 patients, using bortezomib, lenalidomide, and dexamethasone maintenance for 3 years versus bortezomib, thalidomide, and dexamethasone in year 1 and thalidomide/dexamethasone in years 2 and 3 in the 2003-33 protocol. Overall survival (OS) and event-free survival (EFS) plots were super-imposable for the 2 trials, as were onset of complete response and complete response duration (CRD), regardless of GEP risk. GEP-defined high-risk designation, pertinent to 17% of patients, imparted inferior OS, EFS, and CRD in both protocols and, on multivariate analysis, was the sole adverse feature affecting OS, EFS, and CRD. Mathematical modeling of CRD in low-risk myeloma predicted a 55% cure fraction (P < .001). Despite more rapid onset and higher rate of CR than in other molecular subgroups, CRD was inferior in CCND1 without CD20 myeloma, resembling outcomes in MAF/MAFB and proliferation entities. The robustness of the GEP risk model should be exploited in clinical trials aimed at improving the notoriously poor outcome in high-risk disease.

54 [131]. Offidani, M., L. Corvatta, et al. (2009). "Thalidomide-dexamethasone versus interferon-alpha-dexamethasone as maintenance treatment after ThaDD induction for multiple myeloma: a prospective, multicentre, randomised study." Br J Haematol **144**(5): 653-659.

Maintenance therapy was explored in multiple myeloma (MM) patients after conventional thalidomide, dexamethasone and pegylated liposomal doxorubicin (ThaDD). Patients with newly or relapsed MM

obtaining at least minor response after 6 ThaDD courses, were randomised to receive alpha-interferon (IFN) 3 MU 3 times a week or thalidomide 100 mg daily until relapse. Both groups also received pulsed dexamethasone 20 mg 4 d a month. Fifty-one patients were randomized in the IFN-dexamethasone (ID) arm and 52 in the thalidomide-dexamethasone (TD) arm. The characteristics of two groups were similar. A significantly better 2-years progression-free survival (PFS; 63% vs. 32%; P = 0.024) and overall survival (84% vs. 68%; P = 0.030) was observed in the thalidomide arm. In high-risk patients and in those achieving less than very good partial response after induction, TD fared better in term of PFS. Main side effects were peripheral neuropathy and constipation in TD group, fatigue, anorexia and haematological toxicity in ID arm. There was a 21% probability of discontinuation at 3 years in the thalidomide arm and 44% in the IFN arm (P = 0.014). Low-dose thalidomide plus pulsed low-dose dexamethasone after conventional thalidomide combination-based therapy was also feasible in the long term, enabling significantly better residual disease control if compared with a standard maintenance therapy.

55 [177]. Palumbo, A., S. Bringhen, et al. (2006). "Oral melphalan and prednisone chemotherapy plus thalidomide compared with melphalan and prednisone alone in elderly patients with multiple myeloma: randomised controlled trial." Lancet **367**(9513): 825-831.

BACKGROUND: Since 1960, oral melphalan and prednisone (MP) has been regarded as the standard of care in elderly multiple myeloma patients. This multicentre randomised trial compared oral MP plus thalidomide (MPT) with MP alone in patients aged 60-85 years. METHODS: Patients with newly diagnosed multiple myeloma were randomly assigned to receive oral MP for six 4-week cycles plus thalidomide (n=129; 100 mg per day continuously until any sign of relapse or progressive disease) or MP alone (n=126). Analysis was intention-to-treat. This study is registered at , number NCT00232934. RESULTS: Patients treated with thalidomide had higher response rates and longer event-free survival (primary endpoints) than patients who were not. Combined complete or partial response rates were 76.0% for MPT and 47.6% for MP alone (absolute difference 28.3%, 95% CI 16.5-39.1), and the near-complete or complete response rates were 27.9% and 7.2%, respectively. 2-year event-free survival rates were 54% for MPT and 27% for MP (hazard ratio [HR] for MPT 0.51, 95% CI 0.35-0.75, p=0.0006). 3-year survival rates were 80% for MPT and 64% for MP (HR for MPT 0.68, 95% CI 0.38-1.22, p=0.19). Rates of grade 3 or 4 adverse events were 48% in MPT patients and 25% in MP patients (p=0.0002).

Introduction of enoxaparin prophylaxis reduced rate of thromboembolism from 20% to 3% (p=0.005). CONCLUSION: Oral MPT is an effective first-line treatment for elderly patients with multiple myeloma. Anticoagulant prophylaxis reduces frequency of thrombosis. Longer follow-up is needed to assess effect on overall survival.

56 [5]. Palumbo, A., S. Bringhen, et al. (2014). "Bortezomib-melphalan-prednisone-thalidomide followed by maintenance with bortezomib-thalidomide compared with bortezomib-melphalan-prednisone for initial treatment of multiple myeloma: updated follow-up and improved survival." J Clin Oncol **32**(7): 634-640.

PURPOSE: Bortezomib-melphalan-prednisone (VMP) has improved overall survival in multiple myeloma. This randomized trial compared VMP plus thalidomide (VMPT) induction followed by bortezomib-thalidomide maintenance (VMPT-VT) with VMP in patients with newly diagnosed multiple myeloma. PATIENTS AND METHODS: We randomly assigned 511 patients who were not eligible for transplantation to receive VMPT-VT (nine 5-week cycles of VMPT followed by 2 years of VT maintenance) or VMP (nine 5-week cycles without maintenance). RESULTS: In the initial analysis with a median follow-up of 23 months, VMPT-VT improved complete response rate from 24% to 38% and 3-year progression-free-survival (PFS) from 41% to 56% compared with VMP. In this analysis, median follow-up was 54 months. The median PFS was significantly longer with VMPT-VT (35.3 months) than with VMP (24.8 months; hazard ratio [HR], 0.58; P < .001). The time to next therapy was 46.6 months in the VMPT-VT group and 27.8 months in the VMP group (HR, 0.52; P < .001). The 5-year overall survival (OS) was greater with VMPT-VT (61%) than with VMP (51%; HR, 0.70; P = .01). Survival from relapse was identical in both groups (HR, 0.92; P = .63). In the VMPT-VT group, the most frequent grade 3 to 4 adverse events included neutropenia (38%), thrombocytopenia (22%), peripheral neuropathy (11%), and cardiologic events (11%). All of these, except for thrombocytopenia, were significantly more frequent in the VMPT-VT patients. CONCLUSION: Bortezomib and thalidomide significantly improved OS in multiple myeloma patients not eligible for transplantation.

57 [139]. Palumbo, A., S. Bringhen, et al. (2008). "Oral melphalan, prednisone, and thalidomide in elderly patients with multiple myeloma: updated results of a randomized controlled trial." Blood **112**(8): 3107-3114.

The initial analysis of the oral combination melphalan, prednisone, and thalidomide (MPT) in newly diagnosed patients with myeloma showed significantly higher response rate and longer progression-free survival (PFS)

than did the standard melphalan and prednisone (MP) combination and suggested a survival advantage. In this updated analysis, efficacy and safety end points were updated. Patients were randomly assigned to receive oral MPT or MP alone. Updated analysis was by intention to treat and included PFS, overall survival (OS), and survival after progression. After a median follow-up of 38.1 months, the median PFS was 21.8 months for MPT and 14.5 months for MP (P = .004). The median OS was 45.0 months for MPT and 47.6 months for MP (P = .79). In different patient subgroups, MPT improved PFS irrespective of age, serum concentrations of beta(2)-microglobulin, or high International Staging System. Thalidomide or bortezomib administration as salvage regimens significantly improved survival after progression in the MP group (P = .002) but not in the MPT group (P = .34). These data confirm activity of MPT for PFS but failed to show any survival advantage. New agents in the management of relapsed disease could explain this finding. The study is registered at www.clinicaltrials.gov as #NCT00232934.

58 [94]. Palumbo, A., S. Bringhen, et al. (2010). "Bortezomib-melphalan-prednisone-thalidomide followed by maintenance with bortezomib-thalidomide compared with bortezomib-melphalan-prednisone for initial treatment of multiple myeloma: a randomized controlled trial." J Clin Oncol **28**(34): 5101-5109.

PURPOSE: The combination of bortezomib-melphalan-prednisone (VMP) is a new standard of care for newly diagnosed multiple myeloma. This phase III study examined the efficacy of the four-drug combination of bortezomib-melphalan-prednisone-thalidomide (VMPT) followed by maintenance with bortezomib-thalidomide (VMPT-VT) compared with VMP treatment alone in untreated multiple myeloma patients who are ineligible for autologous stem-cell transplantation. PATIENTS AND METHODS: A total of 511 patients were randomly assigned to receive nine cycles of VMPT followed by continuous VT as maintenance, or nine cycles of VMP at the same doses with no additional therapy. The primary end point was progression-free survival. RESULTS: The 3-year estimates of progression-free survival were 56% in patients receiving VMPT-VT and 41% in those receiving VMP (hazard ratio [HR], 0.67; 95% CI, 0.50 to 0.90; P = .008). At 3 years, the cumulative proportions of patients who did not go on to the next therapy were 72% with VMPT-VT and 60% with VMP (HR, 0.58; 95% CI, 0.50 to 0.90; P = .007). Complete response rates were 38% in the VMPT-VT group and 24% in the VMP group (P < .001). The 3-year overall survival was 89% with VMPT-VT and 87% with VMP (HR, 0.92; 95% CI, 0.53 to 1.60; P = .77).

Grade 3 to 4 neutropenia (38% v 28%; P = .02), cardiologic events (10% v 5%; P = .04), and thromboembolic events (5% v 2%; P = .08) were more frequent among patients assigned to the VMPT-VT group than among those assigned to the VMP group; treatment-related deaths were 4% with VMPT-VT and 3% with VMP. CONCLUSION: VMPT followed by VT as maintenance was superior to VMP alone in patients with multiple myeloma who are ineligible for autologous stem-cell transplantation.

59 [2]. Palumbo, A., F. Cavallo, et al. (2014). "Autologous transplantation and maintenance therapy in multiple myeloma." N Engl J Med **371**(10): 895-905.

BACKGROUND: This open-label, randomized, phase 3 study compared melphalan at a dose of 200 mg per square meter of body-surface area plus autologous stem-cell transplantation with melphalan-prednisone-lenalidomide (MPR) and compared lenalidomide maintenance therapy with no maintenance therapy in patients with newly diagnosed multiple myeloma. METHODS: We randomly assigned 273 patients 65 years of age or younger to high-dose melphalan plus stem-cell transplantation or MPR consolidation therapy after induction, and 251 patients to lenalidomide maintenance therapy or no maintenance therapy. The primary end point was progression-free survival. RESULTS: The median follow-up period was 51.2 months. Both progression-free and overall survival were significantly longer with high-dose melphalan plus stem-cell transplantation than with MPR (median progression-free survival, 43.0 months vs. 22.4 months; hazard ratio for progression or death, 0.44; 95% confidence interval [CI], 0.32 to 0.61; P<0.001; and 4-year overall survival, 81.6% vs. 65.3%; hazard ratio for death, 0.55; 95% CI, 0.32 to 0.93; P=0.02). Median progression-free survival was significantly longer with lenalidomide maintenance than with no maintenance (41.9 months vs. 21.6 months; hazard ratio for progression or death, 0.47; 95% CI, 0.33 to 0.65; P<0.001), but 3-year overall survival was not significantly prolonged (88.0% vs. 79.2%; hazard ratio for death, 0.64; 95% CI, 0.36 to 1.15; P=0.14). Grade 3 or 4 neutropenia was significantly more frequent with high-dose melphalan than with MPR (94.3% vs. 51.5%), as were gastrointestinal adverse events (18.4% vs. 0%) and infections (16.3% vs. 0.8%); neutropenia and dermatologic toxic effects were more frequent with lenalidomide maintenance than with no maintenance (23.3% vs. 0% and 4.3% vs. 0%, respectively). CONCLUSIONS: Consolidation therapy with high-dose melphalan plus stem-cell transplantation, as compared with MPR, significantly prolonged progression-free and overall survival among patients with multiple myeloma who were 65 years of age or younger.

Lenalidomide maintenance, as compared with no maintenance, significantly prolonged progression-free survival. (Funded by Celgene; ClinicalTrials.gov number, NCT00551928.).

60 [87]. Palumbo, A., M. Cavo, et al. (2011). "Aspirin, warfarin, or enoxaparin thromboprophylaxis in patients with multiple myeloma treated with thalidomide: a phase III, open-label, randomized trial." J Clin Oncol **29**(8): 986-993.

PURPOSE: In patients with myeloma, thalidomide significantly improves outcomes but increases the risk of thromboembolic events. In this randomized, open-label, multicenter trial, we compared aspirin (ASA) or fixed low-dose warfarin (WAR) versus low molecular weight heparin (LMWH) for preventing thromboembolism in patients with myeloma treated with thalidomide-based regimens. PATIENTS AND METHODS: A total of 667 patients with previously untreated myeloma who received thalidomide-containing regimens and had no clinical indication or contraindication for a specific antiplatelet or anticoagulant therapy were randomly assigned to receive ASA (100 mg/d), WAR (1.25 mg/d), or LMWH (enoxaparin 40 mg/d). A composite primary end point included serious thromboembolic events, acute cardiovascular events, or sudden deaths during the first 6 months of treatment. RESULTS: Of 659 analyzed patients, 43 (6.5%) had serious thromboembolic events, acute cardiovascular events, or sudden death during the first 6 months (6.4% in the ASA group, 8.2% in the WAR group, and 5.0% in the LMWH group). Compared with LMWH, the absolute differences were +1.3% (95% CI, -3.0% to 5.7%; P = .544) in the ASA group and +3.2% (95% CI, -1.5% to 7.8%; P = .183) in the WAR group. The risk
of thromboembolism was 1.38 times higher in patients treated with thalidomide without bortezomib. Three major (0.5%) and 10 minor (1.5%) bleeding episodes were recorded. CONCLUSION: In patients with myeloma treated with thalidomide-based regimens, ASA and WAR showed similar efficacy in reducing serious thromboembolic events, acute cardiovascular events, and sudden deaths compared with LMWH, except in elderly patients where WAR showed less efficacy than LMWH.

61 [54]. Palumbo, A., R. Hajek, et al. (2012). "Continuous lenalidomide treatment for newly diagnosed multiple myeloma." N Engl J Med **366**(19): 1759-1769.

BACKGROUND: Lenalidomide has tumoricidal and immunomodulatory activity against multiple myeloma. This double-blind, multicenter, randomized study compared melphalan-prednisone-lenalidomide

induction followed by lenalidomide maintenance (MPR-R) with melphalan-prednisone-lenalidomide (MPR) or melphalan-prednisone (MP) followed by placebo in patients 65 years of age or older with newly diagnosed multiple myeloma. METHODS: We randomly assigned patients who were ineligible for transplantation to receive MPR-R (nine 4-week cycles of MPR followed by lenalidomide maintenance therapy until a relapse or disease progression occurred [152 patients]) or to receive MPR (153 patients) or MP (154 patients) without maintenance therapy. The primary end point was progression-free survival. RESULTS: The median follow-up period was 30 months. The median progression-free survival was significantly longer with MPR-R (31 months) than with MPR (14 months; hazard ratio, 0.49; P<0.001) or MP (13 months; hazard ratio, 0.40; P<0.001). Response rates were superior with MPR-R and MPR (77% and 68%, respectively, vs. 50% with MP; P<0.001 and P=0.002, respectively, for the comparison with MP). The progression-free survival benefit associated with MPR-R was noted in patients 65 to 75 years of age but not in those older than 75 years of age (P=0.001 for treatment-by-age interaction). After induction therapy, a landmark analysis showed a 66% reduction in the rate of progression with MPR-R (hazard ratio for the comparison with MPR, 0.34; P<0.001) that was age-independent. During induction therapy, the most frequent adverse events were hematologic; grade 4 neutropenia was reported in 35%, 32%, and 8% of the patients in the MPR-R, MPR, and MP groups, respectively. The 3-year rate of second primary tumors was 7% with MPR-R, 7% with MPR, and 3% with MP. CONCLUSIONS: MPR-R significantly prolonged progression-free survival in patients with newly diagnosed multiple myeloma who were ineligible for transplantation, with the greatest benefit observed in patients 65 to 75 years of age. (Funded by Celgene; MM-015 ClinicalTrials.gov number, NCT00405756.).

62 [167]. Pineda-Roman, M., V. Bolejack, et al. (2007). "Complete response in myeloma extends survival without, but not with history of prior monoclonal gammopathy of undetermined significance or smouldering disease." Br J Haematol **136**(3): 393-399.

Complete response (CR) is still considered an important surrogate marker for outcome in multiple myeloma (MM). Long-term survival after transplantation, however, has been observed in a substantial proportion of patients who never achieved CR. The tandem transplant trial, Total Therapy 2, enrolled 668 patients, who were randomised up-front to thalidomide (THAL) or no THAL; 56 patients were identified as having had, for at least 6 months prior to initiation of therapy, monoclonal gammopathy of undetermined significance (MGUS, n = 21), smouldering

MM (SMM, n = 22) or solitary plasmacytoma of bone (SPC, n = 13). The clinical characteristics and outcomes of patients with such 'evolved' MM (E-MM) and of those with 'unknown' prior history (U-MM) were compared. Fewer patients with MGUS/SMM-E-MM had anaemia or renal failure; CR was lower (22% vs. 48%) but 4-year estimates of event-free survival (54% vs. 56% with U-MM) and overall survival (65% vs. 70% with U-MM) were similar to those with SPC-E-MM or U-MM. In the latter group, achieving CR was associated with prolonged survival. In comparison with U-MM, E-MM evolved from MGUS/SMM was associated with lower CR rate without adversely affecting survival. In contrast, CR was an independent favourable feature for survival in U-MM.

63 [182]. Rajkumar, S. V., E. Blood, et al. (2006). "Phase III clinical trial of thalidomide plus dexamethasone compared with dexamethasone alone in newly diagnosed multiple myeloma: a clinical trial coordinated by the Eastern Cooperative Oncology Group." J Clin Oncol **24**(3): 431-436.

PURPOSE: To determine if thalidomide plus dexamethasone yields superior response rates compared with dexamethasone alone as induction therapy for newly diagnosed multiple myeloma. PATIENTS AND METHODS: Patients were randomly assigned to receive thalidomide plus dexamethasone or dexamethasone alone. Patients in arm A received thalidomide 200 mg orally for 4 weeks; dexamethasone was administered at a dose of 40 mg orally on days 1 to 4, 9 to 12, and 17 to 20. Cycles were repeated every 4 weeks. Patients in arm B received dexamethasone alone at the same schedule as in arm A. RESULTS: Two hundred seven patients were enrolled: 103 were randomly assigned to thalidomide plus dexamethasone and 104 were randomly assigned to dexamethasone alone; eight patients were ineligible. The response rate with thalidomide plus dexamethasone was significantly higher than with dexamethasone alone (63% v 41%, respectively; P = .0017). The response rate allowing for use of serum monoclonal protein levels when a measurable urine monoclonal protein was unavailable at follow-up was 72% v 50%, respectively. The incidence rates of grade 3 or higher deep vein thrombosis (DVT), rash, bradycardia, neuropathy, and any grade 4 to 5 toxicity in the first 4 months were significantly higher with thalidomide plus dexamethasone compared with dexamethasone alone (45% v 21%, respectively; P < .001). DVT was more frequent in arm A than in arm B (17% v 3%); grade 3 or higher peripheral neuropathy was also more frequent (7% v 4%, respectively). CONCLUSION: Thalidomide plus dexamethasone demonstrates significantly superior response rates in newly diagnosed

myeloma compared with dexamethasone alone. However, this must be balanced against the greater toxicity seen with the combination.

64 [118]. Rajkumar, S. V., S. Jacobus, et al. (2010). "Lenalidomide plus high-dose dexamethasone versus lenalidomide plus low-dose dexamethasone as initial therapy for newly diagnosed multiple myeloma: an open-label randomised controlled trial." Lancet Oncol **11**(1): 29-37.

BACKGROUND: High-dose dexamethasone is a mainstay of therapy for multiple myeloma. We studied whether low-dose dexamethasone in combination with lenalidomide is non-inferior to and has lower toxicity than high-dose dexamethasone plus lenalidomide. METHODS: Patients with untreated symptomatic myeloma were randomly assigned in this open-label non-inferiority trial to lenalidomide 25 mg on days 1-21 plus dexamethasone 40 mg on days 1-4, 9-12, and 17-20 of a 28-day cycle (high dose), or lenalidomide given on the same schedule with dexamethasone 40 mg on days 1, 8, 15, and 22 of a 28-day cycle (low dose). After four cycles, patients could discontinue therapy to pursue stem-cell transplantation or continue treatment until disease progression. The primary endpoint was response rate after four cycles assessed with European Group for Blood and Bone Marrow Transplant criteria. The non-inferiority margin was an absolute difference of 15% in response rate. Analysis was by modified intention to treat. This trial is registered with ClinicalTrials.gov, number NCT00098475. FINDINGS: 445 patients were randomly assigned: 223 to high-dose and 222 to low-dose regimens. 169 (79%) of 214 patients receiving high-dose therapy and 142 (68%) of 205 patients on low-dose therapy had complete or partial response within four cycles (odds ratio 1.75, 80% CI 1.30-2.32; p=0.008). However, at the second interim analysis at 1 year, overall survival was 96% (95% CI 94-99) in the low-dose dexamethasone group compared with 87% (82-92) in the high-dose group (p=0.0002). As a result, the trial was stopped and patients on high-dose therapy were crossed over to low-dose therapy. 117 patients (52%) on the high-dose regimen had grade three or worse toxic effects in the first 4 months, compared with 76 (35%) of the 220 on the low-dose regimen for whom toxicity data were available (p=0.0001), 12 of 222 on high dose and one of 220 on low-dose dexamethasone died in the first 4 months (p=0.003). The three most common grade three or higher toxicities were deep-vein thrombosis, 57 (26%) of 223 versus 27 (12%) of 220 (p=0.0003); infections including pneumonia, 35 (16%) of 223 versus 20 (9%) of 220 (p=0.04), and fatigue 33 (15%) of 223 versus 20 (9%) of 220 (p=0.08), respectively. INTERPRETATION: Lenalidomide plus low-dose dexamethasone is associated with better short-term overall survival and

with lower toxicity than lenalidomide plus high-dose dexamethasone in patients with newly diagnosed myeloma. FUNDING: National Cancer Institute, Rockville, MD, USA.

65 [144]. Rajkumar, S. V., L. Rosinol, et al. (2008). "Multicenter, randomized, double-blind, placebo-controlled study of thalidomide plus dexamethasone compared with dexamethasone as initial therapy for newly diagnosed multiple myeloma." J Clin Oncol **26**(13): 2171-2177.

PURPOSE: The long-term impact of thalidomide plus dexamethasone (thal/dex) as primary therapy for newly diagnosed multiple myeloma (MM) is unknown. The goal of this study was to compare thalidomide plus dexamethasone versus placebo plus dexamethasone (placebo/dex)as primary therapy for newly diagnosed MM. PATIENTS AND METHODS: In this double-blind, placebo-controlled trial, patients with untreated symptomatic MM were randomized to thal/dex (arm A) or to placebo plus dexamethasone (dex) (arm B). Patients in arm A received oral thalidomide 50 mg daily, escalated to 100 mg on day 15, and to 200 mg from day 1 of cycle 2 (28-day cycles). Oral dex 40 mg was administered on days 1 through 4, 9 through 12, and 17 through 20 during cycles 1 through 4 and on days 1 through 4 only from cycle 5 onwards. Patients in arm B received placebo and dex, administered as in arm A. The primary end point of the study was time to progression. This study is registered at http://ClinicalTrials.gov (NCT00057564). RESULTS: A total of 470 patients were enrolled (235 randomly assigned to thal/dex and 235 to placebo/dex). The overall response rate was significantly higher with thal/dex compared with placebo/dex (63% v 46%), P < .001. Time to progression (TTP) was significantly longer with thal/dex compared with placebo/dex (median, 22.6 v 6.5 months, P < .001). Grade 4 adverse events were more frequent with thal/dex than with placebo/dex (30.3% v 22.8%). CONCLUSION: Thal/dex results in significantly higher response rates and significantly prolongs TTP compared with dexamethasone alone in patients with newly diagnosed MM.

66 [16]. Rawstron, A. C., J. A. Child, et al. (2013). "Minimal residual disease assessed by multiparameter flow cytometry in multiple myeloma: impact on outcome in the Medical Research Council Myeloma IX Study." J Clin Oncol **31**(20): 2540-2547.

PURPOSE: To investigate the prognostic value of minimal residual disease (MRD) assessment in patients with multiple myeloma treated in the MRC (Medical Research Council) Myeloma IX trial. PATIENTS AND METHODS:

Multiparameter flow cytometry (MFC) was used to assess MRD after induction therapy (n = 378) and at day 100 after autologous stem-cell transplantation (ASCT; n = 397) in intensive-pathway patients and at the end of induction therapy in non-intensive-pathway patients (n = 245). RESULTS: In intensive-pathway patients, absence of MRD at day 100 after ASCT was highly predictive of a favorable outcome (PFS, P < .001; OS, P = .0183). This outcome advantage was demonstrable in patients with favorable and adverse cytogenetics (PFS, P = .014 and P < .001, respectively) and in patients achieving immunofixation-negative complete response (CR; PFS, P = .0068). The effect of maintenance thalidomide was assessed, with the shortest PFS demonstrable in those MRD-positive patients who did not receive maintenance and longest in those who were MRD negative and did receive thalidomide (P < .001). Further analysis demonstrated that 28% of MRD-positive patients who received maintenance thalidomide became MRD negative. MRD assessment after induction therapy in the non-intensive-pathway patients did not seem to be predictive of outcome (PFS, P = .1). CONCLUSION: MRD assessment by MFC was predictive of overall outcome in patients with myeloma undergoing ASCT. This predictive value was seen in patients achieving conventional CR as well as patients with favorable and adverse cytogenetics. The effects of maintenance strategies can also be evaluated, and our data suggest that maintenance thalidomide can eradicate MRD in some patients.

67 [172]. Richardson, P. G., E. Blood, et al. (2006). "A randomized phase 2 study of lenalidomide therapy for patients with relapsed or relapsed and refractory multiple myeloma." Blood **108**(10): 3458-3464.

This multicenter, open-label, randomized phase 2 study evaluated 2 dose regimens of lenalidomide for relapsed, refractory myeloma. Seventy patients were randomized to receive either 30 mg once-daily or 15 mg twice-daily oral lenalidomide for 21 days of every 28-day cycle. Patients with progressive or stable disease after 2 cycles received dexamethasone. Analysis of the first 70 patients showed increased grade 3/4 myelo-suppression in patients receiving 15 mg twice daily (41% versus 13%, P = .03). An additional 32 patients received 30 mg once daily. Responses were evaluated according to European Group for Blood and Marrow Transplantation (EBMT) criteria. Overall response rate (complete, partial, or minor) to lenalidomide alone was 25% (24% for once-daily and 29% for twice-daily lenalidomide). Median overall survival in 30-mg once-daily and twice-daily groups was 28 and 27 months, respectively. Median progression-free survival was 7.7 months on once-daily versus 3.9 months

on twice-daily lenalidomide (P = .2). Dexamethasone was added in 68 patients and 29% responded. Time to first occurrence of clinically significant grade 3/4 myelosuppression was shorter in the twice-daily group (1.8 vs 5.5 months, P = .05). Significant peripheral neuropathy and deep vein thrombosis each occurred in only 3%. Lenalidomide is active and well tolerated in relapsed, refractory myeloma, with the 30-mg once-daily regimen providing the basis for future studies as monotherapy and with dexamethasone.

68 [6]. Richardson, P. G., D. S. Siegel, et al. (2014). "Pomalidomide alone or in combination with low-dose dexamethasone in relapsed and refractory multiple myeloma: a randomized phase 2 study." Blood **123**(12): 1826-1832.

This multicenter, open-label, randomized phase 2 study assessed the efficacy and safety of pomalidomide (POM) with/without low-dose dexamethasone (LoDEX) in patients with relapsed/refractory multiple myeloma (RRMM). Patients who had received >/=2 prior therapies (including lenalidomide [LEN] and bortezomib [BORT]) and had progressed within 60 days of their last therapy were randomized to POM (4 mg/day on days 1-21 of each 28-day cycle) with/without LoDEX (40 mg/week). The primary end point was progression-free survival (PFS). In total, 221 patients (median 5 prior therapies, range 1-13) received POM+LoDEX (n = 113) or
POM (n = 108). With a median follow-up of 14.2 months, median PFS was 4.2 and 2.7 months (hazard ratio = 0.68, P = .003), overall response rates (ORRs) were 33% and 18% (P = .013), median response duration was 8.3 and 10.7 months, and median overall survival (OS) was 16.5 and 13.6 months, respectively. Refractoriness to LEN, or resistance to both LEN and BORT, did not affect outcomes with POM+LoDEX (median PFS 3.8 months for both; ORRs 30% and 31%; and median OS 16 and 13.4 months). Grade 3-4 neutropenia occurred in 41% (POM+LoDEX) and 48% (POM); no grade 3-4 peripheral neuropathy was reported. POM+LoDEX was effective and generally well tolerated and provides an important new treatment option for RRMM patients who have received multiple prior therapies. This trial was registered at www.clinicaltrials.gov as #NCT00833833.

69 [47]. Rosinol, L., A. Oriol, et al. (2012). "Superiority of bortezomib, thalidomide, and dexamethasone (VTD) as induction pretransplantation therapy in multiple myeloma: a randomized phase 3 PETHEMA/GEM study." Blood **120**(8):
1589-1596.

The Spanish Myeloma Group conducted a trial to compare bortezomib/thalidomide/dexamethasone (VTD) versus thalidomide/dexamethasone (TD) versus vincristine, BCNU, melphalan, cyclophosphamide, prednisone/vincristine, BCNU, doxorubicin, dexamethasone/bortezomib (VBMCP/VBAD/B) in patients aged 65 years or younger with multiple myeloma. The primary endpoint was complete response (CR) rate postinduction and post-autologous stem cell transplantation (ASCT). Three hundred eighty-six patients were allocated to VTD (130), TD (127), or VBMCP/VBAD/B (129). The CR rate was significantly higher with VTD than with TD (35% vs 14%, P = .001) or with VBMCP/VBAD/B (35% vs 21%, P = .01). The median progression-free survival (PFS) was significantly longer with VTD (56.2 vs 28.2 vs 35.5 months, P = .01). In an intention-to-treat analysis, the post-ASCT CR rate was higher with VTD than with TD (46% vs 24%, P = .004) or with VBMCP/VBAD/B (46% vs 38%, P = .1). Patients with high-risk cytogenetics had a shorter PFS and overall survival in the overall series and in all treatment groups. In conclusion, VTD resulted in a higher pre- and posttransplantation CR rate and in a significantly longer PFS although it was not able to overcome the poor prognosis of high-risk cytogenetics. Our results support the use of VTD as a highly effective induction regimen prior to ASCT. The study was registered with http://www.clinicaltrials.gov (NCT00461747) and Eudra CT (no. 2005-001110-41).

70 [81]. Sacchi, S., R. Marcheselli, et al. (2011). "A randomized trial with melphalan and prednisone versus melphalan and prednisone plus thalidomide in newly diagnosed multiple myeloma patients not eligible for autologous stem cell transplant." Leuk Lymphoma **52**(10): 1942-1948.

Several trials comparing the efficacy of standard melphalan and prednisone (MP) therapy with MP plus thalidomide (MPT) in elderly patients with multiple myeloma (MM) have been reported, with inconsistent results. The primary goal of our study was to evaluate the efficacy and toxicity of MP versus MPT in newly diagnosed patients with MM who were transplant-ineligible or over age 65. A total of 135 patients were enrolled. Either minimal response or better or partial response or better were more frequent with MPT treatment (p = 0.001). After a median follow-up of 30 months, median progression-free survival (PFS) and overall survival (OS) were 33 and 52 months for MPT versus 22 and 32 months for MP, respectively. The comparison showed a significant advantage for MPT versus MP in PFS (p = 0.02) and only a trend for OS (p = 0.07). Severe adverse events were observed more frequently with MPT. In conclusion, our results show an improved activity of MPT at a cost of increased

toxicity. We believe that MPT can be considered one of the new standard of care for elderly or transplant-ineligible patients with MM.

71 [10]. San Miguel, J., K. Weisel, et al. (2013). "Pomalidomide plus low-dose dexamethasone versus high-dose dexamethasone alone for patients with relapsed and refractory multiple myeloma (MM-003): a randomised, open-label, phase 3 trial." Lancet Oncol **14**(11): 1055-1066.

BACKGROUND: Few effective treatments exist for patients with refractory or relapsed and refractory multiple myeloma not responding to treatment with bortezomib and lenalidomide. Pomalidomide alone has shown limited efficacy in patients with relapsed multiple myeloma, but synergistic effects have been noted when combined with dexamethasone. We compared
the efficacy and safety of pomalidomide plus low-dose dexamethasone with high-dose dexamethasone alone in these patients. METHODS: This multicentre, open-label, randomised phase 3 trial was undertaken in Australia, Canada, Europe, Russia, and the USA. Patients were eligible if they had been diagnosed with refractory or relapsed and refractory multiple myeloma, and had failed at least two previous treatments of bortezomib and lenalidomide. They were assigned in a 2:1 ratio with a validated interactive voice and internet response system to either 28 day cycles of pomalidomide (4 mg/day on days 1-21, orally) plus low-dose dexamethasone (40 mg/day on days 1, 8, 15, and 22, orally) or high-dose dexamethasone (40 mg/day on days 1-4, 9-12, and 17-20, orally) until disease progression or unacceptable toxicity. Stratification factors were age (</=75 years vs >75 years), disease population (refractory vs relapsed and refractory vs bortezomib intolerant), and number of previous treatments (two vs more than two). The primary endpoint was progression-free survival (PFS). Analysis was by intention to treat. This trial is registered with ClinicalTrials.gov, number NCT01311687, and with EudraCT, number 2010-019820-30. FINDINGS: The accrual for the study has been completed and the analyses are presented. 302 patients were randomly assigned to receive pomalidomide plus low-dose dexamethasone and 153 high-dose dexamethasone. After a median follow-up of 10.0 months (IQR 7.2-13.2), median PFS with pomalidomide plus low-dose dexamethasone was 4.0 months (95% CI 3.6-4.7) versus 1.9 months (1.9-2.2) with high-dose dexamethasone (hazard ratio 0.48 [95% CI 0.39-0.60]; p<0.0001). The most common grade 3-4 haematological adverse events in the pomalidomide plus low-dose dexamethasone and high-dose dexamethasone groups were neutropenia (143 [48%] of 300 vs 24 [16%] of 150, respectively), anaemia (99 [33%] vs 55 [37%], respectively), and thrombocytopenia (67

[22%] vs 39 [26%], respectively). Grade 3-4 non-haematological adverse events in the pomalidomide plus low-dose dexamethasone and high-dose dexamethasone groups included pneumonia (38 [13%] vs 12 [8%], respectively), bone pain (21 [7%] vs seven [5%], respectively), and fatigue (16 [5%] vs nine [6%], respectively). There were 11 (4%) treatment-related adverse events leading to death in the pomalidomide plus low-dose dexamethasone group and seven (5%) in the high-dose dexamethasone group. INTERPRETATION: Pomalidomide plus low-dose dexamethasone, an oral regimen, could be considered a new treatment option in patients with refractory or relapsed and refractory multiple myeloma. FUNDING: Celgene Corporation.

72 [169]. Sharma, R. A., W. P. Steward, et al. (2006). "Toxicity profile of the immunomodulatory thalidomide analogue, lenalidomide: phase I clinical trial of three dosing schedules in patients with solid malignancies." Eur J Cancer **42**(14): 2318-2325.

Thalidomide is an anti-angiogenic agent currently used to treat patients with malignant cachexia or multiple myeloma. Lenalidomide (CC-5013) is an immunomodulatory thalidomide analogue licensed in the United States of America (USA) for the treatment of a subtype of myelodysplastic syndrome. This two-centre, open-label phase I study evaluated dose-limiting toxicities in 55 patients with malignant solid tumours refractory to standard chemotherapies. Lenalidomide capsules were consumed once daily for 12 weeks according to one of the following three schedules: (I) 25 mg daily for the first 7 d, the daily dose increased by 25 mg each week up to a maximum daily dose of 150 mg; (II) 25mg daily for 21 d followed by a 7-d rest period, the 4-week cycle repeated for 3 cycles; (III) 10 mg daily continuously. Twenty-six patients completed the study period. Two patients experienced a grade 3 hypersensitivity rash. Four patients in cohort I and 4 patients in cohort II suffered grade 3 or 4 neutropaenia. In 2 patients with predisposing medical factors, grade 3 cardiac dysrhythmia was recorded. Grade 1 neurotoxicity was detected in 6 patients. One complete and two partial radiological responses were measured by computed tomography scanning; 8 patients had stable disease after 12 weeks of treatment. Fifteen patients remained on treatment as named patients; 1 with metastatic melanoma remains in clinical remission 3.5 years from trial entry. This study indicates the tolerability and potential clinical efficacy of lenalidomide in patients with advanced solid tumours who have previously received multi-modality treatment. Depending on the extent of myelosuppressive pre-treatment, dose schedules (II) or (III) are advocated for large-scale trials of long-term administration.

73 [46]. Sonneveld, P., I. G. Schmidt-Wolf, et al. (2012). "Bortezomib induction and maintenance treatment in patients with newly diagnosed multiple myeloma: results of the randomized phase III HOVON-65/ GMMG-HD4 trial." J Clin Oncol **30**(24): 2946-2955.

PURPOSE: We investigated whether bortezomib during induction and maintenance improves survival in newly diagnosed multiple myeloma (MM). PATIENTS AND METHODS: In all, 827 eligible patients with newly diagnosed symptomatic MM were randomly assigned to receive induction therapy with vincristine, doxorubicin, and dexamethasone (VAD) or bortezomib, doxorubicin, and dexamethasone (PAD) followed by high-dose melphalan and autologous stem-cell transplantation. Maintenance consisted of thalidomide 50 mg (VAD) once per day or bortezomib 1.3 mg/m(2) (PAD) once every 2 weeks for 2 years. The primary analysis was progression-free survival (PFS) adjusted for International Staging System (ISS) stage. RESULTS: Complete response (CR), including near CR, was superior after PAD induction (15% v 31%; P < .001) and bortezomib maintenance (34% v 49%; P < .001). After a median follow-up of 41 months, PFS was superior in the PAD arm (median of 28 months v 35

months; hazard ratio [HR], 0.75; 95% CI, 0.62 to 0.90; P = .002). In multivariate analysis, overall survival (OS) was better in the PAD arm (HR, 0.77; 95% CI, 0.60 to 1.00; P = .049). In high-risk patients presenting with increased creatinine more than 2 mg/dL, bortezomib significantly improved PFS from a median of 13 months to 30 months (HR, 0.45; 95% CI, 0.26 to 0.78; P = .004) and OS from a median of 21 months to 54 months (HR, 0.33; 95% CI, 0.16 to 0.65; P < .001). A benefit was also observed in patients with deletion 17p13

(median PFS, 12 v 22 months; HR, 0.47; 95% CI, 0.26 to 0.86; P = .01; median OS, 24 months v not reached at 54 months; HR, 0.36; 95% CI, 0.18 to 0.74; P = .003). CONCLUSION: Bortezomib during induction and maintenance improves CR and achieves superior PFS and OS.

74 [129]. Spencer, A., H. M. Prince, et al. (2009). "Consolidation therapy with low-dose thalidomide and prednisolone prolongs the survival of multiple myeloma patients undergoing a single autologous stem-cell transplantation procedure." J Clin Oncol **27**(11): 1788-1793.

PURPOSE: Thalidomide is effective in the treatment of newly diagnosed and relapsed/refractory multiple myeloma (MM). However, the role of thalidomide in the post-autologous stem cell transplantation (ASCT) context remains unclear. This study assessed whether the addition of

thalidomide consolidation following ASCT would improve the durability of responses achieved and overall survival. PATIENTS AND METHODS: Between January 2002 and March 2005, 269 patients with newly diagnosed MM who achieved disease stabilization or better with conventional induction chemotherapy received a single high-dose melphalan conditioned ASCT. Post-ASCT, 129 patients were randomly assigned to receive indefinite prednisolone maintenance therapy (control group) and 114 to receive the same in addition to 12 months of thalidomide consolidation (thalidomide group). The primary study end points were progression-free survival (PFS) and overall survival (OS). The secondary end point was tolerability. RESULTS: After a median follow-up of 3 years, the postrandomization 3-year PFS rates were 42% and 23% (P < .001; hazard ratio [HR], 0.5; 95% CI, 0.35 to 0.71) and the OS rates were 86% and 75% (P = .004; HR, 0.41; 95% CI, 0.22 to 0.76) in the thalidomide and control groups, respectively. There was no difference in survival between groups 12 months after disease progression (79% v 77%; P = .237). Neurological toxicities were more common in the thalidomide arm but there were no differences between arms for thromboembolic events. CONCLUSION: Consolidation therapy with 12 months of thalidomide combined with prednisolone prolongs survival when used after a single high-dose therapy supported ASCT in patients with newly diagnosed MM. Furthermore, thalidomide consolidation therapy did not adversely impact on survival in the subsequent salvage setting.

75 [190]. Stewart, A. K., C. I. Chen, et al. (2004). "Results of a multicenter randomized phase II trial of thalidomide and prednisone maintenance therapy for multiple myeloma after autologous stem cell transplant." Clin Cancer Res **10**(24): 8170-8176.

We report a multicenter, randomized phase II trial conducted to assess the tolerability of combined thalidomide and prednisone maintenance in multiple myeloma. Eligibility required administration of melphalan (200 mg/m2) with blood stem cell support within 1 year of treatment onset and initiation of maintenance within 60 to 100 days after stem cell infusion. All patients received 50 mg of prednisone by mouth on alternate days and thalidomide at a starting dose of either 200 or 400 mg daily by mouth. The primary end point was the incidence of dropout or dose reduction due to treatment toxicity within 6 months. Sixty-seven patients were enrolled. Median follow-up is 36.8 months. The primary end point was reached by 31% of patients on the 200 mg of thalidomide arm and 64% of patients on the 400 mg of thalidomide arm. Allowing for dose reduction, 76% of patients assigned to the 200 mg of thalidomide arm and 41% of patients

assigned to the 400 mg of thalidomide arm remained on any maintenance therapy 18 months after registration. Eighty-eight percent of all patients dose-reduced thalidomide and 72% of all patients dose-reduced prednisone within 2 years of beginning maintenance. The median progression-free survival post-transplant is 32.3 months, or 42.2 months from diagnosis. Only the 200 mg of thalidomide arm of this trial met our definition of a tolerable maintenance therapy, defined as no dose reductions or discontinuation due to toxicity in at least 65% of patients for a minimum of 6 months, thus establishing a dosing schedule for phase III trials.

76 [26]. Stewart, A. K., S. Trudel, et al. (2013). "A randomized phase 3 trial of thalidomide and prednisone as maintenance therapy after ASCT in patients with MM with a quality-of-life assessment: the National Cancer Institute of Canada Clinicals Trials Group Myeloma 10 Trial." Blood **121**(9): 1517-1523.

We conducted a randomized, controlled trial comparing thalidomide-prednisone as maintenance therapy with observation in 332 patients who had undergone autologous stem cell transplantation with melphalan 200 mg/m2. The primary end point was overall survival (OS); secondary end points were myeloma-specific progression-free survival,progression-free survival, incidence of venous thromboembolism, and health-related quality of life (HRQoL). With a median follow-up of 4.1 years, no differences in OS between thalidomide-prednisone and observation were detected (respective 4-year estimates of 68% vs 60%, respectively; hazard ratio = 0.77; P = .18); thalidomide-prednisone was associated with superior myeloma-specific progression-free survival and progression-free survival (for both outcomes, the 4-year estimates were 32% vs 14%; hazard ratio = 0.56; P < .0001) and more frequent venous thromboembolism (7.3% vs none; P = .0004). Median survival after first
disease recurrence was 27.7 months with thalidomide-prednisone and 34.1 months in the observation group. Nine second malignancies were observed with thalidomide-prednisone versus 6 in the observation group. Those allocated to thalidomide-prednisone reported worse HRQoL with respect to cognitive function, dyspnea, constipation, thirst, leg swelling, numbness, dry mouth, and balance problems. We conclude that maintenance therapy with thalidomide-prednisone after autologous stem cell transplantation improves the duration of disease control, but is associated with worsening of patient-reported HRQoL and no detectable OS benefit.

77 [187]. Talamo, G., E. Angtuaco, et al. (2005). "Avascular necrosis of femoral and/or humeral heads in multiple myeloma: results of a prospective study of patients treated with dexamethasone-based regimens and high-dose chemotherapy." J Clin Oncol **23**(22): 5217-5223.

PURPOSE: To assess the prevalence, time of onset, risk factors, and outcome of avascular necrosis (AVN) of bone in patients with multiple myeloma undergoing antineoplastic therapy. PATIENTS AND METHODS: A total of 553 consecutive assessable patients were enrolled onto a treatment protocol consisting of dexamethasone-containing induction chemotherapy, autologous stem-cell transplantation, consolidation chemotherapy, and maintenance with interferon alfa. Patients were randomly assigned to receive thalidomide (269 patients) or no thalidomide (284 patients) throughout the study period. RESULTS: With a median follow-up of 33 months (range, 5 to 114 months), AVN of the femoral head(s) developed in 49 patients (9%). Median time to onset of AVN was 12 months (range, 2 to 41 months). Three risk factors for AVN were identified by multivariate analysis: cumulative dexamethasone dose (odds ratio [OR], 1.028; 95% CI, 1.012 to 1.044; P = .0006 [per 40 mg dexamethasone]), male sex (OR, 0.390; 95% CI, 0.192 to 0.790; P = .009), and younger age (OR, 0.961; 95% CI, 0.934 to 0.991 per year; P = .0122). Thalidomide-treated patients had a prevalence of AVN similar to that of the control group (8% v 10%, respectively; P = .58). AVN-related pain and limited range of motion of the affected joint were present in only nine and four patients, respectively, and four patients underwent hip replacement because of AVN. Fluorine-18 fluorodeoxyglucose positron emission tomography failed to detect abnormal uptake in the AVN-affected bones. CONCLUSION: AVN is a rare and usually asymptomatic complication during myeloma therapy. Cumulative dexamethasone dose, male sex, and younger age, but not thalidomide, increase the risk of AVN.

78 [50]. Usmani, S. Z., R. Sexton, et al. (2012). "Second malignancies in total therapy 2 and 3 for newly diagnosed multiple myeloma: influence of thalidomide and lenalidomide during maintenance." Blood **120**(8): 1597-1600.

Thalidomide and lenalidomide constitute an important part of effective myeloma therapy. Recent data from the Intergroup Francophone du Myelome, Cancer and Leukemia Group B, and Gruppo Italiano Malattie Ematologiche dell Adulto MM-015 trials suggest that lenalidomide maintenance therapy is associated with a higher incidence of second primary malignancies (SPMs), including both hematologic and solid malignancies. In the present study, we analyzed data from the Total

Therapy 2 (TT2) trial, along with the 2 Total Therapy 3 (TT3) trials. TT2 patients were assigned randomly to either a control group (no thalidomide) or to the experimental group (thalidomide during induction, between transplantations, and during consolidation and maintenance). The 2 TT3 trials used thalidomide and bortezomib during induction, before and in consolidation after tandem melphalan-based transplantation; TT3A applied VTD (bortezomib, thalidomide, dexamethasone) in the first year of maintenance and TD for 2 more years, whereas TT3B used VRD (bortezomib, lenalidomide, dexamethasone) maintenance for 3 years. The cumulative incidence of SPMs did not differ significantly among the TT trial components when measured from enrollment (P = .78) or from initiation of maintenance (P = .82). However, a pairwise comparison of the TT2 arms suggested a lower incidence of hematologic SPMs in the thalidomide maintenance arm (hazard ratio = 0.38; P = .09). These trials are registered at www.clinicaltrials.gov as NCT00573391 (TT2), NCT00081939 (TT3A), and NCT00572169 (TT3B).

79 [85]. Verelst, S. G., F. Termorshuizen, et al. (2011). "Effect of thalidomide with melphalan and prednisone on health-related quality of life (HRQoL) in elderly patients with newly diagnosed multiple myeloma: a prospective analysis in a randomized trial." Ann Hematol **90**(12): 1427-1439.

Thalidomide with melphalan/prednisone (MPT) was defined as standard treatment in elderly patients with multiple myeloma (MM) based on five randomized trials. In one of these trials, HOVON49, a prospective health-related quality-of-life (HRQoL) study was initiated in order to assess the impact of thalidomide on QoL. Patients aged >65 years with newly diagnosed MM were randomized to receive melphalan plus prednisone (MP) or MPT, followed by thalidomide maintenance in the MPT arm. Two hundred eighty-four patients were included in this side study (MP, n=149; MPT n=135). HRQoL was assessed with the EORTC Core QoL Questionnaire (QLQ-C30) and the myeloma-specific module (QLQ-MY24) at baseline and at predetermined intervals during treatment. The QLQ-C30 subscales physical function (P=0.044) and constipation (P<0.001) showed an improvement during induction in favour of the MP arm. During thalidomide maintenance, the scores for the QLQ-MY24 paraesthesia became significantly higher in the MPT arm (P<0.001). The QLQ-C30 subscales pain (P=0.12), insomnia (P=0.068), appetite loss (P=0.074) and the QLQ-MY24
item sick (P=0.086) scored marginally better during thalidomide maintenance. The overall QoL-scale QLQ-C30-HRQoL showed a significant time trend towards more favourable mean values during

protocol treatment without differences between MP and MPT. For the QLQ-C30 subscales emotional function and future perspectives, difference in favour of the MPT arm from the start of treatment was observed (P=0.018 and
P=0.045, respectively) with no significant 'time x arm' interaction, indicating a persistent better patient perspective with MPT treatment. This study shows that the higher frequency of toxicity associated with MPT does not translate into a negative effect on HRQoL and that MPT holds a better patient perspective.

80 [105]. Waage, A., P. Gimsing, et al. (2010). "Melphalan and prednisone plus thalidomide or placebo in elderly patients with multiple myeloma." Blood **116**(9): 1405-1412.

In this double-blind, placebo-controlled study, 363 patients with untreated multiple myeloma were randomized to receive either melphalan-prednisone and thalidomide (MPT) or melphalan-prednisone and placebo (MP). The dose of melphalan was 0.25 mg/kg and prednisone was 100 mg given daily for 4 days every 6 weeks until plateau phase. The dose of thalidomide/placebo was escalated to 400 mg daily until plateau phase and thereafter reduced to 200 mg daily until progression. A total of 357 patients were analyzed. Partial response was 34% and 33%, and very good partial response or better was 23% and 7% in the MPT and MP arms, respectively (P < .001). There was no significant difference in progression-free or overall survival, with median survival being 29 months in the MPT arm and 32 months in the MP arm. Most quality of life outcomes improved equally in both arms, apart from constipation, which was markedly increased in the MPT arm. Constipation, neuropathy, nonneuropathy neurologic toxicity, and skin reactions were significantly more frequent in the MPT arm. The number of thromboembolic events was equal in the 2 treatment arms. In conclusion, MPT had a significant antimyeloma effect, but this did not translate into improved survival. This trial was registered at www.clinicaltrials.gov as #NCT00218855.

81 [92]. Walker, B. A., C. P. Wardell, et al. (2011). "Aberrant global methylation patterns affect the molecular pathogenesis and prognosis of multiple myeloma." Blood **117**(2): 553-562.

We used genome-wide methylation microarrays to analyze differences in CpG methylation patterns in cells relevant to the pathogenesis of myeloma plasma cells (B cells, normal plasma cells, monoclonal gammopathy of undetermined significance [MGUS], presentation

myeloma, and plasma cell leukemia). We show that methylation patterns in these cell types are capable of distinguishing nonmalignant from malignant cells and the main reason for this difference is hypomethylation of the genome at the transition from MGUS to presentation myeloma. In addition, gene-specific hypermethylation was evident at the myeloma stage. Differential methylation was also evident at the transition from myeloma to plasma cell leukemia with remethylation of the genome, particularly of genes involved in cell-cell signaling and cell adhesion, which may contribute to independence from the bone marrow microenvironment. There was a high degree of methylation variability within presentation myeloma samples, which was associated with cytogenetic differences between samples. More specifically, we found methylation subgroups were defined by translocations and hyperdiploidy, with t(4;14) myeloma having the greatest impact on DNA methylation. Two groups of hyperdiploid samples were identified, on the basis of unsupervised clustering, which had an impact on overall survival. Overall, DNA methylation changes significantly during disease progression and between cytogenetic subgroups.

82 [151]. Weber, D. M., C. Chen, et al. (2007). "Lenalidomide plus dexamethasone for relapsed multiple myeloma in North America." N Engl J Med **357**(21): 2133-2142.

BACKGROUND: Lenalidomide, an oral immunomodulatory drug that is similar to thalidomide but has a different safety profile, has clinical activity in relapsed or refractory multiple myeloma. METHODS: Patients in the United States and Canada who had received at least one previous therapy for multiple myeloma but who required additional treatment were randomly assigned to receive either 25 mg of lenalidomide or placebo on days 1 to 21 of a 28-day cycle. Both groups also received 40 mg of oral dexamethasone on days 1 to 4, 9 to 12, and 17 to 20 for the first four cycles. After the fourth cycle, 40 mg of dexamethasone was administered only on days 1 to 4. Safety, clinical response, time to progression, and overall survival were assessed. RESULTS: We assigned 177 patients to the lenalidomide group and 176 to the placebo group. Complete, near-complete, or partial responses occurred in 108 patients (61.0%) in the lenalidomide group and in 35 patients (19.9%) in the placebo group ($P<0.001$); complete responses occurred in 14.1% and 0.6%, respectively ($P<0.001$). The median time to progression was 11.1 months in the lenalidomide group and 4.7 months in the placebo group ($P<0.001$). Median overall survival times in the two groups were 29.6 months and 20.2 months, respectively ($P<0.001$). Grade 3 or 4 adverse

events were reported in 85.3% of the lenalidomide group and in 73.1% of the placebo group; these events resulted in study discontinuation in 19.8% and 10.2%, respectively. Grade 3 or 4 neutropenia and venous thromboembolism were more common in the lenalidomide group than in the placebo group
(41.2% vs. 4.6% and 14.7% vs. 3.4%, respectively; P<0.001 for both comparisons). CONCLUSIONS: Lenalidomide plus dexamethasone is superior to placebo plus dexamethasone in patients with relapsed or refractory multiple myeloma. (ClinicalTrials.gov number, NCT00056160 [ClinicalTrials.gov].).

83 [102]. Wijermans, P., M. Schaafsma, et al. (2010). "Phase III study of the value of thalidomide added to melphalan plus prednisone in elderly patients with newly diagnosed multiple myeloma: the HOVON 49 Study." J Clin Oncol **28**(19): 3160-3166.

PURPOSE: For several decades, the treatment of elderly patients with multiple myeloma (MM) has consisted of melphalan and prednisone (MP). The Dutch-Belgium Hemato-Oncology Cooperative Group (HOVON) investigated the efficacy of thalidomide added to MP (MP-T) in a randomized phase III trial. The objective of this study was to investigate the efficacy, toxicity, and effects on quality of life of MP-T. PATIENTS AND METHODS: A randomized phase III trial compared standard MP with MP-T (thalidomide 200 mg/d) in newly diagnosed patients with multiple myeloma older than age 65 years. Maintenance therapy with thalidomide 50 mg/d was administered to patients after MP-T until relapse. The primary end point was event-free survival (EFS); response rate, overall survival (OS), and progression-free survival (PFS) were secondary end points. RESULTS: An intent-to-treat analysis of 333 evaluable patients showed significantly higher response rates in MP-T-treated patients compared with MP-treated patients a response (> or = partial response: 66% v 45%, respectively; P < .001; and > or = very good partial response [VGPR]: 27% v 10%, respectively; P < .001). EFS was 13 months with MP-T versus 9 months with MP (P < .001). OS was 40 months with MP-T versus 31 months with MP (P = .05). CONCLUSION: This study demonstrates that thalidomide improves the response rate and VGPR in elderly patients with newly diagnosed MM. MP-T also results in a better EFS, PFS, and OS.

84 [41]. Witzig, T. E., K. M. Laumann, et al. (2013). "A phase III randomized trial of thalidomide plus zoledronic acid versus zoledronic acid alone in patients with asymptomatic multiple myeloma." Leukemia **27**(1): 220-225.

Patients with asymptomatic (smoldering) multiple myeloma (AMM) have a high risk of transformation to active multiple myeloma (MM). Bisphosphonates such as zoledronic acid (ZLD) reduce skeletal events in MM and the immunomodulatory agent thalidomide (Thal) has proven effectiveness in active MM. We hypothesized that treatment with Thal and ZLD would prolong the time to progression (TTP) to MM over ZLD alone. Eligible patients had asymptomatic MM and all patients received ZLD 4 mg intravenous monthly; the treatment arm also received Thal 200 mg per day. The TTP was superior for Thal/ZLD (n=35) patients compared with ZLD alone (n=33); median TTP of 2.4 years (95% confidence interval (CI): 1.4-3.6) versus 1.2 years (95% CI: 0.7-2.5) (hazard ratio (HR), 2.05; 95% CI: 1.1-3.8; P-value: 0.02). At 1 year, 86% of Thal/ZLD patients were progression free compared with 55% on ZLD alone (P=0.0048). The overall response rate after year 1 was 37% for Thal/ZLD with a median duration of response of 3.3 years (95% CI: 1.1-NA); there were no confirmed responses to ZLD alone (P=0.0004). The addition of Thal to standard ZLD produces anti-tumor responses whereas ZLD alone does not. Thal/ZLD also prolongs TTP from AMM to MM. This study provides the rationale for further studies in patients with AMM to delay chemotherapy.

85 [66]. Yakoub-Agha, I., J. Y. Mary, et al. (2012). "Low-dose vs. high-dose thalidomide for advanced multiple myeloma: a prospective trial from the Intergroupe Francophone du Myelome." Eur J Haematol **88**(3): 249-259.

This multicentre prospective randomised trial compared the efficacy and safety of two doses of thalidomide in patients with relapsed or refractory myeloma. The study was designed to test the non-inferior efficacy and to confirm the better tolerability of low-dose thalidomide as compared to a higher dose. Four hundred patients were randomly assigned to receive either 100 or 400 mg/day of thalidomide. Dexamethasone treatment was added in both arms for patients with stable disease or treatment failure at 12 weeks. The primary endpoint was 1-year overall survival (OS). Thalidomide 100 mg/day was better tolerated than 400 mg/day with less high-grade somnolence, constipation, nausea/vomiting and peripheral neuropathy (P < 0.001, P = 0.007, P = 0.03 and P = 0.007, respectively). In the per-protocol population (PP), the estimated 1-year OS rates were of 74.5% (n = 149) and 67.3% (n = 156) in the 400 and 100 groups, respectively. The upper limit of the difference between these rates was of 15.6% higher than the non-inferiority acceptable limit of 12.75%, and the hypothesis of non-inferiority of 100 could not be established (P = 0.14). On the other hand, when intent-to-treat (ITT) population was analysed, the non-inferiority was demonstrated because the 1-year OS rates were of

72.8% (n = 195) and 68.8% (n = 205) in the same groups, leading to an upper limit of the difference of 11.49% lower than the non-inferiority acceptable limit. In addition, in patients alive 12 weeks postrandomisation and those who received thalidomide plus dexamethasone, there were no significant differences in response rates, time to progression, progression-free survival and OS between the two groups. Collectively, low-dose thalidomide 100 mg/day has significant activity in advanced myeloma with an improved safety profile and can be a good salvage therapy in combination with dexamethasone.

86 [95]. Zonder, J. A., J. Crowley, et al. (2010). "Lenalidomide and high-dose dexamethasone compared with dexamethasone as initial therapy for multiple myeloma: a randomized Southwest Oncology Group trial (S0232)." Blood **116**(26): 5838-5841.

The Southwest Oncology Group conducted a randomized trial comparing lenalidomide (LEN) plus dexamethasone (DEX; n = 97) to placebo (PLC) plus DEX (n = 95) in newly diagnosed myeloma. Three 35-day induction cycles applied DEX 40 mg/day on days 1 to 4, 9 to 12, and 17 to 20 together with LEN 25 mg/day for 28 days or PLC. Monthly maintenance used DEX 40 mg/day on days 1 to 4 and 15 to 18 along with LEN 25 mg/day for 21 days or PLC. Crossover from PLC-DEX to LEN-DEX was encouraged on progression. One-year progression-free survival, overall response rate, and very good partial response rate were superior with LEN-DEX (78% vs 52%, P = .002; 78% vs 48%, P < .001; 63% vs 16%, P < .001), whereas 1-year overall survival was similar (94% vs 88%; P = .25). Toxicities were more pronounced with LEN-DEX (neutropenia grade 3 or 4: 21% vs 5%, P < .001; thromboembolic events despite aspirin prophylaxis: 23.5% [initial LEN-DEX or crossover] vs 5%; P < .001). This trial was registered at www.clinicaltrials.gov as #NCT00064038.

TOPIC: Myeloma + monoclonal antibod* (y or ies)

1 [270]. Adkins, D., V. Ratanatharathorn, et al. (2009). "Safety profile and clinical outcomes in a phase I, placebo-controlled study of siplizumab in acute graft-versus-host disease." Transplantation **88**(2): 198-202.

BACKGROUND: Acute graft-versus-host disease (GVHD) is a major complication of both bone marrow and hematologic stem cell allografts. T cells and natural killer (NK) cells have been linked to the development of GVHD. Modulation of these cells via the CD2 receptor may be a potentially important approach to the management of this disease.

METHODS: The safety profile and tolerability of siplizumab (MEDI-507), a humanized anti-CD2 IgG-1kappa monoclonal antibody, in the treatment of GVHD were evaluated in a phase I, double-blind, multiple-dose, placebo-controlled study. Thirty-four subjects with at least grade II acute GVHD were randomized to receive four doses of 0.012, 0.04, 0.12, or 0.4 mg/kg siplizumab or placebo intravenously every 3 days. Subjects received concurrent 2 mg/kg per day methylprednisolone for more than or equal to 10 days. RESULTS: No meaningful difference occurred between siplizumab and placebo groups in the incidence or severity of adverse events or laboratory test results. No increase in incidence of infection secondary to siplizumab treatment was observed. During 100 days postinitial infusion, a modest increase in resolution of GVHD, grade 0 (67% vs. 54%, P=0.0629), was reported for the siplizumab-treated group. CONCLUSION: Siplizumab administered with corticosteroid therapy for grade II or higher acute GVHD treatment exhibited an acceptable safety profile that would support further clinical development.

2 [284]. Baz, R., S. Fanning, et al. (2007). "Combination of rituximab and oral melphalan and prednisone in newly diagnosed multiple myeloma." Leuk Lymphoma **48**(12): 2338-2344.

Clonotypic B lymphocytes may underlie relapse of patients with multiple myeloma. Rituximab, a CD20 monoclonal antibody, may result in eradication of the monoclonal B cells. We conducted a phase II study of rituximab in combination with melphalan and prednisone therapy (MP) followed by rituximab maintenance in newly diagnosed multiple myeloma patients. Sixteen patients (35%) had CD20 positive bone marrow plasma cells, while 9 patients (20%) had unknown CD20 status. No patient had a complete remission, 26 patients (58%) had a partial response, 6 patients (13%) had a minimal response, and 8 patients (18%) had stable disease. The median event-free survival was 14 months, and the 7-year overall survival was 30%. The toxicity of the combination was overall manageable and consistent with what is generally noted with MP chemotherapy. The combination of rituximab to MP therapy did not result in improved response rate or event-free survival.

3 [224]. Bensinger, W., R. T. Maziarz, et al. (2012). "A phase 1 study of lucatumumab, a fully human anti-CD40 antagonist monoclonal antibody administered intravenously to patients with relapsed or refractory multiple myeloma." Br J Haematol **159**(1): 58-66.

In this open-label, multicentre, phase 1 study a fully human anti-CD40 antagonist monoclonal antibody, lucatumumab, was evaluated in

patients with relapsed/refractory multiple myeloma (MM). The primary objective was to determine the maximum tolerated dose (MTD) based on dose-limiting toxicities (DLTs). Secondary objectives included safety, pharmacokinetics, pharmacodynamics and antimyeloma activity. Twenty-eight patients, enrolled using a standard '3 + 3' dose escalation, received one or two (n = 3) cycles of lucatumumab 1.0, 3.0, 4.5 or 6.0 mg/kg once weekly for 4 weeks. Common lucatumumab-related adverse events were reversible, mild-to-moderate infusion reactions. Severe adverse events were anaemia, chills, hypercalcaemia and pyrexia (7% each). DLTs included grade 4 thrombocytopenia, grade 3 increased alanine aminotransferase and grade 4 increased lipase (n = 1 each). The MTD was 4.5 mg/kg. At doses >/=3.0 mg/kg, sustained receptor occupancy (>/=87%), observed throughout weekly infusions up to 5 weeks after the last infusion, correlated with an estimated half-life of 4-19 d. Twelve patients (43%) had stable disease, and one patient (4%) maintained a partial response for >/=8 months. These findings indicate that single-agent lucatumumab was well tolerated up to 4.5 mg/kg with modest clinical activity in relapsed/refractory MM, warranting further study as a combination therapy.

4 [222]. Benson, D. M., Jr., C. C. Hofmeister, et al. (2012). "A phase 1 trial of the anti-KIR antibody IPH2101 in patients with relapsed/refractory multiple myeloma." Blood **120**(22): 4324-4333.

Natural killer (NK) cells elicit cytotoxicity against multiple myeloma (MM); however, MM cells express HLA class I molecules as ligands to NK cell inhibitory killer immunoglobulin-like receptors (KIRs) as a means of immunoevasion. KIR-ligand mismatch may improve outcomes in allogeneic transplantation for MM. Extrapolating on this concept, we conducted a phase 1 trial of IPH2101, an anti-KIR antibody, in patients with relapsed/refractory MM. IPH2101 was administered intravenously every 28 days in 7 dose-escalated cohorts (0.0003-3 mg/kg) for up to 4 cycles. Pharmacokinetic, pharmacodynamic, and correlative immunologic studies were completed. A total of 32 patients were enrolled. The biologic endpoint of full KIR2D occupancy across the dosing cycle was achieved without dose-limiting toxicity or maximally tolerated dose. One severe adverse event was noted. Pharmacokinetic and pharmacodynamic findings approximated preclinical predictions, and IPH2101 enhanced ex vivo patient-derived NK cell cytotoxicity against MM. No objective responses were seen. No evidence of autoimmunity was observed. These findings suggest that IPH2101 is safe and tolerable at doses that achieve full inhibitory KIR saturation, and this approach warrants further

development in MM. This trial was registered at www.clinicaltrials.gov as #NCT00552396.

5 [212]. Bishton, M., A. Spencer, et al. (2013). "A single-arm, phase II study of the anti-Blys monoclonal antibody belimumab in symptomatic Waldenstrom macroglobulinemia." Clin Lymphoma Myeloma Leuk **13**(5): 575-578.

BACKGROUND: The B-lymphocyte stimulator (BLYS) protein is known to regulate immunoglobulin in normal B cells, and be overexpressed in B-cell malignancies, including Waldenstrom macroglobulinemia (WM). PATIENTS AND METHODS: This trial evaluated the safety and activity of belimumab, a monoclonal antibody targeting BLYS, in 12 patients with WM in a single-arm phase II study. RESULTS: Ten patients had stable disease with therapy, although no objective responses were seen. Correlative studies showed patients to have low or undetectable baseline serum levels of BLYS, with the administration of belimumab having no effect on B-cell numbers. CONCLUSION: Belimumab cannot be recommended as a single-agent therapy for the treatment of symptomatic WM, although further evaluation in combination with other agents would be justified.

6 [294]. Body, J. J., T. Facon, et al. (2006). "A study of the biological receptor activator of nuclear factor-kappaB ligand inhibitor, denosumab, in patients with multiple myeloma or bone metastases from breast cancer." Clin Cancer Res **12**(4): 1221-1228.

PURPOSE: Receptor activator of nuclear factor-kappaB ligand (RANKL) is essential for the differentiation, function, and survival of osteoclasts, which play a key role in establishment and propagation of skeletal disease in patients with multiple myeloma or bone metastases as well as many other skeletal diseases. Denosumab (AMG 162), a fully human monoclonal antibody to RANKL, was developed to treat patients with skeletal diseases. EXPERIMENTAL DESIGN: This was a randomized, double-blind, double-dummy, active-controlled, multicenter study to determine the safety and efficacy of denosumab in patients with breast cancer (n = 29) or multiple myeloma (n = 25) with radiologically confirmed bone lesions. Patients received a single dose of either denosumab (0.1, 0.3, 1.0, or 3.0 mg/kg s.c.) or pamidronate (90 mg i.v.). Bone antiresorptive effect was assessed by changes in urinary and serum N-telopeptide levels.
Pharmacokinetics of denosumab also were assessed. RESULTS: Following a single s.c. dose of denosumab, levels of urinary and serum N-telopeptide decreased within 1 day, and this decrease lasted through 84 days at the higher denosumab doses. Pamidronate also decreased bone turnover, but the effect diminished progressively through follow-up. Denosumab

injections were well tolerated. Mean half-lives of denosumab were 33.3 and 46.3 days for the two highest dosages. CONCLUSIONS: A single s.c. dose of denosumab given to patients with multiple myeloma or bone metastases from breast cancer was well tolerated and reduced bone resorption for at least 84 days. The decrease in bone turnover markers was similar in magnitude but more sustained than with i.v. pamidronate.

7 [253]. de Weers, M., Y. T. Tai, et al. (2011). "Daratumumab, a novel therapeutic human CD38 monoclonal antibody, induces killing of multiple myeloma and other hematological tumors." J Immunol **186**(3): 1840-1848.

CD38, a type II transmembrane glycoprotein highly expressed in hematological malignancies including multiple myeloma (MM), represents a promising target for mAb-based immunotherapy. In this study, we describe the cytotoxic mechanisms of action of daratumumab, a novel, high-affinity, therapeutic human mAb against a unique CD38 epitope. Daratumumab induced potent Ab-dependent cellular cytotoxicity in CD38-expressing lymphoma- and MM-derived cell lines as well as in patient MM cells, both with autologous and allogeneic effector cells. Daratumumab stood out from other CD38 mAbs in its strong ability to induce complement-dependent cytotoxicity in patient MM cells. Importantly, daratumumab-induced Ab-dependent cellular cytotoxicity and complement-dependent cytotoxicity were not affected by the presence of bone marrow stromal cells, indicating that daratumumab can effectively kill MM tumor cells in a tumor-preserving bone marrow microenvironment. In vivo, daratumumab was highly active and interrupted xenograft tumor growth at low dosing. Collectively, our results show the versatility of daratumumab to effectively kill CD38-expressing tumor cells, including patient MM cells, via diverse cytotoxic mechanisms. These findings support clinical development of daratumumab for the treatment of CD38-positive MM tumors.

8 [210]. Dimopoulos, M. A., R. Garcia-Sanz, et al. (2013). "Primary therapy of Waldenstrom macroglobulinemia (WM) with weekly bortezomib, low-dose dexamethasone, and rituximab (BDR): long-term results of a phase 2 study of the European Myeloma Network (EMN)." Blood **122**(19): 3276-3282.

In this phase 2 multicenter trial, we evaluated the activity of bortezomib, dexamethasone, and rituximab (BDR) combination in previously untreated symptomatic patients with Waldenstrom macroglobulinemia (WM). To prevent immunoglobulin M (IgM) "flare," single agent bortezomib (1.3 mg/m(2) IV days 1, 4, 8, and 11; 21-day cycle), was followed by weekly IV bortezomib (1.6 mg/m(2) days 1, 8, 15, and 22) every 35 days for 4

additional cycles, followed by IV dexamethasone (40 mg) and IV rituximab (375 mg/m(2)) in cycles 2 and 5. Fifty-nine patients were treated; 45.5% and 40% were high and intermediate risk per the International Prognostic Scoring System for WM. On intent to treat, 85% responded (3% complete response, 7% very good partial response, 58% partial response [PR]). In 11% of patients, an increase of IgM >/=25% was observed after rituximab; no patient required plasmapheresis. After a minimum follow-up of 32 months, median progression-free survival was 42 months, 3-year duration of response for patients with >/=PR was 70%, and 3-year survival was 81%. Peripheral neuropathy occurred in 46% (grade >/=3 in 7%); only 8% discontinued bortezomib due to neuropathy. BDR is rapidly acting, well tolerated, and nonmyelotoxic, inducing durable responses in previously untreated WM.

9 [250]. Henry, D. H., L. Costa, et al. (2011). "Randomized, double-blind study of denosumab versus zoledronic acid in the treatment of bone metastases in patients with advanced cancer (excluding breast and prostate cancer) or multiple myeloma." J Clin Oncol **29**(9): 1125-1132.

PURPOSE: This study compared denosumab, a fully human monoclonal anti-receptor activator of nuclear factor kappa-B ligand antibody, with zoledronic acid (ZA) for delaying or preventing skeletal-related events (SRE) in patients with advanced cancer and bone metastases (excluding breast and prostate) or myeloma. PATIENTS AND METHODS: Eligible patients were randomly assigned in a double-blind, double-dummy design to receive monthly subcutaneous denosumab 120 mg (n = 886) or intravenous ZA 4 mg (dose adjusted for renal impairment; n = 890). Daily supplemental calcium and vitamin D were strongly recommended. The primary end point was time to first on-study SRE (pathologic fracture, radiation or surgery to bone, or spinal cord compression). RESULTS: Denosumab was noninferior to ZA in delaying time to first on-study SRE (hazard ratio, 0.84; 95% CI, 0.71 to 0.98; P = .0007). Although directionally favorable, denosumab was not statistically superior to ZA in delaying time to first on-study SRE (P = .03 unadjusted; P = .06 adjusted for multiplicity) or time to first-and-subsequent (multiple) SRE (rate ratio, 0.90; 95% CI, 0.77 to 1.04; P = .14). Overall survival and disease progression were similar between groups. Hypocalcemia occurred more frequently with denosumab.

Osteonecrosis of the jaw occurred at similarly low rates in both groups. Acute-phase reactions after the first dose occurred more frequently with ZA, as did renal adverse events and elevations in serum creatinine based on National Cancer Institute Common Toxicity Criteria for Adverse Events

grading. CONCLUSION: Denosumab was noninferior (trending to superiority) to ZA in preventing or delaying first on-study SRE in patients with advanced cancer metastatic to bone or myeloma. Denosumab represents a potential novel treatment option with the convenience of subcutaneous administration and no requirement for renal monitoring or dose adjustment.

10 [264]. Hussein, M., J. R. Berenson, et al. (2010). "A phase I multidose study of dacetuzumab (SGN-40; humanized anti-CD40 monoclonal antibody) in patients with multiple myeloma." Haematologica **95**(5): 845-848.

This first-in-human, phase I study evaluated the safety, maximum-tolerated dose, pharmacokinetics, and antitumor activity of dacetuzumab in 44 patients with advanced multiple myeloma. Patients received intravenous dacetuzumab, either in 4 uniform weekly doses (first 4 cohorts) or using a 5-week intrapatient dose escalation schedule (7 subsequent cohorts; the last 3 cohorts received steroid pre-medication). An initial dose of 4 mg/kg dacetuzumab exceeded the maximum-tolerated dose for uniform weekly dosing. Intrapatient dose escalation with steroid pre-medication appeared effective in reducing symptoms of cytokine release syndrome and the maximum-tolerated dose with this dosing schema was 12 mg/kg/week. Adverse events potentially related to dacetuzumab included cytokine release syndrome symptoms, non-infectious ocular inflammation, and elevated hepatic enzymes. Peak dacetuzumab blood levels increased with dose. Nine patients (20%) had a best clinical response of stable disease. The observed safety profile suggested that dacetuzumab may be combined with other multiple myeloma therapies. Two combination trials are ongoing. Clinical trials gov identifier: NCT00079716.

11 [235]. Jakubowiak, A. J., D. M. Benson, et al. (2012). "Phase I trial of anti-CS1 monoclonal antibody elotuzumab in combination with bortezomib in the treatment of relapsed/refractory multiple myeloma." J Clin Oncol **30**(16): 1960-1965.

PURPOSE: To evaluate the maximum-tolerated dose (MTD), safety, and efficacy of elotuzumab in combination with bortezomib in patients with relapsed or relapsed and refractory multiple myeloma (MM). PATIENTS AND METHODS: Elotuzumab (2.5, 5.0, 10, or 20 mg/kg intravenously [IV]) and bortezomib (1.3 mg/m(2) IV) were administered on days 1 and 11 and days 1, 4, 8, and 11, respectively, in 21-day cycles by using a 3 + 3 dose-escalation design. Patients with stable disease or better after four cycles could continue treatment until disease progression or unexpected

toxicity. Responses were assessed during each cycle by using European Group for Blood and Marrow Transplantation (EBMT) criteria. RESULTS: Twenty-eight patients with a median of two prior therapies were enrolled; three patients each received 2.5, 5.0, and 10 mg/kg of elotuzumab and 19 received 20 mg/kg (six during dose escalation and 13 during an expansion phase). No dose-limiting toxicities were observed during cycle 1 of the dose-escalation phase, and the MTD was not reached up to the maximum planned dose of 20 mg/kg. The most frequent grade 3 to 4 adverse events (AEs) were lymphopenia (25%) and fatigue (14%). Two elotuzumab-related serious AEs of chest pain and gastroenteritis occurred in one patient. An objective response (a partial response or better) was observed in 13 (48%) of 27 evaluable patients and in two (67%) of three patients refractory to bortezomib. Median time to progression was 9.46 months. CONCLUSION: The combination of elotuzumab and bortezomib was generally well-tolerated and showed encouraging activity in patients with relapsed/refractory MM.

12 [203]. Jiang, H., W. Zhang, et al. (2014). "Transfection of chimeric anti-CD138 gene enhances natural killer cell activation and killing of multiple myeloma cells." Mol Oncol **8**(2): 297-310.

Reprogramming of NK cells with a chimeric antigen receptor (CAR) proved an effective strategy to increase NK cell reactivity and recognition specificity toward tumor cells. To enhance the cytotoxicity of NK cells against CD138-positive multiple myeloma (MM) cells, we generated genetically modified NK-92MI cells carrying a CAR that consists of an anti-CD138 single-chain variable fragment (scFv) fused to the CD3zeta chain as a signaling moiety. The genetic modification through a lentiviral vector did not affect the intrinsic cytolytic activity of NK-92MI toward human erythroleukemic cell line K562 cells or CD138-negative targets. However, these retargeted NK-92MI (NK-92MI-scFv) displayed markedly enhanced cytotoxicity against CD138-positive human MM cell lines (RPMI8226, U266 and NCI-H929) and primary MM cells at various effector-to-target ratios (E:T) as compared to the empty
vector-transfected NK-92MI (NK-92MI-mock). In line with the enhanced cytotoxicity of NK-92MI-scFv, significant elevations in the secretion of granzyme B, interferon-gamma and proportion of CD107a expression were also found in NK-92MI-scFv in response to CD138-positive targets compared with NK-92MI-mock. Most importantly, the enhancement in the cytotoxicity of NK-92MI-scFv did not attenuate with 10Gy-irradiation that sufficiently blocked cell proliferation. Moreover, the irradiated NK-92MI-scFv exerted definitely intensified anti-tumor activity toward CD138-

positive MM cells than NK-92MI-mock in the xenograft NOD-SCID mouse model. This study provides the rationale and feasibility for adoptive immunotherapy with CD138-specific CAR-modified NK cells in CD138-positive plasmacytic malignancies, which potentially further improves remission quality and prolongs the remission duration of patients with MM after upfront chemotherapy.

13 [209]. Kaufman, J. L., R. Niesvizky, et al. (2013). "Phase I, multicentre, dose-escalation trial of monotherapy with milatuzumab (humanized anti-CD74 monoclonal antibody) in relapsed or refractory multiple myeloma." Br J Haematol **163**(4): 478-486.

CD74, expressed in multiple myeloma (MM), was evaluated as a target for immunotherapy with milatuzumab (a humanized anti-CD74 antibody). In a multicentre dose escalation study, 25 patients with advanced MM received milatuzumab doses of 1.5 (N = 8), 4.0 (N = 9), 8.0 (N = 4) or 16.0 mg/kg (N = 4) administered twice weekly x 4. They had a median of 5 prior treatments (17 post >/= 1 stem cell transplantation) and were refractory (N = 7) or relapsed (N = 18) with generally short-lived responses to last treatment (median 4.0 months). After increasing prophylactic medications and slowing administration, infusions were well tolerated (National Cancer Institute-Common Terminology Criteria v3 toxicity Grades 1-2) with no dose-limiting toxicity at higher doses. Only one patient developed borderline positive human anti-milatuzumab antibody titres of uncertain clinical significance. Although milatuzumab was rapidly cleared from circulation with little serum accumulation and low trough levels, B-cell levels were moderately decreased with treatment (median decrease, 34%). There were no objective responses by European Group for Blood and Marrow Transplantation criteria, but 5 of 19 patients (26%) who completed treatment in this heavily pretreated and generally refractory group had stable disease for >/= 3 months post-treatment (one continuing for 17 months). Disease stabilization and evidence of pharmacodynamic activity support further development for use in combination with other agents or as a drug conjugate.

14 [214]. Kurzrock, R., P. M. Voorhees, et al. (2013). "A phase I, open-label study of siltuximab, an anti-IL-6 monoclonal antibody, in patients with B-cell non-Hodgkin lymphoma, multiple myeloma, or Castleman disease." Clin Cancer Res **19**(13): 3659-3670.

PURPOSE: To evaluate the safety and pharmacokinetics of siltuximab, an anti-interleukin-6 chimeric monoclonal antibody (mAb) in patients with B-cell non-Hodgkin lymphoma (NHL), multiple myeloma, or Castleman

disease. EXPERIMENTAL DESIGN: In an open-label, dose-finding, 7 cohort, phase I study, patients with NHL, multiple myeloma, or symptomatic Castleman disease received siltuximab 3, 6, 9, or 12 mg/kg weekly, every 2 weeks, or every 3 weeks. Response was assessed in all disease types. Clinical benefit response (CBR; composite of hemoglobin, fatigue, anorexia, fever/night sweats, weight, largest lymph node size) was also evaluated in Castleman disease. RESULTS: Sixty-seven patients received a median of 16 siltuximab doses for a median of 8.5 (maximum 60.5) months; 29 were treated 1 year or longer. There was no dose-limiting toxicity, antibodies to siltuximab, or apparent dose-toxicity relationship. The most frequently reported possible drug-related adverse events were thrombocytopenia (25%), hypertriglyceridemia (19%), neutropenia (19%), leukopenia (18%), hypercholesterolemia (15%), and anemia (10%). None of these events led to dose delay/discontinuation except for neutropenia and thrombocytopenia (n = 1 each). No treatment-related deaths occurred. C-reactive protein (CRP) suppression was most pronounced at 12 mg/kg every 3 weeks. Mean terminal-phase half-life of siltuximab ranged 17.73 to 20.64 days. Thirty-two of 37 (86%) patients with Castleman disease improved in 1 or more CBR component; 12 of 36 evaluable Castleman disease patients had radiologic response [complete response (CR), n = 1; partial response (PR), n = 11], including 8 of 19 treated with 12 mg/kg; 2 of 14 (14%) evaluable NHL patients had PR; 2 of 13 (15%) patients with multiple myeloma had CR. CONCLUSION: No dose-related or cumulative toxicity was apparent across all disease indications. A dose of 12 mg/kg every 3 weeks was recommended on the basis of the high response rates in Castleman disease and the sustained CRP suppression. Randomized studies are ongoing in Castleman disease and multiple myeloma.

15 [280]. Lacy, M. Q., M. Alsina, et al. (2008). "Phase I, pharmacokinetic and pharmacodynamic study of the anti-insulinlike growth factor type 1 Receptor monoclonal antibody CP-751,871 in patients with multiple myeloma." J Clin Oncol **26**(19): 3196-3203.

PURPOSE: A phase I first-in-human study was conducted to characterize the safety, tolerability, pharmacokinetic, and pharmacodynamic properties of the anti-insulinlike growth factor 1 receptor (IGF-IR) monoclonal antibody CP-751,871. PATIENTS AND METHODS: After informed consent and screening, 47 patients with multiple myeloma in relapse or refractory phase were enrolled into 11 dose-escalation cohorts of CP-751,871 at doses from 0.025 to 20 mg/kg for 4 weeks. Patients with less than a partial response to CP-751,871 treatment were eligible to receive CP-

751,871 in combination with oral dexamethasone at the discretion of the investigator. Treatment with CP-751,871 and rapamycin with or without dexamethasone was also offered to patients enrolled in the 10 and 20 mg/kg cohorts with less than a partial response to initial therapy with single-agent CP-751,871. RESULTS: No CP-751,871-related dose-limiting toxicities were identified. Plasma CP-751,871 concentrations increased with dose and concentration-time profiles were consistent with those of antibodies with target-mediated disposition. Importantly, CP-751,871 administration led to a decrease in granulocyte IGF-IR expression and serum insulinlike growth factor 1 accumulation at high doses, suggesting systemic IGF-IR inhibition. Tumor response was assessed according to the European Group for Blood and Marrow Transplantation criteria. Nine responses were reported in 27 patients treated with CP-751,871 in combination with dexamethasone. Of interest, two of the patients with a partial response were progressing from dexamethasone treatment at study entry. CONCLUSION: These data indicate that CP-751,871 is well tolerated and may constitute a novel agent in the treatment of multiple myeloma.

16 [249]. Leleu, X., W. Xie, et al. (2011). "The role of serum immunoglobulin free light chain in response and progression in waldenstrom macroglobulinemia." Clin Cancer Res **17**(9): 3013-3018.

INTRODUCTION: The serum free light chain (sFLC) has been widely used in the assessment of response in patients with multiple myeloma and other plasma cell dyscrasias. However, its use in Waldenstrom macroglobulinemia (WM) has not been previously assessed. We sought to examine the role of sFLC in response and progression of patients with WM. METHODS: This study was conducted in a cohort of 48 patients with a diagnosis of WM, untreated (n = 20) or relapsed/refractory (n = 28), prospectively treated on a bortezomib and rituximab trial. RESULTS: Involved FLC (iFLC) response occurred in 79% patients versus 60% by M-spike protocol criteria. The median time to response was shorter with iFLC than per protocol (2.1 and 3.7 months; P = 0.05). Progression defined using iFLC also correlated well to progression in the protocol (kappa = 0.63). However, the median time to progression (TTP) was more rapid by iFLC than per protocol (13.7 and 18.9 months). We also confirmed that a flare in iFLC in post-rituximab therapy did not correlate with lack of response or shorter TTP. CONCLUSION: Involved sFLC may be a useful marker of tumor measurement, showing earlier response and progression compared with IgM or M-spike measurements.

17 [300]. Ljungman, P., H. Nahi, et al. (2005). "Vaccination of patients with haematological malignancies with one or two doses of influenza vaccine: a randomised study." Br J Haematol **130**(1): 96-98.

An open, randomised study was performed to determine whether two doses of influenza vaccine were more effective than one to elicit an immune response in 70 patients with haematological malignancies. The responses were not improved by two doses compared with one (influenza A virus serotypes H1/N1 18% vs. 22% and H3/N2 26% vs. 14%; influenza B 25% vs. 22%). The results were similar in patients with ongoing and discontinued therapy. Patients treated with monoclonal antibodies for lymphoma had very poor responses. We conclude that two doses of influenza vaccine do not improve the antibody response in patients with haematological malignancies.

18 [229]. Lonial, S., R. Vij, et al. (2012). "Elotuzumab in combination with lenalidomide and low-dose dexamethasone in relapsed or refractory multiple myeloma." J Clin Oncol **30**(16): 1953-1959.

PURPOSE: This phase I study evaluated elotuzumab, lenalidomide, and dexamethasone in patients with relapsed or refractory multiple myeloma (MM). PATIENTS AND METHODS: Three cohorts were enrolled and treated with elotuzumab (5.0, 10, or 20 mg/kg intravenously) on days 1, 8, 15, and 22 of a 28-day cycle in the first two cycles, and days 1 and 15 of each subsequent cycle; lenalidomide 25 mg orally [PO] on days 1 to 21; and dexamethasone 40 mg PO weekly. Dose-limiting toxicities (DLTs) were assessed during cycle 1 of each cohort, and clinical responses were evaluated during each cycle. The first five patients received up to six cycles of therapy; subsequent patients were treated until disease progression. RESULTS: Twenty-nine patients with advanced MM and a median of three prior MM therapies were enrolled; 28 patients were treated, three each in the 5.0-mg/kg and 10-mg/kg cohorts and 22 in the 20-mg/kg cohort. No DLTs were observed up to the maximum proposed dose of 20 mg/kg. The most frequent grade 3 to 4 toxicities were neutropenia (36%) and thrombocytopenia (21%). Two patients experienced a serious infusion reaction (one grade 4 anaphylactic reaction and one grade 3 stridor) during the first treatment cycle. Objective responses were obtained in 82% (23 of 28) of treated patients. After a median of 16.4 months follow-up, the median time to progression was not reached for patients in the 20-mg/kg cohort who were treated until disease progression. CONCLUSION: The combination of elotuzumab, lenalidomide, and low-dose dexamethasone was generally well tolerated and showed encouraging response rates in patients with relapsed or refractory MM.

19 [251]. Moreau, P., F. Cavallo, et al. (2011). "Phase I study of the anti insulin-like growth factor 1 receptor (IGF-1R) monoclonal antibody, AVE1642, as single agent and in combination with bortezomib in patients with relapsed multiple myeloma." Leukemia **25**(5): 872-874.

20 [297]. Moreau, P., C. Hullin, et al. (2006). "Tandem autologous stem cell transplantation in high-risk de novo multiple myeloma: final results of the prospective and randomized IFM 99-04 protocol." Blood **107**(1): 397-403.

The combination of high levels of beta2-microglobulin (beta2-m) and chromosome 13 deletion allows identification of a high-risk subgroup of patients with de novo multiple myeloma (MM). In this population of patients, we have evaluated the impact of a murine anti-interleukin 6 (anti-IL-6) monoclonal antibody (BE-8) as part of the second conditioning regimen in a multicenter prospective randomized trial of tandem autologous stem cell transplantation (ASCT). Conditioning for the first ASCT was accomplished with melphalan 200 mg/m2 and for the second one with melphalan 220 mg/m2 plus dexamethasone with or without BE-8 infusion. This trial included 219 patients, of whom 166 were randomized, 85 without BE-8 (arm A) and 81 with BE-8 (arm B). The median overall survival (OS) and event-free survival (EFS) times of the whole group of patients were 41 and 30 months, respectively. Response rates, OS, and EFS were not different between the 2 arms of the trial. OS at 54 months was 46% in arm A and 51% in arm B (P = .90); median EFS was 35 months in arm A and 31 in arm B (P = .39). In high-risk patients the dose intensity of melphalan at 420 mg/m2 led to encouraging results, but the addition of anti-IL-6 monoclonal antibody to the second conditioning regimen did not improve either OS nor EFS.

21 [290]. Moreau, P., L. Voillat, et al. (2007). "Rituximab in CD20 positive multiple myeloma." Leukemia **21**(4): 835-836.

22 [273]. Ocadlikova, D., L. Zahradova, et al. (2009). "[The preparation of anticancer vaccine for patients with multiple myeloma on the base of monoclonal immunoglobulin loaded dendritic cells]." Klin Onkol **22**(2): 67-72.

BACKGROUND: On June 2006, phase II clinical trial focused on anticancer vaccination of multiple myeloma patients, was started. On September 2007, the immune and clinical response evaluation of first four patients was finished.The anticancer vaccine contained dendritic cells loaded with monoclonal immunoglobulin produced by myeloma cells. METHODS AND PATIENTS: Within the frame of phase II clinical trial were vaccinated

four myeloma patients with stable disease. It was administered six vaccines for each patient, monthly. The dendritic cells were cultured from the patient's peripheral blood mononuclear cells and loaded with autologous monoclonal immunoglobulin under the good manufacturing practice conditions. After the safety and quality control, the satisfactory vaccine was administered to the patient. The functional characteristic of dendritic cells was evaluated using flow cytometry, the immune response was evaluated using ELISpot. The clinical response was monitored using monoclonal immunoglobulin concentration in patient's sera. RESULTS AND CONCLUSION: The immune response detected using ELISpot was observed in 3/4 patients. The monoclonal immunoglobulin concentration was changeable for all twelve months, but never exceeded the range of 25% for minimal clinical response achievement. During the vaccination, no significant toxicities or negative side-effects were observed. The clinical trial is going on with vaccination other patients with multiple myeloma.

23 [200]. Patnaik, A., G. J. Weiss, et al. (2014). "Phase I ficlatuzumab monotherapy or with erlotinib for refractory advanced solid tumours and multiple myeloma." Br J Cancer **111**(2): 272-280.

BACKGROUND: Ficlatuzumab, a humanised hepatocyte growth factor (HGF) IgG1kappa inhibitory monoclonal antibody, was evaluated for recommended phase II dose (RP2D), safety, pharmacokinetics (PKs), antidrug antibody (ADA), pharmacodynamics (PDs) and antitumour activity as monotherapy or combined with erlotinib. METHODS: Patients with solid tumours received ficlatuzumab 2, 5, 10 or 20 mg kg(-1) intravenously every 2 weeks (q2w). Additional patients were treated at the RP2D erlotinib. RESULTS: Forty-one patients enrolled at doses 20 mg kg(-1). Common adverse events (AEs) included peripheral oedema, fatigue and nausea. Three patients experienced grade 3 treatment-related hyperkalaemia/hypokalaemia, diarrhoea or fatigue. Best overall response (44%) was stable disease (SD); median duration was 5.5 months (0.4-18.7 months). One patient has been on therapy with SD for >4 years. Pharmacokinetics of ficlatuzumab showed low clearance (0.17-0.26 ml h(-1) kg(-1)), a half-life of 6.8-9.4 days and dose-proportional exposure. Ficlatuzumab/erlotinib had no impact on the PK of either agent. No ADAs were detected. Ficlatuzumab increased serum HGF levels. CONCLUSIONS: Recommended phase II dose is 20 mg kg(-1) q2w for ficlatuzumab monotherapy or with erlotinib. Preliminary antitumour activity and manageable AEs were observed. Pharmacokinetics were dose-proportional and consistent with other IgG therapeutics.

Ficlatuzumab was not immunogenic, and serum HGF was a potential PD marker.

24 [201]. San-Miguel, J., J. Blade, et al. (2014). "Phase 2 randomized study of bortezomib-melphalan-prednisone with or without siltuximab (anti-IL-6) in multiple myeloma." Blood **123**(26): 4136-4142.

Because interleukin-6 (IL-6) is considered important in the proliferation of early multiple myeloma (MM), we hypothesized that the addition of the anti-IL-6 monoclonal antibody siltuximab to the bortezomib-melphalan-prednisone (VMP) regimen would improve outcomes in transplant-ineligible patients with newly diagnosed MM. One hundred and six patients were randomized to receive 9 cycles of VMP or VMP plus siltuximab (11 mg/kg every 3 weeks) followed by siltuximab maintenance. Baseline characteristics were well balanced except for immunoglobulin A subtype and 17p deletions. With a complete response (CR) rate of 27% on siltuximab plus VMP (S+VMP) and 22% on VMP, the study did not confirm its hypothesis that the addition of siltuximab would increase the CR rate by at least 10%. Overall response rate was 88% on S+VMP and 80% on VMP, and at least very good partial response rates were 71% and 51% (P = .0382), respectively. Median progression-free survival (17 months) and 1-year overall survival (88%) were identical in the 2 arms. Grade >/=3 adverse-event incidence was 92% on S+VMP and 81% on VMP (P = .09), with trends toward more hematologic events and infections on S+VMP. Maintenance therapy with siltuximab was well tolerated. In conclusion, the addition of siltuximab to VMP did not improve the CR rate or long-term outcomes. This study was registered at http://clinicaltrials.gov as #NCT00911859.

25 [207]. Thomas, S. K., A. Suvorov, et al. (2014). "Evaluation of the QTc prolongation potential of a monoclonal antibody, siltuximab, in patients with monoclonal gammopathy of undetermined significance, smoldering multiple myeloma, or low-volume multiple myeloma." Cancer Chemother Pharmacol **73**(1): 35-42.

PURPOSE: A phase 1 study evaluated the QTc prolongation potential of siltuximab, a chimeric, anti-interleukin-6 mAb, in patients with monoclonal gammopathy of undetermined significance (MGUS), smoldering multiple myeloma (SMM), or low-volume MM. METHODS: Patients with baseline QTcF and QTcB </= 500 ms, QRS < 100 ms, PR < 200 ms and no significant cardiac disease received siltuximab 15 mg/kg q3w, the highest dosage used in clinical studies, for 4 cycles. Twelve-lead ECGs obtained at multiple time points pre- and post-infusion at cycles 1 and 4 were

evaluated by central cardiology laboratory. No effect on QTc interval was concluded if the upper limit of least square (LS) mean 90 % CI for QTc change from baseline at each time point was <20 ms. RESULTS: An effect on QTc prolongation was ruled out, as the upper bound of 90 % CI was <10 ms at each time point in 27 evaluable patients (13 MGUS, 13 SMM, 1 low-volume MM) with no differences between disease types. Maximum mean QTc increase from baseline occurred 3 h after cycle 1 infusion (QTcF = 3.2 [LS mean 90 % CI -0.01, 6.45] ms; QTcB = 2.7 [-0.69, 6.14] ms). At all other time points, mean QTcF and QTcB increase from baseline was </=1.5 ms and upper bound 90 % CI was </=5.1 ms. Twenty patients had mostly low-grade AEs, including nausea, fatigue (20 % each); thrombocytopenia, headache (each 13 %); dyspnea, leukopenia, neutropenia, paresthesia, abnormal hepatic function, URTI (each 10 %). Three MGUS patients achieved 50 % M-protein reduction. There was no association between siltuximab pharmacokinetics and QTc interval. CONCLUSIONS: Siltuximab did not affect the QTc interval. Overall safety was similar to other single-agent siltuximab studies.

26 [268]. van Rhee, F., S. M. Szmania, et al. (2009). "Combinatorial efficacy of anti-CS1 monoclonal antibody elotuzumab (HuLuc63) and bortezomib against multiple myeloma." Mol Cancer Ther **8**(9): 2616-2624.

Monoclonal antibody (mAb) therapy for multiple myeloma, a malignancy of plasma cells, has not been clinically efficacious in part due to a lack of appropriate targets. We recently reported that the cell surface glycoprotein CS1 (CD2 subset 1, CRACC, SLAMF7, CD319) was highly and universally expressed on myeloma cells while having restricted expression in normal tissues. Elotuzumab (formerly known as HuLuc63), a humanized mAb targeting CS1, is currently in a phase I clinical trial in relapsed/refractory myeloma. In this report we investigated whether the activity of elotuzumab could be enhanced by bortezomib, a reversible proteasome inhibitor with significant activity in myeloma. We first showed that elotuzumab could induce patient-derived myeloma cell killing within the bone marrow microenvironment using a SCID-hu mouse model. We next showed that CS1 gene and cell surface protein expression persisted on myeloma patient-derived plasma cells collected after bortezomib administration. In vitro bortezomib pretreatment of myeloma targets significantly enhanced elotuzumab-mediated antibody-dependent cell-mediated cytotoxicity, both for OPM2 myeloma cells using natural killer or peripheral blood mononuclear cells from healthy donors and for primary myeloma cells using autologous natural killer effector cells. In an OPM2

myeloma xenograft model, elotuzumab in combination with bortezomib exhibited significantly enhanced in vivo antitumor activity. These findings provide the rationale for a clinical trial combining elotuzumab and bortezomib, which will test the hypothesis that combining both drugs would result in enhanced immune lysis of myeloma by elotuzumab and direct targeting of myeloma by bortezomib.

27 [213]. von Tresckow, B., B. Boell, et al. (2014). "Anti-epidermal growth factor receptor antibody cetuximab in refractory or relapsed multiple myeloma: a phase II prospective clinical trial." Leuk Lymphoma **55**(3): 695-697.

28 [217]. Voorhees, P. M., R. F. Manges, et al. (2013). "A phase 2 multicentre study of siltuximab, an anti-interleukin-6 monoclonal antibody, in patients with relapsed or refractory multiple myeloma." Br J Haematol **161**(3): 357-366.

Interleukin-6 (IL6) plays a central role in multiple myeloma pathogenesis and confers resistance to corticosteroid-induced apoptosis. We therefore evaluated the efficacy and safety of siltuximab, an anti-IL6 monoclonal antibody, alone and in combination with dexamethasone, for patients with relapsed or refractory multiple myeloma who had >/= 2 prior lines of therapy, one of which had to be bortezomib-based. Fourteen initial patients received siltuximab alone, 10 of whom had dexamethasone added for suboptimal response; 39 subsequent patients were treated with concurrent siltuximab and dexamethasone. Patients received a median of four prior lines of therapy, 83% were relapsed and refractory, and 70% refractory to their last dexamethasone-containing regimen. Suppression of serum C-reactive protein levels, a surrogate marker of IL6 inhibition, was demonstrated. There were no responses to siltuximab but combination therapy yielded a partial (17%) + minimal (6%) response rate of 23%, with responses seen in dexamethasone-refractory disease. The median time to progression, progression-free survival and overall survival for combination therapy was 4.4, 3.7 and 20.4 months respectively. Haematological toxicity was common but manageable. Infections occurred in 57% of combination-treated patients, including >/= grade 3 infections in 18%. Further study of siltuximab in modern corticosteroid-containing myeloma regimens is warranted, with special attention to infection-related toxicity.

29 [275]. Wang, M., L. Fayad, et al. (2008). "Phase 2 trial of rituximab plus hyper-CVAD alternating with rituximab plus methotrexate-cytarabine for relapsed or refractory aggressive mantle cell lymphoma." Cancer **113**(10): 2734-2741.

BACKGROUND: Relapsed or refractory mantle cell lymphoma has a very poor prognosis. The authors evaluated the response rates and survival times of patients treated with an intense regimen known to be effective against untreated aggressive mantle cell lymphoma: rituximab plus hyper-CVAD (cyclophosphamide, vincristine, doxorubicin, and dexamethasone) alternating with rituximab plus methotrexate-cytarabine. METHODS: In this prospective, open-label, phase 2 study, patients received this combination for 6 to 8 cycles. Twenty-nine patients were evaluable for response. RESULTS: The median number of cycles received was 5 (range, 1-7 cycles), and the overall response rate was 93% (45% complete response [CR] or CR unconfirmed [CRu] and 48% partial response [PR]). All 5 patients previously resistant to treatment had a response (1 CR, 4 PR), and both patients whose disease did not change in response to prior therapy had PRs. Toxic events occurring in response to the 104 cycles given included neutropenic fever (11%), grade 3 or 4 neutropenia (74%), and grade 3 or 4 thrombocytopenia (63%). There were no deaths from toxicity. At a median follow-up of 40 months (range, 5-48 months), the median failure-free survival time was 11 months with no plateau in the survival curve. CONCLUSIONS: This combination chemotherapy was effective for refractory/relapsed mantle cell lymphoma.

30 [223]. Yang, B., X. C. Lu, et al. (2012). "Repeated transfusions of autologous cytokine-induced killer cells for treatment of haematological malignancies in elderly patients: a pilot clinical trial." Hematol Oncol **30**(3): 115-122.

The elderly population is susceptible to haematological malignancies, and these elderly patients are intolerant to cytotoxic drugs. Therefore, the exploration of a safe and reliable strategy exclusive of chemotherapy is critical in improving the prognosis of elderly patients with haematological malignancies. We evaluated the safety and the efficacy of autologous cytokine-induced killer (CIK) cells combined with recombinant human interleukin 2 (rhIL-2) in the treatment of haematological malignancies in elderly patients. Peripheral blood mononuclear cells were isolated from 20 elderly patients with haematological malignancies, then augmented by priming with interferon gamma, rhIL-2 and CD3 monoclonal antibody. The autologous CIK cells (2-3 x 10(9)) were transfused back to patients, followed by a subcutaneous injection of IL-2 (1 mU/day) for 10 consecutive days. The regimen was repeated every 4 weeks. The host cellular immune function, tumour-related biological parameters, imaging characteristics, disease condition, quality of life and survival time were assessed. Fourteen patients received 8 cycles of transfusion and 6 received 4 cycles. No adverse effects were observed. The percentages of

CD3(+), CD3(+) CD8(+) and CD3(+) CD56(+) cells were significantly increased ($p < 0.05$), and the levels of serum beta2 microglobulin and lactate dehydrogenase (LDH) were markedly decreased ($p < 0.05$) after autologous CIK cell transfusion. Cancer-related symptoms were profoundly alleviated, as demonstrated by the improved quality of life ($p < 0.01$). Complete remission was observed in 11 patients, persistent partial remission in 7 patients and stable disease in 2 patients. At the end of follow-up, the mean survival time was 20 months. Transfusion with autologous CIK cells plus rhIL-2 treatment is safe and effective for treating haematological malignancies in elderly patients.

31 [293]. Zojer, N., K. Kirchbacher, et al. (2006). "Rituximab treatment provides no clinical benefit in patients with pretreated advanced multiple myeloma." Leuk Lymphoma **47**(6): 1103-1109.

In the present phase II study, we tested the efficacy of a single course of rituximab (375 mg/m2 on days 1, 8, 15 and 22) as treatment for relapsed myeloma. The rationale for this study was the identification of a population of clonotypic CD20+ B cells that are believed to be precursors of malignant plasma cells. In addition, CD20 was expressed on 10% and 50% of bone marrow plasma cells in two of the ten patients enrolled. Following rituximab treatment, none of the patients achieved an objective response. Two patients had stable disease at month 6, the predefined end of the study, while, at that time, two patients were classified as having progressive disease. One patient opted to withdraw from the study at month 3, at which time he had stable disease. The other five patients had to be withdrawn early from the post-treatment observation because of need of salvage therapy for progressive disease. WHO grade <or=2 toxicity
was seen in four patients. Peripheral B cells significantly decreased at 3 months, while no significant change of bone marrow myeloma cells was noted at that time. Mean paraprotein levels increased slightly during follow-up but IgM levels dropped in all patients, indicating an effective targeting of normal, short-lived plasma cells. Taken together, rituximab treatment yielded significant reductions in circulating B cells and serum IgM levels but had no beneficial clinical effect.

32 [237]. Zonder, J. A., A. F. Mohrbacher, et al. (2012). "A phase 1, multicenter, open-label, dose escalation study of elotuzumab in patients with advanced multiple myeloma." Blood **120**(3): 552-559.

This multicenter, first-in-human study evaluated the safety, tolerability, and pharmacokinetic and pharmacodynamic properties of the anti-CS1

monoclonal antibody elotuzumab. A standard 3 + 3 design was used to determine maximum tolerated dose; dose-limiting toxicities were assessed during cycle 1. Thirty-five patients with relapsed/refractory multiple myeloma were treated with intravenous elotuzumab at doses ranging from 0.5 to 20 mg/kg every 2 weeks. Patients who achieved at least stable disease after 4 treatments could receive another 4 treatments. No maximum tolerated dose was identified up to the maximum planned dose of 20 mg/kg. The most common adverse events, regardless of attribution, were cough, headache, back pain, fever, and chills. Adverse events were generally mild to moderate in severity, and adverse events attributed to study medication were primarily infusion-related. Plasma elotuzumab levels and terminal half-life increased with dose whereas clearance decreased, suggesting target-mediated clearance. CS1 on bone marrow-derived plasma cells was reliably saturated (>/= 95%) at the 10-mg/kg and 20-mg/kg dose levels. Using the European Group for Bone and Marrow Transplantation myeloma response criteria, 9 patients (26.5%) had stable disease. In summary, elotuzumab was generally well tolerated in this population, justifying further exploration of this agent in combination regimens.

TOPIC: Myeloma + checkpoint blockade or programmed death-1 (PD-1) or PD-L1 or B7-H1

1 [320]. Armand, P., A. Nagler, et al. (2013). "Disabling immune tolerance by programmed death-1 blockade with pidilizumab after autologous hematopoietic stem-cell transplantation for diffuse large B-cell lymphoma: results of an international phase II trial." J Clin Oncol **31**(33): 4199-4206.

PURPOSE: The Programmed Death-1 (PD-1) immune checkpoint pathway may be usurped by tumors, including diffuse large B-cell lymphoma (DLBCL), to evade immune surveillance. The reconstituting immune landscape after autologous hematopoietic stem-cell transplantation (AHSCT) may be particularly favorable for breaking immune tolerance through PD-1 blockade. PATIENTS AND METHODS: We conducted an international phase II study of pidilizumab, an anti-PD-1 monoclonal antibody, in patients with DLBCL undergoing AHSCT, with correlative studies of lymphocyte subsets. Patients received three doses of pidilizumab beginning 1 to 3 months after AHSCT. RESULTS: Sixty-six eligible patients were treated. Toxicity was mild. At 16 months after the first treatment, progression-free survival (PFS) was 0.72 (90% CI, 0.60 to 0.82), meeting the primary end point. Among the 24 high-risk patients who

remained positive on positron emission tomography after salvage chemotherapy, the 16-month PFS was 0.70 (90% CI, 0.51 to 0.82). Among the 35 patients with measurable disease after AHSCT, the overall response rate after pidilizumab treatment was 51%. Treatment was associated with increases in circulating lymphocyte subsets including PD-L1E-bearing lymphocytes, suggesting an on-target in vivo effect of pidilizumab. CONCLUSION: This is the first demonstration of clinical activity of PD-1 blockade in DLBCL. Given these results, PD-1 blockade after AHSCT using pidilizumab may represent a promising therapeutic strategy in this disease.

2 [368]. Berger, R., R. Rotem-Yehudar, et al. (2008). "Phase I safety and pharmacokinetic study of CT-011, a humanized antibody interacting with PD-1, in patients with advanced hematologic malignancies." Clin Cancer Res **14**(10): 3044-3051.

PURPOSE: CT-011 is a humanized IgG1 monoclonal antibody that modulates the immune response through interaction with PD-1, a protein belonging to the B7 receptor family present on lymphocytes. The objectives of this phase I study were to assess the dose-limiting toxicities, to determine the maximum tolerated dose, and to study the pharmacokinetics of CT-011 administered once to patients with advanced hematologic malignancies. EXPERIMENTAL DESIGN: Seventeen patients were treated with escalating doses of CT-011 ranging from 0.2 to 6 mg/kg. For pharmacokinetic analysis, blood samples were withdrawn from the patients before and immediately after treatment and at 24 hours, 48 hours, and on days 7, 14, and 21. CT-011 blood levels were assessed with a specific ELISA and derived concentrations were used to calculate pharmacokinetic parameters. Activation of the immune system was assessed by measuring peripheral blood CD4+, CD8+, and CD69+ lymphocytes. RESULTS: The study showed the antibody to be safe and well tolerated in this patient population. No single maximum tolerated dose was defined in this study. Clinical benefit was observed in 33% of the patients with one complete remission. Pharmacokinetic analyses show that serum Cmax and the AUC of CT-011 increased proportionally with dose.

The median t1/2 of CT-011 ranged from 217 to 410 hours. Sustained elevation in the percentage of peripheral blood CD4+ lymphocytes was observed up to 21 days following CT-011 treatment. CONCLUSIONS: A single administration of 0.2 to 6.0 mg/kg of CT-011 is safe and well tolerated in patients with advanced hematologic malignancies.

3 [357]. Brahmer, J. R., C. G. Drake, et al. (2010). "Phase I study of single-agent anti-programmed death-1 (MDX-1106) in refractory solid tumors: safety,

clinical activity, pharmacodynamics, and immunologic correlates." J Clin Oncol **28**(19): 3167-3175.

PURPOSE: Programmed death-1 (PD-1), an inhibitory receptor expressed on activated T cells, may suppress antitumor immunity. This phase I study sought to determine the safety and tolerability of anti-PD-1 blockade in patients with treatment-refractory solid tumors and to preliminarily assess antitumor activity, pharmacodynamics, and immunologic correlates. PATIENTS AND METHODS: Thirty-nine patients with advanced metastatic melanoma, colorectal cancer (CRC), castrate-resistant prostate cancer, non-small-cell lung cancer (NSCLC), or renal cell carcinoma (RCC) received a single intravenous infusion of anti-PD-1 (MDX-1106) in dose-escalating six-patient cohorts at 0.3, 1, 3, or 10 mg/kg, followed by a 15-patient expansion cohort at 10 mg/kg. Patients with evidence of clinical benefit at 3 months were eligible for repeated therapy. RESULTS: Anti-PD-1 was well tolerated: one serious adverse event, inflammatory colitis, was observed in a patient with melanoma who received five doses at 1 mg/kg. One durable complete response (CRC) and two partial responses (PRs; melanoma, RCC) were seen. Two additional patients (melanoma, NSCLC) had significant lesional tumor regressions not meeting PR criteria. The serum half-life of anti-PD-1 was 12 to 20 days. However, pharmacodynamics indicated a sustained mean occupancy of > 70% of PD-1 molecules on circulating T cells > or = 2 months following infusion, regardless of dose. In nine patients examined, tumor cell surface B7-H1 expression appeared to correlate with the likelihood of response to treatment. CONCLUSION: Blocking the PD-1 immune checkpoint with intermittent antibody dosing is well tolerated and associated with evidence of antitumor activity. Exploration of alternative dosing regimens and combinatorial therapies with vaccines, targeted therapies, and/or other checkpoint inhibitors is warranted.

4 [339]. Brahmer, J. R., S. S. Tykodi, et al. (2012). "Safety and activity of anti-PD-L1 antibody in patients with advanced cancer." N Engl J Med **366**(26): 2455-2465.

BACKGROUND: Programmed death 1 (PD-1) protein, a T-cell coinhibitory receptor, and one of its ligands, PD-L1, play a pivotal role in the ability of tumor cells to evade the host's immune system. Blockade of interactions between PD-1 and PD-L1 enhances immune function in vitro and mediates antitumor activity in preclinical models. METHODS: In this multicenter phase 1 trial, we administered intravenous anti-PD-L1 antibody (at escalating doses ranging from 0.3 to 10 mg per kilogram of body weight) to patients with selected advanced cancers. Anti-PD-L1 antibody

was administered every 14 days in 6-week cycles for up to 16 cycles or until the patient had a complete response or confirmed disease progression. RESULTS: As of February 24, 2012, a total of 207 patients--75 with non-small-cell lung cancer, 55 with melanoma, 18 with colorectal cancer, 17 with renal-cell cancer, 17 with ovarian cancer, 14 with pancreatic cancer, 7 with gastric cancer, and 4 with breast cancer--had received anti-PD-L1 antibody. The median duration of therapy was 12 weeks (range, 2 to 111). Grade 3 or 4 toxic effects that investigators considered to be related to treatment occurred in 9% of patients. Among patients with a response that could be evaluated, an objective response (a complete or partial response) was observed in 9 of 52 patients with melanoma, 2 of 17 with renal-cell cancer, 5 of 49 with non-small-cell lung cancer, and 1 of 17 with ovarian cancer. Responses lasted for 1 year or more in 8 of 16 patients with at least 1 year of follow-up. CONCLUSIONS: Antibody-mediated blockade of PD-L1 induced durable tumor regression (objective response rate of 6 to 17%) and prolonged stabilization of disease (rates of 12 to 41% at 24 weeks) in patients with advanced cancers, including non-small-cell lung cancer, melanoma, and renal-cell cancer. (Funded by Bristol-Myers Squibb and others; ClinicalTrials.gov number, NCT00729664.).

5 [377]. Feng, I. C., L. B. Koay, et al. (2007). "HBcAg-specific CD4+CD25+ regulatory T cells modulate immune tolerance and acute exacerbation on the natural history of chronic hepatitis B virus infection." J Biomed Sci **14**(1): 43-57.

Acute exacerbations (AEs) of chronic hepatitis B (CH-B) are accompanied by increased T cell responses to hepatitis B core and e antigens (HBcAg/HBeAg). Why patients are immunotolerant (IT) to the virus and why AEs occur spontaneously on the immunoactive phase remain unclear. The role of HBcAg-specific CD4(+)CD25(+) regulatory T (T(reg)) cells in AE and IT phases was investigated in this study. The SYFPEITHI scoring system was employed to predict MHC class II-restricted epitope peptides on HBcAg overlapping with HBeAg that were used for T(reg)-cell cloning and for the construction of MHC class II tetramers to measure T(reg) cell frequencies (T(reg) f). The results showed that HBcAg-specific T(reg) f declined during AE accompanied by increased HBcAg peptide-specific cytotoxic T lymphocyte frequencies. Predominant Foxp3-expressing T(reg) cell clones were generated from patients on the immune tolerance phase, while the majority of Th1 clones were obtained from patients on the immunoactive phase. T(reg) cells from liver and peripheral blood of CH-B patients express CD152 and PD1 antigens that

exhibit suppression on PBMCs proliferation to HBcAg. These data suggest that HBcAg peptide-specific T(reg) cells modulate the IT phase, and that their decline may account for the spontaneous AEs on the natural history of chronic hepatitis B virus infection.

6 [315]. Fourcade, J., Z. Sun, et al. (2014). "PD-1 and Tim-3 regulate the expansion of tumor antigen-specific CD8(+) T cells induced by melanoma vaccines." Cancer Res **74**(4): 1045-1055.

Although melanoma vaccines stimulate tumor antigen-specific CD8(+) T cells, objective clinical responses are rarely observed. To investigate this discrepancy, we evaluated the character of vaccine-induced CD8(+) T cells with regard to the inhibitory T-cell coreceptors PD-1 and Tim-3 in patients with metastatic melanoma who were administered tumor vaccines. The vaccines included incomplete Freund's adjuvant, CpG oligodeoxynucleotide (CpG), and the HLA-A2-restricted analog peptide NY-ESO-1 157-165V, either by itself or in combination with the pan-DR epitope NY-ESO-1 119-143. Both vaccines stimulated rapid tumor antigen-specific CD8(+) T-cell responses detected ex vivo, however, tumor antigen-specific CD8(+) T cells produced more IFN-gamma and exhibited higher lytic function upon immunization with MHC class I and class II epitopes. Notably, the vast majority of vaccine-induced CD8(+) T cells upregulated PD-1 and a minority also upregulated Tim-3. Levels of PD-1 and Tim-3 expression by vaccine-induced CD8(+) T cells at the time of vaccine administration correlated inversely with their expansion in vivo. Dual blockade of PD-1 and Tim-3 enhanced the expansion and cytokine production of vaccine-induced CD8(+) T cells in vitro. Collectively, our findings support the use of PD-1 and Tim-3 blockades with cancer vaccines to stimulate potent antitumor T-cell responses and increase the likelihood of clinical responses in patients with advanced melanoma.

7 [307]. Gros, A., P. F. Robbins, et al. (2014). "PD-1 identifies the patient-specific CD8(+) tumor-reactive repertoire infiltrating human tumors." J Clin Invest **124**(5): 2246-2259.

Adoptive transfer of tumor-infiltrating lymphocytes (TILs) can mediate regression of metastatic melanoma; however, TILs are a heterogeneous population, and there are no effective markers to specifically identify and select the repertoire of tumor-reactive and mutation-specific CD8(+) lymphocytes. The lack of biomarkers limits the ability to study these cells and develop strategies to enhance clinical efficacy and extend this therapy to other malignancies. Here, we evaluated unique phenotypic traits of CD8(+) TILs and TCR beta chain (TCRbeta) clonotypic frequency in

melanoma tumors to identify patient-specific repertoires of tumor-reactive CD8(+) lymphocytes. In all 6 tumors studied, expression of the inhibitory receptors programmed cell death 1 (PD-1; also known as CD279), lymphocyte-activation gene 3 (LAG-3; also known as CD223), and T cell immunoglobulin and mucin domain 3 (TIM-3) on CD8(+) TILs identified the autologous tumor-reactive repertoire, including mutated neoantigen-specific CD8(+) lymphocytes, whereas only a fraction of the tumor-reactive population expressed the costimulatory receptor 4-1BB (also known as CD137). TCRbeta deep sequencing revealed oligoclonal expansion of specific TCRbeta clonotypes in CD8(+)PD-1(+) compared with CD8(+)PD-1- TIL populations. Furthermore, the most highly expanded TCRbeta clonotypes in the CD8(+) and the CD8(+)PD-1(+) populations recognized the autologous tumor and included clonotypes targeting mutated antigens. Thus, in addition to the well-documented negative regulatory role of PD-1 in T cells, our findings demonstrate that PD-1 expression on CD8(+) TILs also accurately identifies the repertoire of clonally expanded tumor-reactive cells and reveal a dual importance of PD-1 expression in the tumor microenvironment.

8 [323]. Hamid, O., C. Robert, et al. (2013). "Safety and tumor responses with lambrolizumab (anti-PD-1) in melanoma." N Engl J Med **369**(2): 134-144.

BACKGROUND: The programmed death 1 (PD-1) receptor is a negative regulator of T-cell effector mechanisms that limits immune responses against cancer. We tested the anti-PD-1 antibody lambrolizumab (previously known as MK-3475) in patients with advanced melanoma. METHODS: We administered lambrolizumab intravenously at a dose of 10 mg per kilogram of body weight every 2 or 3 weeks or 2 mg per kilogram every 3 weeks in patients with advanced melanoma, both those who had received prior treatment with the immune checkpoint inhibitor ipilimumab and those who had not. Tumor responses were assessed every 12 weeks. RESULTS: A total of 135 patients with advanced melanoma were treated. Common adverse events attributed to treatment were fatigue, rash, pruritus, and diarrhea; most of the adverse events were low grade. The confirmed response rate across all dose cohorts, evaluated by central radiologic review according to the Response Evaluation Criteria in Solid Tumors (RECIST), version 1.1, was 38% (95% confidence interval [CI], 25 to 44), with the highest confirmed response rate observed in the cohort that received 10 mg per kilogram every 2 weeks (52%; 95% CI, 38 to 66). The response rate did not differ significantly between patients who had received prior ipilimumab treatment and those who had not (confirmed response rate, 38% [95% CI, 23 to 55] and 37% [95% CI, 26 to 49],

respectively). Responses were durable in the majority of patients (median follow-up, 11 months among patients who had a response); 81% of the patients who had a response (42 of 52) were still receiving treatment at the time of analysis in March 2013. The overall median progression-free survival among the 135 patients was longer than 7 months. CONCLUSIONS: In patients with advanced melanoma, including those who had had disease progression while they had been receiving ipilimumab, treatment with lambrolizumab resulted in a high rate of sustained tumor regression, with mainly grade 1 or 2 toxic effects. (Funded by Merck Sharp and Dohme; ClinicalTrials.gov number, NCT01295827.).

9 [311]. Jochems, C., J. A. Tucker, et al. (2014). "A combination trial of vaccine plus ipilimumab in metastatic castration-resistant prostate cancer patients: immune correlates." Cancer Immunol Immunother **63**(4): 407-418.

We recently reported the clinical results of a Phase I trial combining ipilimumab with a vaccine containing transgenes for prostate-specific antigen (PSA) and for a triad of costimulatory molecules (PROSTVAC) in patients with metastatic castration-resistant prostate cancer. Thirty patients were treated with escalating ipilimumab and a fixed dose of vaccine. Of 24 chemotherapy-naive patients, 58 % had a PSA decline. Combination therapy did not exacerbate the immune-related adverse events associated with ipilimumab. Here, we present updated survival data and an evaluation of 36 immune cell subsets pre- and post-therapy. Peripheral blood mononuclear cells were collected before therapy, at 13 days and at 70 days post-initiation of therapy, and phenotyped by flow cytometry for the subsets of T cells, regulatory T cells, natural killer cells, and myeloid-derived suppressor cells. Associations between overall survival (OS) and immune cell subsets prior to treatment, and the change in a given immune cell subset 70 days post-initiation of therapy, were evaluated. The median OS was 2.63 years (1.77-3.45). There were trends toward associations for longer OS and certain immune cell subsets before immunotherapy: lower PD-1(+)Tim-3(NEG)CD4EM (P = 0.005, adjusted P = 0.010), higher PD-1(NEG)Tim-3(+)CD8 (P = 0.002, adjusted P = 0.004), and a higher number of CTLA-4(NEG) Tregs (P = 0.005, adjusted P = 0.010). We also found that an increase in Tim-3(+) natural killer cells post- versus pre-vaccination associated with longer OS (P = 0.0074, adjusted P = 0.015). These results should be considered as hypothesis generating and should be further evaluated in larger immunotherapy trials.

10 [330]. Lipson, E. J., W. H. Sharfman, et al. (2013). "Durable cancer regression off-treatment and effective reinduction therapy with an anti-PD-1 antibody." Clin Cancer Res **19**(2): 462-468.

PURPOSE: Results from the first-in-human phase I trial of the anti-programmed death-1 (PD-1) antibody BMS-936558 in patients with treatment-refractory solid tumors, including safety, tolerability, pharmacodynamics, and immunologic correlates, have been previously reported. Here, we provide long-term follow-up on three patients from that trial who sustained objective tumor regressions off therapy, and test the hypothesis that reinduction therapy for late tumor recurrence can be effective. EXPERIMENTAL DESIGN: Three patients with colorectal cancer, renal cell cancer, and melanoma achieved objective responses on an intermittent dosing regimen of BMS-936558. Following cessation of therapy, patients were followed for more than 3 years. A patient with melanoma who experienced a prolonged partial regression followed by tumor recurrence received reinduction therapy. RESULTS: A patient with colorectal cancer experienced a complete response, which is ongoing after 3 years. A patient with renal cell cancer experienced a partial response lasting 3 years off therapy, which converted to a complete response, which is ongoing at 12 months. A patient with melanoma achieved a partial response that was stable for 16 months off therapy; recurrent disease was successfully treated with reinduction anti-PD-1 therapy. CONCLUSION: These data represent the most prolonged observation to date of patients with solid tumors responding to anti-PD-1 immunotherapy and the first report of successful reinduction therapy following delayed tumor progression. They underscore the potential for immune checkpoint blockade with anti-PD-1 to reset the equilibrium between tumor and the host immune system.

11 [380]. Reuben, J. M., B. N. Lee, et al. (2006). "Biologic and immunomodulatory events after CTLA-4 blockade with ticilimumab in patients with advanced malignant melanoma." Cancer **106**(11): 2437-2444.

BACKGROUND: T-regulatory (TR) cells expressing cytotoxic T lymphocyte-associated antigen-4 (CTLA-4) maintain peripheral immune tolerance and negatively affect host immune responses against cancer. The immunobiologic effects of ticilimumab, a human monoclonal antibody against CTLA-4, was administered to patients with metastatic melanoma who participated in a Phase I/II clinical trial. METHODS: Thirty patients who received ticilimumab at a dose of 10 mg/kg monthly (n=20) or 15 mg/kg every 3 months (n=10) were studied at study entry and at 14-day intervals thereafter to assess lymphocyte immunophenotypes, interleukin (IL)-2 and

IL-10 production, and the expression of TR-related genes in peripheral blood mononuclear cells (PBMC) from a subset of patients was studied by real-time polymerase chain reaction. RESULTS: Four of 12 patients with immune-related adverse events (IRAE) attained objective antitumor responses (ATR), whereas only 1 of 18 patients without IRAE attained ATR (chi2=4.0; P=.0455). Patients with ATR had significant reductions in T(R) cells and constitutive IL-10 production accompanied by a significant increase in IL-2 production by activated T cells. Although IRAE+/ATR+ patients demonstrated a positive correlation between CTLA-4 and glucocorticoid-induced tumor necrosis factor receptor (GITR) transcripts (Spearman rho=.522; P=.015), IRAE-/ATR- patients had a positive correlation between the transcripts of CTLA-4 and program death-1 (PD-1) receptor (Spearman rho=.891; P=.000). CONCLUSIONS: Antitumor responses in patients with metastatic melanoma who were treated with ticilimumab were found to be correlated with reductions in TR cells and constitutive secretion of IL-10, an increase in IL-2 production, and a positive correlation between transcripts of CTLA-4 and GITR. Conversely, a lack of ATR was found to be correlated with steady levels of TR cells and constitutive IL-10 secretion, and a positive correlation between the transcripts of CTLA-4 and PD-1.

12 [340]. Topalian, S. L., F. S. Hodi, et al. (2012). "Safety, activity, and immune correlates of anti-PD-1 antibody in cancer." N Engl J Med **366**(26): 2443-2454.

BACKGROUND: Blockade of programmed death 1 (PD-1), an inhibitory receptor expressed by T cells, can overcome immune resistance. We assessed the antitumor activity and safety of BMS-936558, an antibody that specifically blocks PD-1. METHODS: We enrolled patients with advanced melanoma, non-small-cell lung cancer, castration-resistant prostate cancer, or renal-cell or colorectal cancer to receive anti-PD-1 antibody at a dose of 0.1 to 10.0 mg per kilogram of body weight every 2 weeks. Response was assessed after each 8-week treatment cycle. Patients received up to 12 cycles until disease progression or a complete response occurred. RESULTS: A total of 296 patients received treatment through February 24, 2012. Grade 3 or 4 drug-related adverse events occurred in 14% of patients; there were three deaths from pulmonary toxicity. No maximum tolerated dose was defined. Adverse events consistent with immune-related causes were observed. Among 236 patients in whom response could be evaluated, objective responses (complete or partial responses) were observed in those with non-small-cell lung cancer, melanoma, or renal-cell cancer. Cumulative response rates (all doses) were 18% among patients with non-small-cell lung cancer (14 of 76 patients), 28% among patients with melanoma (26 of 94 patients),

and 27% among patients with renal-cell cancer (9 of 33 patients). Responses were durable; 20 of 31 responses lasted 1 year or more in patients with 1 year or more of follow-up. To assess the role of intratumoral PD-1 ligand (PD-L1) expression in the modulation of the PD-1-PD-L1 pathway, immunohistochemical analysis was performed on pretreatment tumor specimens obtained from 42 patients. Of 17 patients with PD-L1-negative tumors, none had an objective response; 9 of 25 patients (36%) with PD-L1-positive tumors had an objective response (P=0.006). CONCLUSIONS: Anti-PD-1 antibody produced objective responses in approximately one in four to one in five patients with non-small-cell lung cancer, melanoma, or renal-cell cancer; the adverse-event profile does not appear to preclude its use. Preliminary data suggest a relationship between PD-L1 expression on tumor cells and objective response. (Funded by Bristol-Myers Squibb and others; ClinicalTrials.gov number, NCT00730639.).

13 [310]. Topalian, S. L., M. Sznol, et al. (2014). "Survival, durable tumor remission, and long-term safety in patients with advanced melanoma receiving nivolumab." J Clin Oncol **32**(10): 1020-1030.

PURPOSE: Programmed cell death 1 (PD-1) is an inhibitory receptor expressed by activated T cells that downmodulates effector functions and limits the generation of immune memory. PD-1 blockade can mediate tumor regression in a substantial proportion of patients with melanoma, but it is not known whether this is associated with extended survival or maintenance of response after treatment is discontinued. PATIENTS AND METHODS: Patients with advanced melanoma (N = 107) enrolled between 2008 and 2012 received intravenous nivolumab in an outpatient setting every 2 weeks for up to 96 weeks and were observed for overall survival, long-term safety, and response duration after treatment discontinuation. RESULTS: Median overall survival in nivolumab-treated patients (62% with two to five prior systemic therapies) was 16.8 months, and 1- and 2-year survival rates were 62% and 43%, respectively. Among 33 patients with objective tumor regressions (31%), the Kaplan-Meier estimated median response duration was 2 years. Seventeen patients discontinued therapy for reasons other than disease progression, and 12 (71%) of 17 maintained responses off-therapy for at least 16 weeks (range, 16 to 56+ weeks). Objective response and toxicity rates were similar to those reported previously; in an extended analysis of all 306 patients treated on this trial (including those with other cancer types), exposure-adjusted toxicity rates were not cumulative. CONCLUSION: Overall survival following nivolumab treatment in patients with advanced treatment-refractory melanoma

compares favorably with that in literature studies of similar patient populations. Responses were durable and persisted after drug discontinuation. Long-term safety was acceptable. Ongoing randomized clinical trials will further assess the impact of nivolumab therapy on overall survival in patients with metastatic melanoma.

14 [319]. Weber, J. S., R. R. Kudchadkar, et al. (2013). "Safety, efficacy, and biomarkers of nivolumab with vaccine in ipilimumab-refractory or -naive melanoma." J Clin Oncol **31**(34): 4311-4318.

PURPOSE: Nivolumab, a human immunoglobulin G4-blocking antibody against the T-cell programmed death-1 checkpoint protein, has activity against metastatic melanoma. Its safety, clinical efficacy, and correlative biomarkers were assessed with or without a peptide vaccine in ipilimumab-refractory and -naive melanoma. PATIENTS AND METHODS: In this phase I study, 90 patients with unresectable stage III or IV melanoma who were ipilimumab naive and had experienced progression after at least one prior therapy (cohorts 1 to 3, 34 patients) or experienced progression after prior ipilimumab (cohorts 4 to 6, 56 patients) received nivolumab at 1, 3, or 10 mg/kg every 2 weeks for 24 weeks, then every 12 weeks for up to 2 years, with or without a multipeptide vaccine. RESULTS: Nivolumab with vaccine was well tolerated and safe at all doses. The RECIST 1.1 response rate for both ipilimumab-refractory and -naive patients was 25%. Median duration of response was not reached at a median of 8.1 months of follow-up. High pretreatment NY-ESO-1 and MART-1-specific CD8(+) T cells were associated with progression of disease. At week 12, increased peripheral-blood T regulatory cells and decreased antigen-specific T cells were associated with progression. PD-L1 tumor staining was associated with responses to nivolumab, but negative staining did not rule out a response. Patients who experienced progression after nivolumab could respond to ipilimumab. CONCLUSION: In patients with ipilimumab-refractory or -naive melanoma, nivolumab at 3 mg/kg with or without peptide vaccine was well tolerated and induced responses lasting up to 140 weeks. Responses to nivolumab in ipilimumab-refractory patients or to ipilimumab in nivolumab-refractory patients support combination or sequencing of nivolumab and ipilimumab.

15 [322]. Wolchok, J. D., H. Kluger, et al. (2013). "Nivolumab plus ipilimumab in advanced melanoma." N Engl J Med **369**(2): 122-133.

BACKGROUND: In patients with melanoma, ipilimumab (an antibody against cytotoxic T-lymphocyte-associated antigen 4 [CTLA-4]) prolongs overall survival, and nivolumab (an antibody against the programmed

death 1 [PD-1] receptor) produced durable tumor regression in a phase 1 trial. On the basis of their distinct immunologic mechanisms of action and supportive preclinical data, we conducted a phase 1 trial of nivolumab combined with ipilimumab in patients with advanced melanoma. METHODS: We administered intravenous doses of nivolumab and ipilimumab in patients every 3 weeks for 4 doses, followed by nivolumab alone every 3 weeks for 4 doses (concurrent regimen). The combined treatment was subsequently administered every 12 weeks for up to 8 doses. In a sequenced regimen, patients previously treated with ipilimumab received nivolumab every 2 weeks for up to 48 doses. RESULTS: A total of 53 patients received concurrent therapy with nivolumab and ipilimumab, and 33 received sequenced treatment. The objective-response rate (according to modified World Health Organization criteria) for all patients in the concurrent-regimen group was 40%. Evidence of clinical activity (conventional, unconfirmed, or immune-related response or stable disease for >/=24 weeks) was observed in 65% of patients. At the maximum doses that were associated with an acceptable level of adverse events (nivolumab at a dose of 1 mg per kilogram of body weight and ipilimumab at a dose of 3 mg per kilogram), 53% of patients had an objective response, all with tumor reduction of 80% or more. Grade 3 or 4 adverse events related to therapy occurred in 53% of patients in the concurrent-regimen group but were qualitatively similar to previous experience with monotherapy and were generally reversible. Among patients in the sequenced-regimen group, 18% had grade 3 or 4 adverse events related to therapy and the objective-response rate was 20%. CONCLUSIONS: Concurrent therapy with nivolumab and ipilimumab had a manageable safety profile and provided clinical activity that appears to be distinct from that in published data on monotherapy, with rapid and deep tumor regression in a substantial proportion of patients. (Funded by Bristol-Myers Squibb and Ono Pharmaceutical; ClinicalTrials.gov number, NCT01024231.).

TOPIC: Myeloma + oncolytic virus

1 [415]. Adachi, Y., N. Yoshio-Hoshino, et al. (2008). "Gene therapy for multiple myeloma." Curr Gene Ther **8**(4): 247-255.

Prognosis of multiple myeloma (MM) remains insufficient despite the intervention of high dose chemotherapy with auto- or allo- hematopoietic stem cell transplantation and the advent of molecular target drugs such as thalidomide, lenalidomide, and bortezomib. Further development or new concepts of therapeutic approaches are still required for MM

treatment. Current standard protocol for MM treatment does not include gene delivery method or oncolytic virus approaches. Since MM is a disorder originated from B cell lineage, it involves immunological aspects in both pathogenesis and clinical manifestations. Therefore, the comprehension of immunology as well as oncology is essential to exploit new therapeutic approaches. Recently, novel therapeutic concepts for MM have been emerging. In this review, we present current progress of gene therapy related to MM treatments as well as the overview of MM treatment history.

2 [390]. Ammayappan, A., K. W. Peng, et al. (2013). "Characteristics of oncolytic vesicular stomatitis virus displaying tumor-targeting ligands." J Virol **87**(24): 13543-13555.

We sought proof of principle that tumor-targeting ligands can be displayed on the surface of vesicular stomatitis virus (VSV) by engineering its glycoprotein. Here, we successfully rescued VSVs displaying tumor vasculature-targeting ligands. By using a rational approach, we investigated various feasible insertion sites on the G protein of VSV (VSV-G) for display of tumor vasculature-targeting ligands, cyclic RGD (cRGD) and echistatin. We found seven sites on VSV-G that tolerated insertion of the 9-residue cRGD peptide, two of which could tolerate insertion of the 49-amino acid echistatin domain. All of the ligand-displaying viruses replicated as well as the parental virus. In vitro studies demonstrated that the VSV-echistatin viruses specifically bound to targeted integrins. Since the low-density lipoprotein receptor (LDLR) was recently identified as a major receptor for VSV, we investigated the entry of ligand-displaying viruses after masking LDLR. The experiment showed that the modified viruses can enter the cell independently of LDLR, whereas entry of unmodified virus is significantly blocked by a specific monoclonal antibody against LDLR. Both parental and ligand-displaying viruses displayed equal oncolytic efficacies in a syngeneic mouse myeloma model. We further demonstrated that single-chain antibody fragments against tumor-specific antigens can be inserted at the N terminus of the G protein and that corresponding replication-competent VSVs can be rescued efficiently. Overall, we demonstrated that functional tumor-targeting ligands can be displayed on replication-competent VSVs without perturbing viral growth and oncolytic efficacy. This study provides a rational foundation for the future development of fully retargeted oncolytic VSVs.

3 [423]. Au, G. G., L. F. Lincz, et al. (2007). "Oncolytic Coxsackievirus A21 as a novel therapy for multiple myeloma." Br J Haematol **137**(2): 133-141.

Oncolytic viruses are attractive biological agents for the control of human malignancy. This study assessed the capacity of Coxsackievirus A21 (CVA21) to target and destroy multiple myeloma (MM) and precursor aberrant plasma cells in vitro using established MM cell lines and 15 patient bone marrow (BM) biopsies [n = 10 MM and five monoclonal gammopathy of undetermined significance (MGUS)]. Cell surface analysis revealed that all tumour cells lines expressed high levels of intercellular adhesion molecule-1 (ICAM-1) and decay-accelerating factor (DAF), the receptor molecules to which CVA21 can bind, leading to subsequent cell-entry and infection. MM cell lines were remarkably susceptible to CVA21 lytic infection, producing 100-1000-fold increases in viral progeny within 24 h. In contrast, normal peripheral blood cells were refractile to CVA21 infection. Furthermore, challenge of patient BM biopsies with CVA21 for 48 h resulted in specific purging of up to 98.7% of CD138+ plasma cells, with no significant decrease in progenitor cell function. Data generated in this study suggests that CVA21 virotherapy may have potential applications as a systemic anti-tumour agent for MM, or in the ex vivo purging of malignant plasma cells prior to autologous stem cell transplantation.

4 [394]. Ayala-Breton, C., L. Suksanpaisan, et al. (2013). "Amalgamating oncolytic viruses to enhance their safety, consolidate their killing mechanisms, and accelerate their spread." Mol Ther **21**(10): 1930-1937.

Oncolytic viruses are structurally and biologically diverse, spreading through tumors and killing them by various mechanisms and with different kinetics. Here, we created a hybrid vesicular stomatitis/measles virus (VSV/MV) that harnesses the safety of oncolytic MV, the speed of VSV, and the tumor killing mechanisms of both viruses. Oncolytic MV targets CD46 and kills by forcing infected cells to fuse with uninfected neighbors, but propagates slowly. VSV spreads rapidly, directly lysing tumor cells, but is neurotoxic and loses oncolytic potency when neuroattenuated by conventional approaches. The hybrid VSV/MV lacks neurotoxicity, replicates rapidly with VSV kinetics, and selectively targets CD46 on tumor cells. Its in vivo performance in a myeloma xenograft model was substantially superior to either MV or widely used recombinant oncolytic VSV-M51.

5 [392]. Bailey, K., A. Kirk, et al. (2013). "Mathematical model for radial expansion and conflation of intratumoral infectious centers predicts curative oncolytic virotherapy parameters." PLoS One **8**(9): e73759.

Simple, inductive mathematical models of oncolytic virotherapy are needed to guide protocol design and improve treatment outcomes. Analysis of plasmacytomas regressing after a single intravenous dose of oncolytic vesicular stomatitis virus in myeloma animal models revealed that intratumoral virus spread was spatially constrained, occurring almost exclusively through radial expansion of randomly distributed infectious centers. From these experimental observations we developed a simple model to calculate the probability of survival for any cell within a treated tumor. The model predicted that small changes to the density of initially infected cells or to the average maximum radius of infected centers would have a major impact on treatment outcome, and this was confirmed experimentally. The new model provides a useful and flexible tool for virotherapy protocol optimization.

6 [419]. Bajzer, Z., T. Carr, et al. (2008). "Modeling of cancer virotherapy with recombinant measles viruses." J Theor Biol **252**(1): 109-122.

The Edmonston vaccine strain of measles virus has potent and selective activity against a wide range of tumors. Tumor cells infected by this virus or genetically modified strains express viral proteins that allow them to fuse with neighboring cells to form syncytia that ultimately die. Moreover, infected cells may produce new virus particles that proceed to infect additional tumor cells. We present a model of tumor and virus interactions based on established biology and with proper accounting of the free virus population. The range of model parameters is estimated by fitting to available experimental data. The stability of equilibrium states corresponding to complete tumor eradication, therapy failure and partial tumor reduction is discussed. We use numerical simulations to explore conditions for which the model predicts successful therapy and tumor eradication. The model exhibits damped, as well as stable oscillations in a range of parameter values. These oscillatory states are organized by a Hopf bifurcation.

7 [401]. Bartee, E., W. M. Chan, et al. (2012). "Selective purging of human multiple myeloma cells from autologous stem cell transplantation grafts using oncolytic myxoma virus." Biol Blood Marrow Transplant **18**(10): 1540-1551.

Autologous stem cell transplantation and novel therapies have improved overall survival of patients with multiple myeloma; however, most patients relapse and eventually succumb to their disease. Evidence indicates that residual cancer cells contaminate autologous grafts and may contribute to early relapses after autologous stem cell transplantation. Here, we demonstrate that ex vivo treatment with an oncolytic poxvirus called

myxoma virus results in specific elimination of human myeloma cells by inducing rapid cellular apoptosis while fully sparing normal hematopoietic stem and progenitor cells. The specificity of this elimination is based on strong binding of the virus to myeloma cells coupled with an inability of the virus to bind or infect CD34(+) hematopoietic stem and progenitor cells. These 2 features allow myxoma to readily identify and distinguish even low levels of myeloma cells in complex mixtures. This ex vivo rabbit-specific oncolytic poxvirus called myxoma virus treatment also effectively inhibits systemic in vivo engraftment of human myeloma cells into immunodeficient mice and results in efficient elimination of primary CD138(+) myeloma cells contaminating patient hematopoietic cell products. We conclude that ex vivo myxoma treatment represents a safe and effective method to selectively eliminate myeloma cells from hematopoietic autografts before reinfusion.

8 [387]. Bell, J. C. (2014). "Taming measles virus to create an effective cancer therapeutic." Mayo Clin Proc **89**(7): 863-865.

9 [404]. Chen, C. Y., J. S. Senac, et al. (2011). "Species D adenoviruses as oncolytics against B-cell cancers." Clin Cancer Res **17**(21): 6712-6722.

PURPOSE: Oncolytic viruses are self-amplifying anticancer agents that make use of the natural ability of viruses to kill cells. Adenovirus serotype 5 (Ad5) has been extensively tested against solid cancers, but less so against B-cell cancers because these cells do not generally express the coxsackie and adenoviral receptor (CAR). To determine whether other adenoviruses might have better potency, we "mined" the adenovirus virome of 55 serotypes for viruses that could kill B-cell cancers. EXPERIMENTAL DESIGN: Fifteen adenoviruses selected to represent Ad species B, C, D, E, and F were tested in vitro against cell lines and primary patient B-cell cancers for their ability to infect, replicate in, and kill these cells. Select viruses were also tested against B-cell cancer xenografts in immunodeficient mice. RESULTS: Species D adenoviruses mediated most robust killing against a range of B-cell cancer cell lines, against primary patient marginal zone lymphoma cells, and against primary patient CD138+ myeloma cells in vitro. When injected into xenografts in vivo, single treatment with select species D viruses Ad26 and Ad45 delayed lymphoma growth. CONCLUSIONS: Relatively unstudied species D adenoviruses have a unique ability to infect and replicate in B-cell cancers as compared with

other adenovirus species. These data suggest these viruses have unique biology in B cells and support translation of novel species D adenoviruses as oncolytics against B-cell cancers.

10 [416]. Deng, H., N. Tang, et al. (2008). "Oncolytic virotherapy for multiple myeloma using a tumour-specific double-deleted vaccinia virus." Leukemia **22**(12): 2261-2264.

11 [428]. Dingli, D., M. D. Cascino, et al. (2006). "Mathematical modeling of cancer radiovirotherapy." Math Biosci **199**(1): 55-78.

Cancer virotherapy represents a dynamical system that requires mathematical modeling for complete understanding of the outcomes. The combination of virotherapy with radiation (radiovirotherapy) has been recently shown to successfully eliminate tumors when virotherapy alone failed. However, it introduces a new level of complexity. We have developed a mathematical model, based on population dynamics, that captures the essential elements of radiovirotherapy. The existence of corresponding equilibrium points related to complete cure, partial cure, and therapy failure is proved and discussed. The parameters of the model were estimated by fitting to experimental data. By using simulations we analyzed the influence of parameters that describe the interaction between virus and tumor cell on the outcome of the therapy. Furthermore, we evaluated relevant therapeutic scenarios for radiovirotherapy, and offered elements for optimization.

12 [411]. Dingli, D., C. Offord, et al. (2009). "Dynamics of multiple myeloma tumor therapy with a recombinant measles virus." Cancer Gene Ther **16**(12): 873-882.

Replication-competent viruses are being tested as tumor therapy agents. The fundamental premise of this therapy is the selective infection of the tumor cell population with the amplification of the virus. Spread of the virus in the tumor ultimately should lead to eradication of the cancer. Tumor virotherapy is unlike any other form of cancer therapy as the outcome depends on the dynamics that emerge from the interaction between the virus and tumor cell populations both of which change in time. We explore these interactions using a model that captures the salient biological features of this system in combination with in vivo data. Our results show that various therapeutic outcomes are possible ranging from tumor eradication to oscillatory behavior. Data from in vivo studies support these conclusions and validate our modeling approach. Such realistic models can be used to understand experimental observations,

explore alternative therapeutic scenarios and develop techniques to optimize therapy.

13 [430]. Dingli, D., K. W. Peng, et al. (2004). "Image-guided radiovirotherapy for multiple myeloma using a recombinant measles virus expressing the thyroidal sodium iodide symporter." Blood **103**(5): 1641-1646.

The Edmonston vaccine strain of measles virus (MV-Edm) propagates efficiently in a broad range of human tumor cells, killing them selectively. However, the oncolytic potency of MV-Edm in different human tumor xenograft therapy models is highly variable and there is no convenient way to map the distribution of virus-infected tissues in vivo. To enhance the oncolytic potency of MV-Edm against radiosensitive malignancies and to facilitate noninvasive imaging of infected tissues, we generated a recombinant MV-Edm encoding the human thyroidal iodide symporter (NIS). MV-NIS replicated almost as efficiently as unmodified MV-Edm, and human tumor cells efficiently concentrated radioiodine when infected with MV-NIS. Intratumoral spread of MV-NIS was noninvasively demonstrated by serial gamma-camera imaging of iodine-123 (123I) uptake both in MV-sensitive KAS-6/1 myeloma xenografts, which regressed completely after a single intravenous dose of MV-NIS, and in MM1 myeloma xenografts, which were unresponsive to MVNIS therapy. However, MV-resistant MM1 tumors regressed completely when 131I was administered 9 days after a single intravenous injection of MV-NIS (radiovirotherapy). 131I alone had no effect on MM1 tumor growth. While the potential hematopoietic toxicity of this new therapy requires further evaluation, image-guided radiovirotherapy is a promising new approach to the treatment of multiple myeloma, an incurable but highly radiosensitive plasma cell malignancy. Testing in other radiosensitive cancers is warranted.

14 [409]. Fernandes, M. S., E. M. Gomes, et al. (2009). "Growth inhibition of human multiple myeloma cells by an oncolytic adenovirus carrying the CD40 ligand transgene." Clin Cancer Res **15**(15): 4847-4856.

PURPOSE: The growth-inhibitory activity of recombinant CD40 ligand (CD40L) is well documented in human multiple myeloma (MM). We examined MM-targeted delivery of CD40L by a conditional replicative oncolytic adenovirus, AdEHCD40L. EXPERIMENTAL DESIGN: The growth-regulatory activity of AdEHCD40L was determined in vitro and in vivo. Differential analysis with AdEHCD40L and parental virus (AdEHNull)-infected cultures allowed the identification of cellular and molecular pathways modulated by the CD40L transgene. RESULTS:

Conditional expression of viral E1A and CD40L transgene was shown in human MM lines RPMI 8226 [interleukin (IL)-6 independent] and Kas-6/1 (IL-6 dependent) under hypoxic conditions commonly found in MM in situ. AdEHCD40L inhibited MM cell growth more effectively than AdEHNull. This enhanced growth-inhibitory activity was abrogated by cotreatment with a CD40L antibody. Chemoresistant MM lines (MR20 and LR5) were similarly susceptible to AdEHCD40L treatment. AdEHCD40L induced apoptosis and S-phase cell cycle blockade while uniquely up-regulating the previously described proapoptotic elements tumor necrosis factor-related apoptosis-inducing ligand, Fas, and IL-8. Intratumoral injections of AdEHCD40L reduced the growth of severe combined immunodeficient/hu RPMI 8226 xenografts by >50% compared with 28% reduction by AdEHNull. Adenoviral hexon and CD40L were detected in AdEHCD40L-treated tumors at day 35 after infection primarily in necrotic areas, suggesting viral replicative activity. CONCLUSIONS: These findings show that CD40L acts in concert with viral oncolysis to produce MM growth inhibition through activation of cellular apoptosis. The direct growth-inhibitory activity of AdEHCD40L, together with the well-known immune-potentiating features of CD40L, may be clinically applicable for the experimental treatment of MM or plasma cell leukemia.

15 [421]. Goel, A., S. K. Carlson, et al. (2007). "Radioiodide imaging and radiovirotherapy of multiple myeloma using VSV(Delta51)-NIS, an attenuated vesicular stomatitis virus encoding the sodium iodide symporter gene." Blood **110**(7): 2342-2350.

Multiple myeloma is a radiosensitive malignancy that is currently incurable. Here, we generated a novel recombinant vesicular stomatitis virus [VSV(Delta51)-NIS] that has a deletion of methionine 51 in the matrix protein and expresses the human sodium iodide symporter (NIS) gene. VSV(Delta51)-NIS showed specific oncolytic activity against myeloma cell lines and primary myeloma cells and was able to replicate to high titers in myeloma cells in vitro. Iodide uptake assays showed accumulation of radioactive iodide in VSV(Delta51)-NIS-infected myeloma cells that was specific to the function of the NIS transgene. In bg/nd/xid mice with established subcutaneous myeloma tumors, administration of VSV(Delta51)-NIS resulted in high intratumoral virus replication and tumor regression. VSV-associated neurotoxicity was not observed. Intratumoral spread of the infection was monitored noninvasively by serial gamma camera imaging of (123)I-iodide biodistribution. Dosimetry calculations based on these images pointed to the feasibility of combination radiovirotherapy with VSV(Delta51)-NIS plus (131)I. Immunocompetent

mice with syngeneic 5TGM1 myeloma tumors (either subcutaneous or orthotopic) showed significant enhancements of tumor regression and survival when VSV(Delta51)-NIS was combined with (131)I. These results show that VSV(Delta51)-NIS is a safe oncolytic agent with significant therapeutic potential in multiple myeloma.

16 [405]. Hadac, E. M., E. J. Kelly, et al. (2011). "Myeloma xenograft destruction by a nonviral vector delivering oncolytic infectious nucleic acid." Mol Ther **19**(6): 1041-1047.

The feasibility of using a nonviral vector formulation to initiate an oncolytic viral infection has not been previously demonstrated. We therefore sought to determine whether infectious nucleic acid (INA) could be used in place of virus particles to initiate an oncolytic picornavirus infection in vivo.

Infectious RNA encoding coxsackievirus A21 (CVA21) was transcribed from plasmid DNA using T7 polymerase. Within 48 hours of injecting this RNA into KAS6/1 myeloma xenografts, high titers of infectious CVA21 virions were detected in the bloodstream. Tumors regressed rapidly thereafter and mice developed signs of myositis. At euthanasia, CVA21 was recovered from regressing tumors and from skeletal muscles. Treatment outcomes were comparable following intratumoral injection of naked RNA or fully infectious CVA21 virus. Dose-response studies showed that an effective oncolytic infection could be established by intratumoral injection of 1 microg of infectious RNA. The oncolytic infection could also be initiated by intravenous injection of infectious RNA. Our study demonstrates that INA is a highly promising alternative drug formulation for oncolytic virotherapy.

17 [407]. Liu, C., S. J. Russell, et al. (2010). "Systemic therapy of disseminated myeloma in passively immunized mice using measles virus-infected cell carriers." Mol Ther **18**(6): 1155-1164.

Multiple myeloma (MM) is bone marrow plasma cell malignancy. A clinical trial utilizing intravenous administration of oncolytic measles virus (MV) encoding the human sodium-iodide symporter (MV-NIS) is ongoing in myeloma patients. However, intravenously administered MV-NIS is rapidly neutralized by antiviral antibodies. Because myeloma cell lines retain bone marrow tropism, they may be ideal as carriers for delivery of MV-NIS to myeloma deposits. A disseminated human myeloma (KAS 6/1) model was established. Biodistribution of MM1, a myeloma cell line, was determined after intravenous infusion. MM1 cells were found in the spine, femurs, and mandibles of tumor-bearing mice. Lethally irradiated MM1 cells remained susceptible to measles infection and transferred MV to KAS

6/1 cells in the presence of measles immune sera. Mice-bearing disseminated myeloma and passively immunized with measles immune serum were given MV-NIS or lethally irradiated MV-NIS-infected MM1 carriers. The antitumor activity of MV-NIS was evident only in measles naive mice and not in passively immunized mice. In contrast, survivals of both measles naive and immune mice were extended using MV-NIS-infected MM1 cell carriers. Hence, we demonstrate for the first time that systemically administered cells can serve as MV carriers and prolonged survival of mice with pre-existing antimeasles antibodies.

18 [395]. Liu, C., L. Suksanpaisan, et al. (2013). "Enhancing cytokine-induced killer cell therapy of multiple myeloma." Exp Hematol **41**(6): 508-517.

Cytokine-induced killer (CIK) cells are in clinical testing against various tumor types, including multiple myeloma. In this study, we show that CIK cells have activity against subcutaneous and disseminated models of human myeloma (KAS-6/1), which can be enhanced by infecting the CIK cells with an oncolytic measles virus (MV) or by pretreating the myeloma cells with ionizing radiation (XRT). KAS-6/1 cells were killed by coculture with CIK or MV-infected CIK (CIK/MV) cells, and the addition of an anti-NKG2D antibody inhibited cytolysis by 50%. However, human bone marrow stromal cells can reduce CIK and CIK/MV mediated killing of myeloma cells (RPMI 8226, JJN-3 and MM1). In vivo, CIK and CIK/MV prolonged the survival of mice with systemic myeloma, although CIK/MV showed enhanced antitumor activity compared with CIK. Irradiation of the KAS-6/1 cells induced mRNA and protein expression of NKG2D ligands, MICA, and MICB in a dose-dependent manner and enhanced delivery of CIK/MV to the irradiated tumors. In both subcutaneous and disseminated myeloma models, XRT at 2 Gy resulted in superior prolongation of the survival of mice given CIK/MV therapy compared with CIK/MV with no XRT. This study demonstrates the potential of CIK against myeloma and that the combination of virotherapy with radiation could be used to further enhance therapeutic outcome using CIK cells.

19 [403]. Liu, Y. P., C. Tong, et al. (2012). "Polyinosinic acid decreases sequestration and improves systemic therapy of measles virus." Cancer Gene Ther **19**(3): 202-211.

Off-target binding or vector sequestration can significantly limit the efficiency of systemic virotherapy. We report here that systemically administered oncolytic measles virus (MV) was rapidly sequestered by the mononuclear phagocytic system (MPS) of the liver and spleen in measles receptor CD46-positive and CD46-negative mice. Since scavenger

receptors on Kupffer cells are responsible for the elimination of blood-borne pathogens, we investigated here if MV uptake was mediated by scavenger receptors on Kupffer cells. Pretreatment of cells with poly(I), a scavenger receptor ligand, reduced MV expression by 99% in murine (J774A.1) macrophages and by 50% in human (THP-1) macrophages. Pre-dosing of mice with poly(I) reduced MPS sequestration of MV and increased circulating levels of MV by 4 to 15-folds at 2 min post virus administration. Circulating virus was still detectable 30 min post infusion in mice pre-dosed with poly(I) whereas no detectable MV was found at 5-10 min post infusion if mice did not receive poly(I). MPS blockade by poly(I) enhanced virus delivery to human ovarian SKOV3ip.1 and myeloma KAS6/1 xenografts in mice. Higher gene expression and improved control of tumor growth was noted early post therapy. Based on these results, incorporation of MPS blockade into MV treatment regimens is warranted.

20 [410]. Peng, K. W., A. Dogan, et al. (2009). "Tumor-associated macrophages infiltrate plasmacytomas and can serve as cell carriers for oncolytic measles virotherapy of disseminated myeloma." Am J Hematol **84**(7): 401-407.

In multiple myeloma, some of the neoplastic plasma cells are diffusely dispersed among the normal bone marrow cells (bone marrow resident), whereas others are located in discrete, well-vascularized solid tumors (plasmacytomas) that may originate in bone or soft tissue. Interactions between bone marrow-resident myeloma cells and bone marrow stromal cells (BMSCs) are important determinants of myeloma pathogenesis. However, little is known of the factors sustaining myeloma growth and cell viability at the centers of expanding plasmacytomas, where there are no BMSCs. Histologic sections of 22 plasmacytomas from myeloma patients were examined after immunostaining. Abundant CD68+, CD163+, S100-negative macrophage infiltrates were identified in all tumors, accompanied by scattered collections of CD3+ T lymphocytes. The CD68+ tumor-associated macrophages (TAM) accounted for 2-12% of nucleated cells and were evenly distributed through the parenchyma. The TAM generally had dendritic morphology, and each dendrite was in close contact with multiple plasma cells. In some cases, the TAM were strikingly clustered around CD34+ blood vessels. To determine whether cells of the monocytic lineage might be exploitable as carriers for delivery of therapeutic agents to plasmacytomas, primary human CD14+ cells were infected with oncolytic measles virus and administered intravenously to mice bearing KAS6/1 human myeloma xenografts. The cell carriers localized to KAS6/1 tumors, where they transferred MV infection to

myeloma cells and prolonged the survival of mice bearing disseminated human myeloma disease. Thus, TAM are a universal stromal component of the plasmacytomas of myeloma patients and may offer a promising new target for therapeutic exploitation. Am. J. Hematol. 2009. (c) 2009 Wiley-Liss, Inc.

21 [388]. Russell, S. J., M. J. Federspiel, et al. (2014). "Remission of disseminated cancer after systemic oncolytic virotherapy." Mayo Clin Proc **89**(7): 926-933.

MV-NIS is an engineered measles virus that is selectively destructive to myeloma plasma cells and can be monitored by noninvasive radioiodine imaging of NIS gene expression. Two measles-seronegative patients with relapsing drug-refractory myeloma and multiple glucose-avid plasmacytomas were treated by intravenous infusion of 10(11) TCID50 (50% tissue culture infectious dose) infectious units of MV-NIS. Both patients responded to therapy with M protein reduction and resolution of bone marrow plasmacytosis. Further, one patient experienced durable complete remission at all disease sites. Tumor targeting was clearly documented by NIS-mediated radioiodine uptake in virus-infected plasmacytomas. Toxicities resolved within the first week after therapy. Oncolytic viruses offer a promising new modality for the targeted infection and destruction of disseminated cancer.

22 [408]. Senac, J. S., K. Doronin, et al. (2010). "Infection and killing of multiple myeloma by adenoviruses." Hum Gene Ther **21**(2): 179-190.

Oncolytic virotherapy makes use of the natural ability of viruses to infect and kill cancer cells. Adenovirus serotype 5 (Ad5) has been approved for use in humans as a therapy for solid cancers. In this study, we have tested whether Ad5 and low-seroprevalence adenoviruses can be used as oncolytics for multiple myeloma (MM). We show that Ad5 productively infects most myeloma cell lines, replicates to various degrees, and mediates oncolytic cell killing in vitro and in vivo. Comparison of Ad5 with low-seroprevalence Ads on primary marrow samples from MM patients revealed striking differences in the abilities of different adenoviral serotypes to kill normal CD138(-) cells and CD138(+) MM cells. Ad5 and Ad6 from species C and Ad26 and Ad48 from species D all mediated killing of

CD138(+) cells with low-level killing of CD138(-) cells. In contrast, Ad11, Ad35, Ad40, and Ad41 mediated weak oncolytic effects in all of the cells. Comparison of cell binding, cell entry, and replication revealed that Ad11 and Ad35 bound MM cells 10 to 100 times better than other serotypes. However, after this efficient interaction, Ad11 and Ad35 viral DNA was not

replicated and cell killing did not occur. In contrast, Ad5, Ad6, Ad26, and Ad48 all replicated 10- to 100-fold in MM cells and this correlated with cell killing. These data suggest that Ad5 and other low-seroprevalence adenoviruses may have utility as oncolytic agents against MM and other hematologic malignancies.

23 [393]. Thirukkumaran, C. M., Z. Q. Shi, et al. (2014). "Reovirus as a successful ex vivo purging modality for multiple myeloma." Bone Marrow Transplant **49**(1): 80-86.

Autologous stem cell rescue (ASCT) following high-dose myeloablative chemotherapy is considered to be a therapeutic option for many multiple myeloma (MM) patients; however relapse post ASCT presents a major challenge. The oncolytic potential of reovirus has been previously demonstrated and is currently undergoing phase I monotherapy clinical trials for MM and phase II/III clinical trials for solid tumors. Here we tested the hypothesis that reovirus can successfully purge MM in a murine model that partially recapitulates human MM. RPMI 8226, MM1S, H929 and U266 human myeloma cell lines were exposed to reovirus and oncolysis was assessed. Apheresis product admixed with MM cells was purged with live reovirus (LV) or dead virus (DV) and purging efficacy was monitored via flow cytometry, reverse transcribed-PCR (RT-PCR) and disease relapse in non obese diabetic/severe combined immune deficient (NOD/SCID) mice. Significant LV purging was seen with MM1S, H929 and U266 and the complete ex vivo purging achieved with RPMI 8226 was confirmed by flow cytometry, RT-PCR and absence of disease relapse in vivo. Mice that received LV-purged autografts exhibited 100% survival in comparison to mice that received DV-purged controls. Reovirus's unique ability to kill MM while sparing hematopoietic stem cells places it as an attractive purging agent for MM during ASCT.

24 [399]. Thirukkumaran, C. M., Z. Q. Shi, et al. (2012). "Reovirus as a viable therapeutic option for the treatment of multiple myeloma." Clin Cancer Res **18**(18): 4962-4972.

PURPOSE: Despite the recent advances made in the treatment of multiple myeloma, the disease still remains incurable. The oncolytic potential of reovirus has previously been shown and is currently in phase III clinical trials for solid tumors. We tested the hypothesis that reovirus can successfully target human multiple myeloma in vitro, ex vivo, and in vivo without affecting human hematopoietic stem cell (HHSC) re-population/differentiation in a murine model that partially recapitulates human multiple myeloma. EXPERIMENTAL DESIGN: Human myeloma cell

lines and ex vivo tumor specimens were exposed to reovirus and oncolysis and mechanisms of cell death were assessed. RPMI 8226(GFP+) cells were injected intravenously to non-obese diabetic/severe combined immune deficient (NOD/SCID) mice and treated with live reovirus (LV) or dead virus
(DV). Multiple myeloma disease progression was evaluated via whole-body fluorescence and bone marrow infiltration. HHSCs exposed to LV/DV were injected to NOD/SCID mice and re-population/differentiation was monitored. RESULTS: A total of six of seven myeloma cell lines and five of seven patient tumor specimens exposed to reovirus showed significant in vitro sensitivity. Tumor response of multiple myeloma by LV, but not DV, was confirmed by comparison of total tumor weights (P = 0.05), and bone marrow infiltration (1/6, LV; 5/6, DV). Mice injected with LV- or DV-exposed HHSCs maintained in vivo re-population/lineage differentiation showing a lack of viral effect on the stem cell compartment. Reovirus oncolysis was mediated primarily by activation of the apoptotic pathways. CONCLUSIONS: The unique ability of reovirus to selectively kill multiple myeloma while sparing HHSCs places it as a promising systemic multiple myeloma therapeutic for clinical testing.

TOPIC: Myeloma + virotherapy

1 [441]. Thirukkumaran, C. M., Z. Q. Shi, et al. (2013). "Reovirus modulates autophagy during oncolysis of multiple myeloma." Autophagy **9**(3): 413-414.

Multiple myeloma (MM) is a clonal plasma cell malignancy that accounts for 10-15% of newly diagnosed hematological cancers. Although significant advances have been made in the treatment of MM the disease
still remains incurable. The oncolytic potential of reovirus has previously been demonstrated by others and us and is currently in phase III clinical trials for solid tumors. In addition a phase I clinical trial has recently been initiated for MM. Despite the clinical activity, the mechanism(s) of cell death caused by reovirus in MM is yet not yet well elucidated. A comprehensive understanding of reovirus-mediated histology-specific cell death mechanisms is imperative if this therapeutic is to become a standard of care for patients. Previously we have shown that reovirus-mediated cell death of breast and prostate cancer is orchestrated via apoptosis. The present study demonstrates for the first time that in addition to inducing apoptosis reovirus also upregulates autophagy during oncolysis of MM.

TOPIC: Myeloma + dendritic cell vaccine or idiotype vaccine

1 [492]. Bendandi, M., M. Rodriguez-Calvillo, et al. (2006). "Combined vaccination with idiotype-pulsed allogeneic dendritic cells and soluble protein idiotype for multiple myeloma patients relapsing after reduced-intensity conditioning allogeneic stem cell transplantation." Leuk Lymphoma **47**(1): 29-37.

BACKGROUND AND OBJECTIVE: To combine the use of idiotype-pulsed allogeneic dendritic cells (alloDC) and soluble protein Id conjugated with KLH (Id-KLH) in a vaccine strategy for multiple myeloma (MM). DESIGN AND METHODS: Four MM patients received the combined vaccine after having experienced disease relapse/progression following reduced intensity conditioning (RIC) allogeneic stem cell transplantation (alloSCT) and failure to rescue therapy with donor lymphocyte infusion or chemotherapy (CHT). RESULTS: Vaccination was well tolerated and induced an anti-KLH antibody response in all 4 patients as well as substantial cell proliferation. In contrast, no case showed similar effects against either tumor-specific Id or irrelevant isotype control immunoglobulins (Ig). In turn, vaccination was associated with modulation of biological responses linked to both inflammatory and T-cell activation, with secretion of effector Th1 cytokines. In particular, an important increase in the spontaneous ex vivo secretion of TNFalpha, IL-6 and IFNgamma as well as IL-2 and IL-10 was frequently observed prior to the fourth vaccination. Moreover, in vitro stimulation with Id-KLH and Id-KLH plus alloDC, but not with alloDC alone was associated with an enhanced number of TNF-alpha+ T-cells and an increased secretion of IFNgamma and IL-2 before the third and fourth vaccination. From a clinical standpoint, 2 patients had a transient response and 1 has stable disease after stopping vaccination, while 3 of them ultimately progressed. INTERPRETATION AND CONCLUSIONS: The results show for the first time that the use of Id-pulsed alloDC following RIC alloSCT is safe and feasible. However, crucial strategy improvements are warranted to possibly achieve clinical benefit.

2 [482]. Curti, A., P. Tosi, et al. (2007). "Phase I/II clinical trial of sequential subcutaneous and intravenous delivery of dendritic cell vaccination for refractory multiple myeloma using patient-specific tumour idiotype protein or idiotype (VDJ)-derived class I-restricted peptides." Br J Haematol **139**(3): 415-424.

Fifteen multiple myeloma (MM) patients who had failed maintenance therapy after tandem autologous stem cell transplantation underwent anti-idiotype (Id) vaccination with dendritic cells (DCs). CD14(+)-derived DCs were loaded with the autologous Id as whole protein (=6) or

Id-derived class I-restricted peptides (=9) and keyhole limpet hemocyanin (KLH). Vaccination consisted of three subcutaneous (sc) and two intravenous injections of increasing DC doses at 2 weeks interval. DC therapy was well tolerated. Most patients developed both humoral and T-cell responses to KLH, suggesting immunocompetence. Eight of 15 patients developed an Id-specific T-cell proliferative response, 8/15 increased interferon-gamma-secreting T cells and 4/15 showed an Id-positive delayed-type hypersensitivity test. Anti-Id cytotoxic T-lymphocyte precursors increased after DC vaccination in 2/2 evaluable patients. A more robust T-cell response was observed after sc DC injections and increased Id-specific T-cell proliferation was found up to 1 year after vaccination. VDJ-derived peptides were as effective as the whole protein in stimulating T-cell responses. Clinically, 7/15 patients have stable disease after a median follow-up of 26 months, one patient achieved durable partial remission after 40 months, and seven patients progressed. In conclusion, sc injections of cryopreserved Id-pulsed DCs were safe and, in contrast with intravenous administrations, induced anti-MM T-cell responses.

3 [476]. Freedman, A., S. S. Neelapu, et al. (2009). "Placebo-controlled phase III trial of patient-specific immunotherapy with mitumprotimut-T and granulocyte-macrophage colony-stimulating factor after rituximab in patients with follicular lymphoma." J Clin Oncol **27**(18): 3036-3043.

PURPOSE: To evaluate patient-specific immunotherapy with mitumprotimut-T (idiotype keyhole limpet hemocyanin [Id-KLH]) and granulocyte-macrophage colony-stimulating factor (GM-CSF) in CD20(+) follicular lymphoma. PATIENTS AND METHODS: Patients with treatment-naive or relapsed/refractory disease achieving a complete response (CR), partial response (PR), or stable disease (SD) with four weekly rituximab infusions were randomly assigned to mitumprotimut-T/GM-CSF or placebo/GM-CSF, with doses given monthly for six doses, every 2 months for six doses, and then every 3 months until disease progression (PD). Randomization was stratified by prior therapy (treatment-naive or relapsed/refractory) and response to rituximab (CR/PR or SD). The primary end point was time to progression (TTP) from randomization. RESULTS: A total of 349 patients were randomly assigned; median age was 54 years, 79% were treatment naive, and 86% had stage III/IV disease. Median TTP was 9.0 months for mitumprotimut-T/GM-CSF and 12.6 months for placebo/GM-CSF (hazard ratio [HR] = 1.384; P = .019). TTP was comparable between the two arms in treatment-naive patients (HR = 1.196; P = .258) and shorter with mitumprotimut-T/GM-CSF in

relapsed/refractory disease (HR = 2.265; P = .004). After adjusting for Follicular Lymphoma International Prognostic Index (FLIPI) scores, the difference in TTP between the two arms was no longer significant. Overall objective response rate, rate of response improvement, and duration of response were comparable between the two arms. Toxicity was similar in the two arms; 76% of adverse events were mild or moderate, and 94% of patients had injection site reactions. CONCLUSION: TTP was shorter with mitumprotimut-T/GM-CSF compared with placebo/GM-CSF. This difference was possibly due to the imbalance in FLIPI scores.

4 [484]. Hansson, L., A. O. Abdalla, et al. (2007). "Long-term idiotype vaccination combined with interleukin-12 (IL-12), or IL-12 and granulocyte macrophage colony-stimulating factor, in early-stage multiple myeloma patients." Clin Cancer Res **13**(5): 1503-1510.

PURPOSE AND EXPERIMENTAL DESIGN: Twenty-eight patients with immunoglobulin G myeloma stages I to II were immunized i.d. over 110 weeks with autologous M protein combined with interleukin-12 (IL-12; n = 15) or with IL-12 and granulocyte macrophage colony-stimulating factor (GM-CSF; n = 13). Idiotype-specific T-cell responses were assessed by [(3)H]thymidine incorporation, enzyme-linked immunospot assay, and delayed-type hypersensitivity reaction. RESULTS: Based on these three assays, idiotype-specific immune responses were noted in 5 of 15 (33%) patients in the IL-12 group and in 11 of 13 (85%) patients in the GM-CSF/IL-12 group (P < 0.01). Immune response was seen only in patients with M-component concentration of <50 g/L. Three of 16 (19%) responders showed a gradually increasing idiotype-specific T-cell response, whereas 11 of 16 (69%) patients showed initial response, which then disappeared rapidly; the latter pattern was frequently associated with subsequent progressive disease. Immune nonresponse was associated with an increase in the numbers of CD4(+)/CD25(+) cells (regulatory T cells), which was absent in responding patients. Median time to progression for immune responders (n = 16) was 108 weeks compared with 26 weeks for nonresponders (n = 12; P = 0.03). CONCLUSIONS: These results indicate that idiotype immunization of myeloma patients with GM-CSF and IL-12 may induce specific T-cell response more frequently than with IL-12 alone and that immune response may correlate with time to progression and nonresponse with increased numbers of regulatory T cells.

5 [466]. Hobo, W., L. Strobbe, et al. (2013). "Immunogenicity of dendritic cells pulsed with MAGE3, Survivin and B-cell maturation antigen mRNA for

vaccination of multiple myeloma patients." Cancer Immunol Immunother **62**(8): 1381-1392.

The introduction of autologous stem cell transplantation (SCT) and novel drugs has improved overall survival in multiple myeloma (MM) patients. However, minimal residual disease (MRD) remains and most patients eventually relapse. Myeloma plasma cells express tumor-associated antigens (TAA), which are interesting targets for immunotherapy. In this phase 1 study, we investigated the safety and immunological effects of TAA-mRNA-loaded dendritic cell (DC) vaccination for treatment for MRD in MM after SCT. Mature monocyte-derived DCs were pulsed with keyhole limpet hemocyanin (KLH) and electroporated with MAGE3, Survivin or B-cell maturation antigen (BCMA) mRNA. Twelve patients were vaccinated three times with intravenous (5-22 x 10(6) DCs) and intradermal vaccines (4-11 x 10(6) DCs), at biweekly intervals. Immunological responses were monitored in blood and delayed-type hypersensitivity (DTH) biopsies. All patients developed strong anti-KLH T-cell responses, but not KLH antibodies. In 2 patients, vaccine-specific T cells were detected in DTH biopsies. In one patient, we found MAGE3-specific CD4(+) and CD8(+) T cells, and CD3(+) T cells reactive against BCMA and Survivin. In the other patient, we detected low numbers of MAGE3 and BCMA-reactive CD8(+) T cells. Vaccination was well tolerated with limited toxicity. These findings illustrate that TAA-mRNA-electroporated mature DCs are capable of inducing TAA-T-cell responses in MM patients after SCT.

6 [487]. Inoges, S., M. Rodriguez-Calvillo, et al. (2006). "Clinical benefit associated with idiotypic vaccination in patients with follicular lymphoma." J Natl Cancer Inst **98**(18): 1292-1301.

BACKGROUND: Follicular lymphoma is considered incurable, although cyclophosphamide, doxorubicin, vincristine, and prednisone (CHOP) chemotherapy can induce sequential remissions. A patient's second complete response is typically shorter than that patient's first complete response. Idiotype vaccines can elicit specific immune responses and molecular remissions in patients with follicular lymphoma. However, a clinical benefit has never been formally proven. METHODS: Thirty-three consecutive follicular lymphoma patients in first relapse received six monthly cycles of CHOP-like chemotherapy. Patients who achieved a second complete response were vaccinated periodically for more than 2 years with autologous lymphoma-derived idiotype protein vaccine. Specific humoral and cellular responses were assessed, and patients were followed for disease recurrence. Statistical tests were two-sided. RESULTS:

Idiotype vaccine could be produced for 25 patients who had a second complete response. In 20 patients (80%), a humoral (13/20) and/or a cellular (18/20) idiotype-specific response was detected. The median duration of the second complete response has not been reached, but it exceeds 33 months (range = 20+ to 51+ months). None of the 20 responders relapsed while undergoing active vaccination. All responders with enough follow-up for the comparison to be made experienced a second complete response that was statistically significantly (P<.0001) longer than both their first complete response (18 of 18 patients) and than the median duration of a CHOP-induced second complete response, i.e., 13 months (20 of 20 patients). The five nonresponders all had a second complete response that was shorter (median = 10 months; range = 8-13 months) than their first complete response (median = 17 months; range = 10-39 months). CONCLUSIONS: Idiotypic vaccination induced a specific immune response in the majority of patients with follicular lymphoma. Specific immune response was associated with a dramatic and highly statistically significant increase in disease-free survival. This is the first formal demonstration of clinical benefit associated with the use of a human cancer vaccine.

7 [474]. Lacy, M. Q., S. Mandrekar, et al. (2009). "Idiotype-pulsed antigen-presenting cells following autologous transplantation for multiple myeloma may be associated with prolonged survival." Am J Hematol **84**(12): 799-802.

Vaccines are attractive as consolidation therapy after autologous stem cell transplantation (ASCT) for multiple myeloma (MM). We report the results of a phase II trial of the immunotherapeutic, APC8020 (Mylovenge), given after ASCT for MM. We compared the results with that of other patients with MM who underwent ASCT at Mayo Clinic during the same time period. Twenty-seven patients were enrolled on the trial between July, 1998 and June, 2001, and the outcomes were compared to that of 124 consecutive patients transplanted during the same period, but not enrolled on the trial. The median (range) follow-up for patients still alive from the vaccine trial is 6.5 (2.9-8 years), and 7.1 (6-8 years) in the control group. The median age was 57.4 range (36.1-71.3) in the DB group and 56.4 (range, 30-69) in the trial group. Known prognostic factors including PCLI, B2M, and CRP were comparable between the groups. The median overall survival for the trial patients was 5.3 years (95% CI: 4.0 years-N/A) compared to 3.4 years (95% CI: 2.7-4.6 years) for the DB group (P = 0.02). The median time to progression and progression-free survival for the trial group was similar to the DB group. Although not a controlled trial, the

vaccines given after ASCT appear to be associated with improved overall survival compared to historical controls. This approach warrants further investigation to confirm this and define the role of vaccine therapy in myeloma.

8 [465]. Levy, R., K. N. Ganjoo, et al. (2014). "Active idiotypic vaccination versus control immunotherapy for follicular lymphoma." J Clin Oncol **32**(17): 1797-1803.

PURPOSE: Idiotypes (Ids), the unique portions of tumor immunoglobulins, can serve as targets for passive and active immunotherapies for lymphoma. We performed a multicenter, randomized trial comparing a specific vaccine (MyVax), comprising Id chemically coupled to keyhole limpet hemocyanin (KLH) plus granulocyte macrophage colony-stimulating factor (GM-CSF) to a control immunotherapy with KLH plus GM-CSF. PATIENTS AND METHODS: Patients with previously untreated advanced-stage follicular lymphoma (FL) received eight cycles of chemotherapy with cyclophosphamide, vincristine, and prednisone. Those achieving sustained partial or complete remission (n=287 [44%]) were randomly assigned at a ratio of 2:1 to receive one injection per month for 7 months of MyVax or control immunotherapy. Anti-Id antibody responses (humoral immune responses [IRs]) were measured before each immunization. The primary end point was progression-free survival (PFS). Secondary end points included IR and time to subsequent antilymphoma therapy. RESULTS: At a median follow-up of 58 months, no significant difference was observed in either PFS or time to next therapy between the two arms. In the MyVax group (n=195), anti-Id IRs were observed in 41% of patients, with a median PFS of 40 months, significantly exceeding the median PFS observed in patients without such Id-induced IRs and in those receiving control immunotherapy. CONCLUSION: This trial failed to demonstrate clinical benefit of specific immunotherapy. The subset of vaccinated patients mounting specific anti-Id responses had superior outcomes. Whether this reflects a therapeutic benefit or is a marker for more favorable underlying prognosis requires further study.

9 [471]. Navarrete, M. A., K. Heining-Mikesch, et al. (2011). "Upfront immunization with autologous recombinant idiotype Fab fragment without prior cytoreduction in indolent B-cell lymphoma." Blood **117**(5): 1483-1491.

Idiotype vaccination for follicular lymphoma is primarily being developed as remission consolidation after chemotherapy. We investigated idiotype vaccination as primary intervention for treatment-naive indolent B-cell lymphoma and in a separate cohort as remission consolidation after

chemotherapy to assess immunization-induced immune responses in relation to progression-free survival (German Clinical Trials Register, DRKS00000227). Twenty-one patients in each cohort received 6 intradermal injections of adjuvanted recombinant idiotype Fab fragment (Fab(Id)); 76% of patients in both groups developed anti-idiotype antibodies and/or cellular immunity as measured by enzyme-linked immunosorbent assay and interferon-gamma ELISpot. In treatment-naive patients, only cellular responses correlated with superior progression-free survival ($P < .002$) and durable objective remissions ($P = .04$). Immunization-induced T cells recognized hypermutated or complementarity-determining region 3 epitopes. After remission consolidation immunization, induction of anti-idiotype antibodies correlated with progression-free survival. Low B-cell counts after rituximab therapy predicted for failure to develop anti-idiotype antibodies. These results are similar to published trials showing an association of humoral immunity with control of residual lymphoma. In contrast, effective immunity against untreated lymphoma appears to be dependent on idiotype-specific T cells. Sustained remissions in patients with vaccination-induced cellular immunity suggest clinical benefit and warrant a randomized comparison of this vaccine with expectant management for asymptomatic follicular lymphoma.

10 [470]. Rollig, C., C. Schmidt, et al. (2011). "Induction of cellular immune responses in patients with stage-I multiple myeloma after vaccination with autologous idiotype-pulsed dendritic cells." J Immunother **34**(1): 100-106.

Idiotype vaccines have shown both biological efficacy and clinical benefit in lymphoma. Circulating idiotype proteins (Id) in multiple myeloma patients offer a suitable target for immunotherapy. So far, specific immune responses after vaccination with Ids have been evaluated mostly in advanced myeloma. We explored the potential of dendritic-cell (DC)-based immunotherapy in 9 patients with stage-I disease. Mature monocyte-derived Id-pulsed DCs and keyhole limpet hemocyanin (KLH) were administered at dose levels between 2 and 20x10(6) cells. Patients received 5 immunizations every 4 weeks. A median number of 6.8x10(6) DCs were administered per vaccination. Five out of 9 patients (56%) developed Id-specific T cells as showed in proliferation assays and 8 out of 9 patients (89%) showed specific T-cell-mediated cytokine release after Id stimulation. The cytokine-secretion did not show a distinct Th1-type or Th2-type pattern. The M protein dropped slightly in 3 out of 9 patients. We could show that DC-based Id vaccination is a feasible way of inducing specific T-cell responses in stage-I myeloma patients. Further trials are needed to increase the rate of responses and to

define the role of DC-based vaccination in the era of new pharmacologic therapies.

11 [472]. Rosenblatt, J., B. Vasir, et al. (2011). "Vaccination with dendritic cell/tumor fusion cells results in cellular and humoral antitumor immune responses in patients with multiple myeloma." Blood **117**(2): 393-402.

We have developed a tumor vaccine in which patient-derived myeloma cells are chemically fused with autologous dendritic cells (DCs) such that a broad spectrum of myeloma-associated antigens are presented in the context of DC-mediated costimulation. We have completed a phase 1 study in which patients with multiple myeloma underwent serial vaccination with the DC/multiple myeloma fusions in conjunction with granulocyte-macrophage colony-stimulating factor. DCs were generated from adherent mononuclear cells cultured with granulocyte-macrophage colony-stimulating factor, interleukin-4, and tumor necrosis factor-alpha and fused with myeloma cells obtained from marrow aspirates. Vaccine generation was successful in 17 of 18 patients. Successive cohorts were treated with 1 x 10(6), 2 x 10(6), and 4 x 10(6) fusion cells, respectively, with 10 patients treated at the highest dose level. Vaccination was well tolerated, without evidence of dose-limiting toxicity. Vaccination resulted in the expansion of circulating CD4 and CD8 lymphocytes reactive with autologous myeloma cells in 11 of 15 evaluable patients. Humoral responses were documented by SEREX (Serologic Analysis of Recombinant cDNA Expression Libraries) analysis. A majority of patients with advanced disease demonstrated disease stabilization, with 3 patients showing ongoing stable disease at 12, 25, and 41 months, respectively. Vaccination with DC/multiple myeloma fusions was feasible and well tolerated and resulted in antitumor immune responses and disease stabilization in a majority of patients.

12 [468]. Schuster, S. J., S. S. Neelapu, et al. (2011). "Vaccination with patient-specific tumor-derived antigen in first remission improves disease-free survival in follicular lymphoma." J Clin Oncol **29**(20): 2787-2794.

PURPOSE: Vaccination with hybridoma-derived autologous tumor immunoglobulin (Ig) idiotype (Id) conjugated to keyhole limpet hemocyanin (KLH) and administered with granulocyte-monocyte colony-stimulating factor (GM-CSF) induces follicular lymphoma (FL) -specific immune responses. To determine the clinical benefit of this vaccine, we conducted a double-blind multicenter controlled phase III trial. PATIENTS AND METHODS: Treatment-naive patients with advanced stage FL achieving complete response (CR) or CR unconfirmed (CRu) after

chemotherapy were randomly assigned two to one to receive either Id vaccine (Id-KLH + GM-CSF) or control (KLH + GM-CSF). Primary efficacy end points were disease-free survival (DFS) for all randomly assigned patients and DFS for randomly assigned patients receiving at least one dose of Id vaccine or control. RESULTS: Of 234 patients enrolled, 177 (81%) achieved CR/CRu after chemotherapy and were randomly assigned. For 177 randomly assigned patients, including 60 patients not vaccinated because of relapse (n = 55) or other reasons (n = 5), median DFS between Id-vaccine and control arms was 23.0 versus 20.6 months, respectively (hazard ratio [HR], 0.81; 95% CI, 0.56 to 1.16; P = .256). For 117 patients who received Id vaccine (n = 76) or control (n = 41), median DFS after randomization was 44.2 months for Id-vaccine arm versus 30.6 months for control arm (HR, 0.62; 95% CI, 0.39 to 0.99; P = .047) at median follow-up of 56.6 months (range, 12.6 to 89.3 months). In an unplanned subgroup analysis, median DFS was significantly prolonged for patients receiving IgM-Id (52.9 v 28.7 months; P = .001) but not IgG-Id vaccine (35.1 v 32.4 months; P = .807) compared with isotype-matched control-treated patients. CONCLUSION: Vaccination with patient-specific hybridoma-derived Id vaccine after chemotherapy-induced CR/CRu may prolong DFS in patients with FL. Vaccine isotype may affect clinical outcome and explain differing results between this and other controlled Id-vaccine trials.

13 [477]. Timmerman, J. M., J. M. Vose, et al. (2009). "Tumor-specific recombinant idiotype immunisation after chemotherapy as initial treatment for follicular non-Hodgkin lymphoma." Leuk Lymphoma **50**(1): 37-46.

Tumor-specific variable regions of the clonal immunoglobulin (idiotype, Id) expressed by B cell non-Hodgkin lymphoma (NHL) can be targeted by active immunotherapy. We conducted a phase I/II trial to determine the safety and immunogenicity of a patient-specific, recombinant, mammalian cell-derived Id protein conjugated to keyhole limpet hemocyanin (Id-KLH; MyVax personalised immunotherapy) in 22 patients with follicular NHL in first remission after chemotherapy. Subjects received five subcutaneous immunisations with MyVax plus locally administered granulocyte-macrophage colony-stimulating factor (GM-CSF). Among 21 evaluable patients, 62% mounted Id-specific immune responses. Evoked anti-Id antibodies recognised both recombinant Id and native Id, and could specifically stain autologous tumor cells. At median follow-up of more than 6 years, median progression-free survival is 38 months.

Immunisation of follicular lymphoma patients with MyVax Id-KLH is safe and patients often mount tumor-specific immune responses. These results form the basis of a pivotal phase 3 trial of MyVax in follicular NHL.

14 [473]. Yi, Q., S. Szmania, et al. (2010). "Optimizing dendritic cell-based immunotherapy in multiple myeloma: intranodal injections of idiotype-pulsed CD40 ligand-matured vaccines led to induction of type-1 and cytotoxic T-cell immune responses in patients." Br J Haematol **150**(5): 554-564.

Vaccination with idiotype (Id) protein-pulsed dendritic cells (DCs) has been explored in multiple myeloma and the results have been disappointing. To improve the efficacy of DC vaccination in myeloma, we investigated the use of Id- and keyhole limpet haemocyanin (KLH)-pulsed, CD40 ligand-matured DCs administered intranodally. Nine patients with smouldering or stable myeloma without treatment were enrolled and DC vaccines were administered at weekly intervals for a total of four doses. Following vaccination, all patients mounted Id-specific gamma-interferon T-cell response. Interleukin-4 response was elicited in two, and skin delayed-type hypersensitivity reaction occurred in seven patients. More importantly, Id-specific cytotoxic T-cell responses were also detected in five patients. Most if not all patients mounted a positive T-cell response to KLH following vaccination. At 1-year follow-up, six of the nine patients had stable disease, while three patients had slowly progressive disease even during the vaccination period. At 5-year follow-up, four of the six patients continued with stable disease. No major side effects were noted. In summary, intranodal administration of Id-pulsed CD40 ligand-matured DCs was able to induce Id-specific T and B-cell responses in patients. Current efforts are geared towards breaking tumour-mediated immune suppression and improving clinical efficacy of this immunotherapy.

15 [467]. Zahradova, L., K. Mollova, et al. (2012). "Efficacy and safety of Id-protein-loaded dendritic cell vaccine in patients with multiple myeloma--phase II study results." Neoplasma **59**(4): 440-449.

In aphase II clinical study, pretreated multiple myeloma patients with relapsing or stable disease received autologous anticancer vaccine containing dendritic cells loaded with Id-protein. Patients received atotal of 6 vaccine doses intradermally in monthly intervals. No clinical responses were observed. During the follow-up with amedian of 33.1 months (range: 11-43 months), the disease remained stable in 7/11 (64%) of patients. Immune responses measured by ELISpot were noted in 3/11 (27%) and DTH skin test for Id-protein was positive in 8/11 (73%) of patients; out of those, 1/11 (9%) and 5/11 (46%), respectively, had preexisting immune response

to Id-protein before the vaccination began. Outcomes were compared to those of acontrol group of 13 patients. Atrend to lower cumulative incidence of progression in the vaccinated group was observed at 12 months from the first vaccination (p= 0.099). More patients from the control group compared to vaccinated patients required active anticancer therapy [4/11 (36%) vs. 8/13 (62%)]. Vaccines based on dendritic cells loaded with Id-protein are safe and induce specific immune response in multiple myeloma patients. Our results suggest that the vaccination could stabilize the disease in approximately two-thirds of patients. KEYWORDS: dendritic cells, immunotherapy, anticancer vaccines, Id-protein, multiple myeloma.
