# Supplementary material for: The Society for Immunotherapy of Cancer consensus statement on immunotherapy for the treatment of hematologic malignancies: multiple myeloma, lymphoma, and acute leukemia
Source: J Immunother Cancer. 2016 Dec 20;4:90. doi: 10.1186/s40425-016-0188-z (PMC5168808; doi:10.1186/s40425-016-0188-z)
Supplement: Additional file 3: — Annotated Bibliography (Myeloma), Annotated Bibliography (Lymphoma), and Annotated Bibliography (Leukemia). (ZIP 1812 kb) [file 40425_2016_188_MOESM3_ESM.zip › Add/APPENDIX II-Supplementary Biobliography II Cancer Immunotherapy Guidelines Lymphoma.pdf]

Cancer Immunotherapy Guidelines (Lymphoma)

An Annotated Bibliography of the Literature (in order of topic)

Society for Immunotherapy of Cancer

November 30, 2014

Table of Contents

TOPIC: Lymphoma + Rituximab (OR) Ofatumumab 7

TOPIC: Lymphoma + checkpoint blockade 52

TOPIC: Lymphoma + chimeric antigen receptor (OR) CAR (OR) CART 54

TOPIC: Lymphoma + (idiotype) vaccine 57

TOPIC: Lymphoma + Denileukin diftitox 57

TOPIC: Lymphoma + Interferon Alfa-2b 64

TOPIC: (Mantle cell) Lymphoma + Lenalidomide 71

TOPIC: (Mantle cell) Lymphoma + Bortezomib 74

TOPIC: Lymphoma + checkpoint inhibitor(s) or nivolumab or ipilimumab 74

TOPIC: Lymphoma + adoptive T Cell therapy or adoptive T cell transfer 74

TOPIC: Epstein-Barr virus (EBV) Positive Lymphomas 76

TOPIC: Epstein-Barr virus (EBV) Positive Lymphomas + adoptive T Cell therapy or adoptive T cell transfer ....

83 TOPIC: Selected References from Dr. Bishop..

84

Note: The bibliography is organized in topic order and then in alphabetical order by author. Duplicates and selected references have been removed in this bibliography. Dr. Bishop has also added selected references.

Note: The bibliography is organized in topic order and then in alphabetical order by author. Duplicates and selected references (by Dr. Bishop) have been removed in this bibliography. Dr. Bishop has also added selected references. The searches were conducted on 11/17/14 and 11/20/14 and 11/29/14 in the sequence and with the limits as follows:

Lymphoma Immunotherapy Literature Searches Conducted November 17 and 20 and 29, 2014									
**Search Terms**	**Date Limits**	**Query Translation**	**Date Search Completed**	**File Name with Dupes/File Name without Dupes/File Name without Dupes + Adds and Drops**	**EndNote record numbers**	**total records found**	**total # dupes**	**total # drops**	**Resulting # of records in bibliography**
**Lymphoma + Rituximab (OR) Ofatumumab**	**2004-2014**	**("lymphoma"[MeSH Major Topic] AND "rituximab"[Supplementary Concept]) OR "ofatumumab"[Supplementary Concept] AND (Randomized Controlled Trial[ptyp] AND ("2004/01/01"[PDAT] : "2014/12/31"[PDAT]) AND "humans"[MeSH Terms])**	11/17/2014	Lymphoma 112014.enl/ Lymphoma 112014 Duplicates Removed.enl/Lymphoma 113014 Dupes Removes Adds & Drops.enl	1-126	126	0	55	71
**Lymphoma + checkpoint blockade**	**2004-2014**	**"lymphoma"[MeSH Major Topic] AND (("cell cycle checkpoints"[MeSH Terms] OR ("cell"[All Fields] AND "cycle"[All Fields] AND "checkpoints"[All Fields]) OR "cell cycle checkpoints"[All Fields] OR "checkpoint"[All Fields]) AND blockade[All Fields]) AND ((Randomized Controlled Trial[ptyp] OR Clinical Trial[ptyp] OR Controlled Clinical Trial[ptyp]) AND ("2004/01/01"[PDAT] : "2014/12/31"[PDAT]) AND "humans"[MeSH Terms])**	11/17/2014	Lymphoma 112014.enl/ Lymphoma 112014 Duplicates Removed.enl/Lymphoma 113014 Dupes Removes Adds & Drops.enl	127-128	2	0	0	2
**Lymphoma + chimeric antigen receptor (OR) CAR (OR) CART**	**2004-2014**	**lymphoma[MeSH Major Topic] AND (("chimera"[MeSH Terms] OR "chimera"[All Fields] OR "chimeric"[All Fields]) AND ("receptors, antigen"[MeSH Terms] OR ("receptors"[All Fields] AND "antigen"[All Fields]) OR "antigen receptors"[All Fields] OR ("antigen"[All Fields] AND "receptor"[All Fields]) OR "antigen receptor"[All Fields])) AND ((Clinical Trial[ptyp] OR Controlled Clinical Trial[ptyp] OR Randomized Controlled Trial[ptyp]) AND ("2004/01/01"[PDAT] : "2014/12/31"[PDAT]) AND "humans"[MeSH Terms])**	11/17/2014	Lymphoma 112014.enl/ Lymphoma 112014 Duplicates Removed.enl/Lymphoma 113014 Dupes Removes Adds & Drops.enl	129-134	6	0	0	6

Lymphoma Immunotherapy Literature Searches Conducted November 17 and 20 and 29, 2014									
**Search Terms**	**Date Limits**	**Query Translation**	**Date Search Completed**	**File Name with Dupes/File Name without Dupes/File Name without Dupes + Adds and Drops**	**EndNote record numbers**	**total records found**	**total # dupes**	**total # drops**	**Resulting # of records in bibliography**
**Lymphoma + (idiotype) vaccine**	**2004-2014**	**lymphoma[MeSH Major Topic] AND (("immunoglobulin idiotypes"[MeSH Terms] OR ("immunoglobulin"[All Fields] AND "idiotypes"[All Fields]) OR "immunoglobulin idiotypes"[All Fields] OR "idiotype"[All Fields]) AND ("vaccines"[MeSH Terms] OR "vaccines"[All Fields] OR "vaccine"[All Fields])) AND ((Clinical Trial[ptyp] OR Controlled Clinical Trial[ptyp] OR Randomized Controlled Trial[ptyp]) AND ("2004/01/01"[PDAT] : "2014/12/31"[PDAT]) AND "humans"[MeSH Terms])**	11/17/2014	Lymphoma 112014.enl/ Lymphoma 112014 Duplicates Removed.enl/Lymphoma 113014 Dupes Removes Adds & Drops.enl	135-147	13	1	1	11
**Lymphoma + Denileukin diftitox**	**2004-2014**	**lymphoma[MeSH Major Topic] AND ("denileukin diftitox"[Supplementary Concept] OR "denileukin diftitox"[All Fields]) AND ((Clinical Trial[ptyp] OR Controlled Clinical Trial[ptyp] OR Randomized Controlled Trial[ptyp]) AND ("2004/01/01"[PDAT] : "2014/12/31"[PDAT]) AND "humans"[MeSH Terms])**	11/17/2014	Lymphoma 112014.enl/ Lymphoma 112014 Duplicates Removed.enl/Lymphoma 113014 Dupes Removes Adds & Drops.enl	148-163	16	0	3	13
**Lymphoma + Interferon Alfa-2b**	**2004-2014**	**lymphoma[MeSH Major Topic] AND (("interferon-alpha"[MeSH Terms] OR "interferon-alpha"[All Fields] OR ("interferon"[All Fields] AND "alpha"[All Fields]) OR "interferon alpha"[All Fields]) AND 2b[All Fields]) AND ((Clinical Trial[ptyp] OR Controlled Clinical Trial[ptyp] OR Randomized Controlled Trial[ptyp]) AND ("2004/01/01"[PDAT] : "2014/12/31"[PDAT]) AND "humans"[MeSH Terms])**	11/17/2014	Lymphoma 112014.enl/ Lymphoma 112014 Duplicates Removed.enl/Lymphoma 113014 Dupes Removes Adds & Drops.enl	164-172	9	1	3	5
**(Mantle cell) Lymphoma + Lenalidomide**	**2004-2014**	**(mantel[All Fields] AND ("cells"[MeSH Terms] OR "cells"[All Fields] OR "cell"[All Fields]) AND ("lymphoma"[MeSH Terms] OR "lymphoma"[All Fields])) AND ("lenalidomide"[Supplementary Concept] OR "lenalidomide"[All Fields]) AND ((Clinical Trial[ptyp] OR Controlled Clinical Trial[ptyp] OR Randomized Controlled Trial[ptyp]) AND ("2004/01/01"[PDAT] : "2014/12/31"[PDAT]) AND "humans"[MeSH Terms])**	11/17/2014	Lymphoma 112014.enl/ Lymphoma 112014 Duplicates Removed.enl/Lymphoma 113014 Dupes Removes	0	0	0	0	0

				Adds & Drops.enl					

Search Terms	Date Limits	Query Translation	Date Search Completed	File Name with Dupes/File Name without Dupes/File Name without Dupes + Adds and Drops	EndNote record numbers	total records found	total # dupes	total # drops	Resulting # of records in bibliography
(Mantle cell) Lymphoma + Bortezomib	2004-2014	(mantel[All Fields] AND ("cells"[MeSH Terms] OR "cells"[All Fields] OR "cell"[All Fields]) AND ("lymphoma"[MeSH Terms] OR "lymphoma"[All Fields])) AND ("bortezomib"[Supplementary Concept] OR "bortezomib"[All Fields]) AND ((Clinical Trial[ptyp] OR Controlled Clinical Trial[ptyp] OR Randomized Controlled Trial[ptyp]) AND ("2004/01/01"[PDAT] : "2014/12/31"[PDAT]) AND "humans"[MeSH Terms])	11/17/2014	Lymphoma 112014.enl/ Lymphoma 112014 Duplicates Removed.enl/Lymphoma 113014 Dupes Removes Adds & Drops.enl	0	0	0	0	0
Lymphoma + checkpoint inhibitor(s) or nivolumab or ipilimumab	2004-2014	(("lymphoma"[MeSH Major Topic] AND (("cell cycle checkpoints"[MeSH Terms] OR ("cell"[All Fields] AND "cycle"[All Fields] AND "checkpoints"[All Fields]) OR "cell cycle checkpoints"[All Fields] OR "checkpoint"[All Fields]) AND inhibitor[All Fields])) OR ("nivolumab"[Supplementary Concept] OR "nivolumab"[All Fields])) OR ("ipilimumab"[Supplementary Concept] OR "ipilimumab"[All Fields]) AND (Clinical Trial[ptyp] AND ("2004/01/01"[PDAT] : "2014/12/31"[PDAT]) AND "humans"[MeSH Terms])	11/20/2014	Lymphoma 112014.enl/ Lymphoma 112014 Duplicates Removed.enl/Lymphoma 113014 Dupes Removes Adds & Drops.enl	173-243	71	0	68	3

**Lymphoma + adoptive T Cell therapy or adoptive T cell transfer**	**2004-2014**	**("lymphoma"[MeSH Major Topic] AND (adoptive[All Fields] AND ("t-lymphocytes"[MeSH Terms] OR "t-lymphocytes"[All Fields] OR "t cell"[All Fields]) AND ("therapy"[Subheading] OR "therapy"[All Fields] OR "therapeutics"[MeSH Terms] OR "therapeutics"[All Fields]))) OR (adoptive[All Fields] AND ("tlymphocytes"[MeSH Terms] OR "t-lymphocytes"[All Fields] OR "t cell"[All Fields]) AND ("transfer (psychology)"[MeSH Terms] OR ("transfer"[All Fields] AND "(psychology)"[All Fields]) OR "transfer (psychology)"[All Fields] OR "transfer"[All Fields])) AND (Clinical Trial[ptyp] AND ("2004/01/01"[PDAT] : "2014/12/31"[PDAT]) AND "humans"[MeSH Terms])**	11/20/2014	Lymphoma 112014.enl/ Lymphoma 112014 Duplicates Removed.enl/Lymphoma 113014 Dupes Removes Adds & Drops.enl	244-357	114	6	97	11
**Epstein-Barr virus (EBV) Positive Lymphomas**	**2004-2014**	**(("herpesvirus 4, human"[MeSH Terms] OR "human herpesvirus 4"[All Fields] OR ("epstein"[All Fields] AND "barr"[All Fields] AND "virus"[All Fields]) OR "epstein barr virus"[All Fields]) AND positive[All Fields] AND ("lymphoma"[MeSH Terms] OR "lymphoma"[All Fields] OR "lymphomas"[All Fields])) AND (Clinical Trial[ptyp] AND ("2004/01/01"[PDAT] : "2014/12/31"[PDAT]) AND "humans"[MeSH Terms])**	11/20/2014	Lymphoma 112014.enl/ Lymphoma 112014 Duplicates Removed.enl/Lymphoma 113014 Dupes Removes Adds & Drops.enl	358-383	26	2	23	1
**Lymphoma Immunotherapy Literature Searches Conducted November 17 and 20 and 29, 2014**									
**Search Terms**	**Date Limits**	**Query Translation**	**Date Search Completed**	**File Name with Dupes/File Name without Dupes/File Name without Dupes + Adds and Drops**	**EndNote record numbers**	**total records found**	**total # dupes**	**total # drops**	**Resulting # of records in bibliography**

**Epstein-Barr virus (EBV) Positive Lymphomas + adoptive T Cell therapy or adoptive T cell transfer**	**2004-2014**	**((("herpesvirus 4, human"[MeSH Terms] OR "human herpesvirus 4"[All Fields] OR ("epstein"[All Fields] AND "barr"[All Fields] AND "virus"[All Fields]) OR "epstein barr virus"[All Fields]) AND positive[All Fields] AND ("lymphoma"[MeSH Terms] OR "lymphoma"[All Fields] OR "lymphomas"[All Fields])) AND (adoptive[All Fields] AND ("t-lymphocytes"[MeSH Terms] OR "tlymphocytes"[All Fields] OR "t cell"[All Fields]) AND ("therapy"[Subheading] OR "therapy"[All Fields] OR "therapeutics"[MeSH Terms] OR "therapeutics"[All Fields]))) OR (adoptive[All Fields] AND ("t-lymphocytes"[MeSH Terms] OR "tlymphocytes"[All Fields] OR "t cell"[All Fields]) AND ("transfer (psychology)"[MeSH Terms] OR ("transfer"[All Fields] AND "(psychology)"[All Fields]) OR "transfer (psychology)"[All Fields] OR "transfer"[All Fields])) AND (Clinical Trial[ptyp] AND ("2004/01/01"[PDAT] : "2014/12/31"[PDAT]) AND "humans"[MeSH Terms])**	11/20/2014	Lymphoma 112014.enl/ Lymphoma 112014 Duplicates Removed.enl/Lymphoma 113014 Dupes Removes Adds & Drops.enl	384-491	108	108	0	0
**Selected references from Dr. Bishop**	**1998-2014**	**Email from Dr. Bishop 11/25/14: Attached is the lymphoma bibliography with abstracts highlighted that are not relevant to lymphoma and/or immunotherapy. In addition I have attached a list of citations that need to be included in the bibliography that were not identified in the literature search. Thanks to Lori for all her hard work. Sincerely, Michael Michael R. Bishop, MD**	11/29/2014	Lymphoma 112014.enl/ Lymphoma 112014 Duplicates Removed.enl/Lymphoma 113014 Dupes Removes Adds & Drops.enl	492-511	20	5	0	15
				**TOTALS**		**511**	**123**	**250**	**138**

NOTE: IN THE BIBLIOGRAPHY, THE NUMBER IN BRACKETS IS THE RECORD NUMBER IN THE ENDNOTE DATABASE (e.g., 100 is the record number for the first item in this bibliography). This is the correct number to use for identifying references in the manuscript during the manuscript draft stages and for any other purposes.

TOPIC: Lymphoma + Rituximab (OR) Ofatumumab

1 [100]. Ansell, S. M., S. M. Geyer, et al. (2006). "Randomized phase II study of interleukin-12 in combination with rituximab in previously treated non-Hodgkin's lymphoma patients." Clin Cancer Res **12**(20 Pt 1): 6056-6063.

PURPOSE: Rituximab is a chimeric antibody that induces B-cell apoptosis and recruits immune effector cells to mediate cell lysis. Interleukin-12 (IL-12) facilitates cytolytic responses by T cells and natural killer cells. This phase II study was done to determine the efficacy and toxicity of IL-12 in combination with rituximab in patients with B-cell non-Hodgkin's lymphoma (NHL). EXPERIMENTAL DESIGN: Fifty-eight patients with histologically confirmed relapsed B-cell NHL were randomized to receive concurrent treatment with rituximab and IL-12 (arm A) or rituximab with subsequent treatment with IL-12 after documented nonresponse or progression after rituximab (arm B). Treatment consisted of 375 mg/m2 rituximab on days 1, 8, 15, and 22 and 300 ng/kg IL-12 given s.c. twice weekly starting on day 2 for arm A or upon progression for arm B. RESULTS: The overall response rate was 37% (11 of 30) in arm A and 52% (13 of 25) in arm B. All of the responses seen in arm B occurred while patients received rituximab, and no responses occurred during treatment with subsequent IL-12. The median duration of response was 16 months for arm A and 12 months for arm B. Biopsy specimens were serially obtained in a subset of patients and showed that changes in gene expression were different when cells from the peripheral blood were compared with cells from lymph node biopsies. CONCLUSIONS: The concomitant use of IL-12 and rituximab had modest disease activity in patients with B-cell NHL, but the sequential administration of IL-12 after rituximab did not result in additional clinical responses.

2 [7]. Ardeshna, K. M., W. Qian, et al. (2014). "Rituximab versus a watch-and-wait approach in patients with advanced-stage, asymptomatic, non-bulky follicular lymphoma: an open-label randomised phase 3 trial." Lancet Oncol **15**(4): 424-435.

BACKGROUND: Patients with advanced-stage, low-tumour-burden follicular lymphoma have conventionally undergone watchful waiting until disease progression. We assessed whether rituximab use could delay the

need for chemotherapy or radiotherapy compared with watchful waiting and the effect of this strategy on quality of life (QoL). METHODS: Asymptomatic patients (aged >/=18 years) with low-tumour-burden follicular lymphoma (grades 1, 2, and 3a) were randomly assigned centrally (1:1:1), by the minimisation approach stratified by institution, grade, stage, and age, to watchful waiting, rituximab 375 mg/m(2) weekly for 4 weeks (rituximab induction), or rituximab induction followed by a maintenance schedule of 12 further infusions given at 2-monthly intervals for 2 years (maintenance rituximab). On Sept 30, 2007, recruitment into the rituximab induction group was closed and the study was amended to a two-arm study. The primary endpoints were time to start of new treatment and QoL at month 7 (ie, 6 months after completion of rituximab induction). All randomly assigned patients were included in the analysis of time to start of new treatment on an intention-to-treat basis. The main study is now completed and is in long-term follow-up. The study is registered with ClinicalTrials.gov, NCT00112931. FINDINGS: Between Oct 15, 2004, and
March 25, 2009, 379 patients from 118 centres in the UK, Australia, New Zealand, Turkey, and Poland were randomly assigned to watchful waiting or maintenance rituximab. 84 patients were recruited to the rituximab induction group before it was closed early. There was a significant difference in the time to start of new treatment, with 46% (95% CI 39-53) of patients in the watchful waiting group not needing treatment at 3 years compared with 88% (83-92) in the maintenance rituximab group (hazard ratio [HR] 0.21, 95% CI 0.14-0.31; p<0.0001). 78% (95% CI 69-87) of patients in the rituximab induction group did not need treatment at 3 years, which was significantly more than in the watchful waiting group (HR 0.35, 95% CI 0.22-0.56; p<0.0001), but no different compared with the maintenance rituximab group (0.75, 0.41-1.34; p=0.33). Compared with the watchful waiting group, patients in the maintenance rituximab group had significant improvements in the Mental Adjustment to Cancer scale score (p=0.0004), and Illness Coping Style score (p=0.0012) between baseline and month 7. Patients in the rituximab induction group did not show improvements in their QoL compared with the watchful waiting group. There were 18 serious adverse events reported in the rituximab groups (four in the rituximab induction group and 14 in the maintenance rituximab group), 12 of which were grade 3 or 4 (five infections, three allergic reactions, and four cases of neutropenia), all of which fully resolved. INTERPRETATION: Rituximab monotherapy should be considered as a treatment option for patients with asymptomatic, advanced-stage, low-tumour-burden follicular lymphoma. FUNDING: Cancer Research UK, Lymphoma Research Trust, Lymphoma Association, and Roche.

3 [95]. Aviles, A., M. J. Nambo, et al. (2007). "Rituximab and escalated chemotherapy in elderly patients with aggressive diffuse large-cell lymphoma: a controlled clinical trial." Cancer Biother Radiopharm **22**(2): 194-199.

The treatment of elderly patients with aggressive malignant lymphoma has not been defined. The addition of rituximab to conventional chemotherapy has been reported to improve the outcome, but most patients have good prognostic factors (performance status < 2, no severe associated diseases, low or low-intermediate clinical risk). Thus, we developed a combined regimen, including escalated doses of anthracycline and rituximab. The endpoint was to improve event-free survival (EFS) and overall survival. Two hundred and four (204) patients were randomly assigned to receive an escalated chemotherapy regimen (CEOP) with escalated dose of epirubicin, compared to the same regimen and addition of rituximab. All patients had poor prognostic factors: high- or high-intermediate clinical risk, poor performance status, bulky disease, and more than 2 with extranodal involvement. In an intent-to-treat analysis, all patients were evaluable for efficacy and toxicity. The complete response rates were similar in both arms: 74% in chemotherapy and 78% in the rituximab + chemotherapy program. EFS and overall survival were similar: 77% and 84%, respectively, in combined chemotherapy and 75% and 81% in the rituximab-chemotherapy regimen. Toxicity was mild and well tolerated. In elderly patients with diffuse large-cell lymphoma and poor prognostic factors, rituximab did not improve their outcome.

4 [94]. Aviles, A., M. J. Nambo, et al. (2007). "Dose dense (CEOP-14) vs dose dense and rituximab (CEOP-14 +R) in high-risk diffuse large cell lymphoma." Med Oncol **24**(1): 85-89.

To assess efficacy and toxicity of rituximab and dose chemotherapy in high-risk diffuse large cell lymphoma, we conducted a controlled clinical trial to assess efficacy and toxicity of a dose-dense regimen CEOP- 14 (cyclophosphamide, epirubicin, vincristine, and prednisone every 14 d) compared to CEOP-14 plus rituximab. One hundred and ninety-six patients were randomized to received CEOP-rituximab (cyclophosphamide 1500 mg/m2, epirubicin 120 mg/m2, vincristine, and prednisone at standard dose and rituximab at 375 mg/m2) compared with the same chemotherapy administered every 14 d (CEOP-14). In an intent-to-treat analysis all patients were available for efficacy and toxicity. Complete response in CEOP-14 was observed in 73 cases (74%) and in 75 patients (76%) in the CEOP-R regimen (76%) (p = 0.8). With a median follow-up of 53.4 mo, median has not been reached in time to tumor-progression (TTP) and overall survival (OS). Actuarial curves at 5 yr showed that TTP and OS in patients treated with CEOP-R were 74% and 67%, respectively, that were not statistical different when compared to CEOP-14, 72% and 65%, respectively (p = 0.8). Acute toxicity was mild and well tolerated. The use

of a dense-dose regimen is useful and well tolerated in patients with very high risk diffuse large cell lymphoma. The addition of rituximab did not improve outcome in these setting of patients.

5 [23]. Bachy, E., R. Houot, et al. (2013). "Long-term follow up of the FL2000 study comparing CHVP-interferon to CHVP-interferon plus rituximab in follicular lymphoma." Haematologica **98**(7): 1107-1114.

Anti-CD20-containing chemotherapy regimens have become the standard of care for patients with follicular lymphoma needing cytotoxic therapy. Four randomized trials demonstrated a clinical benefit for patients treated with rituximab. However, no long-term follow up (i.e. > 5 years) of these trials is yet available. Between May 2000 and May 2002, 358 newly diagnosed patients with high tumor burden follicular lymphoma were randomized to receive cyclophosphamide, adriamycin, etoposide and prednisolone plus interferon-alpha2a or a similar chemotherapy-based regimen plus rituximab, and outcome was up-dated. With a median follow up of 8.3 years, addition of rituximab remained significantly associated with prolonged event-free survival (primary end point) (P=0.0004) with a trend towards a benefit for overall survival (P=0.076). The Follicular Lymphoma International Prognostic Index score was strongly associated with outcome for both event-free and overall survival in univariate analysis and its prognostic value remained highly significant after adjusting for other significant covariates in multivariate models (P<0.0001 and P=0.001, respectively). Considering long-term toxicity, the addition of rituximab in the first-line setting was confirmed as safe with regards to development of secondary malignancies. Long-term follow up of patients with follicular lymphoma treated in the FL2000 study confirms the sustained clinical benefit of rituximab without long-term toxicity.

6 [3]. Byrd, J. C., J. R. Brown, et al. (2014). "Ibrutinib versus ofatumumab in previously treated chronic lymphoid leukemia." N Engl J Med **371**(3): 213-223.

BACKGROUND: In patients with chronic lymphoid leukemia (CLL) or small lymphocytic lymphoma (SLL), a short duration of response to therapy or adverse cytogenetic abnormalities are associated with a poor outcome. We evaluated the efficacy of ibrutinib, a covalent inhibitor of Bruton's tyrosine kinase, in patients at risk for a poor outcome. METHODS: In this multicenter, open-label, phase 3 study, we randomly assigned 391 patients with relapsed or refractory CLL or SLL to receive daily ibrutinib or the anti-CD20 antibody ofatumumab. The primary end point was the duration of progression-free survival, with the duration of overall survival and the overall response rate as secondary end points. RESULTS: At a median follow-up of 9.4 months, ibrutinib significantly improved

progression-free survival; the median duration was not reached in the ibrutinib group (with a rate of progression-free survival of 88% at 6 months), as compared with a median of 8.1 months in the ofatumumab group (hazard ratio for progression or death in the ibrutinib group, 0.22; P<0.001). Ibrutinib also significantly improved overall survival (hazard ratio for death, 0.43; P=0.005). At 12 months, the overall survival rate was 90% in the ibrutinib
group and 81% in the ofatumumab group. The overall response rate was significantly higher in the ibrutinib group than in the ofatumumab group (42.6% vs. 4.1%, P<0.001). An additional 20% of ibrutinib-treated patients had a partial response with lymphocytosis. Similar effects were observed regardless of whether patients had a chromosome 17p13.1 deletion or resistance to purine analogues. The most frequent nonhematologic adverse events were diarrhea, fatigue, pyrexia, and nausea in the ibrutinib group and fatigue, infusion-related reactions, and cough in the ofatumumab group. CONCLUSIONS: Ibrutinib, as compared with ofatumumab, significantly improved progression-free survival, overall survival, and response rate among patients with previously treated CLL or SLL. (Funded by Pharmacyclics and Janssen; RESONATE ClinicalTrials.gov number, NCT01578707.).

7 [90]. Canioni, D., G. Salles, et al. (2008). "High numbers of tumor-associated macrophages have an adverse prognostic value that can be circumvented by rituximab in patients with follicular lymphoma enrolled onto the GELA-GOELAMS FL-2000 trial." J Clin Oncol **26**(3): 440-446.

PURPOSE: High amounts of intratumoral macrophages have been shown to correlate with poor prognosis in patients with follicular lymphoma (FL) treated with chemotherapy without rituximab. We tried to establish whether intratumoral macrophage count (MC) definitely is able to predict the outcome of FL patients in the rituximab era. PATIENTS AND METHODS: We analyzed immunohistochemical CD68 expression in 194 FL patients from the FL-2000 trial, randomly assigned to receive cyclophosphamide, doxorubicin, etoposide, prednisolone, and interferon (CHVP-I) or rituximab plus CHVP-I. Immunohistochemistry was performed on paraffin sections using anti-CD68 KP1 antibody, and stained macrophages were scored on high-power field (hpf) in either intrafollicular (IF) or extrafollicular (EF) areas. RESULTS: For IF MC, the best cutoff point was estimated at 10 macrophages/hpf. Low IF MC was significantly associated with a better event-free survival (EFS; P = .011). However, this effect was observed only in the CHVP-I arm (P = .012) and not in the rituximab plus CHVP-I arm. Using a cutoff of 15 IF MC, we found no significant association with EFS. For EF MC, fewer than 22 macrophages/hpf were associated with better EFS

in the CHVP-I arm (P = .02) but not in the rituximab plus CHVP-I arm. CONCLUSION: These results show that MC can predict outcome of FL patients and that rituximab is able to circumvent the unfavorable outcome associated with high MC.

8 [46]. Coiffier, B., E. A. Osmanov, et al. (2011). "Bortezomib plus rituximab versus rituximab alone in patients with relapsed, rituximab-naive or rituximab-sensitive, follicular lymphoma: a randomised phase 3 trial." Lancet Oncol **12**(8): 773-784.

BACKGROUND: Bortezomib and rituximab have shown additive activity in preclinical models of lymphoma, and have been shown to be active and generally well tolerated in a randomised phase 2 study in patients with follicular and marginal zone lymphoma. We compared the efficacy and safety of rituximab alone or combined with bortezomib in patients with relapsed or refractory follicular lymphoma in a phase 3 setting. METHODS: In this multicentre phase 3 trial, rituximab-naive or rituximab-sensitive patients aged 18 years or older with relapsed grade 1 or 2 follicular lymphoma were randomly assigned (1:1) to receive five 35-day cycles consisting of intravenous infusions of rituximab 375 mg/m(2) on days 1, 8, 15, and 22 of cycle 1, and on day 1 of cycles 2-5, either alone or with bortezomib 1.6 mg/m(2), administered by intravenous injection on days 1, 8, 15, and 22 of all cycles. Randomisation was stratified by FLIPI score, previous use of rituximab, time since last therapy, and region. Treatment assignment was based on a computer-generated randomisation schedule prepared by the sponsor. Patients and treating physicians were not masked to treatment allocation. The primary endpoint was progression-free survival analysed by intention to treat. This trial has been completed and is registered with ClinicalTrials.gov, number NCT00312845. FINDINGS: Between April 10, 2006, and Aug 12, 2008, 676 patients were randomised to receive rituximab (n=340) or bortezomib plus rituximab (n=336). After a median follow-up of 33.9 months (IQR 26.4-39.7), median progression-free survival was 11.0 months (95% CI 9.1-12.0) in the rituximab group and 12.8 months (11.5-15.0) in the bortezomib plus rituximab group (hazard ratio 0.82, 95% CI 0.68-0.99; p=0.039). The magnitude of clinical benefit was not as large as the anticipated prespecified improvement of 33% in progression-free survival. Patients in both groups received a median of five treatment cycles (range 1-5); 245 of 339 (72%) and 237 of 334 (71%) patients in the rituximab and bortezomib plus rituximab groups, respectively, completed five cycles. Of patients who did not complete five cycles, most discontinued early because of disease progression (77 [23%] patients in the rituximab group, and 56 [17%] patients in the bortezomib plus rituximab group). Rates of adverse events of grade 3 or higher (70 [21%] of 339 rituximab-treated patients vs 152 [46%] of 334

bortezomib plus rituximab treated patients), and serious adverse events (37 [11%] patients vs 59 [18%] patients) were lower in the rituximab group than in the combination group. The most common adverse events of grade 3 or higher were neutropenia (15 [4%] patients in the rituximab group and 37 [11%] patients in the bortezomib plus rituximab group), infection (15 [4%] patients and 36 [11%] patients, respectively), diarrhoea (no patients and 25 [7%] patients, respectively), herpes zoster (one [<1%] patient and 12 [4%] patients, respectively), nausea or vomiting (two [<1%] patients and 10 [3%] patients, respectively) and thrombocytopenia (two [<1%] patients and 10
[3%] patients, respectively). No individual serious adverse event was reported by more than three patients in the rituximab group; in the bortezomib plus rituximab group, only pneumonia (seven patients [2%]) and pyrexia (six patients [2%]) were reported in more than five patients. In the bortezomib plus rituximab group 57 (17%) of 334 patients had peripheral neuropathy (including sensory, motor, and sensorimotor neuropathy), including nine (3%) with grade 3 or higher, compared with three (1%) of 339 patients in the rituximab group (no events of grade >/=3). No patients in the rituximab group but three (1%) patients in the bortezomib plus rituximab group died of adverse events considered at least possibly related to treatment. INTERPRETATION: Although a regimen of bortezomib plus rituximab is feasible, the improvement in progression-free survival provided by this regimen versus rituximab alone was not as great as expected. The regimen might represent a useful addition to the armamentarium, particularly for some subgroups of patients. FUNDING: Johnson & Johnson Pharmaceutical Research & Development and Millennium Pharmaceuticals, Inc.

9 [60]. Coiffier, B., C. Thieblemont, et al. (2010). "Long-term outcome of patients in the LNH-98.5 trial, the first randomized study comparing rituximab-CHOP to standard CHOP chemotherapy in DLBCL patients: a study by the Groupe d'Etudes des Lymphomes de l'Adulte." Blood **116**(12): 2040-2045.

We report the outcome of patients included in the LNH-98.5 study, which compared cyclophosphamide, doxorubicin, vincristine, and prednisone (CHOP) to rituximab plus CHOP (R-CHOP) therapy in 399 patients with diffuse large B-cell lymphoma (DLBCL) aged 60 to 80 years, with a median follow-up time of 10 years. Clinical event information was updated in all living patients (with the exception of 3 patients) in 2009. Survival end points were improved in patients treated with R-CHOP: the 10-year progression-free survival was 36.5%, compared with 20% with CHOP alone, and the 10-year overall survival was 43.5% compared with 27.6%. The same risk of death due to other diseases, secondary cancers, and late relapses was

observed in both study arms. Relapses occurring after 5 years represented 7% of all disease progressions. The results from the 10-year analysis confirm the benefits and tolerability of the addition of rituximab to CHOP. Our findings underscore the need to treat elderly patients as young patients, with the use of curative chemotherapy.

10 [115]. Crump, M., L. Shepherd, et al. (2005). "A randomized phase III study of gemcitabine, dexamethasone, and cisplatin versus dexamethasone, cytarabine, and cisplatin as salvage chemotherapy followed by posttransplantation rituximab maintenance therapy versus observation for treatment of aggressive B-Cell and T-Cell non-Hodgkin's lymphoma." Clin Lymphoma **6**(1): 56-60.

11 [39]. Czuczman, M. S., L. Fayad, et al. (2012). "Ofatumumab monotherapy in rituximab-refractory follicular lymphoma: results from a multicenter study." Blood **119**(16): 3698-3704.

New treatments are required for rituximab-refractory follicular lymphoma (FL). In the present study, patients with rituximab-refractory FL received 8 weekly infusions of ofatumumab (CD20 mAb; dose 1, 300 mg and doses 2-8, 500 or 1000 mg; N = 116). The median age of these patients was 61 years, 47% had high-risk Follicular Lymphoma International Prognostic Index scores, 65% were chemotherapy-refractory, and the median number of prior therapies was 4. The overall response rate was 13% and 10% for the 500-mg and 1000-mg arms, respectively. Among 27 patients refractory to rituximab monotherapy, the overall response rate was 22%. The median progression-free survival was 5.8 months. Forty-six percent of patients demonstrated tumor reduction 3 months after therapy initiation, and the median progression-free survival for these patients was 9.1 months. The most common adverse events included infections, rash, urticaria, fatigue, and pruritus. Three patients experienced grade 3 infusion-related reactions, none of which were considered serious events. Grade 3-4 neutropenia, leukopenia, anemia, and thrombocytopenia occurred in a subset of patients. Ofatumumab was well tolerated and modestly active in this heavily pretreated, rituximab-refractory population and is therefore now being studied in less refractory FL and in combination with other agents in various B-cell neoplasms. The present study was registered at www.clinicaltrials.gov as NCT00394836.

12 [37]. Czuczman, M. S., G. Hess, et al. (2012). "Chemoimmunotherapy with ofatumumab in combination with CHOP in previously untreated follicular lymphoma." Br J Haematol **157**(4): 438-445.

An international, Phase II trial was conducted to assess two doses of ofatumumab, a human CD20 monoclonal antibody, combined with cyclophosphamide (750 mg/m(2)), doxorubicin (50 mg/m(2)), prednisone (100 mg days 3-7) and vincristine (1.4 mg/m(2)) (O-CHOP), as frontline treatment for follicular lymphoma (FL). 59 patients with previously untreated FL were randomized to ofatumumab 500 mg (n = 29) or 1000 mg (n = 30) day 1, with CHOP on day 3 every 3 weeks for six cycles. Median duration of FL was 0.1 years for both dose groups; 34% and 38% of patients had high-risk Follicular Lymphoma International Prognostic Index (FLIPI) scores in the 500- and 1000-mg dose groups, respectively. Overall response rate was 90% for the 500-mg group and 100% for the 1000-mg group. 62% of patients achieved complete response (CR)/unconfirmed CR (CRu). 76% of patients with FLIPI score 3-5 attained CR/CRu. Longer follow-up time is needed for analysis of survival end points. The most common Common Terminology Criteria grade 3-4 investigator-reported adverse events were leucopenia (29%) and neutropenia (22%). No deaths have been reported.
O-CHOP was safe and efficacious in patients with previously untreated FL, including high-risk FLIPI groups. This trial was registered at www.clinicaltrials.gov (NCT00494780).

13 [9]. Davies, A., F. Merli, et al. (2014). "Pharmacokinetics and safety of subcutaneous rituximab in follicular lymphoma (SABRINA): stage 1 analysis of a randomised phase 3 study." Lancet Oncol **15**(3): 343-352.

BACKGROUND: Intravenous rituximab is a mainstay of treatment for follicular lymphoma. A subcutaneous formulation that achieves equivalent rituximab serum concentrations might improve convenience and save health-care resources without sacrificing clinical activity. We aimed to assess pharmacokinetic non-inferiority of 3 week cycles of fixed-dose subcutaneous rituximab versus standard intravenous rituximab. METHODS: In our two-stage, randomised, open-label, phase 3 trial, we enrolled patients with previously untreated grade 1-3a, CD20-positive follicular lymphoma at 67 centres in 23 countries. In stage 1, we randomly allocated patients 1:1 with the Pocock and Simon algorithm to intravenous rituximab (375 mg/m(2)) or fixed-dose subcutaneous rituximab (1400 mg), stratified by induction chemotherapy regimen (cyclophosphamide, doxorubicin, vincristine, prednisone or cyclophosphamide, vincristine, prednisone), Follicular Lymphoma International Prognostic Index score, and region. After randomisation, patients received one induction dose of intravenous rituximab in cycle 1 and then allocated treatment for cycles 2-8. Patients with a complete or partial response following induction therapy continued intravenous or

subcutaneous rituximab as maintenance every 8 weeks. The primary endpoint was the ratio of observed rituximab serum trough concentrations (Ctrough) between groups at cycle 7 (before cycle 8 dosing) of induction treatment in a per-protocol population. Patients were analysed as treated for safety endpoints. Stage 2 follow-up is ongoing and is fully accrued. This study is registered with ClinicalTrials.gov, number NCT01200758. FINDINGS: Between Feb 4, 2010, and Oct 21, 2011, we enrolled 127 patients. Pharmacokinetic data were available for 48 (75%) of 64 patients randomly allocated intravenous rituximab and 54 (86%) of 63 patients randomly allocated subcutaneous rituximab. Geometric mean Ctrough was 83.13 mug/mL in the intravenous group and 134.58 mug/mL in the subcutaneous group (ratio 1.62, 90% CI 1.36-1.94), showing non-inferiority of subcutaneous rituximab. 57 (88%) of 65 patients in the intravenous rituximab safety population had adverse events (30 [46%] grade >/=3), as did 57 (92%) of 62 patients in the subcutaneous rituximab safety population (29 [47%] grade >/=3). The most common grade 3 or worse adverse event in both groups was neutropenia (14 [22%] patients in the intravenous group and 16 [26%] patients in the subcutaneous group). Adverse events related to administration were mostly grade 1-2 and occurred in 21 (32%) patients in the intravenous group and 31 (50%) patients in the subcutaneous group. INTERPRETATION: Stage 1 data show that the pharmacokinetic profile of subcutaneous rituximab was non-inferior to intravenous rituximab and was not associated with new safety concerns. Stage 2 will provide data for efficacy and safety of the subcutaneous administration. FUNDING: F Hoffmann-La Roche.

14 [75]. Eve, H. E., D. Linch, et al. (2009). "Toxicity of fludarabine and cyclophosphamide with or without rituximab as initial therapy for patients with previously untreated mantle cell lymphoma: results of a randomised phase II study." Leuk Lymphoma **50**(2): 211-215.

The National Cancer Research Network (NCRN) is currently coordinating a Phase III randomised study (LY05) comparing fludarabine and cyclophosphamide (FC) with or without rituximab (R) for previously untreated mantle cell lymphoma (MCL). The combination of FC is well-recognised as significantly immunosuppressive and there are concerns that adding rituximab may increase infection risk further. The impact of rituximab on other markers of toxicity is also unclear. We analysed the toxicity data on 139 patients treated within the NCRN LY05 trial. Non-hematological toxicity was similar between the two treatment arms. The only difference in hematological toxicity was a higher rate of lymphocytopenia with fludarabine cyclophosphamide and rituximab

(FCR), which did not translate into increased febrile episodes or infections. In conclusion, the addition of rituximab to FC for previously untreated MCL has no significant impact on toxicity.

15 [117]. Feugier, P., A. Van Hoof, et al. (2005). "Long-term results of the R-CHOP study in the treatment of elderly patients with diffuse large B-cell lymphoma: a study by the Groupe d'Etude des Lymphomes de l'Adulte." J Clin Oncol **23**(18): 4117-4126.

PURPOSE: To analyze the long-term outcome of patients included in the Lymphome Non Hodgkinien study 98-5 (LNH98-5) comparing cyclophosphamide, doxorubicin, vincristine, and prednisone (CHOP) to rituximab plus CHOP (R-CHOP) in elderly patients with diffuse large B-cell lymphoma. PATIENTS AND METHODS: LNH98-5 was a randomized study that included 399 previously untreated patients, age 60 to 80 years, with diffuse large B-cell lymphoma. Patients received eight cycles of classical CHOP (cyclophosphamide 750 mg/m(2), doxorubicin 50 mg/m(2), vincristine 1.4 mg/m(2), and prednisone 40 mg/m(2) for 5 days) every 3 weeks. In R-CHOP, rituximab 375 mg/m(2) was administered the same day as CHOP. Survivals were analyzed using the intent-to-treat principle. RESULTS: Median follow-up is 5 years at present. Event-free survival, progression-free survival, disease-free survival, and overall survival remain statistically significant in favor of the combination of R-CHOP (P = .00002, P < .00001, P < .00031, and P < .0073, respectively, in the log-rank test). Patients with low-risk or high-risk lymphoma according to the age-adjusted International Prognostic Index have longer survivals if treated with the combination. No long-term toxicity appeared to be associated with the R-CHOP combination. CONCLUSION: Using the combination of R-CHOP leads to significant improvement of the outcome of elderly patients with diffuse large B-cell lymphoma, with significant survival benefit maintained during a 5-year follow-up. This combination should become the standard for treating these patients.

16 [124]. Forstpointner, R., M. Dreyling, et al. (2004). "The addition of rituximab to a combination of fludarabine, cyclophosphamide, mitoxantrone (FCM) significantly increases the response rate and prolongs survival as compared with FCM alone in patients with relapsed and refractory follicular and mantle cell lymphomas: results of a prospective randomized study of the German Low-Grade Lymphoma Study Group." Blood **104**(10): 3064-3071.

In follicular lymphoma (FL) and mantle cell lymphoma (MCL) the monoclonal antibody rituximab may improve the prognosis when combined with chemotherapy. This was investigated in a prospective randomized study in patients with relapsed disease. A total of 147 patients

were randomized to receive 4 courses of chemotherapy with 25 mg/m(2) fludarabine on days 1 to 3, 200 mg/m(2) cyclophosphamide on days 1 to 3, and 8 mg/m(2) mitoxantrone on day 1 (FCM), alone or combined with rituximab (375 mg/m(2); R-FCM). Of 128 evaluable patients, 62 were randomized for FCM and 66 for R-FCM. R-FCM revealed an overall response rate of 79% (33% complete remission [CR], 45% partial remission [PR]) as compared with 58% for FCM alone (13% CR, 45% PR; P = .01), with similar results in a subgroup analysis of FL (94% vs 70%) and MCL (58% vs 46%). In the total group, the R-FCM arm was significantly superior concerning progression-free survival (PFS; P = .0381) and overall survival (OS; P = .0030). In FL PFS was significantly longer in the R-FCM arm (P = .0139) whereas in MCL a significantly longer OS was observed (P = .0042). There were no differences in clinically relevant side effects in both study arms. Hence, the addition of rituximab to FCM chemotherapy significantly improves the outcome of relapsed or refractory FL and MCL.

17 [101]. Forstpointner, R., M. Unterhalt, et al. (2006). "Maintenance therapy with rituximab leads to a significant prolongation of response duration after salvage therapy with a combination of rituximab, fludarabine, cyclophosphamide, and mitoxantrone (R-FCM) in patients with recurring and refractory follicular and mantle cell lymphomas: Results of a prospective randomized study of the German Low Grade Lymphoma Study Group (GLSG)." Blood **108**(13): 4003-4008.

In follicular lymphoma (FL) and mantle cell lymphoma (MCL) the monoclonal antibody rituximab (R) improves the prognosis when combined with chemotherapy. The present study investigated R-maintenance after R-chemotherapy. Patients with recurring or refractory FL and MCL were randomized to 4 courses of fludarabine, cyclophosphamide, and mitoxantrone (FCM) alone or combined with R (R-FCM). Responding patients underwent a second randomization for R-maintenance comprising 2 further courses of 4-times-weekly doses of R after 3 and 9 months. The first randomization was stopped after 147 patients, when R-FCM revealed a significantly better outcome. All subsequent patients received R-FCM. Of the 176 patients who are currently evaluable (as of October 2005), 138 received R-FCM for remission induction. Response duration was significantly prolonged by R-maintenance after R-FCM, with the median not being reached in this evaluation versus an estimated median of 16 months (P = .001). This beneficial effect was also observed when analyzing FL (P = .035) and MCL (P = .049) separately. Hence, R-maintenance is effective after salvage with R-chemotherapy and significantly prolongs response duration in patients with recurring or refractory FL or MCL.

18 [73]. Freedman, A., S. S. Neelapu, et al. (2009). "Placebo-controlled phase III trial of patient-specific immunotherapy with mitumprotimut-T and granulocyte-macrophage colony-stimulating factor after rituximab in patients with follicular lymphoma." J Clin Oncol **27**(18): 3036-3043.

PURPOSE: To evaluate patient-specific immunotherapy with mitumprotimut-T (idiotype keyhole limpet hemocyanin [Id-KLH]) and granulocyte-macrophage colony-stimulating factor (GM-CSF) in CD20(+) follicular lymphoma. PATIENTS AND METHODS: Patients with treatment-naive or relapsed/refractory disease achieving a complete response (CR), partial response (PR), or stable disease (SD) with four weekly rituximab infusions were randomly assigned to mitumprotimut-T/GM-CSF or placebo/GM-CSF, with doses given monthly for six doses, every 2 months for six doses, and then every 3 months until disease progression (PD). Randomization was stratified by prior therapy (treatment-naive or relapsed/refractory) and response to rituximab (CR/PR or SD). The primary end point was time to progression (TTP) from randomization. RESULTS: A total of 349 patients were randomly assigned; median age was 54 years, 79% were treatment naive, and 86% had stage III/IV disease. Median TTP was 9.0 months for mitumprotimut-T/GM-CSF and 12.6 months for placebo/GM-CSF (hazard ratio [HR] = 1.384; P = .019). TTP was comparable between the two arms in treatment-naive patients (HR = 1.196; P = .258) and shorter with mitumprotimut-T/GM-CSF in relapsed/refractory disease (HR = 2.265; P = .004). After adjusting for Follicular Lymphoma International Prognostic Index (FLIPI) scores, the difference in TTP between the two arms was no longer significant. Overall objective response rate, rate of response improvement, and duration of response were comparable between the two arms. Toxicity was similar in the two arms; 76% of adverse events were mild or moderate, and 94% of patients had injection site reactions. CONCLUSION: TTP was shorter with mitumprotimut-T/GM-CSF compared with placebo/GM-CSF. This difference was possibly due to the imbalance in FLIPI scores.

19 [34]. Ghesquieres, H., G. Cartron, et al. (2012). "Clinical outcome of patients with follicular lymphoma receiving chemoimmunotherapy in the PRIMA study is not affected by FCGR3A and FCGR2A polymorphisms." Blood **120**(13): 2650-2657.

In patients with follicular lymphoma treated with single-agent rituximab, single nucleotide polymorphisms in the FCGR3A gene are known to influence response and progression-free survival. The prognostic role of

FCGR3A and FCGR2A polymorphisms in patients with follicular lymphoma treated with rituximab and chemotherapy combination remains controversial and has not been evaluated in the context of rituximab maintenance. FCGR3A and FCGR2A single nucleotide polymorphisms were evaluated in, respectively, 460 and 455 patients treated in the PRIMA study to investigate whether these were associated with response rate and patient outcome after rituximab chemotherapy induction and 2-year rituximab maintenance. In this representative patient cohort, complete and unconfirmed complete responses after rituximab chemotherapy were observed in 65%, 67%, 66% (P = .86) and 60%, 72%, 66% (P = .21) of FCGR3A VV, VF, FF and FCGR2A HH, HR, RR carriers, respectively. After 2 years of rituximab maintenance (or observation), response rates did not differ among the different genotypes. Progression-free survival measured from either treatment initiation or randomization to observation or maintenance was not influenced by these polymorphisms. These data indicate that FCGR3A and FCGR2A polymorphisms do not influence response rate and outcome when rituximab is combined with chemotherapy or used as maintenance treatment. The PRIMA study is registered at www.clinicaltrials.gov as NCT00140582.

20 [120]. Ghielmini, M., S. F. Schmitz, et al. (2005). "Effect of single-agent rituximab given at the standard schedule or as prolonged treatment in patients with mantle cell lymphoma: a study of the Swiss Group for Clinical Cancer Research (SAKK)." J Clin Oncol **23**(4): 705-711.

PURPOSE: To evaluate the effect of single-agent rituximab given at the standard or a prolonged schedule in patients with newly diagnosed, or refractory or relapsed mantle cell lymphoma (MCL). PATIENTS AND METHODS: After induction treatment with the standard schedule (375 mg/m2 weekly x 4), patients who were responding or who had stable disease at week 12 from the start of treatment were randomly assigned to no further treatment (arm A) or prolonged rituximab administration (375 mg/m2) every 8 weeks for four times (arm B). RESULTS: The trial enrolled 104 patients. After induction, clinical response was 27% with 2% complete responses. Among patients with detectable t(11;14)-positive cells in blood and bone marrow at baseline, four of 20, and one of 14, respectively, became polymerase chain-reaction-negative after induction. Anemia was the only adverse predictor of response in the multivariate analysis. After a median follow-up of 29 months, response rate and duration of response were not significantly different between the two schedules in 61 randomly assigned patients. Median event-free survival (EFS) was 6 months in arm A versus 12 months in arm B; the difference was not significant (P = .1). Prolonged treatment seemed to improve EFS in the

subgroup of pretreated patients (5 months in arm A v 11 months in arm B; P = .04). Thirteen percent of patients in arm A and 9% in arm B presented with grade 3 to 4 hematologic toxicity. CONCLUSION: Single-agent rituximab is active in MCL, but the addition of four single doses at 8-week intervals does not seem to significantly improve response rate, duration of response, or EFS after treatment with the standard schedule.

21 [126]. Ghielmini, M., S. F. Schmitz, et al. (2004). "Prolonged treatment with rituximab in patients with follicular lymphoma significantly increases event-free survival and response duration compared with the standard weekly x 4 schedule." Blood **103**(12): 4416-4423.

The potential benefits of extended rituximab treatment have been investigated in a randomized trial comparing the standard schedule with prolonged treatment in 202 patients with newly diagnosed or refractory/relapsed follicular lymphoma (FL). All patients received standard treatment (rituximab 375 mg/m(2) weekly x 4). In 185 evaluable patients, the overall response rate was 67% in chemotherapy-naive patients and 46% in pretreated cases (P <.01). Patients responding or with stable disease at week 12 (n = 151) were randomized to no further treatment or prolonged rituximab administration (375 mg/m(2) every 2 months for 4 times). At a median follow-up of 35 months, the median event-free survival (EFS) was 12 months in the no further treatment versus 23 months in the prolonged treatment arm (P =.02), the difference being particularly notable in chemotherapy-naive patients (19 vs 36 months; P =.009) and in patients responding to induction treatment (16 vs 36 months; P =.004). The number of t(14;18)-positive cells in peripheral blood (P =.0035) and in bone marrow (P =.0052) at baseline was predictive for clinical response. Circulating normal B lymphocytes and immunoglobulin M (IgM) plasma levels decreased for a significantly longer time after prolonged treatment, but the incidence of adverse events was not increased. In patients with FL, the administration of 4 additional doses of rituximab at 8-week intervals significantly improves the EFS.

22 [32]. Gisselbrecht, C., N. Schmitz, et al. (2012). "Rituximab maintenance therapy after autologous stem-cell transplantation in patients with relapsed CD20(+) diffuse large B-cell lymphoma: final analysis of the collaborative trial in relapsed aggressive lymphoma." J Clin Oncol **30**(36): 4462-4469.

PURPOSE: The standard treatment for relapsed diffuse large B-cell lymphoma (DLBCL) is salvage chemotherapy followed by high-dose therapy and autologous stem-cell transplantation (ASCT). The impact of maintenance rituximab after ASCT is not known. PATIENTS AND METHODS: In total, 477 patients with CD20(+) DLBCL who were in their first relapse or

refractory to initial therapy were randomly assigned to one of two salvage regimens. After three cycles of salvage chemotherapy, the responding patients received high-dose chemotherapy followed by ASCT. Then, 242 patients were randomly assigned to either rituximab every 2 months for 1 year or observation. RESULTS: After ASCT, 122 patients received rituximab, and 120 patients were observed only. The median follow-up time was 44 months. The 4-year event-free survival (EFS) rates after ASCT were 52% and 53% for the rituximab and observation groups, respectively (P = .7). Treatment with rituximab was associated with a 15% attributable risk of serious adverse events after day 100, with more deaths (six deaths v three deaths in the observation arm). Several factors affected EFS after ASCT (P < .05), including relapsed disease within 12 months (EFS: 46% v 56% for relapsed disease after 12 months), secondary age-adjusted International Prognostic Index (saaIPI) more than 1 (EFS: 37% v 61% for saaIPI < 1), and prior treatment with rituximab (EFS: 47% v 59% for no prior rituximab). A significant difference in EFS between women (63%) and men (46%) was also observed in the rituximab group. In the Cox model for maintenance, the saaIPI was a significant prognostic factor (P < .001), as was male sex (P = .01). CONCLUSION: In relapsed DLBCL, we observed no difference between the control group and the rituximab maintenance group and do not recommend rituximab after ASCT.

23 [4]. Glass, B., J. Hasenkamp, et al. (2014). "Rituximab after lymphoma-directed conditioning and allogeneic stem-cell transplantation for relapsed and refractory aggressive non-Hodgkin lymphoma (DSHNHL R3): an open-label, randomised, phase 2 trial." Lancet Oncol **15**(7): 757-766.

BACKGROUND: Allogeneic stem-cell transplantation has had limited success for patients with refractory and relapsed aggressive B-cell or T-cell lymphoma. We investigated the effect of adding rituximab to standard prophylaxis for graft-versus-host disease after transplantation and estimated overall survival when using a lymphoma-directed myeloablative conditioning regimen. METHODS: We did this randomised, open-label, phase 2 study at seven German transplantation centres. We enrolled patients with aggressive B-cell or T-cell lymphoma and primary refractory disease, early relapse (<12 months after first-line treatment), or relapse after autologous transplantation. Conditioning with fludarabine (125
mg/m(2)), busulfan (12 mg/kg oral or 9.6 mg/kg intravenous), and cyclophosphamide (120 mg/kg) was followed by allogeneic stem-cell transplantation. Patients were randomly assigned (1:1) to receive rituximab (375 mg/m(2) on days 21, 28, 35, 42, 175, 182, 189, and 196) or not. Allocation was done with a centralised computer-generated

procedure; patients were stratified by histological subtype (B-cell vs T-cell lymphoma) and donor match (HLA-identical vs non-identical). Neither investigators nor patients were masked to allocation. The primary endpoints were the incidence of acute graft-versus-host disease grade 2-4 in each treatment group and overall survival at 1 year in both groups combined. All analyses were done for the intention-to-treat population. The study is registered with ClinicalTrials.gov, number NCT00785330. FINDINGS: Between June 16, 2004, and March 24, 2009, we screened 86 patients and enrolled 84; 42 were randomly assigned to each group. The cumulative incidence of grade 2-4 acute graft-versus-host disease was 46% (95% CI 32-62) in the rituximab group and 42% (95% CI 29-59) in the no rituximab group (hazard ratio [HR] 0.91, 95% CI 0.52-1.60; p=0.74). Overall survival at 1 year for the whole study population was 52% (95% CI 41-62). Grade 4 haematological toxic effects and grade 3 alopecia occurred in all patients. The most common non-haematological grade 5 toxic effects were pneumonia (nine in the no rituximab group vs ten in the rituximab group) and other infections (seven vs four). INTERPRETATION: The lymphoma-directed myeloablative conditioning regimen developed here is promising for patients with refractory and relapsed aggressive B-cell and T-cell lymphomas. However, the addition of rituximab did not affect the incidence of graft-versus-host disease or overall survival. FUNDING: Hoffmann-La Roche, Amgen, Astellas Pharma.

24 [103]. Habermann, T. M., E. A. Weller, et al. (2006). "Rituximab-CHOP versus CHOP alone or with maintenance rituximab in older patients with diffuse large B-cell lymphoma." J Clin Oncol **24**(19): 3121-3127.

PURPOSE: To address early and late treatment failures in older patients with diffuse large B-cell lymphoma (DLBCL), we designed a two-stage randomized trial of cyclophosphamide, doxorubicin, vincristine, and prednisone (CHOP) versus rituximab plus CHOP (R-CHOP), with a second random assignment to maintenance rituximab (MR) or observation in responding patients. PATIENTS AND METHODS: Untreated DLBCL patients who were 60 years or older were randomly assigned to R-CHOP (n = 318) or CHOP (n = 314); 415 responders were randomly assigned to MR (n = 207) or observation (n = 208). The primary end point was failure-free survival (FFS).

All P values were two sided. RESULTS: Three-year FFS rate was 53% for R-CHOP patients and 46% for CHOP patients (P = .04) at a median follow-up time of 3.5 years. Two-year FFS rate from second random assignment was 76% for MR compared with 61% for observation (P = .009).

No significant differences in survival were seen according to induction or maintenance therapy. FFS was prolonged with MR after CHOP (P = .0004)

but not after R-CHOP (P = .81) with 2-year FFS rates from second random assignment of 77%, 79%, 74%, and 45% for R-CHOP, R-CHOP + MR, CHOP + MR, and CHOP, respectively. In a secondary analysis excluding MR patients, R-CHOP alone reduced the risks of treatment failure (P = .003) and death (P = .05) compared with CHOP alone. CONCLUSION: Rituximab administered as induction or maintenance with CHOP chemotherapy significantly prolonged FFS in older DLBCL patients. After R-CHOP, no benefit was provided by MR. These results, which are consistent with an additive effect of rituximab, suggest that future studies could focus on maintenance strategies with novel agents as well as new induction therapies.

25 [104]. Hagberg, H., C. Gisselbrecht, et al. (2006). "Randomised phase III study of R-ICE versus R-DHAP in relapsed patients with CD20 diffuse large B-cell lymphoma (DLBCL) followed by high-dose therapy and a second randomisation to maintenance treatment with rituximab or not: an update of the CORAL study." Ann Oncol **17 Suppl 4**: iv31-32.

The multicentre phase III CORAL study aims to guide choice of salvage chemotherapy in diffuse large B-cell lymphoma (DLBCL) and assess the role of rituximab maintenance after autologous stem cell transplantation (ASCT). Patients are first randomised between ICE (ifosfamide, carboplatin, etoposide) and DHAP (dexamethasone, ara-C and cisplatin), both combined with rituximab (R-ICE or R-DHAP). After three courses, responders are treated by ASCT with BEAM. A second randomisation then allocates patients to maintenance treatment with rituximab 375 mg/m(2), one injection every 2 months six times, or observation. Accrual to the study is now proceeding well and the planned 400 patients are likely to be enrolled within the next 1.5 years. Results to date are very preliminary but suggest encouraging rates of response. However, they also indicate that initial exposure to rituximab may increase the difficulty of salvaging patients who fail first-line therapy.

26 [119]. Hainsworth, J. D., S. Litchy, et al. (2005). "Maximizing therapeutic benefit of rituximab: maintenance therapy versus re-treatment at progression in patients with indolent non-Hodgkin's lymphoma--a randomized phase II trial of the Minnie Pearl Cancer Research Network." J Clin Oncol **23**(6): 1088-1095.

PURPOSE: To compare the benefit of maintenance rituximab therapy versus rituximab re-treatment at progression in patients with previously treated indolent non-Hodgkin's lymphoma. PATIENTS AND METHODS: Between June 1998 and August 2002, 114 patients who had received previous chemotherapy for indolent non-Hodgkin's lymphoma were treated with a standard 4-week course of rituximab. Patients with

objective response or stable disease were randomly assigned to receive either maintenance rituximab therapy (standard 4-week courses administered at 6-month intervals) or rituximab re-treatment at the time of lymphoma progression. The duration of rituximab benefit was measured from the date of first rituximab treatment until the date other treatment was required. RESULTS: Ninety (79%) of 114 patients had objective response or stable disease after initial rituximab treatment, and were randomly assigned to treatment. Progression-free survival was prolonged in the maintenance group (31.3 v 7.4 months; P = .007). Final overall and complete response rates were higher in the maintenance group. Duration of rituximab benefit was similar in the maintenance and re-treatment groups (31.3 v 27.4 months, respectively). More maintenance patients remain in continuous remission, and more are currently in complete remission. Both treatment approaches were well tolerated. CONCLUSION: In patients who have objective response or stable disease with single-agent rituximab therapy, duration of rituximab benefit is substantially prolonged with either scheduled maintenance treatment or rituximab re-treatment at the time of progression. At present, the magnitude of benefit with either approach appears similar. However, additional follow-up of this trial is required, and completion of phase III randomized trials is necessary to definitively answer this question.

27 [72]. Haioun, C., N. Mounier, et al. (2009). "Rituximab versus observation after high-dose consolidative first-line chemotherapy with autologous stem-cell transplantation in patients with poor-risk diffuse large B-cell lymphoma." Ann Oncol **20**(12): 1985-1992.

BACKGROUND: This study compared the induction regimens doxorubicin, cyclophosphamide and etoposide (ACE) with doxorubicin, cyclophosphamide, vincristine, bleomycin and prednisone (ACVBP) before high-dose therapy (HDT) followed by autologous stem-cell transplantation (ASCT) for patients with poor-risk diffuse large B-cell lymphoma (DLBCL). A second randomisation compared rituximab with observation post-ASCT. MATERIALS AND METHODS: Four hundred and seventy-six patients <60 years old with newly diagnosed CD20+ DLBCL were randomised to induction with ACE or ACVBP. Three hundred and thirty responders received HDT followed by ASCT. After ASCT, 269 patients were re-randomised to receive either maintenance rituximab or observation alone. Randomisation was stratified by the quality of response to ASCT. The primary end point of this study was event-free survival (EFS). RESULTS: At a median of 4 years' follow-up from the second randomisation, there was a trend (P = 0.1) towards increased EFS for patients who received rituximab compared with observation. CONCLUSION: The type of

induction therapy (ACVBP or ACE) did not significantly affect overall survival at a median 51 months' follow-up.

28 [97]. Herold, M., A. Haas, et al. (2007). "Rituximab added to first-line mitoxantrone, chlorambucil, and prednisolone chemotherapy followed by interferon maintenance prolongs survival in patients with advanced follicular lymphoma: an East German Study Group Hematology and Oncology Study." J Clin Oncol **25**(15): 1986-1992.

PURPOSE: Rituximab has been shown to be active in follicular lymphoma (FL), both as monotherapy and in combination with chemotherapy. We conducted a randomized trial comparing mitoxantrone, chlorambucil, and prednisolone (MCP) chemotherapy plus rituximab with MCP alone. PATIENTS AND METHODS: Previously untreated patients with stage III or IV CD20+ indolent or mantle cell lymphoma were randomly assigned to either eight 28-day cycles of MCP plus rituximab (R-MCP; n = 181) or eight cycles of MCP alone (n = 177). All patients who achieved a complete or partial remission were treated with interferon maintenance until relapse. Herein, we report the results from the primary analysis population of patients with FL, who constituted the majority of patients (56%) recruited to the trial (n = 201; R-MCP, n = 105; MCP, n = 96). RESULTS: Rates of overall and complete response were significantly higher in the R-MCP arm than the MCP arm (overall response, 92% v 75%, respectively; P = .0009; complete response, 50% v 25%, respectively; P = .004). With a median follow-up time of 47 months, median event-free survival (EFS) and progression-free survival (PFS) times were significantly prolonged with R-MCP compared with MCP (EFS, not reached v 26 months, respectively; P < .0001; PFS, not reached v 28.8 months, respectively; P < .0001), and overall survival (OS) was significantly improved with R-MCP compared with MCP (4-year OS rate, 87% v 74%, respectively; P = .0096). CONCLUSION: The R-MCP regimen significantly improves complete and overall response rates, EFS, PFS, and OS in patients with previously untreated advanced FL, without a clinically significant increase in toxicity.

29 [51]. Heutte, N., C. Haioun, et al. (2011). "Quality of life in 269 patients with poor-risk diffuse large B-cell lymphoma treated with rituximab versus observation after autologous stem cell transplant." Leuk Lymphoma **52**(7): 1239-1248.

We aimed to assess quality of life (QoL) following front-line autologous stem cell transplant (ASCT) and the QoL relationship with rituximab maintenance, in patients with diffuse large B-cell lymphoma. Patients were then randomized to either one weekly rituximab injection for 4 weeks, or observation alone. Patients (n = 269) were given the European

Organisation for Research and Treatment of Cancer (EORTC) QLQ-C30 questionnaires. Scales for all symptoms exhibited similar temporal patterns, with a marked increase, followed by a plateau after 1 year. The proportion of patients with a clinically significant improvement varied from 6%
(constipation) to 56% (fatigue). Age, gender, and previous treatment-induced toxicities were not predictive of variations in QoL. Rituximab significantly reduced pain and symptom severity. Our results for QoL showed that patients experienced rapid recovery after ASCT in all the domains tested. Differences in QoL improvement with time were not connected with rituximab maintenance.

30 [114]. Hiddemann, W., M. Kneba, et al. (2005). "Frontline therapy with rituximab added to the combination of cyclophosphamide, doxorubicin, vincristine, and prednisone (CHOP) significantly improves the outcome for patients with advanced-stage follicular lymphoma compared with therapy with CHOP alone: results of a prospective randomized study of the German Low-Grade Lymphoma Study Group." Blood **106**(12): 3725-3732.

Phase 2 studies suggest that the monoclonal antibody rituximab may improve the prognosis of patients with follicular lymphoma (FL) when it is added to chemotherapy. In the current study, 428 patients with untreated, advanced-stage FL were randomly assigned for therapy with cyclophosphamide, doxorubicin, vincristine, and prednisone (CHOP) alone (n = 205) or CHOP combined with rituximab (R-CHOP) (n = 223). R-CHOP reduced the relative risk for treatment failure by 60% and significantly prolonged the time to treatment failure (P < .001). In addition, a significantly higher overall response rate (96% vs 90%; P = .011) and a prolonged duration of remission (P = .001) were achieved. In spite of a relatively short observation time, these beneficial effects even translated to superior overall survival (P = .016), with 6 deaths in the R-CHOP group compared with 17 deaths in the CHOP group within the first 3 years. The predominant treatment-related adverse effect was myelosuppression. Severe granulocytopenia was more frequently observed after R-CHOP (63% vs 53%; P = .01). However, severe infections were rare and of similar frequency after R-CHOP and CHOP (5% and 7%). Hence, adding rituximab to CHOP significantly improves the outcome for patients with previously untreated advanced-stage FL and does not induce major adverse effects.

31 [84]. Hirt, C., F. Schuler, et al. (2008). "Rapid and sustained clearance of circulating lymphoma cells after chemotherapy plus rituximab: clinical

significance of quantitative t(14;18) PCR monitoring in advanced stage follicular lymphoma patients." Br J Haematol **141**(5): 631-640.

This study of first-line treatment in advanced-stage follicular lymphoma patients analysed the effects of MCP (mitoxantrone, chlorambucil and prednisolone) chemotherapy alone or in combination with rituximab (R-MCP) on circulating lymphoma cells (CLC) and assessed the prognostic value of a quantitative monitoring of CLC. CLC numbers were determined by quantitative polymerase chain reaction (PCR) for the t(14;18)-translocation or by allele-specific PCR for rearranged immunoglobulin heavy chain genes. We analysed blood samples from 43 patients treated in a randomized trial comparing eight cycles of MCP versus R-MCP. Clearance of CLC at the end of therapy was achieved in 21/25 patients (84%) treated with R-MCP compared with 0/18 after MCP alone (P < 0.0001). A > or = 2 log CLC reduction was associated with a favourable clinical response (P = 0.0004) and prolonged event-free survival (P = 0.02). In R-MCP patients, stable CLC numbers or consistently PCR-negative blood samples were associated with a continuing clinical remission whereas in two patients a relapse was preceded by a > or = 2 log CLC increase. This study demonstrated that R-MCP led to a rapid and sustained eradication of CLC and a > or = 2 log CLC reduction was associated with a superior quality and duration of the clinical response.

32 [74]. Hochster, H., E. Weller, et al. (2009). "Maintenance rituximab after cyclophosphamide, vincristine, and prednisone prolongs progression-free survival in advanced indolent lymphoma: results of the randomized phase III ECOG1496 Study." J Clin Oncol **27**(10): 1607-1614.

PURPOSE: To determine if maintenance rituximab (MR) after standard chemotherapy improves progression-free survival (PFS) in advanced-stage indolent lymphoma. PATIENTS AND METHODS: Patients with stage III-IV indolent lymphoma with responding or stable disease after cyclophosphamide, vincristine, and prednisone (CVP) chemotherapy were stratified by initial tumor burden, residual disease after CVP (minimal or gross), and histology, and randomly assigned to observation (OBS) or MR 375 mg/m(2) once per week for 4 weeks every 6 months for 2 years. PFS was the primary end point. RESULTS: Three hundred eleven (282 with follicular lymphoma) evaluable patients who received CVP were randomly assigned to OBS (n = 158) or MR (n = 153). Best response improved in 22% MR versus 7% OBS patients (P = .00006). Toxicity was minimal in both study arms. Three-year PFS after random assignment was 68% MR versus 33% OBS (hazard ratio [HR] = 0.4; P = 4.4 x 10(-10) [all patients]) and 64% MR v 33% OBS (HR = 0.4; P = 9.2 x 10(-8) [patients with follicular lymphoma]). There was an advantage for MR regardless of

Follicular Lymphoma International Prognostic Index score, tumor burden, residual disease, or histology. In multivariate analysis of MR patients, minimal disease after CVP was a favorable prognostic factor. OS at 3 years was 92% MR versus 86% OBS (HR = 0.6; log-rank one-sided P = .05) and, among patients with follicular lymphoma, OS was 91% MR versus 86% (HR = 0.6; log-rank one-sided P = .08). A trend favoring MR was observed among patients with high tumor burden (log-rank one-sided P = .03). CONCLUSION: The E1496 study provides the first phase III data in untreated indolent lymphoma that MR after chemotherapy significantly prolongs PFS.

33 [116]. Kaplan, L. D., J. Y. Lee, et al. (2005). "Rituximab does not improve clinical outcome in a randomized phase 3 trial of CHOP with or without rituximab in patients with HIV-associated non-Hodgkin lymphoma: AIDS-Malignancies Consortium Trial 010." Blood **106**(5): 1538-1543.

The addition of rituximab to cyclophosphamide, doxorubicin, vincristine, and prednisone (CHOP) chemotherapy results in significant improvement in clinical outcome for individuals with non-HIV-associated aggressive B-cell lymphoma. To assess the potential risks and benefits of the addition of rituximab to CHOP for HIV-associated non-Hodgkin lymphoma (HIV-NHL) 150 patients receiving CHOP for HIV-NHL were randomized (2:1) to receive 375 mg/m(2) rituximab with each chemotherapy cycle (n = 99) or no immunotherapy (n = 50) in a multicenter phase 3 trial. The complete response rate (CR + CRu) was 57.6% for R-CHOP and 47% for CHOP (P = .147). With a median follow-up of 137 weeks, time to progression, progression-free survival, and overall survival times were 125, 45, and 139 weeks, respectively, for R-CHOP and 85, 38, and 110 weeks, respectively, for CHOP (P = not significant, all comparisons). Treatment-related infectious deaths occurred in 14% of patients receiving R-CHOP compared with 2% in the chemotherapy-alone group (P = .035). Of these deaths, 60% occurred in patients with CD4 counts less than 50/mm(3). Progression-free survival was significantly influenced by CD4(+) count (P < .001) and International Prognostic Index score (P = .022), but not bcl-2 status. The addition of rituximab to CHOP in patients with HIV-NHL may be associated with improved tumor responses. However, these benefits may be offset by an increase in infectious deaths, particularly in those individuals with CD4(+) lymphocyte counts less than 50/mm(3).

34 [83]. Kasteng, F., M. Erlanson, et al. (2008). "Cost-effectiveness of maintenance rituximab treatment after second line therapy in patients with follicular lymphoma in Sweden." Acta Oncol **47**(6): 1029-1036.

INTRODUCTION: Rituximab has significantly improved the prognosis for patients with both indolent and aggressive non-Hodgkin's lymphoma. An economic evaluation was carried out to assess the cost-effectiveness in Sweden of rituximab as maintenance therapy for patients with follicular lymphoma in remission after second line therapy. MATERIALS AND METHODS: The incremental cost and effectiveness of rituximab maintenance therapy versus observation were evaluated in a health-state transition model. Primary effect measures were quality-adjusted life-years (QALY) and life-years gained (LYG). Model state transitions were calculated based on progression-free and overall survival data from the EORTC20981 trial. The analysis was made from the perspective of the healthcare provider, including direct medical costs presented in euro, 2007 value. Effects and costs were discounted at a 3% annual rate. The stability of the base case results were tested in one-way and probabilistic sensitivity analyses. RESULTS: The evaluation assessed rituximab maintenance therapy to be associated with an incremental cost per QALY gained of euro 12,600 and an incremental cost per LYG of euro 11,200. The average discounted life expectancy for patients on rituximab maintenance was 1.0 year longer than for patients on observation (5.96 vs. 4.94 years). Rituximab maintenance was associated with an additional 0.9 QALY, and total costs per patient were euro 11,500 higher in the treatment arm, compared to observation. DISCUSSION: The results indicate that rituximab maintenance treatment after successful induction therapy for patients with relapsed/refractory follicular lymphoma in Sweden is cost-effective compared to observation.

35 [29]. Ketterer, N., B. Coiffier, et al. (2013). "Phase III study of ACVBP versus ACVBP plus rituximab for patients with localized low-risk diffuse large B-cell lymphoma (LNH03-1B)." Ann Oncol **24**(4): 1032-1037.

BACKGROUND: The superiority of a chemotherapy with doxorubicin, cyclophosphamide, vindesine, bleomycin and prednisone (ACVBP) in comparison with cyclophosphamide, doxorubicin, vincristin and prednisone plus radiotherapy for young patients with localized diffuse large B-cell lymphoma (DLBCL) was previously demonstrated. We report the results of a trial which evaluates the role of rituximab combined with ACVBP (R-ACVBP) in these patients. PATIENTS AND METHODS: Untreated patients younger than 66 years with stage I or II DLBCL and no adverse prognostic factors of the age-adjusted International Prognostic Index were randomly assigned to receive three cycles of ACVBP plus sequential consolidation with or without the addition of four infusions of rituximab. RESULTS: A total of 223 patients were randomly allocated to the study, 110 in the R-ACVBP group and 113 in the ACVBP group. After a median follow-

up of 43 months, our 3-year estimate of event-free survival was 93% in the R-ACVBP group and 82% in the ACVBP group (P = 0.0487). Three-year estimate of progression-free survival was increased in the R-ACVBP group (95% versus 83%, P = 0.0205). Overall survival did not differ between the two groups with a 3-year estimates of 98% and 97%, respectively (P = 0.686). CONCLUSION: In young patients with low-risk localized DLBCL, rituximab combined with three cycles of ACVBP plus consolidation is significantly superior to ACVBP plus consolidation alone.

36 [89]. Kimby, E., J. Jurlander, et al. (2008). "Long-term molecular remissions in patients with indolent lymphoma treated with rituximab as a single agent or in combination with interferon alpha-2a: a randomized phase II study from the Nordic Lymphoma Group." Leuk Lymphoma **49**(1): 102-112.

The purpose of this phase II randomized trial was to evaluate the effect and safety of interferon-alpha2a (IFN) in combination with extended dosing rituximab in patients with symptomatic, advanced indolent lymphoma responding to a standard single course of rituximab. Totally 123 patients were treated with rituximab 375 mg/m2 once weekly for 4 weeks leading to 14 complete response (CR; 11%), 56 partial response (PR; 46%), and 13 minor responses (MR; 11%). Patients achieving either PR or MR were randomized to four more infusions of rituximab alone (n = 36) or in combination with five weeks of IFN (n = 33), with an overall response rate (CR + PR) of 78% and 94%, respectively. Significantly more patients in the combination arm improved their response from PR/MR to CR (P < 0.05) and more maintained their responses for > or = 24 months (72% versus 50%), respectively. Overall, 26 out of the 52 patients who achieved CR underwent minimal residual disease (MRD) evaluation. Totally 17 of these (65%) achieved MRD negativity, 14 of whom remain in CR after 4.8 years' follow-up. The addition of IFN to rituximab was generally safe, but reversible thrombocytopenia and neutropenia were noted in one and six patients, respectively, requiring a reduction in the IFN dose. Extended rituximab is effective and well tolerated and combination with IFN seems to improve both the quality and duration of the responses, providing the opportunity to achieve long-term molecular CRs and prolonged failure-free survival without chemotherapy.

37 [118]. Lenz, G., M. Dreyling, et al. (2005). "Immunochemotherapy with rituximab and cyclophosphamide, doxorubicin, vincristine, and prednisone significantly improves response and time to treatment failure, but not long-term outcome in patients with previously untreated mantle cell lymphoma: results of a prospective randomized trial of the German Low Grade Lymphoma Study Group (GLSG)." J Clin Oncol **23**(9): 1984-1992.

PURPOSE: Mantle cell lymphoma (MCL) is characterized by a poor prognosis with a low to moderate sensitivity to chemotherapy and a median survival of only 3 to 4 years. In an attempt to improve outcome, the German Low Grade Lymphoma Study Group (GLSG) initiated a randomized trial comparing the combination of cyclophosphamide, doxorubicin, vincristine, and prednisone (CHOP) and rituximab (R-CHOP) with CHOP alone as first-line therapy for advanced-stage MCL. PATIENTS AND METHODS: One hundred twenty-two previously untreated patients with advanced-stage MCL were randomly assigned to six cycles of CHOP (n = 60) or R-CHOP (n = 62). Patients up to 65 years of age achieving a partial or complete remission underwent a second randomization to either myeloablative radiochemotherapy followed by autologous stem-cell transplantation or interferon alfa maintenance (IFNalpha). All patients older than 65 years received IFNalpha maintenance. RESULTS: R-CHOP was significantly superior to CHOP in terms of overall response rate (94% v 75%; P = .0054), complete remission rate (34% v 7%; P = .00024), and time to treatment failure (TTF; median, 21 v 14 months; P = .0131). No differences were observed for progression-free survival. Toxicity was acceptable, with no major differences between the two therapeutic groups. CONCLUSION:
The combined immunochemotherapy with R-CHOP resulted in a significantly higher response rate and a prolongation of the TTF as compared with chemotherapy alone. Hence, R-CHOP may serve as a new baseline regimen for advanced stage MCL, but needs to be further improved by novel strategies in remission.

38 [109]. Lin, T. Y., H. Y. Zhang, et al. (2005). "[Comparison between R-CHOP regimen and CHOP regimen in treating naive diffuse large B-cell lymphoma in China--a multi-center randomized trail]." Ai Zheng **24**(12): 1421-1426.

BACKGROUND & OBJECTIVE: CHOP regimen is a standard treatment for patients with diffuse large B-cell non-Hodgkin's lymphoma (NHL), and its 5-year overall survival (OS) rate is 30%-40%. Rituximab is a chimeric monoclonal antibody (MoAb) directly against CD20-positive B cells, and has good effect on diffuse large B-cell NHL. Rituximab combined with standard chemotherapy has been approved for treating aggressive B-cell NHL in Europe and the US. This study was to determine efficacy and safety of the combination of Rituximab and CHOP regimen in treating Chinese patients with CD20-positive diffuse large B-cell NHL. METHODS: From Sep. 2003 to Nov. 2004, a total of 63 patients in 9 centers were enrolled. All the patients were randomized into 2 groups: 32 received CHOP regimen alone (CHOP group), and 31 received Rituximab and CHOP regimen (R-CHOP group). All patients signed informed consent. The complete

response rates, overall response rates, and side events of the 2 groups were compared. RESULTS: The complete response rates were similar in R-CHOP and CHOP groups (41.9% vs. 37.5%, P=0.719); the overall response rates were slightly higher in R-CHOP group than in CHOP group (83.8% vs. 65.6%, P=0.096). Disease progression during treatment was reported for 7 (21.9%) patients in CHOP group and 1 (3.2%) patient in R-CHOP group (P=0.026). The occurrence rates of adverse events were similar in R-CHOP and CHOP groups (65.6% vs. 67.7%, P=0.859). The most common adverse event was leukopenia; fever and chills were rather common in R-CHOP group. Clinically relevant toxicity was similar in both groups. CONCLUSION: When compared with standard CHOP alone, the addition of Rituximab to standard CHOP regimen reduces the risk of treatment failure in patients with diffuse large B-cell NHL, and doesn't increase the occurrence of chemotherapy-related adverse events.

39 [122]. Marcus, R., K. Imrie, et al. (2005). "CVP chemotherapy plus rituximab compared with CVP as first-line treatment for advanced follicular lymphoma." Blood **105**(4): 1417-1423.

The combination of cyclophosphamide, vincristine, and prednisone (CVP) is one of several standard treatment options for advanced follicular lymphoma. This, like similar chemotherapeutic regimens, induces response rates of 60% to 80%, with a median response duration of under 2 years. Rituximab, a chimeric monoclonal antibody against CD20, is active in follicular lymphoma, both as monotherapy and in combination with chemotherapy. Previously untreated patients with stages III to IV follicular lymphoma were randomly assigned to receive either 8 cycles of CVP plus rituximab (R-CVP; n = 162) or CVP (n = 159). Overall and complete response rates were 81% and 41% in the R-CVP arm versus 57% and 10% in the CVP arm, respectively (P < .0001). At a median follow-up of 30 months, patients treated with R-CVP had a very significantly prolonged time to progression (median 32 months versus 15 months for CVP; P < .0001). Median time to treatment failure was 27 months in patients receiving R-CVP and 7 months in the CVP arm (P < .0001). Rituximab did not add significantly to the toxicity of CVP. The addition of rituximab to the CVP regimen significantly improves the clinical outcome in patients with previously untreated advanced follicular lymphoma, without increased toxicity.

40 [82]. Marcus, R., K. Imrie, et al. (2008). "Phase III study of R-CVP compared with cyclophosphamide, vincristine, and prednisone alone in patients with previously untreated advanced follicular lymphoma." J Clin Oncol **26**(28): 4579-4586.

PURPOSE: To compare the long-term outcome of patients with previously untreated follicular lymphoma (FL) needing therapy, after treatment with cyclophosphamide, vincristine and prednisone (CVP) versus CVP plus rituximab (R-CVP) and to evaluate the predictive value of known prognostic factors after treatment with R-CVP. PATIENTS AND METHODS: Patients with previously untreated CD20-positive stage III/IV FL were randomly assigned to eight cycles of R-CVP (n = 159) or CVP alone (n = 162). The median follow-up period was 53 months. RESULTS: The primary end point-time to treatment failure (TTF), which included patients without a response after four cycles as an event-was significantly prolonged in patients receiving R-CVP versus CVP (P < .0001). Improvements in all other end points, including overall and complete response rates (P < .0001), time to progression (TTP; P < .0001), response duration (P < .0001), time to next antilymphoma treatment (P < .0001), and overall survival (OS; P = .029; 4-year OS: 83% v 77%;) were achieved with R-CVP versus CVP alone. Univariate analyses demonstrated an improvement in TTP with R-CVP versus CVP irrespective of the Follicular Lymphoma International Prognostic Index (FLIPI) subgroup, the International Prognostic Index (IPI) subgroup, baseline histology, and the presence or absence of B symptoms or bulky disease. By multivariate analysis, FLIPI retains a strong predictive power for TTP in the presence of the trial treatment effect. CONCLUSION: Analysis of all outcome measures, including OS, confirm the benefit of adding R to CVP in the front-line treatment of FL.

41 [58]. Martinelli, G., S. F. Schmitz, et al. (2010). "Long-term follow-up of patients with follicular lymphoma receiving single-agent rituximab at two different schedules in trial SAKK 35/98." J Clin Oncol **28**(29): 4480-4484.

PURPOSE: We report the long-term results of a randomized clinical trial comparing induction therapy with once per week for 4 weeks single-agent rituximab alone versus induction followed by 4 cycles of maintenance therapy every 2 months in patients with follicular lymphoma. PATIENTS AND METHODS: Patients (prior chemotherapy 138; chemotherapy-naive 64) received single-agent rituximab and if nonprogressive, were randomly assigned to no further treatment (observation) or four additional doses of rituximab given at 2-month intervals (prolonged exposure). RESULTS: At a median follow-up of 9.5 years and with all living patients having been observed for at least 5 years, the median event-free survival (EFS) was 13 months for the observation and 24 months for the prolonged exposure arm (P < .001). In the observation arm, patients without events at 8 years were 5%, while in the prolonged exposure arm they were 27%. Of previously untreated patients receiving prolonged treatment after responding to

rituximab induction, at 8 years 45% were still without event. The only favorable prognostic factor for EFS in a multivariate Cox regression was the prolonged rituximab schedule (hazard ratio, 0.59; 95% CI, 0.39 to 0.88; P = .009), whereas being chemotherapy naive, presenting with stage lower than IV, and showing a VV phenotype at position 158 of the Fc-gamma RIIIA receptor were not of independent prognostic value. No long-term toxicity potentially due to rituximab was observed. CONCLUSION: An important proportion of patients experienced long-term remission after prolonged exposure to rituximab, particularly if they had no prior treatment and responded to rituximab induction.

42 [36]. McClanahan, F., T. Hielscher, et al. (2012). "Final results of a randomized trial comparing 1, 3, or 6 infusions of Rituximab plus 6 cycles CHOP provide valuable preliminary data towards a more cost-effective and safer treatment of advanced follicular lymphoma." Am J Hematol **87**(10): E68-71.

43 [77]. Morschhauser, F., J. Radford, et al. (2008). "Phase III trial of consolidation therapy with yttrium-90-ibritumomab tiuxetan compared with no additional therapy after first remission in advanced follicular lymphoma." J Clin Oncol **26**(32): 5156-5164.

PURPOSE: We conducted an international, randomized, phase III trial to evaluate the efficacy and safety of consolidation with yttrium-90 ((90)Y)-ibritumomab tiuxetan in patients with advanced-stage follicular lymphoma in first remission. PATIENTS AND METHODS: Patients with CD20(+) stage III or IV follicular lymphoma, who achieved a complete response (CR)/unconfirmed CR (CRu) or partial response (PR) after first-line induction treatment, were randomly assigned to receive (90)Y-ibritumomab tiuxetan
(rituximab 250 mg/m(2) on day -7 and day 0 followed on day 0 by (90)Y-ibritumomab tiuxetan 14.8 MBq/kg; maximum of 1,184 MBq) or no further treatment (control). The primary end point was progression-free survival (PFS), which was calculated from the time of random assignment. RESULTS: A total of 414 patients (consolidation, n = 208; control, n = 206) were enrolled at 77 centers. (90)Y-ibritumomab tiuxetan consolidation significantly prolonged median PFS (after a median observation time of 3.5 years) in all patients (36.5 v 13.3 months in control arm; hazard ratio [HR] = 0.465; P < .0001) and regardless of whether patients achieved PR (29.3 v 6.2 months in control arm; HR = 0.304; P < .0001) or CR/CRu (53.9 v 29.5 months in control arm; HR = 0.613; P = .0154) after induction treatment. Median PFS with consolidation was prolonged in all Follicular Lymphoma International Prognostic Index risk subgroups. After (90)Y-ibritumomab tiuxetan consolidation, 77% of patients in PR after induction

converted to CR/CRu, resulting in a final CR rate of 87%. The most common toxicity with
(90)Y-ibritumomab tiuxetan was hematologic, and grade 3 or 4 infections occurred in 8% of patients. CONCLUSION: Consolidation of first remission with (90)Y-ibritumomab tiuxetan in advanced-stage follicular lymphoma is highly effective with no unexpected toxicities, prolonging PFS by 2 years and resulting in high PR-to-CR conversion rates regardless of type of first-line induction treatment.

44 [106]. Ogura, M., Y. Morishima, et al. (2006). "Randomized phase II study of concurrent and sequential rituximab and CHOP chemotherapy in untreated indolent B-cell lymphoma." Cancer Sci **97**(4): 305-312.

CHOP combined with rituximab (R-CHOP) is regarded as one of the most effective treatments for indolent B-cell non-Hodgkin lymphoma (B-NHL), however, its optimal combination schedule remains unknown. We performed a randomized phase II study to explore a more promising schedule in untreated, advanced indolent B-NHL. Patients were randomized to receive either six courses of CHOP concurrently with rituximab (Arm C), or six courses of CHOP followed by six courses of weekly rituximab (Arm S). A total of 69 patients received the concurrent (n=34) or sequential (n=35) regimen. Overall response rate (ORR) in Arm C was 94% (95% confidence interval [CI], 79 to 99), including a 66% complete response (CR) compared with 97% (95% CI, 85-100), including a 68% CR in Arm S. Patients in Arm C experienced more grade 4 neutropenia (85%versus 70%) and experienced more grade 3 or greater non-hematological toxicities (21%versus 12%). Both arms were tolerated well. With a median follow-up of 28.2 months, the median progression-free survival (PFS) time was 34.2 months in Arm C, and was not reached in Arm S. R-CHOP is highly effective in untreated indolent B-NHL, either concurrent or in a sequential combination. Both combination schedules deserve further investigation.

45 [25]. Pettengell, R., N. Schmitz, et al. (2013). "Rituximab purging and/or maintenance in patients undergoing autologous transplantation for relapsed follicular lymphoma: a prospective randomized trial from the lymphoma working party of the European group for blood and marrow transplantation." J Clin Oncol **31**(13): 1624-1630.

PURPOSE: The objective of this randomized trial was to assess the efficacy and safety of rituximab as in vivo purging before transplantation and as maintenance treatment immediately after high-dose chemotherapy and autologous stem-cell transplantation (HDC-ASCT) in patients with relapsed follicular lymphoma (FL). PATIENTS AND METHODS: Patients with relapsed FL

who achieved either complete or very good partial remission with salvage chemotherapy were randomly assigned using a factorial design to rituximab purging (P+; 375 mg/m(2) once per week for 4 weeks) or observation (NP) before HDC-ASCT and to maintenance rituximab (M+; 375 mg/m(2) once every 2 months for four infusions) or observation (NM). RESULTS: From October 1999 to April 2006, 280 patients were enrolled. The median age was 51 years (range, 26 to 70 years), and baseline characteristics were well balanced between groups. On average, patients were 44 months (range, 3 to 464 months) from diagnosis, with 79% having received two lines and 15% three lines of prior therapy. Median follow-up was 8.3 years. In contrast to purging, 10-year progression-free survival (PFS) was 48% for P+ and 42% for NP groups (hazard ratio [HR], 0.80; 95% CI, 0.58 to 1.11; P = .18); maintenance had a significant effect on PFS (10-year PFS, 54% for M+ and 37% for NM; HR, 0.66; 95% CI, 0.47 to 0.91; P = .012). Overall survival (OS) was not improved by either rituximab purging or maintenance. CONCLUSION: Rituximab maintenance after HDC-ASCT is safe and significantly prolongs PFS but not OS in patients undergoing transplantation for relapsed FL. Pretransplantation rituximab in vivo purging, even in rituximab-naive patients, failed to improve PFS or OS.

46 [85]. Pfreundschuh, M., A. D. Ho, et al. (2008). "Prognostic significance of maximum tumour (bulk) diameter in young patients with good-prognosis diffuse large-B-cell lymphoma treated with CHOP-like chemotherapy with or without rituximab: an exploratory analysis of the MabThera International Trial Group (MInT) study." Lancet Oncol **9**(5): 435-444.

BACKGROUND: The definition and role of bulky disease in young patients (ie, aged 18-60 years) with good-prognosis diffuse large-B-cell lymphoma (DLBCL), who have been treated with CHOP (cyclophosphamide, doxorubicin, vincristine, and prednisolone)-like chemotherapy with or without rituximab, remain controversial. We aimed to assess the effect of maximum tumour diameter (MTD) in these patients. METHODS: Patients from the MInT (Mabthera International Trial Group) study were eligible. We analysed event-free (EFS) and overall survival (OS) after CHOP-like chemotherapy with or without rituximab, according to MTD, by Martingale residual analyses and Cox regression models. Radiotherapy was given to sites of primary bulky disease according to national standards, and to primary extranodal disease at physician discretion. The primary endpoint was EFS and the secondary endpoint was OS. Analyses were by intention to treat. FINDINGS: Of the 824 patients enrolled in the MInT study, the informed-consent form of one patient was missing, leaving 823 patients evaluable for intention-to-treat analysis. Data on MTD of involved sites were available for 802 patients. Martingale residual analysis showed an

adverse prognostic effect of MTD on EFS and OS, which increased linearly. In a multivariable analysis with MTD as a linear regression variable, the effect of MTD was significant after CHOP-like treatment alone for EFS (hazard ratio 1.090 [95% CI 1.051-1.130], p < 0.0001) and OS (1.119 [1.057-1.184], p = 0.0001), and after CHOP-like treatment and rituximab for OS (1.089 [1.003-1.183], p = 0.043), but not for EFS (1.044 [0.991-1.099], p=0.103). For CHOP-like treatment alone, 3-year EFS ranged from 78.2% (MTD < 5.0 cm, 95% CI 68.3-85.4) to 41.3% (MTD > or = 10.0 cm, 31.8-50.4). For CHOP-like treatment and rituximab, 3-year EFS ranged from 83.2% (MTD < 5.0 cm, 72.8-89.9) to 72.7% (MTD > or = 10.0 cm, 63.8-79.7). With CHOP-like treatment alone, 3-year OS decreased from 92.9% (MTD < 5.0 cm, 84.9-96.8) to 73.5% (MTD > or = 10.0 cm, 63.9-81.0); for CHOP-like treatment and rituximab, 3-year OS decreased from 98.0% (MTD < 5.0 cm, 92.2-99.5) to 85.2% (MTD > or = 10.0 cm, 77.0-90.6). For CHOP-like treatment, any cut-off point between 5.0 cm and 10.0 cm separated two populations with a significant EFS difference (p < 0.0001 for all log-rank tests) and OS difference (p < or = 0.003 for all log-rank tests). For CHOP-like treatment and rituximab, only a cut-off point of 10.0 cm separated two populations with a significant EFS difference (log-rank p = 0.047), but any cut-off point of 6.0 cm or more separated two populations with a significant OS difference (log-rank p values 0.0009-0.037). INTERPRETATION: Rituximab decreased, but did not eliminate the adverse prognostic effect of MTD in young patients with good-prognosis DLBCL. Due to the linear prognostic effect of MTD on outcome, arbitrary cut-off points for bulky disease can be set between 5.0 cm and 10.0 cm, depending on clinical considerations. Based on this study, a cut-off point of 10.0 cm might be a suitable margin in the rituximab era to delineate those patients with bulky disease.

47 [41]. Pfreundschuh, M., E. Kuhnt, et al. (2011). "CHOP-like chemotherapy with or without rituximab in young patients with good-prognosis diffuse large-B-cell lymphoma: 6-year results of an open-label randomised study of the MabThera International Trial (MInT) Group." Lancet Oncol **12**(11): 1013-1022.

BACKGROUND: The MInT study was the first to show improved 3-year outcomes with the addition of rituximab to a CHOP (cyclophosphamide, doxorubicin, vincristine, and prednisone)-like regimen in young patients with good-prognosis diffuse large-B-cell lymphoma. Extended follow-up was needed to establish long-term effects. METHODS: In the randomised open-label MInT study, patients from 18 countries (aged 18-60 years with none or one risk factor according to the age-adjusted International Prognostic Index [IPI], stage II-IV disease or stage I disease with bulk) were randomly assigned to receive six cycles of a CHOP-like chemotherapy

with or without rituximab. Bulky and extranodal sites received additional radiotherapy. Randomisation was done centrally with a computer-based tool and was stratified by centre, bulky disease, age-adjusted IPI, and chemotherapy regimen by use of a modified minimisation algorithm that incorporated a stochastic component. Patients and investigators were not masked to treatment allocation. The primary endpoint was event-free survival. Analyses were by intention to treat. This observational study is a follow-up of the MInT trial, which was stopped in 2003, and is registered at ClinicalTrials.gov, number NCT00400907. FINDINGS: The intention-to-treat population included 410 patients assigned to chemotherapy alone and 413 assigned to chemotherapy plus rituximab. After a median follow-up of 72 months (range 0.03-119), 6-year event-free survival was 55.8% (95% CI 50.4-60.9; 166 events) for patients assigned to chemotherapy alone and 74.3% (69.3-78.6; 98 events) for those assigned to chemotherapy plus rituximab (difference between groups 18.5%, 11.5-25.4, log-rank p<0.0001). Multivariable analyses showed that event-free survival was affected by treatment group, presence of bulky disease, and age-adjusted IPI and that overall survival was affected by treatment group and presence of bulky disease only. After chemotherapy and rituximab, a favourable subgroup (IPI=0, no bulk) could be defined from a less favourable subgroup (IPI=1 or bulk, or both; event-free survival 84.3% [95% CI 74.2-90.7] vs 71.0% [65.1-76.1], log-rank p=0.005). 18 (4.4%, 95% CI 2.6-6.9) second malignancies occurred in the chemotherapy-alone group and 16 (3.9%, 2.2-6.2) in the chemotherapy and rituximab group (Fisher's exact p=0.730). INTERPRETATION: Rituximab added to six cycles of CHOP-like chemotherapy improved long-term outcomes for young patients with good-prognosis diffuse large-B-cell lymphoma. The definition of two prognostic subgroups allows a more refined therapeutic approach to these patients than does assessment by IPI alone. FUNDING: Hoffmann-La Roche.

48 [105]. Pfreundschuh, M., L. Trumper, et al. (2006). "CHOP-like chemotherapy plus rituximab versus CHOP-like chemotherapy alone in young patients with good-prognosis diffuse large-B-cell lymphoma: a randomised controlled trial by the MabThera International Trial (MInT) Group." Lancet Oncol **7**(5): 379-391.

BACKGROUND: The role of rituximab in combination with different CHOP (cyclophosphamide, doxorubicin, vincristine, and prednisone)-like chemotherapy regimens in young patients with good-prognosis diffuse large-B-cell lymphoma remains to be defined. We aimed to compare CHOP-like chemotherapy and rituximab with CHOP-like chemotherapy alone in these patients. METHODS: 824 patients who were from 18

countries; aged 18-60 years; and who had no risk factors or one risk factor according to age-adjusted International Prognostic Index (IPI), stage II-IV disease, or stage I disease with bulk were enrolled. These patients were randomly assigned to six cycles of CHOP-like chemotherapy and rituximab (n=413) or to six cycles of CHOP-like chemotherapy alone (n=411). Bulky and extranodal sites received additional radiotherapy. The primary endpoint was event-free survival; secondary endpoints were response, progression under therapy, progression-free survival, overall survival, and frequency of toxic effects. Analyses were done by intention to treat and per protocol. This trial is registered at http://www.clinicaltrials.gov, NCT 00064116. FINDINGS: After a median follow-up of 34 months (range 0.03-61), patients assigned chemotherapy and rituximab had increased 3-year event-free survival compared with those assigned chemotherapy alone (79% [95% CI 75-83] vs 59% [54-64]; difference between groups 20% [13-27], log-rank p<0.0001), and had increased 3-year overall survival (93% [90-95] vs 84% [80-88]; difference between groups 9% [3-13], log-rank p=0.0001). Event-free survival was affected by treatment group, presence of bulky disease, and age-adjusted IPI: after chemotherapy and rituximab, a favourable subgroup (ie, IPI=0, no bulk) could be defined from a less-favourable subgroup (ie, IPI=1 or bulk, or both). Groups did not differ in the frequency of adverse events. INTERPRETATION: Rituximab added to six cycles of CHOP is an effective treatment for young patients with good-prognosis diffuse large-B-cell lymphoma. The definition of two prognostic subgroups allows for a more refined therapeutic approach for these patients.

49 [30]. Press, O. W., J. M. Unger, et al. (2013). "Phase III randomized intergroup trial of CHOP plus rituximab compared with CHOP chemotherapy plus (131)iodine-tositumomab for previously untreated follicular non-Hodgkin lymphoma: SWOG S0016." J Clin Oncol **31**(3): 314-320.

PURPOSE: Advanced follicular lymphomas (FL) are considered incurable with conventional chemotherapy and there is no consensus on the best treatment approach. Southwest Oncology Group (SWOG) and Cancer and Leukemia Group B compared the safety and efficacy of two immunochemotherapy regimens for FL in a phase III randomized intergroup protocol (SWOG S0016) that enrolled 554 patients with previously untreated, advanced-stage FL between March 1, 2001, and September 15, 2008. PATIENTS AND METHODS: Patients were eligible for the study if they had advanced-stage (bulky stage II, III, or IV) evaluable FL of any grade (1, 2, or 3) and had not received previous therapy. In one arm of the study, patients received six cycles of cyclophosphamide,

doxorubicin, vincristine, and prednisone (CHOP) chemotherapy at 3-week intervals with six doses of rituximab (CHOP-R). In another arm of the study, patients received six cycles of CHOP followed by consolidation with tositumomab/iodine I-131 tositumomab radioimmunotherapy (RIT). RESULTS: After a median follow-up period of 4.9 years, the 2-year estimate of progression-free survival (PFS) was 76% on the CHOP-R arm and 80% on the CHOP-RIT arm (P = .11). The 2-year estimate of overall survival (OS) was 97% on the CHOP-R arm and 93% on the CHOP-RIT arm (P = .08). CONCLUSION: There was no evidence of a significant improvement in PFS comparing CHOP-RIT with CHOP-R. However, PFS and OS were outstanding on both arms of the study. Future studies are needed to determine the potential benefits of combining CHOP-R induction chemotherapy with RIT consolidation and/or extended rituximab maintenance therapy.

50 [92]. Raynaud, P., S. Caulet-Maugendre, et al. (2008). "T-cell lymphoid aggregates in bone marrow after rituximab therapy for B-cell follicular lymphoma: a marker of therapeutic efficacy?" Hum Pathol **39**(2): 194-200.

Rituximab, an anti-CD20 monoclonal antibody, is widely used in the treatment of B-cell lymphoma. Some reports have outlined histologic modifications in bone marrow specimens from patients treated with this antibody, notably the presence of CD3(+) lymphoid aggregates morphologically mimicking residual lymphoma. To gain insight into the significance of such infiltrates, serial BM trephines obtained in 39 patients with B-cell follicular lymphoma treated by rituximab and enrolled in the GOELAMS-GELA intergroup FL2000 protocol were reexamined. The 39 patients were 22 women and 17 men with a median age of 50 years (range, 29-75 years). All pretreatment bone marrow biopsies showed CD20(+) lymphomatous cells. A second biopsy was obtained between 30 and 100 days after the last rituximab injection: 19 (48%) were morphologically diagnosed as negative (no lymphoid infiltrates or only minor lymphoid aggregates) and 20 (51%) as positive because of persistent lymphoid nodules. After immunohistochemical analysis, 13 (33%) cases were reinterpreted as false-positive because of the complete absence of CD20(+) cells, with the lymphoid nodules consisting of CD3(+) and CD5(+) T cells. Most of them also expressed CD4(+), whereas only a few CD8(+) cells were present. Among these 13 false-positive cases, 12 were BCL2-IGH polymerase chain reaction-negative in the bone marrow aspirate at the time of biopsy. The 13th case turned out to be negative in the 18th-month bone marrow aspirate. In all of these cases, lymphoid aggregates had disappeared on bone marrow biopsies performed 18 months after treatment. After a mean follow-up of 4.5 years, 9 of 13

patients were in remission as compared with only 2 among the 7 patients with postrituximab persistent CD20(+) lymphomatous cells. There was no statistically significant difference between this false-positive group of patients and that with negative postrituximab bone marrow regarding sex, age, medullar involvement pattern before treatment, delay between rituximab treatment, and molecular status. Interestingly, we noted a more favorable outcome (70% versus 52% remission) for the false-positive cases, suggesting that these T-cell reactions could be the hallmark of specific antitumoral immunity after rituximab treatment and should be properly investigated.

51 [57]. Rieger, M., A. Osterborg, et al. (2011). "Primary mediastinal B-cell lymphoma treated with CHOP-like chemotherapy with or without rituximab: results of the Mabthera International Trial Group study." Ann Oncol **22**(3): 664-670.

BACKGROUND: The aim of this subgroup analysis of the Mabthera International Trial Group study was to evaluate the impact of chemotherapy and rituximab in primary mediastinal B-cell lymphoma (PMBCL) in comparison to other diffuse large B-cell lymphoma (DLBCL). METHODS: Patients were randomly assigned to six cycles of CHOP-like regimens with or without rituximab. RESULTS: Of 824 patients enrolled, 87 had PMBCL and 627 other types of DLBCL. Rituximab increased the rates of complete remission (unconfirmed) in both PMBCL (from 54% to 80%, P = 0.015) and DLBCL (from 72% to 87%, P < 0.001). In PMBCL, rituximab virtually eliminated progressive disease (PD) (2.5% versus 24%, P < 0.001), whereas without rituximab, PD was more frequent in PMBCL than in DLBCL (24% versus 10%, P = 0.010). With a median observation time of 34 months, 3-year event-free survival (EFS) was improved by rituximab for PMBCL (78% versus 52%, P = 0.012) and for DLBCL (81% versus 61%, P < 0.001). Overall survival benefit was similar for DLBCL (93% versus 85%, P < 0.001) and PMBCL (89% versus 78%, P = 0.158). CONCLUSION: In young patients with PMBCL (age-adjusted International Prognostic Index 0-1), rituximab added to six cycles of CHOP-like chemotherapy increases response rate and EFS to the same extent as other DLBCL. The combination of rituximab with CHOP chemotherapy is an effective treatment in PMBCL with good prognosis features.

52 [5]. Salar, A., I. Avivi, et al. (2014). "Comparison of subcutaneous versus intravenous administration of rituximab as maintenance treatment for follicular lymphoma: results from a two-stage, phase IB study." J Clin Oncol **32**(17): 1782-1791.

PURPOSE: This two-stage phase IB study investigated the pharmacokinetics and safety of subcutaneous (SC) versus intravenous (IV) administration of rituximab as maintenance therapy in follicular lymphoma. PATIENTS AND METHODS: In stage 1 (dose finding), 124 patients who responded to rituximab induction were randomly assigned to SC rituximab (375 mg/m2, 625 mg/m2, or an additional group at 800 mg/m2) or IV rituximab (375 mg/m2). The objective was to determine an SC dose that would yield a rituximab serum trough concentration (Ctrough) in the same range as that of IV rituximab. In stage 2, 154 additional patients were randomly assigned (1:1) to SC rituximab (1,400 mg) or IV rituximab (375 mg/m2) given at 2- or 3-month intervals. The objective was to demonstrate noninferior rituximab Ctrough of SC rituximab relative to IV rituximab 375 mg/m2. RESULTS: Stage 1 data predicted that a fixed dose of 1,400 mg SC rituximab would result in a serum Ctrough in the range of that of IV rituximab. Noninferiority (ie, meeting the prespecified 90% CI lower limit of 0.8) was then confirmed in stage 2, with geometric mean Ctrough SC:Ctrough IV ratios for the 2- and 3-month regimens of 1.24 (90% CI, 1.02 to 1.51) and 1.12 (90% CI, 0.86 to 1.45), respectively. Overall safety profiles were similar between formulations (in stage 2, 79% of patients experienced one or more adverse events in each group). Local administration-related reactions (mainly mild to moderate) occurred more frequently after SC administration. CONCLUSION: The fixed dose of 1,400 mg SC rituximab predicted by using stage 1 results was confirmed to have noninferior Ctrough levels relative to IV rituximab 375 mg/m2 dosing during maintenance, with a comparable safety profile. Additional investigation will be required to determine whether the SC route of administration for rituximab provides equivalent efficacy compared with that of IV administration.

53 [78]. Salles, G., N. Mounier, et al. (2008). "Rituximab combined with chemotherapy and interferon in follicular lymphoma patients: results of the GELA-GOELAMS FL2000 study." Blood **112**(13): 4824-4831.

The FL2000 study was undertaken to evaluate the combination of the anti-CD20 monoclonal antibody rituximab with chemotherapy plus interferon in the first-line treatment of follicular lymphoma patients with a high tumor burden. Patients were randomly assigned to receive either 12 courses of the chemotherapy regimen CHVP (cyclophosphamide, adriamycin, etoposide, and prednisolone) plus interferon-alpha2a (CHVP+I arm) over 18 months or 6 courses of the same chemotherapy regimen combined with 6 infusions of 375 mg/m(2) rituximab and interferon for the same time period (R-CHVP+I arm). After a median follow-up of 5 years, event-free survival estimates were, respectively, 37% (95% confidence interval [CI], 29%-44%) and 53% (95% CI, 45%-60%) in the CHVP+I and R-CHVP+I arm (P =

.001). Five-year overall survival estimates were not statistically different in the CHVP+I (79%; 95% CI, 72%-84%) and R-CHVP+I (84%; 95% CI, 78%-84%) arms. In a multivariate regression analysis, event-free survival was significantly influenced by both the Follicular Lymphoma International Prognostic Index score (hazard ratio = 2.08; 95% CI, 1.6%-2.8%) and the treatment arm (hazard ratio = 0.59; 95% CI, 0.44%-0.78%). With a 5-year follow-up, the combination of rituximab with CHVP+I provides superior disease control in follicular lymphoma patients despite a shorter duration of chemotherapy. This study's clinical trial was registered at the National Institutes of Health website as no. NCT00136552.

54 [6]. Sarkozy, C., J. F. Seymour, et al. (2014). "Rituximab maintenance obviates the poor prognosis associated with circulating lymphoma cells in patients with follicular lymphoma." Blood **123**(17): 2740-2742.

55 [66]. Sparano, J. A., J. Y. Lee, et al. (2010). "Rituximab plus concurrent infusional EPOCH chemotherapy is highly effective in HIV-associated B-cell non-Hodgkin lymphoma." Blood **115**(15): 3008-3016.

Rituximab plus intravenous bolus chemotherapy is a standard treatment for immunocompetent patients with B-cell non-Hodgkin lymphoma (NHL). Some studies have suggested that rituximab is associated with excessive toxicity in HIV-associated NHL, and that infusional chemotherapy may be more effective. We performed a randomized phase 2 trial of rituximab (375 mg/m(2)) given either concurrently before each infusional etoposide, vincristine, doxorubicin, cyclophosphamide, and prednisone (EPOCH) chemotherapy cycle or sequentially (weekly for 6 weeks) after completion of all chemotherapy in HIV-associated NHL. EPOCH consisted of a 96-hour intravenous infusion of etoposide, doxorubicin, and vincristine plus oral prednisone followed by intravenous bolus cyclophosphamide given every 21 days for 4 to 6 cycles. In the concurrent arm, 35 of 48 evaluable patients (73%; 95% confidence interval, 58%-85%) had a complete response. In the sequential arm, 29 of 53 evaluable patients (55%; 95% confidence interval, 41%-68%) had a complete response. The primary efficacy endpoint was met for the concurrent arm only. Toxicity was comparable in the 2 arms, although patients with a baseline CD4 count less than 50/microL had a high infectious death rate in the concurrent arm. We conclude that concurrent rituximab plus infusional EPOCH is an effective regimen for HIV-associated lymphoma.

56 [54]. Tobinai, K., M. Ogura, et al. (2010). "Randomized phase II study of concurrent and sequential combinations of rituximab plus CHOP

(cyclophosphamide, doxorubicin, vincristine and prednisolone) chemotherapy in untreated indolent B-cell non-Hodgkin lymphoma: 7-year follow-up results." Cancer Sci **101**(12): 2579-2585.

Rituximab plus cyclophosphamide, doxorubicin, vincristine and prednisolone (R-CHOP) is one of the most frequently applied initial treatments for indolent B-cell non-Hodgkin lymphoma (B-NHL); however, information on its long-term outcome is limited. Untreated patients in the concurrent arm (Arm C) received six R (375 mg/m(2)) treatments, 2 days prior to each cycle of CHOP, and patients in the sequential arm (Arm S) received 6 weekly R (375 mg/m(2)) treatments following six cycles of CHOP. Sixty-nine patients were randomized but two patients were withdrawn before receiving the protocol treatment. Sixty-five patients (94%) had follicular lymphoma, and 37 (55%) were at low risk, 23 (34%) at intermediate risk and seven (10%) at high risk according to the Follicular Lymphoma International Prognostic Index. We previously reported that the overall response rate (ORR) in Arm C and in Arm S was 94% and 97%, respectively. The median progression-free survival (PFS)/7-year PFS rate in Arm C, Arm S and all 67 assessable patients was 2.4 years/23% (95% confidence interval [CI], 9-40%), 3.8 years/41% (95% CI, 23-57%) and 2.8 years/32% (95% CI, 20-45%), respectively. There was no significant difference between the two arms (P = 0.107). The overall survival (OS) of the 67 patients was 95% at 7 years. In conclusion, R-CHOP is a highly effective initial treatment for untreated indolent B-NHL in terms of ORR and OS; however, its long-term PFS is not good enough either in concurrent or sequential combination, warranting further investigations on post-remission therapy.

57 [102]. van Oers, M. H., R. Klasa, et al. (2006). "Rituximab maintenance improves clinical outcome of relapsed/resistant follicular non-Hodgkin lymphoma in patients both with and without rituximab during induction: results of a prospective randomized phase 3 intergroup trial." Blood **108**(10): 3295-3301.

We evaluated the role of rituximab (R) both in remission induction and maintenance treatment of relapsed/resistant follicular lymphoma (FL). A total of 465 patients were randomized to induction with 6 cycles of cyclophosphamide, doxorubicin, vincristine, and prednisone (CHOP) (every 3 weeks) or R-CHOP (R: 375 mg/m(2) intravenously, day 1). Those in complete remission (CR) or partial remission (PR) were randomized to maintenance with R (375 mg/m(2) intravenously once every 3 months for a maximum of 2 years) or observation. R-CHOP induction yielded an increased overall response rate (CHOP, 72.3%; R-CHOP, 85.1%; P < .001) and CR rate (CHOP, 15.6%; R-CHOP, 29.5%; P < .001). Median progression-free survival (PFS) from first randomization was 20.2 months after CHOP

versus 33.1 months after R-CHOP (hazard ratio [HR], 0.65; P < .001). Rituximab maintenance yielded a median PFS from second randomization of 51.5 months versus 14.9 months with observation (HR, 0.40; P < .001). Improved PFS was found both after induction with CHOP (HR, 0.30; P < .001) and R-CHOP (HR, 0.54; P = .004). R maintenance also improved overall survival from second randomization: 85% at 3 years versus 77% with observation (HR, 0.52; P = .011). This is the first trial showing that in relapsed/resistant FL rituximab maintenance considerably improves PFS not only after CHOP but also after R-CHOP induction.

58 [62]. van Oers, M. H., M. Van Glabbeke, et al. (2010). "Rituximab maintenance treatment of relapsed/resistant follicular non-Hodgkin's lymphoma: long-term outcome of the EORTC 20981 phase III randomized intergroup study." J Clin Oncol **28**(17): 2853-2858.

PURPOSE: In 2006, we published the results of the European Organisation for Research and Treatment of Cancer phase III trial EORTC 20981 on the role of rituximab in remission induction and maintenance treatment of relapsed/resistant follicular lymphoma (FL). At that time, the median follow-up for the maintenance phase was 33 months. Now, we report the long-term outcome of maintenance treatment, with a median follow-up of 6 years. PATIENTS AND METHODS: Overall, 465 patients were randomly assigned to induction with either six cycles of cyclophosphamide, doxorubicin, vincristine, prednisone (CHOP) or rituximab plus CHOP (R-CHOP). Those in complete remission or partial remission after induction (n = 334) were randomly assigned to maintenance treatment with rituximab (375 mg/m(2) intravenously once every 3 months) or observation. RESULTS: Rituximab maintenance significantly improved progression-free survival (PFS) compared with observation (median, 3.7 years v 1.3 years; P < .001; hazard ratio [HR], 0.55), both after CHOP induction (P < .001; HR, 0.37) and R-CHOP (P = .003; HR, 0.69). The 5-year overall survival (OS) was 74% in the rituximab maintenance arm, and it was 64% in the observation arm (P = .07). After progression, a rituximab-containing salvage therapy was given to 59% of patients treated with CHOP followed by observation, compared with 26% after R-CHOP followed by rituximab maintenance. Rituximab maintenance was associated with a significant increase in grades 3 to 4 infections: 9.7% v 2.4% (P = .01). CONCLUSION: With long-term follow-up, we confirm the superior PFS with rituximab maintenance in relapsed/resistant FL. The improvement of OS did not reach statistical significance, possibly because of the unbalanced use of rituximab in post-protocol salvage treatment.

59 [91]. Vellenga, E., W. L. van Putten, et al. (2008). "Rituximab improves the treatment results of DHAP-VIM-DHAP and ASCT in relapsed/progressive aggressive CD20+ NHL: a prospective randomized HOVON trial." Blood **111**(2): 537-543.

We evaluated the role of rituximab during remission induction chemotherapy in relapsed aggressive CD20+ non-Hodgkin lymphoma. Of 239 patients, 225 were evaluable for analysis. Randomized to DHAP (cisplatin-cytarabine-dexamethasone)-VIM (etoposide-ifosfamide-methotrexate)-DHAP (cisplatin-cytarabine-dexamethasone) chemotherapy with rituximab (R; R-DHAP arm) were 119 patients (113 evaluable) and to chemotherapy without rituximab (DHAP arm) 120 patients (112 evaluable). Patients in complete remission (CR) and partial remission (PR) after 2 chemotherapy courses were eligible for autologous stem-cell transplantation. After the second chemotherapy cycle, 75% of the patients in the R-DHAP arm had responsive disease (CR or PR) versus 54% in the DHAP arm (P=.01). With a median follow-up of 24 months, there was a significant difference in failure-free survival (FFS24; 50% vs 24% vs, P<.001), and progression free survival (PFS24; 52% vs 31% P<.002) in favor of the R-DHAP arm. Cox-regression analysis demonstrated a significant effect of rituximab treatment on FFS24 (HR 0.41, 95% confidence interval [CI] 0.29-0.57 versus 0.51, 95% CI 0.37-0.70) and overall-survival (OS24: HR 0.60 [0.41-0.89] vs 0.76 [0.52-1.10]) when adjusted for time since upfront treatment, age, World Health Organization performance status, and secondary age-adjusted international prognostic index. These results demonstrate improved FFS and PFS for relapsed aggressive B-cell NHL if rituximab is added to the re-induction chemotherapy regimen.

60 [16]. Vitolo, U., M. Ladetto, et al. (2013). "Rituximab maintenance compared with observation after brief first-line R-FND chemoimmunotherapy with rituximab consolidation in patients age older than 60 years with advanced follicular lymphoma: a phase III randomized study by the Fondazione Italiana Linfomi." J Clin Oncol **31**(27): 3351-3359.

PURPOSE: To evaluate the efficacy of rituximab maintenance in 60- to 75-year-old patients with advanced follicular lymphoma responding to brief first-line chemoimmunotherapy followed by rituximab consolidation. PATIENTS AND METHODS: A total of 234 treatment-naive 60- to 75-year-old patients began chemoimmunotherapy with four monthly courses of rituximab, fludarabine, mitoxantrone, and dexamethasone (R-FND) followed by four weekly cycles of rituximab consolidation. Of these, 210 patients completed the planned treatment, and 202 responders were randomly assigned to rituximab maintenance (arm A) for 8 months, once

every 2 months for a total of four doses, or to observation (arm B). RESULTS: Median ages in arms A and B were 66 and 65 years, respectively. After induction and consolidation therapy, the overall response rate was 86%, with 69% complete remissions (CR). After a 42-month median follow-up from diagnosis, 3-year progression-free survival (PFS; the primary end point) and overall survival (OS) were 66% (95% CI, 59% to 72%) and 89% (95% CI, 85% to 93%), respectively. After randomization, 2-year PFS was 81% for rituximab maintenance versus 69% for observation, with a hazard ratio of 0.74 (95% CI, 0.45 to 1.21; P = .226), although this was not statistically significant. No differences between the two arms were detected for OS. Overall, the regimen was well-tolerated. The most frequent grade 3 to 4 toxicity was neutropenia (25% of treatment courses), with 13 infections. Two toxic deaths (0.8%) occurred during induction treatment. CONCLUSION: A brief R-FND induction plus rituximab consolidation achieved excellent results with high CR and PFS rates, supporting the feasibility of this regimen in patients older than 60 years. A short rituximab maintenance did not achieve a statistically significant PFS improvement over observation.

61 [26]. Vose, J. M., S. Carter, et al. (2013). "Phase III randomized study of rituximab/carmustine, etoposide, cytarabine, and melphalan (BEAM) compared with iodine-131 tositumomab/BEAM with autologous hematopoietic cell transplantation for relapsed diffuse large B-cell lymphoma: results from the BMT CTN 0401 trial." J Clin Oncol **31**(13): 1662-1668.

PURPOSE: This clinical trial evaluated standard-dose radioimmunotherapy with a chemotherapy-based transplantation regimen followed by autologous hematopoietic cell transplantation versus rituximab with the same regimen in patients with relapsed diffuse large B-cell lymphoma (DLBCL). PATIENTS AND METHODS: Patients with chemotherapy-sensitive persistent or relapsed DLBCL were randomly assigned to receive iodine-131 tositumomab (dosimetric dose of 5 mCi on day -19 and therapeutic dose of 0.75 Gy on day -12), carmustine 300 mg/m(2) (day -6), etoposide 100 mg/m(2) twice daily (days -5 to -2), cytarabine 100 mg/m(2) twice daily (days -5 to -2), and melphalan 140 mg/m(2) (day -1; B-BEAM) or rituximab
375 mg/m(2) on days -19 and -12 and the same chemotherapy regimen (R-BEAM). RESULTS: Two hundred twenty-four patients were enrolled, with 113 patients randomly assigned to R-BEAM and 111 patients assigned to B-BEAM. Two-year progression-free survival (PFS) rates, the primary end point, were 48.6% (95% CI, 38.6% to 57.8%) for R-BEAM and 47.9% (95% CI, 38.2% to 57%; P = .94) for B-BEAM, and the 2-year overall survival (OS) rates were 65.6% (95% CI, 55.3% to 74.1%) for R-BEAM and 61% (95% CI, 50.9% to

69.9%; P = .38) for B-BEAM. The 100-day treatment-related mortality rates were 4.1% (95% CI, 0.2% to 8.0%) for R-BEAM and 4.9% (95% CI, 0.8% to 9.0%; P = .97) for B-BEAM. The maximum mucositis score was higher in the B-BEAM arm (0.72) compared with the R-BEAM arm (0.31; P < .001). CONCLUSION: The B-BEAM and R-BEAM regimens produced similar 2-year PFS and OS rates for patients with chemotherapy-sensitive relapsed DLBCL. No differences in toxicities other than mucositis were noted.

62 [42]. Watanabe, T., K. Tobinai, et al. (2011). "Phase II/III study of R-CHOP-21 versus R-CHOP-14 for untreated indolent B-cell non-Hodgkin's lymphoma: JCOG 0203 trial." J Clin Oncol **29**(30): 3990-3998.

PURPOSE: Rituximab with cyclophosphamide, doxorubicin, vincristine, and prednisone (R-CHOP) is one of the most effective front-line therapies to treat indolent B-cell lymphoma. Granulocyte colony-stimulating factor (G-CSF), which potentiates antibody-dependent rituximab cytotoxicity, is used to shorten CHOP intervals. To improve progression-free survival (PFS) in patients treated with R-CHOP as the primary end point, we conducted a phase III study. PATIENTS AND METHODS: Patients with untreated stages III to IV indolent B-cell lymphoma were randomly assigned to six cycles of R-CHOP every 3 weeks (R-CHOP-21) or every 2 weeks (R-CHOP-14) with G-CSF. Maintenance rituximab was not allowed. RESULTS: Three hundred patients were enrolled. At the median follow-up time of 5.2 years, there was no significant difference in PFS between arms for the 299 eligible patients; the median was 3.7 (R-CHOP-21) v 4.7 (R-CHOP-14) years, 57% v 58% at 3 years, and 41% v 43% at 6 years, respectively (hazard ratio [HR], 0.92; 95% CI, 0.68 to 1.25; one-sided P = .30). The median overall survival (OS) time was not reached in either arm, and there was no significant difference (6-year OS: 87% [R-CHOP-21] v 88% [R-CHOP-14]; HR, 1.15; 95% CI, 0.57 to 2.30; one-sided P = .65). Although grade 4 neutropenia and grade 3 infections were more frequent in the R-CHOP-21 group, R-CHOP was feasible in both arms. CONCLUSION: The R-CHOP dose-dense strategy failed to improve PFS of patients with untreated indolent B-cell lymphoma. Further improvement of first-line treatment or investigations on postremission therapy following R-CHOP should be explored.

63 [49]. Wierda, W. G., T. J. Kipps, et al. (2011). "Chemoimmunotherapy with O-FC in previously untreated patients with chronic lymphocytic leukemia." Blood **117**(24): 6450-6458.

We conducted an international phase 2 trial to evaluate 2 dose levels of ofatumumab, a human CD20 mAb, combined with fludarabine and cyclophosphamide (O-FC) as frontline therapy for chronic lymphocytic leukemia (CLL). Patients with active CLL were randomized to ofatumumab

500 mg (n = 31) or 1000 mg (n = 30) day 1, with fludarabine 25 mg/m(2) and cyclophosphamide 250 mg/m(2) days 2-4, course 1; days 1-3, courses 2-6; every 4 weeks for 6 courses. The first ofatumumab dose was 300 mg for both cohorts. The median age was 56 years; 13% of patients had a 17p deletion; 64% had beta2-microglobulin > 3.5 mg/L. Based on the 1996 National Cancer Institute Working Group (NCI-WG) guidelines, the complete response (CR) rate as assessed by an independent review committee was 32% for the 500-mg and 50% for the 1000-mg cohort; the overall response (OR) rate was 77% and 73%, respectively. Based on univariable regression analyses, beta2-microglobulin and the number of O-FC courses were significantly correlated (P < .05) with CR and OR rates and progression-free survival (PFS). The most frequent Common Terminology Criteria (CTC) grade 3-4 investigator-reported adverse events were neutropenia (48%), thrombocytopenia (15%), anemia (13%), and infection (8%). O-FC is active and safe in treatment-naive patients with CLL, including high-risk patients. This trial was registered at www.clinicaltrials.gov as NCT00410163.

64 [121]. Williams, M. E. (2004). "ECOG 4402: randomized phase III-trial comparing two different rituximab dosing regimens for patients with low tumor burden indolent non-Hodgkin's lymphoma." Curr Hematol Rep **3**(6): 395-396.

65 [64]. Winter, J. N., S. Li, et al. (2010). "Expression of p21 protein predicts clinical outcome in DLBCL patients older than 60 years treated with R-CHOP but not CHOP: a prospective ECOG and Southwest Oncology Group correlative study on E4494." Clin Cancer Res **16**(8): 2435-2442.

PURPOSE: To prospectively investigate the prognostic significance of p21 and p53 expression in diffuse large B-cell lymphoma in the context of the U.S. Intergroup trial comparing conventional cyclophosphamide, doxorubicin, vincristine, and prednisone (CHOP) chemotherapy to rituximab-CHOP (R-CHOP) induction, with or without maintenance rituximab. EXPERIMENTAL DESIGN: Immunohistochemical staining of 197 paraffin-embedded biopsy specimens was scored by an independent panel of experts. RESULTS: The cyclin-dependent kinase inhibitor, p21, was expressed in 55% of cases examined. In a multivariable analysis adjusting for International Prognostic Index score and BCL2 status, p21 expression was a significant, independent, favorable predictive factor for failure-free survival (relative risk, 0.3; P = 0.001) and overall survival (relative risk, 0.3; P = 0.003) for patients treated with R-CHOP. Expression of p21 was not predictive of outcome for CHOP-treated patients. Only p21-positive cases benefited from the addition of rituximab to CHOP. Among p21-positive patients, treatment with R-CHOP was associated with a higher failure-free

survival rate at 5 years compared with CHOP (61% versus 24%; P = 0.01). In contrast, no significant differences were detected in failure-free survival according to treatment arm for p21-negative patients. Expression of p53, alone or in combination with p21, did not predict for outcome in univariable or multivariable analyses. CONCLUSIONS: In this study, p21 protein expression emerged as an important independent predictor of a favorable clinical outcome when rituximab was added to CHOP therapy. These data suggest that rituximab-related effects on lymphoma survival pathways may be functionally linked to p21 activity.

66 [81]. Witzens-Harig, M., M. Reiz, et al. (2009). "Quality of life during maintenance therapy with the anti-CD20 antibody rituximab in patients with B cell non-Hodgkin's lymphoma: results of a prospective randomized controlled trial." Ann Hematol **88**(1): 51-57.

The introduction of rituximab into the primary treatment of malignant lymphomas of the B cell lineage has had a major impact on the management of these diseases. In addition, prolonged exposure to rituximab as maintenance therapy has been beneficial in patients with follicular lymphoma and mantle cell lymphoma. For the individual patient, the effect of any prolonged antitumor therapy on the quality of life (QoL) is a very important question. However, so far, the question whether rituximab maintenance therapy may impair QoL in patients with non-Hodgkin's lymphoma remains unanswered. To investigate this subject, we have performed a prospective randomized trial of rituximab maintenance therapy (8 cycles rituximab 375 mg/m(2) every 3 months) versus observation in patients with CD20+B cell non-Hodgkin's lymphoma in our institution. Between July 2002 and December 2005, 122 patients were included into the trial. QoL was assessed with the standardized questionnaires EORTC-QLQ-C30, EuroQol-5D, and EuroQol-5D (VAS) in 91 patients. After statistical analysis with the exact Wilcoxon rank sum test, we found no significant differences of the QoL between the rituximab treatment group and the observation group. We conclude that rituximab maintenance therapy seems to be safe and does not impair quality of life in this patient population.

67 [98]. Witzig, T. E., A. Molina, et al. (2007). "Long-term responses in patients with recurring or refractory B-cell non-Hodgkin lymphoma treated with yttrium 90 ibritumomab tiuxetan." Cancer **109**(9): 1804-1810.

BACKGROUND: Radioimmunotherapy with radiolabeled monoclonal antibodies to CD20 produces a high response rate in patients with recurring non-Hodgkin lymphoma (NHL), but the durability of those remissions is not well defined. METHODS: Data on patients with recurring

NHL treated with yttrium Y 90 ibritumomab tiuxetan in 4 clinical trials were reviewed to identify patients with a long-term response, defined as a time to progression of 12 months or longer. RESULTS: Long-term responses were seen in 37% (78/211) of patients. At a median follow-up of 53.5 months (range, 12.7-88.9) the median duration of response was 28.1 months and the median time to progression was 29.3 months. A third of these patients had been treated with at least 3 previous therapies, and 37% of them had not responded to their last therapy. The findings in patients with follicular lymphoma (n=59) were similar to those in the overall population of long-term responders. The estimated overall survival at 5 years was 53% for all patients treated with 90Y ibritumomab tiuxetan and 81% for long-term responders. CONCLUSIONS: A single dose of 90Y ibritumomab tiuxetan can produce durable responses and prolonged overall survival in a substantial number of patients in whom previous therapies have failed.

68 [63]. Ziepert, M., D. Hasenclever, et al. (2010). "Standard International prognostic index remains a valid predictor of outcome for patients with aggressive CD20+ B-cell lymphoma in the rituximab era." J Clin Oncol **28**(14): 2373-2380.

PURPOSE: The International Prognostic Index (IPI) is widely used for risk stratification of patients with aggressive B-cell lymphoma. The introduction of rituximab has markedly improved outcome, and R-CHOP (rituximab + cyclophosphamide, doxorubicin, vincristine, prednisone) has become the standard treatment for CD20(+) diffuse large B-cell lymphoma. To investigate whether the IPI has maintained its power for risk stratification when rituximab is combined with CHOP, we analyzed the prognostic relevance of IPI in three prospective clinical trials. PATIENTS AND METHODS: In total, 1,062 patients treated with rituximab were included (MabThera International Trial [MInT], 380 patients; dose-escalated regimen of cyclophosphamide, doxorubicin, vincristine, etoposide, and prednisone (MegaCHOEP) trial, 72 patients; CHOP + rituximab for patients older than age 60 years [RICOVER-60] trial, 610 patients). A multivariate proportional hazards modeling was performed for single IPI factors under rituximab on event-free, progression-free, and overall survival. RESULTS: IPI score was significant for all three end points. Rituximab significantly improved treatment outcome within each IPI group resulting in a quenching of the Kaplan-Meier estimators. However, IPI was a significant prognostic factor in all three end points and the ordering of the IPI groups remained valid. The relative risk estimates of single IPI factors and their order in patients treated with R-CHOP were similar to those found with CHOP. CONCLUSION: The effects of rituximab were superimposed on the effects of CHOP with no interactions between chemotherapy and antibody

therapy. These results demonstrate that the IPI is still valid in the R-CHOP era.

69 [33]. Zinzani, P. L., N. K. Khuageva, et al. (2012). "Bortezomib plus rituximab versus rituximab in patients with high-risk, relapsed, rituximab-naive or rituximab-sensitive follicular lymphoma: subgroup analysis of a randomized phase 3 trial." J Hematol Oncol **5**: 67.

BACKGROUND: The randomized phase 3 LYM3001 trial in relapsed follicular lymphoma (FL) demonstrated higher overall (ORR) and complete response
(CR) rates and prolonged progression-free survival (PFS) with bortezomib-rituximab versus rituximab. We report findings in high-risk patients (FL International Prognostic Index [FLIPI] score >/=3, and high tumor burden by modified Groupe d'Etude des Lymphomas Folliculaires [GELF] criteria). METHODS: Patients aged >/=18 years with grade 1/2 FL, >/=1 measurable lesion, and documented relapse or progression following prior therapy, rituximab-naive or rituximab-sensitive, were enrolled at 164 centers in 29 countries across Europe, the Americas, and Asia-Pacific. Patients were randomized (1:1) to five 5-week cycles of
bortezomib-rituximab (bortezomib 1.6 mg/m2, days 1, 8, 15, and 22, all cycles; rituximab 375 mg/m2, days 1, 8, 15, and 22, cycle 1, and day 1, cycles 2-5; N=336) or rituximab alone (N=340). Randomization was stratified by FLIPI score, prior rituximab, time since last dose of anti-lymphoma therapy, and geographical region. The primary endpoint of the study was PFS. RESULTS: 103 bortezomib-rituximab and 98 rituximab patients had high-risk FL. The ORR was 59% versus 37% (p=0.002), the CR/CRu rate was 13% versus 6% (p=0.145), and the durable response rate was 45% versus
26% (p=0.008) with bortezomib-rituximab versus rituximab. Median PFS was 9.5 versus 6.7 months (hazard ratio [HR] 0.667, p=0.012) with bortezomib-rituximab versus rituximab; median time to progression was 10.9 versus 6.8 months (HR 0.656, p=0.009); median time to next anti-lymphoma treatment was 14.8 versus 9.1 months (HR 0.762, p=0.103); and the 1-year Overall Survival rate was 83.1% versus 76.6%. Overall, 51% of bortezomib-rituximab and 32% of rituximab patients reported grade >/=3 adverse events, including neutropenia (18%, 6%), anemia (4%, 5%), diarrhea (8%, 0%), thrombocytopenia (5%, 2%), and sensory neuropathy (1%, 0%). CONCLUSIONS: High-risk FL patients treated with bortezomib-rituximab had significantly higher ORR and longer PFS than patients receiving rituximab alone, with greater clinical benefit than in the overall study population; additional toxicity was acceptable and did not affect

treatment feasibility. TRIAL REGISTRATION: The phase 3 LYM3001 trial is registered with ClinicalTrials.gov, with the identifier NCT00312845.

70 [125]. Zinzani, P. L., A. Pulsoni, et al. (2004). "Fludarabine plus mitoxantrone with and without rituximab versus CHOP with and without rituximab as front-line treatment for patients with follicular lymphoma." J Clin Oncol **22**(13): 2654-2661.

PURPOSE: Promising new therapeutic options for follicular lymphoma (FL) include fludarabine plus mitoxantrone (FM) and the mouse/human anti-CD20 antibody, rituximab. We performed a randomized comparative trial of FM with cyclophosphamide, doxorubicin, vincristine, and prednisone (CHOP) front-line chemotherapy with and without sequential rituximab. PATIENTS AND METHODS: All previously untreated CD20(+) FL patients presenting in 15 Italian cooperative institutions from October 1999 were randomly allocated to FM or CHOP. Following clinical or molecular restaging, patients in complete remission (CR) with bcl-2/IgH negativity (CR(-)) received no further treatment; those in CR with bcl-2/IgH positivity (CR(+)) received rituximab, as did those in partial remission (PR) with bcl-2/IgH negativity (PR(-)) or positivity (PR(+)); nonresponders (NR subgroup) were off study. RESULTS: After chemotherapy, the FM arm achieved higher rates of CR (68% [49 of 72 patients] v 42% [29 of 68 patients]; P =.003) and CR(-) (39% [28 of 72 patients] v 13 of 68 patients [19%]; P =.001). Rituximab elicited CR(-) in 55 of 95 treated patients (58%). The final CR(-) rate was higher in the FM arm (71% [51 of 72 patients] v 51% [35 of 68 patients]; P =.01). However, with a median follow-up of 19 months (range, 9 to 37 months), no statistically significant difference was found among the various study arms in terms of both progression-free (PFS) and overall survival (OS). CONCLUSION: These results indicate that FM is superior to CHOP for front-line treatment of FL and that rituximab is an effective sequential treatment option. However, they also confirm that this superiority is unlikely to translate into either better PFS or OS.

71 [28]. Zucca, E., A. Conconi, et al. (2013). "Addition of rituximab to chlorambucil produces superior event-free survival in the treatment of patients with extranodal marginal-zone B-cell lymphoma: 5-year analysis of the IELSG-19 Randomized Study." J Clin Oncol **31**(5): 565-572.

PURPOSE: Apart from localized gastric disease, there is no consensus on standard initial treatment of mucosa-associated lymphoid tissue lymphoma. The IELSG-19 study (Randomized Trial of Chlorambucil Versus Chlorambucil Plus Rituximab Versus Rituximab in MALT Lymphoma) was launched to compare chlorambucil alone versus chlorambucil plus rituximab in patients not previously given systemic anticancer therapy. PATIENTS AND METHODS: Patients not responding to or not suitable for

local therapy were eligible. In arm A, chlorambucil was given daily 6 mg/m(2) orally (PO) for 6 weeks. Responding patients and those with stable disease continued to be given daily chlorambucil 6 mg/m(2) PO for 14 consecutive days every 28 days for four cycles. In arm B, intravenous rituximab 375 mg/m(2) per day was added on days 1, 8, 15, 22, 56, 84, 112, and 140. After completion of the planned accrual, the protocol was amended to introduce a third arm with rituximab alone. We report the planned final analysis of the first two arms (113 patients in arm A and 114 in arm B). RESULTS: At a median follow-up of 62 months, the 5-year event-free survival
(EFS) was significantly better for the patients treated in arm B (68% v 50%; P = .002) who, despite similar overall response rates (90% v 87%), achieved a higher complete remission rate (78% v 65%; P = .025). Progression-free survival was also improved but it did not reach statistical significance (P = .057). Five-year overall survival (OS) was 89% in both arms. Both treatments were well tolerated without unexpected toxicities. CONCLUSION: Both treatments were active; the better response rate and EFS obtained with the addition of rituximab did not translate into improved OS.

TOPIC: Lymphoma + checkpoint blockade

1 [128]. Armand, P., A. Nagler, et al. (2013). "Disabling immune tolerance by programmed death-1 blockade with pidilizumab after autologous hematopoietic stem-cell transplantation for diffuse large B-cell lymphoma: results of an international phase II trial." J Clin Oncol **31**(33): 4199-4206.

PURPOSE: The Programmed Death-1 (PD-1) immune checkpoint pathway may be usurped by tumors, including diffuse large B-cell lymphoma (DLBCL), to evade immune surveillance. The reconstituting immune landscape after autologous hematopoietic stem-cell transplantation (AHSCT) may be particularly favorable for breaking immune tolerance through PD-1 blockade. PATIENTS AND METHODS: We conducted an international phase II study of pidilizumab, an anti-PD-1 monoclonal antibody, in patients with DLBCL undergoing AHSCT, with correlative studies of lymphocyte subsets. Patients received three doses of pidilizumab beginning 1 to 3 months after AHSCT. RESULTS: Sixty-six eligible patients were treated. Toxicity was mild. At 16 months after the first treatment, progression-free survival (PFS) was 0.72 (90% CI, 0.60 to 0.82), meeting the primary end point. Among the 24 high-risk patients who remained positive on positron emission tomography after salvage chemotherapy, the

16-month PFS was 0.70 (90% CI, 0.51 to 0.82). Among the 35 patients with measurable disease after AHSCT, the overall response rate after pidilizumab treatment was 51%. Treatment was associated with increases in circulating lymphocyte subsets including PD-L1E-bearing lymphocytes, suggesting an on-target in vivo effect of pidilizumab. CONCLUSION: This is the first demonstration of clinical activity of PD-1 blockade in DLBCL. Given these results, PD-1 blockade after AHSCT using pidilizumab may represent a promising therapeutic strategy in this disease.

2 [127]. Westin, J. R., F. Chu, et al. (2014). "Safety and activity of PD1 blockade by pidilizumab in combination with rituximab in patients with relapsed follicular lymphoma: a single group, open-label, phase 2 trial." Lancet Oncol **15**(1): 69-77.

BACKGROUND: Endogenous or iatrogenic antitumour immune responses can improve the course of follicular lymphoma, but might be diminished by immune checkpoints in the tumour microenvironment. These checkpoints might include effects of programmed cell death 1 (PD1), a co-inhibitory receptor that impairs T-cell function and is highly expressed on intratumoral T cells. We did this phase 2 trial to investigate the activity of pidilizumab, a humanised anti-PD1 monoclonal antibody, with rituximab in patients with relapsed follicular lymphoma. METHODS: We did this open-label, non-randomised trial at the University of Texas MD Anderson Cancer Center (Houston, TX, USA). Adult (>/=18 years) patients with rituximab-sensitive follicular lymphoma relapsing after one to four previous therapies were eligible. Pidilizumab was administered at 3 mg/kg intravenously every 4 weeks for four infusions, plus eight optional infusions every 4 weeks for patients with stable disease or better. Starting 17 days after the first infusion of pidilizumab, rituximab was given at 375 mg/m(2) intravenously weekly for 4 weeks. The primary endpoint was the proportion of patients who achieved an objective response (complete response plus partial response according to Revised Response Criteria for Malignant Lymphoma). Analysis was by intention to treat. This trial is registered with ClinicalTrials.gov, number NCT00904722. FINDINGS: We enrolled 32 patients between Jan 13, 2010, and Jan 20, 2012. Median follow-up was 15.4 months (IQR 10.1-21.0). The combination of pidilizumab and rituximab was well tolerated, with no autoimmune or treatment-related adverse events of grade 3 or 4. The most common adverse events of grade 1 were anaemia (14 patients) and fatigue (13 patients), and the most common adverse event of grade 2 was respiratory infection (five patients). Of the 29 patients evaluable for activity, 19 (66%) achieved an objective response: complete responses were noted in 15 (52%) patients and partial responses in four (14%). INTERPRETATION: The combination of pidilizumab

plus rituximab is well tolerated and active in patients with relapsed follicular lymphoma. Our results suggest that immune checkpoint blockade is
worthy of further study in follicular lymphoma. FUNDING: National Institutes of Health, Leukemia and Lymphoma Society, Cure Tech, and University of Texas MD Anderson Cancer Center.

TOPIC: Lymphoma + chimeric antigen receptor (OR) CAR (OR) CART

1 [134]. Jensen, M. C., L. Popplewell, et al. (2010). "Antitransgene rejection responses contribute to attenuated persistence of adoptively transferred CD20/CD19-specific chimeric antigen receptor redirected T cells in humans." Biol Blood Marrow Transplant **16**(9): 1245-1256.

Immunotherapeutic ablation of lymphoma is a conceptually attractive treatment strategy that is the subject of intense translational research. Cytotoxic T lymphocytes (CTLs) that are genetically modified to express CD19- or CD20-specific, single-chain antibody-derived chimeric antigen receptors (CARs) display HLA-independent antigen-specific recognition/killing of lymphoma targets. Here, we describe our initial experience in applying CAR-redirected autologous CTL adoptive therapy to patients with recurrent lymphoma. Using plasmid vector electrotransfer/drug selection systems, cloned and polyclonal CAR(+) CTLs were generated from autologous peripheral blood mononuclear cells and expanded in vitro to cell numbers sufficient for clinical use. In 2 FDA-authorized trials, patients with recurrent diffuse large cell lymphoma were treated with cloned CD8(+) CTLs expressing a CD20-specific CAR (along with NeoR) after autologous hematopoietic stem cell transplantation, and patients with refractory follicular lymphoma were treated with polyclonal T cell preparations expressing a CD19-specific CAR (along with HyTK, a fusion of hygromycin resistance and HSV-1 thymidine kinase suicide genes) and low-dose s.c. recombinant human interleukin-2. A total of 15 infusions were administered (5 at 10(8)cells/m(2), 7 at 10(9)cells/m(2), and 3 at 2 x 10(9)cells/m(2)) to 4 patients. Overt toxicities attributable to CTL administration were not observed; however, detection of transferred CTLs in the circulation, as measured by quantitative polymerase chain reaction, was short (24 hours to 7 days), and cellular antitransgene immune rejection responses were noted in 2 patients. These studies reveal the primary barrier to therapeutic efficacy is limited persistence, and provide the rationale to prospectively define T cell populations intrinsically programmed for survival after adoptive

transfer and to modulate the immune status of recipients to prevent/delay antitransgene rejection responses.

2 [132]. Kebriaei, P., H. Huls, et al. (2012). "Infusing CD19-directed T cells to augment disease control in patients undergoing autologous hematopoietic stem-cell transplantation for advanced B-lymphoid malignancies." Hum Gene Ther **23**(5): 444-450.

Limited curative treatment options exist for patients with advanced B-lymphoid malignancies, and new therapeutic approaches are needed to augment the efficacy of hematopoietic stem-cell transplantation (HSCT). Cellular therapies, such as adoptive transfer of T cells that are being evaluated to target malignant disease, use mechanisms independent of chemo- and radiotherapy with nonoverlapping toxicities. Gene therapy is employed to generate tumor-specific T cells, as specificity can be redirected through enforced expression of a chimeric antigen receptor (CAR) to achieve antigen recognition based on the specificity of a monoclonal antibody. By combining cell and gene therapies, we have opened a new Phase I protocol at the MD Anderson Cancer Center (Houston, TX) to examine the safety and feasibility of administering autologous genetically modified T cells expressing a CD19-specific CAR (capable of signaling through chimeric CD28 and CD3-zeta) into patients with high-risk B-lymphoid malignancies undergoing autologous HSCT. The T cells are genetically modified by nonviral gene transfer of the Sleeping Beauty system and CAR(+) T cells selectively propagated in a CAR-dependent manner on designer artificial antigen-presenting cells. The results of this study will lay the foundation for future protocols including CAR(+) T-cell infusions derived from allogeneic sources.

3 [130]. Kochenderfer, J. N., M. E. Dudley, et al. (2013). "Donor-derived CD19-targeted T cells cause regression of malignancy persisting after allogeneic hematopoietic stem cell transplantation." Blood **122**(25): 4129-4139.

New treatments are needed for B-cell malignancies persisting after allogeneic hematopoietic stem cell transplantation (alloHSCT). We conducted a clinical trial of allogeneic T cells genetically modified to express a chimeric antigen receptor (CAR) targeting the B-cell antigen CD19. T cells for genetic modification were obtained from each patient's alloHSCT donor. All patients had malignancy that persisted after alloHSCT and standard donor lymphocyte infusions (DLIs). Patients did not receive chemotherapy prior to the CAR T-cell infusions and were not lymphocyte depleted at the time of the infusions. The 10 treated patients received a single infusion of allogeneic anti-CD19-CAR T cells. Three patients had regressions of their malignancies. One patient with chronic lymphocytic

leukemia (CLL) obtained an ongoing complete remission after treatment with allogeneic anti-CD19-CAR T cells, another CLL patient had tumor lysis syndrome as his leukemia dramatically regressed, and a patient with mantle cell lymphoma obtained an ongoing partial remission. None of the 10 patients developed graft-versus-host disease (GVHD). Toxicities included transient hypotension and fever. We detected cells containing the anti-CD19-CAR gene in the blood of 8 of 10 patients. These results show for the first time that donor-derived allogeneic anti-CD19-CAR T cells can
cause regression of B-cell malignancies resistant to standard DLIs without causing GVHD.

4 [131]. Kochenderfer, J. N., M. E. Dudley, et al. (2012). "B-cell depletion and remissions of malignancy along with cytokine-associated toxicity in a clinical trial of anti-CD19 chimeric-antigen-receptor-transduced T cells." Blood **119**(12): 2709-2720.

We conducted a clinical trial to assess adoptive transfer of T cells genetically modified to express an anti-CD19 chimeric Ag receptor (CAR). Our clinical protocol consisted of chemotherapy followed by an infusion of anti-CD19-CAR-transduced T cells and a course of IL-2. Six of the 8 patients treated on our protocol obtained remissions of their advanced, progressive B-cell malignancies. Four of the 8 patients treated on the protocol had long-term depletion of normal polyclonal CD19(+) B-lineage cells. Cells containing the anti-CD19 CAR gene were detected in the blood of all patients. Four of the 8 treated patients had prominent elevations in serum levels of the inflammatory cytokines IFNgamma and TNF. The severity of acute toxicities experienced by the patients correlated with serum IFNgamma and TNF levels. The infused anti-CD19-CAR-transduced T cells were a possible source of these inflammatory cytokines because we demonstrated peripheral blood T cells that produced TNF and IFNgamma ex vivo in a CD19-specific manner after anti-CD19-CAR-transduced T-cell infusions. Anti-CD19-CAR-transduced T cells have great promise to improve the treatment of B-cell malignancies because of a potent ability to eradicate CD19(+) cells in vivo; however, reversible cytokine-associated toxicities occurred after CAR-transduced T-cell infusions.

5 [133]. Kochenderfer, J. N., W. H. Wilson, et al. (2010). "Eradication of B-lineage cells and regression of lymphoma in a patient treated with autologous T cells genetically engineered to recognize CD19." Blood **116**(20): 4099-4102.

Adoptive transfer of genetically modified T cells is an attractive approach for generating antitumor immune responses. We treated a patient with

advanced follicular lymphoma by administering a preparative chemotherapy regimen followed by autologous T cells genetically engineered to express a chimeric antigen receptor (CAR) that recognized the B-cell antigen CD19. The patient's lymphoma underwent a dramatic regression, and B-cell precursors were selectively eliminated from the patient's bone marrow after infusion of anti-CD19-CAR-transduced T cells. Blood B cells were absent for at least 39 weeks after anti-CD19-CAR-transduced T-cell infusion despite prompt recovery of other blood cell counts. Consistent with eradication of B-lineage cells, serum immunoglobulins decreased to very low levels after treatment. The prolonged and selective elimination of B-lineage cells could not be attributed to the chemotherapy that the patient received and indicated antigen-specific eradication of B-lineage cells. Adoptive transfer of anti-CD19-CAR-expressing T cells is a promising new approach for treating B-cell malignancies. This study is registered at www.clinicaltrials.gov as #NCT00924326.

6 [129]. Xu, Y., M. Zhang, et al. (2014). "Closely related T-memory stem cells correlate with in vivo expansion of CAR.CD19-T cells and are preserved by IL-7 and IL-15." Blood **123**(24): 3750-3759.

Adoptive transfer of T lymphocytes expressing a CD19-specific chimeric antigen receptor (CAR.CD19) induces complete tumor regression in patients with lymphoid malignancies. Although in vivo persistence of CAR-T cells correlates with clinical responses, it remains unknown whether specific cell subsets within the CAR-T-cell product correlate with their subsequent in vivo expansion and persistence. We analyzed 14 patients with B-cell malignancies infused with autologous CAR.CD19-redirected T cells expanded ex vivo using IL-2, and found that their in vivo expansion only correlated with the frequency within the infused product of a CD8(+)CD45RA(+)CCR7(+) subset, whose phenotype is closest to "T-memory stem cells." Preclinical models showed that increasing the frequency of CD8(+)CD45RA(+)CCR7(+) CAR-T cells in the infused line by culturing the cells with IL-7 and IL-15 produced greater antitumor activity of CAR-T cells mediated by increased resistance to cell death, following repetitive encounters with the antigen, while preserving their migration to secondary lymphoid organs. This trial was registered at www.clinicaltrials.gov as #NCT00586391 and #NCT00709033.

TOPIC: Lymphoma + (idiotype) vaccine

1 [145]. Bertinetti, C., K. Zirlik, et al. (2006). "Phase I trial of a novel intradermal idiotype vaccine in patients with advanced B-cell lymphoma: specific immune responses despite profound immunosuppression." Cancer Res **66**(8): 4496-4502.

The immunoglobulin receptor of B-cell lymphomas constitutes a specific tumor antigen (idiotype) and a target for active immunotherapy. Encouraging results have been reported in phase II trials after s.c. vaccination of follicular lymphoma patients during clinical remission with idiotype produced from eukaryotic cell lines and coupled to an immunogenic carrier macromolecule. We have developed a good manufacturing protocol for rapid expression of idiotype vaccines as recombinant Fab fragments in Escherichia coli. The objectives of this trial were to show safety and feasibility of intradermal immunization with this vaccine and to investigate whether immune responses were induced by this immunization route. Patients (n = 18) with advanced B-cell malignancies received repetitive intradermal vaccinations with 0.5 to 1.65 mg recombinant idiotype Fab fragment mixed with lipid-based adjuvant in combination with 150 mug granulocyte macrophage colony-stimulating factor s.c. at the same location. The patients' immune status was assessed by flow cytometry of peripheral blood lymphocytes and concomitant hepatitis B vaccination. Cellular and humoral immune responses to the vaccine were assessed by enzyme-linked immunospot and ELISA. Side effects of a total of 65 vaccinations were mild and did not affect the immunization schedule. No patient developed hepatitis B surface antibodies (anti-HBs) after two hepatitis B immunizations. Of 17 evaluable patients, five developed specific anti-vaccine antibodies, and eight developed anti-Fab T-cell responses. T-cell reactivity was independent of the cellular immune status and was idiotype specific as shown by statistical regression analysis (P = 0.0024) and epitope mapping studies. Intradermal administration of uncoupled recombinant idiotype with appropriate adjuvants may overcome profound clinical immunosuppression and induce specific immune responses.

2 [143]. Inoges, S., M. Rodriguez-Calvillo, et al. (2006). "Clinical benefit associated with idiotypic vaccination in patients with follicular lymphoma." J Natl Cancer Inst **98**(18): 1292-1301.

BACKGROUND: Follicular lymphoma is considered incurable, although cyclophosphamide, doxorubicin, vincristine, and prednisone (CHOP) chemotherapy can induce sequential remissions. A patient's second complete response is typically shorter than that patient's first complete response. Idiotype vaccines can elicit specific immune responses and molecular remissions in patients with follicular lymphoma. However, a clinical benefit has never been formally proven. METHODS: Thirty-three

consecutive follicular lymphoma patients in first relapse received six monthly cycles of CHOP-like chemotherapy. Patients who achieved a second complete response were vaccinated periodically for more than 2 years with autologous lymphoma-derived idiotype protein vaccine. Specific humoral and cellular responses were assessed, and patients were followed for disease recurrence. Statistical tests were two-sided. RESULTS: Idiotype vaccine could be produced for 25 patients who had a second complete response. In 20 patients (80%), a humoral (13/20) and/or a cellular (18/20) idiotype-specific response was detected. The median duration of the second complete response has not been reached, but it exceeds 33 months (range = 20+ to 51+ months). None of the 20 responders relapsed while undergoing active vaccination. All responders with enough follow-up for the comparison to be made experienced a second complete response that was statistically significantly (P<.0001) longer than both their first complete response (18 of 18 patients) and than the median duration of a CHOP-induced second complete response, i.e., 13 months (20 of 20 patients). The five nonresponders all had a second complete response that was shorter (median = 10 months; range = 8-13 months) than their first complete response (median = 17 months; range = 10-39 months). CONCLUSIONS: Idiotypic vaccination induced a specific immune response in the majority of patients with follicular lymphoma. Specific immune response was associated with a dramatic and highly statistically significant increase in disease-free survival. This is the first formal demonstration of clinical benefit associated with the use of a human cancer vaccine.

3 [135]. Levy, R., K. N. Ganjoo, et al. (2014). "Active idiotypic vaccination versus control immunotherapy for follicular lymphoma." J Clin Oncol **32**(17): 1797-1803.

PURPOSE: Idiotypes (Ids), the unique portions of tumor immunoglobulins, can serve as targets for passive and active immunotherapies for lymphoma. We performed a multicenter, randomized trial comparing a specific vaccine (MyVax), comprising Id chemically coupled to keyhole limpet hemocyanin (KLH) plus granulocyte macrophage colony-stimulating factor (GM-CSF) to a control immunotherapy with KLH plus GM-CSF. PATIENTS AND METHODS: Patients with previously untreated advanced-stage follicular lymphoma (FL) received eight cycles of chemotherapy with cyclophosphamide, vincristine, and prednisone. Those achieving sustained partial or complete remission (n=287 [44%]) were randomly assigned at a ratio of 2:1 to receive one injection per month for 7 months of MyVax or control immunotherapy. Anti-Id antibody responses (humoral immune responses [IRs]) were measured before each

immunization. The primary end point was progression-free survival (PFS). Secondary end points included IR and time to subsequent antilymphoma therapy. RESULTS: At a median follow-up of 58 months, no significant difference was observed in either PFS or time to next therapy between the two arms. In the MyVax group (n=195), anti-Id IRs were observed in 41% of patients, with a median PFS of 40 months, significantly exceeding the median PFS observed in patients without such Id-induced IRs and in those receiving control immunotherapy. CONCLUSION: This trial failed to demonstrate clinical benefit of specific immunotherapy. The subset of vaccinated patients mounting specific anti-Id responses had superior outcomes. Whether this reflects a therapeutic benefit or is a marker for more favorable underlying prognosis requires further study.

4 [141]. McCormick, A. A., S. Reddy, et al. (2008). "Plant-produced idiotype vaccines for the treatment of non-Hodgkin's lymphoma: safety and immunogenicity in a phase I clinical study." Proc Natl Acad Sci U S A **105**(29): 10131-10136.

Plant-made vaccines have been the subject of intense interest because they can be produced economically in large scale without the use of animal-derived components. Plant-made therapeutic vaccines against challenging chronic diseases, such as cancer, have received little research attention, and no previous human clinical trials have been conducted in this vaccine category. We document the feasibility of using a plant viral expression system to produce personalized (patient-specific) recombinant idiotype vaccines against follicular B cell lymphoma and the results of administering these vaccines to lymphoma patients in a phase I safety and immunogenicity clinical trial. The system allowed rapid production and recovery of idiotypic single-chain antibodies (scFv) derived from each patient's tumor and immunization of patients with their own individual therapeutic antigen. Both low and high doses of vaccines, administered alone or co-administered with the adjuvant GM-CSF, were well tolerated with no serious adverse events. A majority (>70%) of the patients developed cellular or humoral immune responses, and 47% of the patients developed antigen-specific responses. Because 15 of 16 vaccines were glycosylated in plants, this study also shows that variation in patterns of antigen glycosylation do not impair the immunogenicity or affect the safety of the vaccines. Collectively, these findings support the conclusion that plant-produced idiotype vaccines are feasible to produce, safe to administer, and a viable option for idiotype-specific immune therapy in follicular lymphoma patients.

5 [138]. Navarrete, M. A., K. Heining-Mikesch, et al. (2011). "Upfront immunization with autologous recombinant idiotype Fab fragment without prior cytoreduction in indolent B-cell lymphoma." Blood **117**(5): 1483-1491.

Idiotype vaccination for follicular lymphoma is primarily being developed as remission consolidation after chemotherapy. We investigated idiotype vaccination as primary intervention for treatment-naive indolent B-cell lymphoma and in a separate cohort as remission consolidation after chemotherapy to assess immunization-induced immune responses in relation to progression-free survival (German Clinical Trials Register, DRKS00000227). Twenty-one patients in each cohort received 6 intradermal injections of adjuvanted recombinant idiotype Fab fragment (Fab(Id)); 76% of patients in both groups developed anti-idiotype antibodies and/or cellular immunity as measured by enzyme-linked immunosorbent assay and interferon-gamma ELISpot. In treatment-naive patients, only cellular responses correlated with superior progression-free survival ($P < .002$) and durable objective remissions ($P = .04$). Immunization-induced T cells recognized hypermutated or complementarity-determining region 3 epitopes. After remission consolidation immunization, induction of anti-idiotype antibodies correlated with progression-free survival. Low B-cell counts after rituximab therapy predicted for failure to develop anti-idiotype antibodies. These

results are similar to published trials showing an association of humoral immunity with control of residual lymphoma. In contrast, effective immunity against untreated lymphoma appears to be dependent on idiotype-specific T cells. Sustained remissions in patients with vaccination-induced cellular immunity suggest clinical benefit and warrant a randomized comparison of this vaccine with expectant management for asymptomatic follicular lymphoma.

6 [147]. Neelapu, S. S., S. Baskar, et al. (2004). "Human autologous tumor-specific T-cell responses induced by liposomal delivery of a lymphoma antigen." Clin Cancer Res **10**(24): 8309-8317.

PURPOSE: The idiotype (Id) of the immunoglobulin on a given B-cell malignancy is a clonal marker that can serve as a tumor-specific antigen. We developed a novel vaccine formulation by incorporating Id protein with liposomal lymphokine that was more potent than a prototype, carrier-conjugated Id protein vaccine in preclinical studies. In the present study, we evaluated the safety and immunogenicity of this vaccine in follicular lymphoma patients. EXPERIMENTAL DESIGN: Ten patients with advanced-stage follicular lymphoma were treated with five doses of this second generation vaccine after chemotherapy-induced clinical remission. All patients were evaluated for cellular and humoral immune responses.

RESULTS: Autologous tumor and Id-specific type I cytokine responses were induced by vaccination in 10 and 9 patients, respectively. Antitumor immune responses were mediated by both CD4+ and CD8+ T cells, were human lymphocyte antigen class I and II associated, and persisted 18 months beyond the completion of vaccination. Specific anti-Id antibody responses were detected in four patients. After a median follow-up of 50 months, 6 of the 10 patients remain in continuous first complete remission. CONCLUSIONS: This first clinical report of a liposomal cancer vaccine demonstrates that liposomal delivery is safe, induces sustained tumor-specific CD4+ and CD8+ T-cell responses in lymphoma patients, and may serve as a model for vaccine development against other human cancers and infectious pathogens.

7 [142]. Neelapu, S. S., B. L. Gause, et al. (2007). "A novel proteoliposomal vaccine induces antitumor immunity against follicular lymphoma." Blood **109**(12): 5160-5163.

Clinical studies suggest that treatment with vaccines comprised of idiotype protein may be associated with improved clinical outcome in follicular lymphoma patients. The time-consuming process required to generate patient-specific vaccines is a major limitation, however. Here we report results of a pilot clinical trial with a novel autologous, tumor-derived proteoliposome vaccine formulation that could be rapidly produced within a single day. Vaccination was safe, induced autologous tumor-specific type 1 cytokine responses in 5 out of 10 follicular lymphoma patients, and was associated with induction of a sustained complete response in one patient. Other patients had large tumor burdens and progressed after a median duration of 8 months. These results suggest that further testing of this vaccine formulation, particularly in the setting of minimal disease, is warranted. Furthermore, the proteoliposome formulation may provide a model for vaccine development for other human cancers, for which tumor-associated antigens need not be defined.

8 [146]. Neelapu, S. S., L. W. Kwak, et al. (2005). "Vaccine-induced tumor-specific immunity despite severe B-cell depletion in mantle cell lymphoma." Nat Med **11**(9): 986-991.

The role of B cells in T-cell priming is unclear, and the effects of B-cell depletion on immune responses to cancer vaccines are unknown. Although results from some mouse models suggest that B cells may inhibit induction of T cell-dependent immunity by competing with antigen-presenting cells for antigens, skewing T helper response toward a T helper 2 profile and/or inducing T-cell tolerance, results from others

suggest that B cells are necessary for priming as well as generation of T-cell memory. We assessed immune responses to a well-characterized idiotype vaccine in individuals with severe B-cell depletion but normal T cells after CD20-specific antibody-based chemotherapy of mantle cell lymphoma in first remission. Humoral antigen- and tumor-specific responses were detectable but delayed, and they correlated with peripheral blood B-cell recovery. In contrast, vigorous CD4(+) and CD8(+) antitumor type I T-cell cytokine responses were induced in most individuals in the absence of circulating B cells. Analysis of relapsing tumors showed no mutations or change in expression of target antigen to explain escape from therapy. These results show that severe B-cell depletion does not impair T-cell priming in humans. Based on these results, it is justifiable to administer vaccines in the setting of B-cell depletion; however, vaccine boosts after B-cell recovery may be necessary for optimal humoral responses.

9 [144]. Redfern, C. H., T. H. Guthrie, et al. (2006). "Phase II trial of idiotype vaccination in previously treated patients with indolent non-Hodgkin's lymphoma resulting in durable clinical responses." J Clin Oncol **24**(19): 3107-3112.

PURPOSE: To evaluate idiotype (Id) vaccination as a single agent in previously treated patients with indolent non-Hodgkin's lymphoma. PATIENTS AND METHODS: Patients underwent biopsy for determination of their lymphoma-specific Id sequence. Recombinant Id protein was manufactured and covalently linked with keyhole limpet hemocyanin (KLH) to generate Id/KLH. Patients received Id/KLH 1 mg on day 1 subcutaneously, with granulocyte-macrophage colony-stimulating factor 250 mug on days 1 to 4, monthly for 6 months. Booster injections were administered until progression. Both clinical and immune responses were evaluated. RESULTS: Thirty-two previously treated patients received at least one injection of Id/KLH, and 31 were assessed for efficacy. Responses were observed in four patients (one complete response and three partial responses). Median time to onset of response was 5.9 months (range, 2.3 to 14.1 months). Median duration of response has not been reached but should be at least 19.4 months (range, 10.4 to 27.2+ months). Median time to progression is 13.5 months. The most common adverse events were mild to moderate injection site reactions. Six (67%) of nine patients tested demonstrated a cellular immune response, and four (20%) of 20 patients demonstrated an antibody response against their Id. CONCLUSION: This trial demonstrates that Id/KLH alone can induce tumor regression and durable objective responses. Further study of Id/KLH is recommended in other settings where efficacy may be further enhanced as in first-line therapy or after cytoreductive therapy.

10 [136]. Schuster, S. J., S. S. Neelapu, et al. (2011). "Vaccination with patient-specific tumor-derived antigen in first remission improves disease-free survival in follicular lymphoma." J Clin Oncol **29**(20): 2787-2794.

PURPOSE: Vaccination with hybridoma-derived autologous tumor immunoglobulin (Ig) idiotype (Id) conjugated to keyhole limpet hemocyanin (KLH) and administered with granulocyte-monocyte colony-stimulating factor (GM-CSF) induces follicular lymphoma (FL) -specific immune responses. To determine the clinical benefit of this vaccine, we conducted a double-blind multicenter controlled phase III trial. PATIENTS AND METHODS: Treatment-naive patients with advanced stage FL achieving complete response (CR) or CR unconfirmed (CRu) after chemotherapy were randomly assigned two to one to receive either Id vaccine (Id-KLH + GM-CSF) or control (KLH + GM-CSF). Primary efficacy end points were disease-free survival (DFS) for all randomly assigned patients and DFS for randomly assigned patients receiving at least one dose of Id vaccine or control. RESULTS: Of 234 patients enrolled, 177 (81%) achieved CR/CRu after chemotherapy and were randomly assigned. For 177 randomly assigned patients, including 60 patients not vaccinated because of relapse (n = 55) or other reasons (n = 5), median DFS between Id-vaccine and control arms was 23.0 versus 20.6 months, respectively (hazard ratio [HR], 0.81; 95% CI, 0.56 to 1.16; P = .256). For 117 patients who received Id vaccine (n = 76) or control (n = 41), median DFS after randomization was 44.2 months for Id-vaccine arm versus 30.6 months for control arm (HR, 0.62; 95% CI, 0.39 to 0.99; P = .047) at median follow-up of 56.6 months (range, 12.6 to 89.3 months). In an unplanned subgroup analysis, median DFS was significantly prolonged for patients receiving IgM-Id (52.9 v 28.7 months; P = .001) but not IgG-Id vaccine (35.1 v 32.4 months; P = .807) compared with isotype-matched control-treated patients. CONCLUSION: Vaccination with patient-specific hybridoma-derived Id vaccine after chemotherapy-induced CR/CRu may prolong DFS in patients with FL. Vaccine isotype may affect clinical outcome and explain differing results between this and other controlled Id-vaccine trials.

11 [140]. Timmerman, J. M., J. M. Vose, et al. (2009). "Tumor-specific recombinant idiotype immunisation after chemotherapy as initial treatment for follicular non-Hodgkin lymphoma." Leuk Lymphoma **50**(1): 37-46.

Tumor-specific variable regions of the clonal immunoglobulin (idiotype, Id) expressed by B cell non-Hodgkin lymphoma (NHL) can be targeted by active immunotherapy. We conducted a phase I/II trial to determine the safety and immunogenicity of a patient-specific, recombinant,

mammalian cell-derived Id protein conjugated to keyhole limpet hemocyanin (Id-KLH; MyVax personalised immunotherapy) in 22 patients with follicular NHL in first remission after chemotherapy. Subjects received five subcutaneous immunisations with MyVax plus locally administered granulocyte-macrophage colony-stimulating factor (GM-CSF). Among 21 evaluable patients, 62% mounted Id-specific immune responses. Evoked anti-Id antibodies recognised both recombinant Id and native Id, and could specifically stain autologous tumor cells. At median follow-up of more than 6 years, median progression-free survival is 38 months. Immunisation of follicular lymphoma patients with MyVax Id-KLH is safe and patients often mount tumor-specific immune responses. These results form the basis of a pivotal phase 3 trial of MyVax in follicular NHL.

TOPIC: Lymphoma + Denileukin diftitox

1 [155]. Ansell, S. M., H. Tang, et al. (2012). "Denileukin diftitox in combination with rituximab for previously untreated follicular B-cell non-Hodgkin's lymphoma." Leukemia **26**(5): 1046-1052.

Follicular lymphoma exhibits intratumoral infiltration by non-malignant T lymphocytes, including CD4+CD25+ regulatory T (T(reg)) cells. We combined denileukin diftitox with rituximab in previously untreated, advanced-stage follicular lymphoma patients anticipating that denileukin diftitox would deplete CD25+ T(reg) cells while rituximab would deplete malignant B cells. Patients received rituximab 375 mg/m(2) weekly for 4 weeks and denileukin diftitox 18 mcg/kg/day for 5 days every 3 weeks for 4 cycles; neither agent was given as maintenance therapy. Between August 2008 and March 2010, 24 patients were enrolled. One patient died before treatment was given and was not included in the analysis. Eleven of 23 patients (48%; 95% confidence interval (CI): 27-69%) responded; 2 (9%) had complete responses and 9 (39%) had partial responses. The progression-free rate at 2 years was 55% (95%CI: 37-82%). Thirteen patients (57%) experienced grade >/=3 adverse events and one patient (4%) died. In correlative studies, soluble CD25 and the number of CD25+ T cells decreased after treatment; however, there was a compensatory increase in IL-15 and IP-10. We conclude that although the addition of denileukin diftitox to rituximab decreased the number of CD25+ T cells, denileukin diftitox contributed to the toxicity of the combination without an improvement in response rate or time to progression.

2 [161]. Chin, K. M. and F. M. Foss (2006). "Biologic correlates of response and survival in patients with cutaneous T-cell lymphoma treated with denileukin diftitox." Clin Lymphoma Myeloma **7**(3): 199-204.

BACKGROUND: Denileukin diftitox, a fusion protein consisting of peptide sequences for the enzymatically active and membrane translocation domains of diphtheria toxin and human interleukin, resulted in a response rate of 30% in the phase III registration trial in patients with recurrent or persistent cutaneous T-cell lymphoma (CTCL). Little is known with regard to the biologic correlates of response or the impact of denileukin diftitox on disease progression and survival. PATIENTS AND METHODS: In our single-center series of 37 patients with early- and advanced-stage disease with CTCL treated with denileukin diftitox at a dose of 9 microg/kg or 18 microg/kg per day, we observed an overall response rate of 51%. RESULTS: In 8 patients with early-stage (< IIA) CTCL, there were 5 responses (62.5%), and the median survival has not been reached, with 70% of patients still alive at 46 months. In 29 patients with advanced-stage (>/= IIB) disease, there were 14 responses (49.3%), and the median survival was 31 months. Changes in the number of CD4+ CD25+ T-cell populations were observed in 7 of 19 responders, with no overall changes in the absolute lymphocyte counts during the course of therapy. Decrease in lactate dehydrogenase was strongly correlated with clinical response (P < 0.05). CONCLUSION: Denilekin diftitox was a well-tolerated treatment in early- and advanced-stage CTCL and was not associated with detrimental immunologic efects on lymphocyte populations.

3 [159]. Dang, N. H., L. Fayad, et al. (2007). "Phase II trial of the combination of denileukin diftitox and rituximab for relapsed/refractory B-cell non-Hodgkin lymphoma." Br J Haematol **138**(4): 502-505.

Denileukin diftitox plus rituximab was evaluated in relapsed/refractory B-cell non-Hodgkin lymphoma patients. Of the 38 evaluable patients, 30 (80%) were rituximab-refractory. The overall response rate (ORR) was 32%, with six complete responses (CR) and six partial responses (PR). The median time to progression for responders was 8 months (range: 2-36+); two patients with rituximab-refractory follicular lymphoma were in CR at 25 and
36+ months. The ORR was 55% (4 CRs, 2 PRs) in 11/14 patients with rituximab-refractory follicular lymphoma, and 100% in the three patients with rituximab-sensitive tumour. Most toxicities were low grade and transient, and myelotoxicity was uncommon.

4 [163]. Dang, N. H., F. B. Hagemeister, et al. (2004). "Phase II study of denileukin diftitox for relapsed/refractory B-Cell non-Hodgkin's lymphoma." J Clin Oncol **22**(20): 4095-4102.

PURPOSE: Denileukin diftitox is a fusion protein combining diphtheria toxin and interleukin-2 (IL-2) that targets tumor cells expressing the IL-2 receptor. Its efficacy has been shown in CD25+ cutaneous T-cell lymphoma, but not in B-cell non-Hodgkin's lymphoma (NHL). A phase II study was performed to evaluate the efficacy and tolerability of denileukin diftitox for relapsed or refractory B-cell NHL. PATIENTS AND METHODS: Patients with relapsed or refractory B-cell NHL were eligible. Tumor CD25 expression was determined by immunohistochemistry or flow cytometry. Denileukin diftitox was administered intravenously at a dose of 18 microg/kg once daily for 5 days every 3 weeks, up to eight cycles. RESULTS: Of the 45 patients assessable for response, 32 (71%) were refractory to the last chemotherapy treatment, and all were previously treated with rituximab. Three complete responses (6.7%) and eight partial responses (17.8%) were observed, for an overall response rate of 24.5%. Nine patients (20%) had stable disease. Objective response rates were similar in CD25+ (22%) and CD25- histologies (29%), as were stable disease rates (22% and 18%, respectively). For responding patients, the median time to treatment failure was 7 months, with a median follow-up in survivors of 18 months (range, 9 to 28 months), and the projected progression-free survival at 20 months was 24% (95% CI, 0% to 60%). Most toxicities were low-grade and transient. CONCLUSION: Denileukin diftitox seems to be effective in relapsed or refractory, CD25+ and CD25- B-cell NHL and is well-tolerated at the dosage evaluated. Evaluation of denileukin diftitox in combination with other agents may be warranted.

5 [160]. Dang, N. H., B. Pro, et al. (2007). "Phase II trial of denileukin diftitox for relapsed/refractory T-cell non-Hodgkin lymphoma." Br J Haematol **136**(3): 439-447.

This phase II study evaluated the safety and efficacy of denileukin diftitox, an interleukin-2-diphtheria toxin fusion protein, in relapsed/refractory T-cell non-Hodgkin lymphoma (T-NHL), excluding cutaneous T-cell lymphoma. Eligible patients received denileukin diftitox 18 microg/kg/d x 5 d every 3 weeks for up to eight cycles. Tumour staging was performed every two cycles and the primary endpoint was the objective response rate [complete response (CR) + partial response (PR)]. For 27 patients enrolled, median age: 55 years (range 26-80 years), 70.4% male, and mean prior therapies: 2.5 (range 1-6). Objective responses (six CRs, seven PRs) were achieved in 13 patients (48.1%), stable disease in eight (29.6%) and six (22.2%) had progressive disease. An objective response was achieved in

eight of 13 patients (61.5%) with CD25(+) tumours (four CR/four PR) and five of 11 patients (45.5%) with CD25(-) tumours (two CR/three PR). Median progression-free survival was 6 months (range, 1-38+ months). Most adverse reactions were grade 1/2 and transient. No grade 4-5 toxicities were reported. Denileukin diftitox had significant activity and was well tolerated in relapsed/refractory T-NHL, with responses observed in both CD25(+) and CD25(-) tumours. Further studies of denileukin diftitox in combination with
other agents are warranted in previously untreated and relapsed/refractory T-NHL.

6 [148]. Duvic, M., L. Geskin, et al. (2013). "Duration of response in cutaneous T-cell lymphoma patients treated with denileukin diftitox: results from 3 phase III studies." Clin Lymphoma Myeloma Leuk **13**(4): 377-384.

BACKGROUND: We aimed to determine duration of response in patients with CTCL treated using DD who experienced partial response, complete clinical response, or complete response. PATIENTS AND METHODS: Data from 3 phase III studies were pooled. Patients received up to 8 courses of 9 or 18 mug/kg intravenous DD daily for 5 days every 21 days, or placebo. Data on DD-treated patients were analyzed by dose and CD25 status. Kaplan-Meier product limit estimates and 95% confidence intervals were calculated for duration of response and time to response. RESULTS: The pooled population comprised 263 DD-treated and 44 placebo-treated patients, and 100 and 7, respectively, had at least a partial response. Median duration of response using DD was 277 days vs. 81 days using placebo. Overall response vs. placebo (n = 7; 16%) as (n = 25; 31%) for DD 9 mug/kg (P = .05), (n = 56; 47%) for DD 18 mug/kg (P = .004), (n = 8; 28%) for re-treated patients (DD 18 mug/kg; P = .21), and (n = 11; 31%) for CD25 low-expression patients (DD 18 mug/kg; P = .14). Overall response rates were similar between patients who did (n = 95; 36%) and did not (n = 105; 40%) develop capillary leak syndrome (CLS); median duration of response was longer in patients who developed CLS, but was not significant (619 vs. 267 days, respectively; P = .28). Adverse events occurred in 98% of DD-treated patients; most frequent were nausea, pyrexia, fatigue, CLS, and rigors. CONCLUSION: These data indicate a durable response with DD in CTCL, even in heavily pretreated patients and those with CD25 low-expression disease.

7 [151]. Duvic, M., A. G. Martin, et al. (2013). "Efficacy and safety of denileukin diftitox retreatment in patients with relapsed cutaneous T-cell lymphoma." Leuk Lymphoma **54**(3): 514-519.

This open-label phase III trial, a companion to an earlier placebo-controlled trial, evaluated safety and efficacy of denileukin diftitox (DD) in patients with cutaneous T-cell lymphoma (CTCL) who relapsed after responding to DD primary treatment in the earlier trial. Twenty relapsed patients (stages IA-III) received DD 18 mug/kg/day intravenously on days 1-5 of a 21-day cycle, for </= 8 cycles. Efficacy was assessed monthly during the first year then every 3 months. The overall response rate was 40%, mostly partial responses. Nine patients (all baseline stages </= IIA) experienced progression. Intent-to-treat median progression-free survival was 205 days, and median duration of response was 274 days. The most common adverse events were nausea, upper respiratory tract infections, fatigue and rigors. Three patients withdrew because of toxicity. This study showed that DD may provide clinically meaningful benefit in patients with CTCL who relapsed after initial response to DD.

8 [162]. Foss, F., M. F. Demierre, et al. (2005). "A phase-1 trial of bexarotene and denileukin diftitox in patients with relapsed or refractory cutaneous T-cell lymphoma." Blood **106**(2): 454-457.

Denileukin diftitox, a genetically engineered fusion protein combining the enzymatically active domains of diphtheria toxin and the full-length sequence for interleukin-2 (IL-2), efficiently targets lymphoma cells expressing the high-affinity IL-2 receptor (IL-2R) consisting of the alpha/p55/CD25, beta/p75/CD122, and gamma/p64/CD132 chains. In vitro studies demonstrated that the retinoid X receptor (RXR) retinoid, bexarotene, at biologically relevant concentrations of 10(-6) M to 10(-8) M, upregulated both the p55 and p75 subunits of the IL-2R and enhanced 5- to 10-fold the susceptibility of T-cell leukemia cells to denileukin diftitox. To determine whether this biomodulatory effect could be recapitulated in vivo, we treated 14 patients with relapsed or refractory cutaneous T-cell lymphoma with escalating doses of bexarotene (75 mg/day-300 mg/day) and denileukin diftitox (18 mcg/kg per day x 3 days every 21 days) in a phase 1 trial. Overall response was 67% (4 complete responses, 4 partial responses). Modulation of IL-2R expression was observed at or above a bexarotene dose of 150 mg/day. Four patients experienced grade 2 or 3 leukopenia, and 2 had grade 4 lymphopenia. Our results demonstrate that the combination of denileukin diftitox and bexarotene is well tolerated and that even low doses (150 mg/day) of bexarotene are capable of in vivo upregulation of CD25 expression on circulating leukemia cells.

9 [149]. Foss, F. M., N. Sjak-Shie, et al. (2013). "A multicenter phase II trial to determine the safety and efficacy of combination therapy with denileukin diftitox and cyclophosphamide, doxorubicin, vincristine and prednisone in

untreated peripheral T-cell lymphoma: the CONCEPT study." Leuk Lymphoma **54**(7): 1373-1379.

This phase II study to determine the safety and efficacy of denileukin diftitox (DD) and cyclophosphamide, doxorubicin, vincristine and prednisone (CHOP) enrolled patients with newly diagnosed peripheral T-cell lymphoma (PTCL). Forty-nine received DD 18 mug/kg/day (days 1, 2) with CHOP (day 3) every 21 days for </= 6-8 cycles. Intent-to-treat (ITT) and safety populations comprised all patients. In the ITT population, the overall response rate was 65%, median duration of response was 30 months and median progression-free survival was 12 months. Median overall survival was not attained at the end of the study, and the overall survival rate was 63.3%. The two most frequent treatment-related adverse events (AEs) were fatigue and nausea. Most frequent AEs >/= grade 3 within the hematologic system were lymphopenia (24.5%), neutropenia (20.4%) and leukopenia (18.4%). Three treatment-related deaths occurred. DD plus CHOP was well tolerated, and progression-free and overall survival improved versus historical comparison with CHOP alone. Confirmation in larger trials is warranted.

10 [156]. Kadin, M. E. and E. C. Vonderheid (2010). "Targeted therapies: Denileukin diftitox--a step towards a 'magic bullet' for CTCL." Nat Rev Clin Oncol **7**(8): 430-432.

11 [158]. Kuzel, T. M., S. Li, et al. (2007). "Phase II study of denileukin diftitox for previously treated indolent non-Hodgkin lymphoma: final results of E1497." Leuk Lymphoma **48**(12): 2397-2402.

Denileukin diftitox (DD) is approved for treatment of CD-25 expressing cutaneous T-cell lymphomas (CTCL). Initial studies of DD demonstrated responses in patients with B-cell non-Hodgkin lymphoma (NHL). This phase II trial evaluated response rate (RR) and tolerability of DD in this population. Patients were stratified into two arms: those with NHL expressing > or =20% IL-2R (IL-2R+) or <20% IL-2R (IL-2R-). DD was dosed at 18 microg/kg/day for 5 days every 21 days. Corticosteroid pre-medication was not allowed. Thirty-five patients of a planned 77 accrued due to closure for slow accrual. This report is on 29 patients (18 males) with indolent B-cell NHL (11
IL-2R+ and 18 IL-2R-). Histologic subtypes included small lymphocytic (SLL) (8 patients) and follicular grade I/II lymphoma (21 patients). Patients received a median of three prior regimens, including rituximab in 76%. Three partial responses were observed (RR 10%). The RR for the IL-2R- and IL-2R+ patients was 11% and 9%, respectively. Of 8 patients with SLL, 2 responded. Toxicities were generally grade I - II and transient but 1 patient

experienced a fatal thrombo-embolism. Therapy with DD is tolerable and modest efficacy was observed in SLL subtype. Measured IL-2R status did not correlate with efficacy.

12 [157]. Prince, H. M., M. Duvic, et al. (2010). "Phase III placebo-controlled trial of denileukin diftitox for patients with cutaneous T-cell lymphoma." J Clin Oncol **28**(11): 1870-1877.

PURPOSE This phase III, placebo-controlled, randomized trial was designed to investigate efficacy and safety of two doses of denileukin diftitox (DD; DAB(389)-interleukin-2 [IL-2]), a recombinant fusion protein targeting IL-2 receptor-expressing malignant T lymphocytes, in patients with stage IA to III, CD25 assay-positive cutaneous T-cell lymphoma (CTCL), including the mycosis fungoides and Sezary syndrome forms of the disease, who had received up to three prior therapies. The primary end point was overall response rate (ORR). PATIENTS AND METHODS Patients (N = 144) with biopsy-confirmed, CD25 assay-positive CTCL were randomly assigned to DD 9 microg/kg/d (n = 45), DD 18 microg/kg/d (n = 55), or placebo infusions (n = 44), administered for 5 consecutive days every 3 weeks for up to eight cycles. Patients were monitored for drug efficacy, clinical benefit, and safety of DD. RESULTS ORR was 44% for all participants treated with DD (n = 100; 10% complete response [CR] and 34% partial response [PR]) compared with 15.9% for placebo-treated patients (2% CR and 13.6% PR). ORR was higher in the 18 microg/kg/d group versus the 9 microg/kg/d group (49.1% v 37.8%, respectively), and both doses were significantly superior to placebo. Progression-free survival (PFS) was significantly longer (median, > 2 years) for both DD doses compared with placebo (median,
124 days; P < .001). Rates of moderately severe and severe adverse events (AEs) were slightly higher in the DD groups, whereas moderate and mild AEs were similar to placebo. No statistical differences were observed for drug-related serious AEs. CONCLUSION DD had a significant and durable effect on ORR and PFS with an acceptable safety profile in patients with early- and late-stage CTCL.

13 [152]. Prince, H. M., A. G. Martin, et al. (2013). "Denileukin diftitox for the treatment of CD25 low-expression mycosis fungoides and Sezary syndrome." Leuk Lymphoma **54**(1): 69-75.

In a placebo-controlled study, denileukin diftitox (DD) was effective against cutaneous T-cell lymphoma (CTCL) expressing CD25. An open-label companion study examined the efficacy and safety of DD in 36 patients with skin biopsies containing < 20% CD25 cells by immunohistochemistry staining (CD25 low expression). Patients received

DD 18 mug/kg/day for 5 consecutive days every 3 weeks for up to eight courses. The primary endpoint, overall response rate, was 30.6% (95% confidence interval: 16.3, 48.1), 33.3% for stage IIA or lower disease, and 26.7% for stage IIB or greater disease. Median progression-free survival (PFS) was > 487 days, and median time to treatment failure was 68.5 days. No difference in PFS by disease stage was observed. The safety profile of DD in CD25 low-expression disease was similar to that in CD25+ disease. These findings suggest that CD25 low expression does not preclude a meaningful clinical response to DD in patients with CTCL.

TOPIC: Lymphoma + Interferon Alfa-2b

1 [170]. Aviles, A., N. Neri, et al. (2004). "Interferon alpha 2b as maintenance therapy improves outcome in follicular lymphoma." Leuk Lymphoma **45**(11): 2247-2251.

The role of interferon alpha as maintenance therapy in follicular lymphoma (FL) remains unsolved. We started a controlled clinical trial to assess if interferon alpha 2b could improve outcome, measured with event free survival (EFS) and overall survival (OS) in patients with FL in complete remission after chemotherapy based anthracyclines and adjuvant radiotherapy to sites of initial bulky disease. Three hundred and eighty four patients in complete response after 6 cycles of CEOP-Bleo (cyclophosphamide, epirubicin, vincristine, prednisone and bleomycin, at standard doses), and adjuvant radiotherapy when necessary, were randomized to received Interferon alpha 2b, three times a week for 1 year or no treatment (control group). Median follow up was 9.8 years (range 7.0-15 years); actuarial curves showed that EFS was 64% (95% confidence interval (CI) 56-71%) in patients treated with interferon that was statistically significant to patients in the control group: 35% (95% CI: 28-43%) (p<.01). OS was also statistically significant: 81% in patients treated with interferon (95% CI: 74-93%) and 57% (95% CI: 50-63%) in the control group (p<.001). Toxicity was mild, all patients received the planned dose of interferon on time. The use of aggressive chemotherapy and maintenance therapy with interferon alpha 2b in follicular lymphoma improved outcome; more than 60% of patients remain alive free of disease at longer follow-up.

2 [171]. Aviles, A., N. Neri, et al. (2004). "Maintenance therapy with interferon-alpha 2b, cyclophosphamide, and prednisone in aggressive diffuse large cell lymphoma." Stem Cells Dev **13**(2): 205-209.

Maintenance therapy in patients with aggressive malignant lymphoma using biological modifiers remains uncertain. We conducted a controlled

clinical trial to evaluate the efficacy and toxicity of interferon-alpha 2b, cyclophosphamide, and prednisone as maintenance therapy in patients with aggressive diffuse large B cell lymphomas in complete remission after aggressive chemotherapy. In an intent-to-treat analysis, 169 patients were eligible for this study; the end points were event-free survival (EFS) and overall survival (OS). With a median follow-up of 49.3 months, no statistical differences were observed and actuarial curves at 5 years showed that EFS was 71% (95% confidence interval [CI], 63-79%) for patients who received maintenance compared to 63% (95% CI, 59-71%) for patients in control group (p = 0.05). No statistical differences were observed in OS between maintenance arm: 84% (95% CI, 78-89%) and control group 83% (95% CI, 77-88%) in control group (p = 0.2). All patients received the maintenance therapy as planned and in time, thus dose intensity was considered 1.0 in all cases. Acute toxicity was mild, and no delay or suspension of treatment was necessary. Late toxicity was not evident until now. We conclude that use of maintenance therapy combining interferon-alpha 2b, cyclophosphamide, and prednisone is not useful in patients with aggressive lymphoma if they had been treated with aggressive combined chemotherapy.

3 [164]. Bosly, A., A. Grigg, et al. (2013). "A randomized study of interferon alpha-2b versus no treatment as consolidation after high dose therapy and autologous stem cell transplantation for patients with relapsed lymphoma." Oncologist **18**(11): 1189.

Patients with lymphoma who have experienced a first relapse or progression and have disease deemed sensitive to salvage chemotherapy nevertheless have a high likelihood of having a second relapse. To decrease the likelihood of a second relapse after high-dose therapy (HDT) and autologous stem cell transplantation (ASCT), interferon (IFN) alpha-2b was given in a prospective randomized international trial. Methods. In this trial, 221 patients with varying histologic diagnoses (8 small lymphocytic, 37 follicular, 9 mantle, 90 diffuse large B-cell, 20 peripheral T-cell, 3 high-grade B-cell non-Hodgkin lymphoma, and 54 Hodgkin lymphoma) were randomly assigned to receive no further treatment (arm A: 117 patients) or IFNalpha-2b, 3 MU three times weekly, for 18 months (arm B: 104 patients). Results. In arm B, 21 patients (20%) did not receive IFNalpha-2b because of early progression or absence of hematologic recovery, 29 patients (28%) completed the 18 months of treatment, and 54 patients (52%) interrupted treatment because of progression (23%) or toxicity (29%). Event-free survival and overall survival were not different between the two arms on an intent-to-treat analysis and also if analysis was restricted to patients who were alive and had not experienced

disease progression three months after transplantation. The study was not sufficiently powered to evaluate effects in histologic subtypes. Conclusion. In this trial, post-autograft IFNalpha-2b did not improve outcomes in a heterogeneous group of patients with lymphoma.

4 [165]. Smith, S. M., J. Johnson, et al. (2009). "Recombinant interferon-alpha2b added to oral cyclophosphamide either as induction or maintenance in treatment-naive follicular lymphoma: final analysis of CALGB 8691." Leuk Lymphoma **50**(10): 1606-1617.

Recombinant interferon alpha-2b (IFN-alpha2) has direct and indirect antiproliferative effects in lymphoma, and may augment cytotoxicity when combined with chemotherapy. CALGB 8691 is a randomized study of daily oral cyclophosphamide (CPA) at 100 mg/m2 with or without IFN-alpha2 at 2 x 106 IU/m2 three times per week, followed by a second randomization between IFN-alpha2 maintenance (2 x 106 IU/m2 three times weekly) versus observation in treatment-naive patients with follicular lymphoma (FL). Five hundred eighty-one patients were randomized to either CPA (n = 293) or CPA plus IFN-alpha2 (n = 288). One hundred five responding patients were randomized to observation and 99 to maintenance IFN-alpha2. With a median follow-up of 11.5 years, the median event-free and overall survival (OS) for CPA induction alone were 2.5 years (95% CI 2.2, 3.0) and 9 years (95% CI 7.7, 10.2), compared to 2.4 years (95% CI 2.1, 3.1) and 8.4 years (95% CI 7.5, 11.1) for the combination arm (p = NS). Patients with a partial response (PR) and randomized to observation had the worst outcome (event-free survival (EFS) 1.8 years versus 3.9 years; p = 0.002). Patients with a PR randomized to IFN-alpha2 had a similar EFS to compared to patients with complete response (CR), but this did not translate into a survival advantage. Myelosuppression was increased in IFN-alpha2-containing arms. Despite the small benefit in EFS in patients with PR randomized to IFN-alpha2 maintenance, we conclude that the addition of low dose IFN-alpha2 did not significantly improve the response rate, duration of response, event-free, or OS obtained with single-agent daily oral CPA in patients with previously untreated FL.

5 [167]. Straus, D. J., M. Duvic, et al. (2007). "Results of a phase II trial of oral bexarotene (Targretin) combined with interferon alfa-2b (Intron-A) for patients with cutaneous T-cell lymphoma." Cancer **109**(9): 1799-1803.

BACKGROUND: Bexarotene is one of the most active single agents for the treatment of recurring or refractory cutaneous T-cell lymphoma (CTCL). Interferon alfa has also been used for many years as an effective treatment for this disease. The results in recent case reports of the combination of bexarotene and interferon alfa have been promising.

Based on more extensive results reported with the combination of other retinoids with interferon alfa, the present study attempted to determine the response rate, response duration, and safety of bexarotene (Targretin capsules, Ligand Pharmaceuticals, San Diego, Calif) alone and then with the addition of interferon alfa-2b (Intron-A, Schering-Plough, Kenilworth, NJ). METHODS: Patients with biopsy-proven CTCL, TNM stages IB, IIA, IIB-IV, were treated with oral bexarotene 300 mg/m2/day for at least 8 weeks. If a complete response was not seen after 8 weeks, interferon alfa-2b 3 million units (MU) subcutaneously was added, and increased to 5 MU if tolerated, 3 times a week. RESULTS: A total of 22 patients were enrolled at 5 sites, and 18 patients were assessable for response. Overall response rate for combined bexarotene and interferon alfa was 39% (95% confidence interval [CI]: 17%-64%), including 1 patient with a clinical complete response, 6 patients with partial response, 3 patients with stable disease, and 8 patients with progressive disease. Three partial responses were first noted during the bexarotene-alone phase. Adverse events were generally manageable, and only 1 patient was withdrawn from study for hypertriglyceridemia. CONCLUSIONS: The addition of interferon alfa-2b did not increase the response rate that would have been expected with bexarotene alone.

TOPIC: (Mantle cell) Lymphoma + Lenalidomide

No citations found.

TOPIC: (Mantle cell) Lymphoma + Bortezomib

No citations found.

TOPIC: Lymphoma + checkpoint inhibitor(s) or nivolumab or ipilimumab

1 [229]. Ansell, S. M., S. A. Hurvitz, et al. (2009). "Phase I study of ipilimumab, an anti-CTLA-4 monoclonal antibody, in patients with relapsed and refractory B-cell non-Hodgkin lymphoma." Clin Cancer Res **15**(20): 6446-6453.

PURPOSE: The growth of non-Hodgkin lymphomas can be influenced by tumor-immune system interactions. Cytotoxic T-lymphocyte antigen 4 (CTLA-4) is a negative regulator of T-cell activation that serves to dampen antitumor immune responses. Blocking anti-CTLA-4 monoclonal antibodies improves host resistance to immunogenic tumors, and the anti-CTLA-4 antibody ipilimumab (MDX-010) has clinical activity against melanoma,

prostate, and ovarian cancers. EXPERIMENTAL DESIGN: We did a phase I trial of ipilimumab in patients with relapsed/refractory B-cell lymphoma to evaluate safety, immunologic activity, and potential clinical efficacy. Treatment consisted of ipilimumab at 3 mg/kg and then monthly at 1 mg/kg x 3 months (dose level 1), with subsequent escalation to 3 mg/kg monthly x 4 months (dose level 2). RESULTS: Eighteen patients were treated, 12 at the lower dose level and 6 at the higher dose level. Ipilimumab was generally well tolerated, with common adverse events attributed to it, including diarrhea, headache, abdominal pain, anorexia, fatigue, neutropenia, and thrombocytopenia. Two patients had clinical responses; one patient with diffuse large B-cell lymphoma had an ongoing complete response (>31 months), and one with follicular lymphoma had a partial response lasting 19 months. In 5 of 16 cases tested (31%), T-cell proliferation to recall antigens was significantly increased (>2-fold) after ipilimumab therapy. CONCLUSIONS: Blockade of CTLA-4 signaling with the use of
ipilimumab is well tolerated at the doses used and has antitumor activity in patients with B-cell lymphoma. Further evaluation of ipilimumab alone or in combination with other agents in B-cell lymphoma patients is therefore warranted.

2 [236]. Bashey, A., B. Medina, et al. (2009). "CTLA4 blockade with ipilimumab to treat relapse of malignancy after allogeneic hematopoietic cell transplantation." Blood **113**(7): 1581-1588.

Relapse of malignancy after allogeneic hematopoietic cell transplantation (allo-HCT) remains a therapeutic challenge. Blockade of the CTLA4 molecule can effectively augment antitumor immunity mediated by autologous effector T cells. We have assessed the safety and preliminary efficacy of a neutralizing, human anti-CTLA4 monoclonal antibody, ipilimumab, in stimulating the graft-versus-malignancy (GVM) effect after allo-HCT. Twenty-nine patients with malignancies that were recurrent or progressive after allo-HCT, received ipilimumab as a single infusion at dose cohorts between 0.1 and 3.0 mg/kg. Dose-limiting toxicity was not encountered, and ipilimumab did not induce graft-versus-host disease (GVHD) or graft rejection. Organ-specific immune adverse events (IAE) were seen in 4 patients (grade 3 arthritis, grade 2 hyperthyroidism, recurrent grade 4 pneumonitis). Three patients with lymphoid malignancy developed objective disease responses following ipilimumab: complete remission (CR) in 2 patients with Hodgkin disease and partial remission (PR) in a patient with refractory mantle cell lymphoma. At the 3.0 mg/kg dose, active serum concentrations of ipilimumab were maintained for more than 30 days after a single infusion. Ipilimumab, as administered in this

clinical trial, does not induce or exacerbate clinical GVHD, but may cause organ-specific IAE and regression of malignancy. This study is registered at (http://clinicaltrials.gov) under NCI protocol ID P6082.

3 [222]. Zhou, J., A. Bashey, et al. (2011). "CTLA-4 blockade following relapse of malignancy after allogeneic stem cell transplantation is associated with T cell activation but not with increased levels of T regulatory cells." Biol Blood Marrow Transplant **17**(5): 682-692.

Cytotoxic T lymphocyte-associated antigen 4 (CTLA-4) is a key negative regulator of T cell activation and proliferation. Ipilimumab is a human monoclonal antibody that specifically blocks the binding of CTLA-4 to its ligand. To test the hypothesis that blockade of CTLA-4 by ipilimumab could augment graft-versus-malignancy (GVM) effects without a significant impact on graft-versus-host disease (GVHD), we conducted a phase I clinical trial of ipilimumab infusion in patients with relapsed malignancy following allogeneic hematopoietic stem cell transplantation (allo-HSCT). Here, we report the analysis of peripheral blood T lymphocyte reconstitution, T regulatory cell (Treg) expression, and T cell activation markers after a single dose of ipilimumab in 29 patients. Peripheral blood samples were collected from all patients before and after ipilimumab infusion. Lymphocyte immunophenotyes, including levels of CD4(+)CD25(high) cells and T cell activation markers, were analyzed in all cases. Levels of CD4(+)CD25(high)Foxp3(+) cells and intracellular CTLA-4 in CD4(+) T cells also were evaluated in the last 11 cases. We found lower baseline levels of CD4(+) and CD45RO(+) T cells in patients compared with normal controls. More than 50% of the patients had abnormally low lymphocyte counts (CD4 or/and CD8 T cells), and some had no circulating
B lymphocytes. The percentages of both CD4(+)CD25(high) and CD4(+)CD25(high)Foxp3(+) T cells were significantly higher in patients before ipilimumab infusion than in healthy donors. Twenty of 29 patients exhibited an elevated level of CD4(+)CD25(low) activated T cells at baseline, compared with only 3 of 26 healthy donors. Both CD4(+) and CD8(+) T lymphocyte counts were significantly increased after ipilimumab infusion. There was no consistent change in absolute lymphocyte count or in the number of T cells expressing the activation marker CD69. However, increases in CD4(+)CD25(low) T cells were seen in 20 of 29 patients and increases in CD4(+)HLA-DR(+) T cells were seen in the last 10 patients in the first 60 days after ipilimumab infusion. Although the percentages of both CD4(+)CD25(high) and CD4(+)CD25(high)Foxp3(+) T cells decreased significantly during the observation period, the absolute cell counts did not change. Intracellular CTLA-4 expression in CD4(+)CD25(lo/-) T cells

increased significantly after ipilimumab infusion. We conclude that CTLA-4 blockade by a single infusion of ipilimumab increased CD4(+) and CD4(+)HLA-DR(+) T lymphocyte counts and intracellular CTLA-4 expression at the highest dose level. There was no significant change in Treg cell numbers after ipilimumab infusion. These data demonstrate that significant changes in T cell populations occur on exposure to a single dose of ipilimumab. Further studies with multiple doses are needed to explore this phenomenon further and to correlate changes in lymphocyte subpopulations with clinical events.

TOPIC: Lymphoma + adoptive T Cell therapy or adoptive T cell transfer

1 [329]. Bollard, C. M., S. Gottschalk, et al. (2007). "Complete responses of relapsed lymphoma following genetic modification of tumor-antigen presenting cells and T-lymphocyte transfer." Blood **110**(8): 2838-2845.

Epstein-Barr virus (EBV)-associated tumors developing in immunocompetent individuals present a challenge to immunotherapy, since they lack expression of immunodominant viral antigens. However, the tumors consistently express viral proteins including LMP2, which are immunologically "weak" but may nonetheless be targets for immune T cells. We previously showed that a majority of cytotoxic T lymphocytes (CTLs) reactivated using EBV-transformed B-lymphoblastoid cells lines (LCLs) contained minor populations of LMP2-specific T cells and homed to tumor sites. However, they did not produce remissions in patients with bulky disease. We have now used gene transfer into antigen-presenting cells (APCs) to augment the expression and immunogenicity of LMP2. These modified APCs increased the frequency of LMP2-specific CTLs by up to 100-fold compared with unmodified LCL-APCs. The LMP2-specific population expanded and persisted in vivo without adverse effects. Nine of 10 patients treated in remission of high-risk disease remain in remission, and 5 of 6 patients with active relapsed disease had a tumor response, which was complete in 4 and sustained for more than 9 months. It is therefore possible to generate immune responses to weak tumor antigens by ex vivo genetic modification of APCs and the CTLs so produced can have substantial antitumor activity. This study is registered at http://www.cancer.gov/clinicaltrials (protocol IDs: BCM-H-9936, NCT00062868, NCT00070226).

2 [254]. Bollard, C. M., S. Gottschalk, et al. (2014). "Sustained complete responses in patients with lymphoma receiving autologous cytotoxic T

lymphocytes targeting Epstein-Barr virus latent membrane proteins." J Clin Oncol **32**(8): 798-808.

PURPOSE: Tumor cells from approximately 40% of patients with Hodgkin or non-Hodgkin lymphoma express the type II latency Epstein-Barr virus (EBV) antigens latent membrane protein 1 (LMP1) and LMP2, which represent attractive targets for immunotherapy. Because T cells specific for these antigens are present with low frequency and may be rendered anergic by the tumors that express them, we expanded LMP-cytotoxic T lymphocytes (CTLs) from patients with lymphoma using autologous dendritic cells and EBV-transformed B-lymphoblastoid cell lines transduced with an adenoviral vector expressing either LMP2 alone (n = 17) or both LMP2 and DeltaLMP1
(n = 33). PATIENTS AND METHODS: These genetically modified antigen-presenting cells expanded CTLs that were enriched for specificity against type II latency LMP antigens. When infused into 50 patients with EBV-associated lymphoma, the expanded CTLs did not produce infusional toxicities. RESULTS: Twenty-eight of 29 high-risk or multiple-relapse patients receiving LMP-CTLs as adjuvant therapy remained in remission at a median of 3.1 years after CTL infusion. None subsequently died as a result of lymphoma, but nine succumbed to complications associated with extensive prior chemoradiotherapy, including myocardial infarction and secondary malignancies. Of 21 patients with relapsed or resistant disease at the time of CTL infusion, 13 had clinical responses, including 11 complete responses. T cells specific for LMP as well as nonviral tumor-associated antigens (epitope spreading) could be detected in the peripheral blood within 2 months after CTL infusion, but this evidence for epitope spreading was seen only in patients achieving clinical responses. CONCLUSION: Autologous T cells directed to the LMP2 or LMP1 and LMP2 antigens can induce durable complete responses without significant toxicity. Their earlier use in the disease course may reduce delayed treatment-related mortality.

3 [320]. Di Nicola, M., R. Zappasodi, et al. (2009). "Vaccination with autologous tumor-loaded dendritic cells induces clinical and immunologic responses in indolent B-cell lymphoma patients with relapsed and measurable disease: a pilot study." Blood **113**(1): 18-27.

Eighteen relapsed patients with measurable indolent non-Hodgkin lymphoma (NHL) were vaccinated with dendritic cells (DCs) loaded with killed autologous tumor cells. Six patients had objective clinical responses including 3 continuous complete responses (CRs) and 3 partial responses (PRs), with a median follow up of 50.5 months. Eight patients had stable disease, whereas 4 had progressive disease. Clinical responses were

significantly associated with a reduction in CD4(+)CD25(+)FOXP3(+) regulatory T cells, an increase in CD3(-)CD56(dim)CD16(+) natural killer (NK) cells, and maturation of lymphocytes to the effector memory stage in either postvaccination peripheral blood or tumor specimen samples. In partial responding patients, vaccination significantly boosted the IFN-gamma-producing T-cell response to autologous tumor challenge. In one HLA-A*0201(+) patient who achieved CR, IL-4 release by circulating T cells in response to tumor-specific IgH-encoded peptides was also documented. Immunohistochemical analysis of tumor biopsies using biotin-conjugated autologous serum samples revealed a tumor-restricted humoral response only in the postvaccination serum from responding patients. Collectively these results demonstrate that vaccination with tumor-loaded DCs may induce both T- and B-cell responses and produces clinical benefits in indolent NHL patients with measurable disease. This study is registered with the Istituto Superiore di Sanita: http://www.iss.it with protocol number 7578-PRE 21-801.

4 [281]. Di Stasi, A., S. K. Tey, et al. (2011). "Inducible apoptosis as a safety switch for adoptive cell therapy." N Engl J Med **365**(18): 1673-1683.

BACKGROUND: Cellular therapies could play a role in cancer treatment and regenerative medicine if it were possible to quickly eliminate the infused cells in case of adverse events. We devised an inducible T-cell safety switch that is based on the fusion of human caspase 9 to a modified human FK-binding protein, allowing conditional dimerization. When exposed to a synthetic dimerizing drug, the inducible caspase 9 (iCasp9) becomes activated and leads to the rapid death of cells expressing this construct. METHODS: We tested the activity of our safety switch by

introducing the gene into donor T cells given to enhance immune reconstitution in recipients of haploidentical stem-cell transplants. Patients received AP1903, an otherwise bioinert small-molecule dimerizing drug, if graft-versus-host disease (GVHD) developed. We measured the effects of AP1903 on GVHD and on the function and persistence of the cells containing the iCasp9 safety switch. RESULTS: Five patients between the ages of 3 and 17 years who had undergone stem-cell transplantation for relapsed acute leukemia were treated with the genetically modified T cells. The cells were detected in peripheral blood from all five patients and increased in number over time, despite their constitutive transgene expression. A single dose of dimerizing drug, given to four patients in whom GVHD developed, eliminated more than 90% of the modified T cells within 30 minutes after administration and ended the GVHD without recurrence. CONCLUSIONS: The iCasp9 cell-suicide system may increase

the safety of cellular therapies and expand their clinical applications. (Funded by the National Heart, Lung, and Blood Institute and the National Cancer Institute; ClinicalTrials.gov number, NCT00710892.).

5 [250]. Gallot, G., S. Vollant, et al. (2014). "T-cell therapy using a bank of EBV-specific cytotoxic T cells: lessons from a phase I/II feasibility and safety study." J Immunother **37**(3): 170-179.

We report herein the results we obtained and the limitations we experienced during the production and use of a bank of Epstein-Barr virus (EBV)-transformed human cytotoxic T lymphocytes (EBV-CTLs). To assess the feasibility and toxicity of this strategy, we selected and stored, in liquid nitrogen, 4 billion EBV-CTLs from each of the 13 selected donors. Subsequently, in a multicenter phase I/II study, 11 patients with EBV-associated lymphoma resistant to conventional treatments received 1-3 doses of 5 million EBV-CTLs/kg with 1-3 and 0-4 compatibilities for human leukocyte antigen (HLA)-I and HLA-II, respectively. Except for one event of fever after injection, no immediate or delayed toxicity, no graft versus host disease, and no graft rejection attributable to CTL infusion were observed. Three patients presented complete remission and 1 partial remission after treatment. Considering the clinical options currently available, and the constrains associated with CTL preparation and implementation, we conclude that CTL banks should consist of a reasonably small number of cell lines with documented specificities. This objective could be more easily achieved if the few homozygous donors for the most frequent HLA alleles of the targeted population could be made available for such a project.

6 [312]. Heslop, H. E., K. S. Slobod, et al. (2010). "Long-term outcome of EBV-specific T-cell infusions to prevent or treat EBV-related lymphoproliferative disease in transplant recipients." Blood **115**(5): 925-935.

T-cell immunotherapy that takes advantage of Epstein-Barr virus (EBV)-stimulated immunity has the potential to fill an important niche in targeted therapy for EBV-related cancers. To address questions of long-term efficacy, safety, and practicality, we studied 114 patients who had received infusions of EBV-specific cytotoxic T lymphocytes (CTLs) at 3 different centers to prevent or treat EBV(+) lymphoproliferative disease (LPD) arising after hematopoietic stem cell transplantation. Toxicity was minimal, consisting mainly of localized swelling at sites of responsive disease. None of the 101 patients who received CTL prophylaxis developed EBV(+) LPD, whereas 11 of 13 patients treated with CTLs for biopsy-proven or probable LPD achieved sustained complete remissions. The gene-marking component of this study enabled us to demonstrate

the persistence of functional CTLs for up to 9 years. A preliminary analysis indicated that a patient-specific CTL line can be manufactured, tested, and infused for $6095, a cost that compares favorably with other modalities used in the treatment of LPD. We conclude that the CTL lines described here provide safe and effective prophylaxis or treatment for lymphoproliferative disease in transplantation recipients, and the manufacturing methodology is robust and can be transferred readily from one institution to another without loss of reproducibility.

7 [313]. Khammari, A., N. Labarriere, et al. (2009). "Treatment of metastatic melanoma with autologous Melan-A/MART-1-specific cytotoxic T lymphocyte clones." J Invest Dermatol **129**(12): 2835-2842.

Immunotherapy by adoptive T-cell transfer aims at maximizing tumor antigen-specific T-cell responses. We treated 14 patients at the metastatic stage in a phase II study with Melan-A-specific T-cell clones generated from patient blood. During the period required for T-cell clone generation, the patients were treated by dacarbazine. Every patient received a T-cell clone suspension followed by subcutaneous injections of interleukin 2 and interferon alpha. Patients were monitored until disease progression occurred. We succeeded in obtaining autologous Melan-A-specific cytotoxic T lymphocyte clones, which were highly reactive against tumor cells for all the patients. Of the 14 patients treated, six (43%) experienced an objective response (CR + PR) with long-term complete remission for two patients (1 CR for 5 years and 1 CR for 28 months). Furthermore, we showed that all the clinical responses were significantly associated with in vivo expansion of the Melan-A-specific T-cell repertoire. This phenomenon appeared to be significantly associated with clinical responses. Thus, over the course of an adoptive cell transfer, monitoring this melanoma-specific T-cell expansion in patient blood appears crucial for predicting the clinical efficiency of such an immunological approach.

8 [354]. Lucas, K. G., D. Salzman, et al. (2004). "Adoptive immunotherapy with allogeneic Epstein-Barr virus (EBV)-specific cytotoxic T-lymphocytes for recurrent, EBV-positive Hodgkin disease." Cancer **100**(9): 1892-1901.

BACKGROUND: It has been shown that adoptive immunotherapy with Epstein-Barr virus (EBV)-specific cytotoxic T-lymphocytes (CTL) is effective for the treatment of EBV-induced lymphoproliferative disease in stem cell transplantation recipients and organ transplantation recipients. The role of EBV CTL in other tumors for which this virus has been implicated in pathogenesis, such as EBV-positive Hodgkin disease (HD), has not been demonstrated clearly. METHODS: To investigate the antitumor effects and toxicity of allogeneic EBV CTL in EBV-positive HD, the authors initiated a

pilot trial in which EBV CTL were cultured from allogeneic, partially human leukocyte antigen-matched donors and were infused into patients who had therapy-refractory disease. The first cohort of 3 patients (Cohort I) received 3 separate infusions of EBV CTL (5.0 x 10(6) EBV CTL/kg per dose), and the second cohort (Cohort II) received 30 mg/m(2) per day of fludarabine for 3 days followed by a single CTL infusion (1.5 x 10(7) EBV CTL/kg). RESULTS: All three patients in Cohort I had decreases in measurable disease after EBV CTL infusions, and one of those patients was without evidence of disease 22 months after infusion. Two of 3 patients in Cohort II had decreases in measurable disease, although it was not determined whether those decreases were related to fludarabine or to CTL, and 1 patient in Cohort II had 7 months without disease progression. Unlike the patients in Cohort I, fludarabine recipients did not have increases in antidonor CTL responses. Donor cells could not be detected in any of the CTL recipients. CONCLUSIONS: Adoptive immunotherapy with allogeneic EBV CTL was safe for patients with recurrent, refractory, EBV-positive HD; and clinical responses may be observed without the establishment of detectable donor lymphoid chimerism.

9 [308]. Moosmann, A., I. Bigalke, et al. (2010). "Effective and long-term control of EBV PTLD after transfer of peptide-selected T cells." Blood **115**(14): 2960-2970.

Posttransplantation lymphoproliferative disease (PTLD) associated with Epstein-Barr virus (EBV) is a life-threatening complication after allogeneic hematopoietic stem cell transplantation. PTLD is efficiently prevented by adoptive transfer of EBV-specific T cells from the donor. To make EBV-specific T cells available in urgent clinical situations, we developed a rapid protocol for their isolation by overnight stimulation of donor blood cells with peptides derived from 11 EBV antigens, interferon-gamma surface capture, and immunomagnetic separation. Six patients with PTLD received 1 transfusion of EBV-specific T cells. No response was seen in 3 patients who had late-stage disease with multiorgan dysfunction at the time of T-cell transfer. In 3 patients who received T cells at an earlier stage of disease, we observed complete and stable remission of PTLD. Two patients have remained free from EBV-associated disease for more than 2 years. CD8(+) T cells specific for EBV early antigens rapidly expanded after T-cell transfer, temporarily constituted greater than 20% of all peripheral blood lymphocytes, and were maintained throughout the observation period. Thus, a rapid and sustained reconstitution of a protective EBV-specific T-cell memory occurred after the infusion of small numbers of directly isolated EBV-specific T cells.

10 [344]. Porter, D. L., B. L. Levine, et al. (2006). "A phase 1 trial of donor lymphocyte infusions expanded and activated ex vivo via CD3/CD28 costimulation." Blood **107**(4): 1325-1331.

Donor lymphocyte infusions (DLIs) induce potent graft versus tumor (GVT) effects for relapsed chronic myelogenous leukemia (CML) after allogeneic stem cell transplantation (SCT) but are disappointing for other diseases. Disease resistance can occur if donor T cells are not appropriately activated in vivo. Ex vivo T-cell activation might overcome disease-induced anergy and augment GVT activity. We performed a phase 1 trial of ex vivo-activated DLI (aDLI) for 18 patients with relapse after SCT. Activated donor T cells are produced through costimulation with anti-CD3- and anti-CD28-coated beads. Patients with aggressive malignancies received induction chemotherapy, and all patients received conventional DLI (median, 1.5 x 10(8) mononuclear cells/kg) followed 12 days later by aDLI. Activated DLI was dose escalated from 1 x 10(6) to 1 x 10(8) CD3+ cells per kilogram in 5 levels. Seven patients developed acute graft versus host disease (GVHD) (5 grade I-II, 2 grade III), and 4 developed chronic GVHD. Eight patients achieved complete remission, including 4 of 7 with acute lymphocytic leukemia (ALL), 2 of 4 with acute myelogenous leukemia (AML), 1 with chronic lymphocytic leukemia (CLL), and 1 of 2 with non-Hodgkin lymphoma (NHL). Four complete responders relapsed while 4 remain alive in remission a median 23 months after aDLI. Overall, 10 of 18 remain alive 11 to 53 months after aDLI. Adoptive transfer of costimulated activated allogeneic T cells is feasible, does not result in excessive GVHD, and may contribute to durable remissions in diseases where conventional DLI has been disappointing.

11 [324]. Till, B. G., M. C. Jensen, et al. (2008). "Adoptive immunotherapy for indolent non-Hodgkin lymphoma and mantle cell lymphoma using genetically modified autologous CD20-specific T cells." Blood **112**(6): 2261-2271.

Adoptive immunotherapy with T cells expressing a tumor-specific chimeric T-cell receptor is a promising approach to cancer therapy that has not previously been explored for the treatment of lymphoma in human subjects. We report the results of a proof-of-concept clinical trial in which patients with relapsed or refractory indolent B-cell lymphoma or mantle cell lymphoma were treated with autologous T cells genetically modified by electroporation with a vector plasmid encoding a CD20-specific chimeric T-cell receptor and neomycin resistance gene. Transfected cells were immunophenotypically similar to CD8(+) effector cells and showed CD20-specific cytotoxicity in vitro. Seven patients received a total of 20 T-cell infusions, with minimal toxicities. Modified T cells persisted in vivo 1 to 3 weeks in the first 3 patients, who received T cells produced by limiting

dilution methods, but persisted 5 to 9 weeks in the next 4 patients who received T cells produced in bulk cultures followed by 14 days of low-dose subcutaneous interleukin-2 (IL-2) injections. Of the 7 treated patients, 2 maintained a previous complete response, 1 achieved a partial response, and 4 had stable disease. These results show the safety, feasibility, and potential antitumor activity of adoptive T-cell therapy using this approach. This trial was registered at www.clinicaltrials.gov as #NCT00012207.

TOPIC: Epstein-Barr virus (EBV) Positive Lymphomas

1 [373]. Kluin-Nelemans, H. C., J. L. Coenen, et al. (2008). "EBV-positive immunodeficiency lymphoma after alemtuzumab-CHOP therapy for peripheral T-cell lymphoma." Blood **112**(4): 1039-1041.

Chemotherapy with alemtuzumab and the combination of cyclophosphamide, adriamycin, oncovin, and prednisone (CHOP) has become experimental trial therapy for aggressive T-cell lymphoma. Several multicenter phase 3 trials will incorporate this scheme. As part of an ongoing phase 2 trial in which we recently treated 20 patients with 8 cycles of CHOP every 2 weeks with 3 additional doses of 30 mg alemtuzumab per cycle, we observed the development of Epstein-Barr virus (EBV)-positive lymphoproliferative disease, after completion of the immunochemotherapy in 3 patients with peripheral T-cell lymphoma. Because the occurrence of EBV-positive lymphoproliferative disease is rare after alemtuzumab monotherapy, such as is given for chronic lymphocytic leukemia, we think that early reporting of this potential side effect is warranted. It may be caused by intrinsic T-cell defects in patients with T-cell lymphoma, or by the combination of alemtuzumab with CHOP chemotherapy.

TOPIC: Epstein-Barr virus (EBV) Positive Lymphomas + adoptive T Cell therapy or adoptive T cell transfer

All the items found in this search were duplicates that were previously found in prior searches resulting in no remaining items for this search.

TOPIC: Selected References from Dr. Bishop

1 [507]. d'Argouges, S., S. Wissing, et al. (2009). "Combination of rituximab with blinatumomab (MT103/MEDI-538), a T cell-engaging CD19-/CD3-bispecific antibody, for highly efficient lysis of human B lymphoma cells." Leuk Res **33**(3): 465-473.

We have compared the cytotoxic activity of rituximab with that of blinatumomab (MT103/MEDI-538), a single-chain CD19-/CD3-bispecific antibody engaging human T cells. Blinatumomab consistently led to a higher degree of lysis of human lymphoma lines than rituximab, and was active at much lower concentration. The cytotoxicity mediated by blinatumomab and rituximab both caused a potent activation of pro-caspases 3 and 7 in target cells, a key event in induction of granzyme-mediated apoptotic cell death. Combination of rituximab with blinatumomab was found to greatly enhance the activity of rituximab, in particular at low effector-to-target cell ratios and at low antibody concentration.

2 [499]. Fehniger, T. A., S. Larson, et al. (2011). "A phase 2 multicenter study of lenalidomide in relapsed or refractory classical Hodgkin lymphoma." Blood **118**(19): 5119-5125.

Relapsed or refractory (rel/ref) classical Hodgkin lymphoma (cHL) remains a clinical challenge, with limited effective treatment options available after stem cell transplantation. In a multicenter phase 2 study, the efficacy of lenalidomide in rel/ref cHL patients was evaluated at a dose of 25 mg/d on days 1-21 of a 28-day cycle. Patients remained on lenalidomide until disease progression or an unacceptable adverse event (AE) occurred. Thirty-eight cHL patients were enrolled with a median of 4 (range, 2-9) prior therapies; 87% had undergone prior stem cell transplantation and 55% of patients did not respond to their last prior therapy. Of 36 evaluable patients, responses were 1 complete remission (CR), 6 partial remissions (PRs), and 5 patients with stable disease (SD) for >/= 6 months resulting in an International Working Committee (IWC) objective overall response rate (ORR) of 19% and a cytostatic ORR of 33%. Decreased chemokine (CCL17 and CCL22) plasma levels at 2 weeks were associated with a subsequent response. The treatment was well tolerated, and the most common grade 3/4 AEs were neutropenia (47%), anemia (29%), and thrombocytopenia (18%). Four patients discontinued lenalidomide because of rash, elevated transaminases/bilirubin, and cytopenias. We provide preliminary evidence of lenalidomide's activity in patients with rel/ref cHL, and therefore exploration of lenalidomide in combination with other active agents is warranted. This trial is registered at www.ClinicalTrials.gov as NCT00540007.

3 [492]. Fisher, R. I., S. H. Bernstein, et al. (2006). "Multicenter phase II study of bortezomib in patients with relapsed or refractory mantle cell lymphoma." J Clin Oncol **24**(30): 4867-4874.

PURPOSE: Evaluate response rate, duration of response (DOR), time-to-progression (TTP), overall survival (OS), and safety of bortezomib treatment in patients with relapsed or refractory mantle cell lymphoma (MCL). PATIENTS AND METHODS: Bortezomib 1.3 mg/m(2) was administered on days 1, 4, 8, and 11 of a 21-day cycle, for up to 17 cycles. Response and progression were determined using International Workshop Response Criteria, both using data from independent radiology review and by the investigators. Primary efficacy analyses were based on data from independent radiology review. RESULTS: In total, 155 patients were treated. Median number of prior therapies was one (range, one to three). Response rate in 141 assessable patients was 33% including 8% complete response (CR)/unconfirmed CR. Median DOR was 9.2 months. Median TTP was 6.2 months. Results by investigator assessments were similar. Median OS has not been reached after a median follow-up of 13.4 months. The safety profile of bortezomib was similar to previous experience in relapsed multiple myeloma. The most common adverse events grade 3 or higher were peripheral neuropathy (13%), fatigue (12%), and thrombocytopenia (11%). Death from causes that were considered to be treatment related was reported for 3% of patients. CONCLUSION: These results confirm the activity of bortezomib in relapsed or refractory MCL, with predictable and manageable toxicities. Bortezomib provides significant clinical activity in terms of durable and complete responses, and may therefore represent a new treatment option for this population with usually very poor outcome. Studies of bortezomib-based combinations in MCL are ongoing.

4 [497]. Goy, A., R. Sinha, et al. (2013). "Single-agent lenalidomide in patients with mantle-cell lymphoma who relapsed or progressed after or were refractory to bortezomib: phase II MCL-001 (EMERGE) study." J Clin Oncol **31**(29): 3688-3695.

PURPOSE: Although dose-intensive strategies or high-dose therapy induction followed by autologous stem-cell transplantation have improved the outcome for patients with mantle-cell lymphoma (MCL), most eventually relapse and subsequently respond poorly to additional therapy. Bortezomib (in the United States) and temsirolimus (in Europe) are currently the only two treatments approved for relapsed disease. Lenalidomide is an immunomodulatory agent with proven tumoricidal and antiproliferative activity in MCL. The MCL-001 (EMERGE) trial is a global, multicenter phase II study examining the safety and efficacy of lenalidomide in patients who had relapsed or were refractory to bortezomib. PATIENTS AND METHODS: Lenalidomide 25 mg orally was administered on days 1 through 21 every 28 days until disease progression

or intolerance. Primary end points were overall response rate (ORR) and duration of response (DOR); secondary
end points included complete response (CR) rate, progression-free survival (PFS), overall survival (OS), and safety. RESULTS: In all, 134 patients were enrolled with a median age of 67 years and a median of four prior therapies (range, two to 10 prior therapies). The ORR was 28% (7.5% CR/CR unconfirmed) with rapid time to response (median, 2.2 months) and a median DOR of 16.6 months (95% CI, 7.7 to 26.7 months). Median PFS was 4.0 months (95% CI, 3.6 to 5.6 months), and median OS was 19.0 months (95% CI, 12.5 to 23.9 months). The most common grade 3 to 4 adverse events were neutropenia (43%), thrombocytopenia (28%), anemia (11%), pneumonia (8%), and fatigue (7%). CONCLUSION: The MCL-001 study demonstrated durable efficacy of lenalidomide with a predictable safety profile in heavily pretreated patients with MCL who had all relapsed or progressed after or were refractory to bortezomib.

5 [493]. Kane, R. C., R. Dagher, et al. (2007). "Bortezomib for the treatment of mantle cell lymphoma." Clin Cancer Res **13**(18 Pt 1): 5291-5294.

PURPOSE: To describe the Food and Drug Administration review and marketing approval considerations for bortezomib (Velcade) for the treatment of patients with mantle cell lymphoma. EXPERIMENTAL DESIGN: Food and Drug Administration reviewed a multicenter study of bortezomib in 155 patients with progressive mantle cell lymphoma after at least one prior therapy. RESULTS: Seventy-seven percent were stage IV, and 75% had one or more extranodal sites of disease. Prior therapy included an anthracycline or mitoxantrone, cyclophosphamide, and rituximab. Median age was 65 years. All received bortezomib 1.3 mg/m(2) i.v. on days 1, 4, 8, and 11 of each 3-week cycle. The primary end point was response. Response and progression were determined by independent review of serial computed tomography scans using International Lymphoma Workshop Response Criteria. The overall response rate was 31%, including complete response (CR) plus CR unconfirmed (CRu) plus partial response; median response duration was 9.3 months. The CR plus CRu response rate was 8% with a median duration of 15.4 months. Adverse events were similar to those observed previously for bortezomib. The most commonly reported treatment-emergent adverse events were asthenia (72%), peripheral neuropathies (55%), constipation (50%), diarrhea (47%), nausea (44%), and anorexia (39%). The most common adverse event leading to discontinuation was neuropathy. CONCLUSIONS: Bortezomib received regular approval for the treatment of patients with mantle cell lymphoma in relapse after prior therapy.

6 [506]. Kochenderfer, J. N., M. E. Dudley, et al. (2014). "Chemotherapy-Refractory Diffuse Large B-Cell Lymphoma and Indolent B-Cell Malignancies Can Be Effectively Treated With Autologous T Cells Expressing an Anti-CD19 Chimeric Antigen Receptor." J Clin Oncol.

PURPOSE: T cells can be genetically modified to express an anti-CD19 chimeric antigen receptor (CAR). We assessed the safety and efficacy of administering autologous anti-CD19 CAR T cells to patients with advanced CD19+ B-cell malignancies. PATIENTS AND METHODS: We treated 15 patients with advanced B-cell malignancies. Nine patients had diffuse large B-cell lymphoma (DLBCL), two had indolent lymphomas, and four had chronic lymphocytic leukemia. Patients received a conditioning chemotherapy regimen of cyclophosphamide and fludarabine followed by a single infusion of anti-CD19 CAR T cells. RESULTS: Of 15 patients, eight achieved complete remissions (CRs), four achieved partial remissions, one had stable lymphoma, and two were not evaluable for response. CRs were obtained by four of seven evaluable patients with chemotherapy-refractory DLBCL; three of these four CRs are ongoing, with durations ranging from 9 to 22 months. Acute toxicities including fever, hypotension, delirium, and other neurologic toxicities occurred in some patients after infusion of anti-CD19 CAR T cells; these toxicities resolved within 3 weeks after cell infusion. One patient died suddenly as a result of an unknown cause 16 days after cell infusion. CAR T cells were detected in the blood of patients at peak levels, ranging from nine to 777 CAR-positive T cells/mu L. CONCLUSION: This is the first report to our knowledge of successful treatment of DLBCL with anti-CD19 CAR T cells. These results demonstrate the feasibility and effectiveness of treating chemotherapy-refractory B-cell malignancies with anti-CD19 CAR T cells. The numerous remissions obtained provide strong support for further development of this approach.

7 [496]. Morrison, V. A., S. H. Jung, et al. (2014). "Therapy with bortezomib plus lenalidomide for relapsed/refractory mantle cell lymphoma: final results of a phase II trial (CALGB 50501)." Leuk Lymphoma: 1-7.

Cancer and Leukemia Group B designed a phase II trial of lenalidomide + bortezomib for relapsed/refractory mantle cell lymphoma (MCL). Induction therapy was lenalidomide (days 1-14) plus bortezomib (days 1/4/8/11), every 21 days for eight cycles. Complete/partial responders (CR, PR) received maintenance lenalidomide (days 1-14) and bortezomib (days 1/8), every 21 days. Primary endpoint was overall response rate; secondary endpoints were CR rate, progression-free (PFS), event-free (EFS) and overall survival (OS). Fifty-three eligible patients, median age 67 years, were accrued. Median number of cycles received was 4 (range, 1-82).

Median followup was 46 (range, 12-67) months. Best response was CR 15%, PR 25%. 5/8 CR, and 4/13 PR patients received maintenance. Six CR and one PR patient remain in remission (median, 3.2 years). Thirty-three (62%) patients have died. One-year PFS, EFS and OS are 40%, 25% and 68%, respectively. This combination will not be pursued further.

8 [498]. Morschhauser, F., O. Fitoussi, et al. (2013). "A phase 2, multicentre, single-arm, open-label study to evaluate the safety and efficacy of single-agent lenalidomide (Revlimid) in subjects with relapsed or refractory peripheral T-cell non-Hodgkin lymphoma: the EXPECT trial." Eur J Cancer **49**(13): 2869-2876.

BACKGROUND: This multicentre, single-arm, open-label phase 2 trial investigated the efficacy and safety of lenalidomide monotherapy in patients with relapsed/refractory peripheral T-cell lymphoma (PTCL). METHODS: Patients received oral lenalidomide 25mg once daily on days 1-21 of each 28-day cycle for a maximum of 24 months, until disease progression or development of unacceptable adverse events (AEs). The primary end-point was efficacy; safety was evaluated as a secondary end-point. This study was registered with ClinicalTrials.gov, number NCT00655668. FINDINGS: A total of 54 patients with PTCL were treated. The overall response rate was 22% (12 of 54), including complete response (CR) or unconfirmed CR (CRu) in 11% of patients; 31% of patients with angioimmunoblastic T-cell lymphoma (AITL) responded (CR/CRu in 15% of patients). The median progression-free survival and median response duration were 2.5 and 3.6 months, respectively, in the intent-to-treat population, and 4.6 and 3.5 months, respectively, in patients with AITL. Thrombocytopenia and neutropenia were the most common grade 3 or 4 haematological AEs, in 11 (20%) and 8 (15%) patients, respectively. Overall, 19 patients (35%) experienced at least 1AE leading to study dose interruption or reduction (commonly neutropenia or thrombocytopenia). Serious AEs were observed in 54% of patients and 12 patients died during the study; lymphoma progression (n=6); and acute respiratory distress syndrome, dyspnea, lung infiltration, neutropenic sepsis, pneumonia and cerebral ischaemia (n=1 each). INTERPRETATION: Lenalidomide exhibited single-agent activity in heavily pretreated patients with PTCL, particularly in patients with AITL. Future development is warranted in specific histologies, such as AITL, and in combination with chemotherapy or other agents considered active in PTCL. FUNDING: Celgene Corporation.

9 [502]. Ricciardelli, I., M. P. Blundell, et al. (2014). "Towards gene therapy for EBV-associated posttransplant lymphoma with genetically modified EBV-specific cytotoxic T cells." Blood **124**(16): 2514-2522.

Epstein-Barr virus (EBV)-associated posttransplant lymphoma (PTLD) is a major cause of morbidity/mortality after hematopoietic stem cell (SCT) or solid organ (SOT) transplant. Adoptive immunotherapy with EBV-specific cytotoxic lymphocytes (CTLs), although effective in SCT, is less successful after SOT where lifelong immunosuppression therapy is necessary. We have genetically engineered EBV-CTLs to render them resistant to calcineurin (CN) inhibitor FK506 through retroviral transfer of a calcineurin A mutant (CNA12). Here we examined whether or not FK506-resistant EBV-CTLs control EBV-driven tumor progression in the presence of immunosuppression in a xenogeneic mouse model. NOD/SCID/IL2rgamma(null) mice bearing human B-cell lymphoma were injected with autologous CTLs transduced with either CNA12 or eGFP in the presence/absence of FK506. Adoptive transfer of autologous CNA12-CTLs induced dramatic lymphoma regression despite the presence of FK506, whereas eGFP-CTLs did not. CNA12-CTLs persisted longer, homed to the tumor, and expanded more than eGFP-CTLs in mice treated with FK506. Mice receiving CNA12-CTLs and treated with FK506 survived significantly longer than control-treated animals. Our results demonstrate that CNA12-CTL induce regression of EBV-associated tumors in vivo despite ongoing immunosuppression. Clinical application of this novel approach may enhance the efficacy of adoptive transfer of EBV-CTL in SOT patients developing PTLD without the need for reduction in immunosuppressive therapy.

10 [505]. Rooney, C. M., C. A. Smith, et al. (1998). "Infusion of cytotoxic T cells for the prevention and treatment of Epstein-Barr virus-induced lymphoma in allogeneic transplant recipients." Blood **92**(5): 1549-1555.

Epstein-Barr virus (EBV) causes potentially lethal immunoblastic lymphoma in up to 25% of children receiving bone marrow transplants from unrelated or HLA-mismatched donors. Because this complication appears to stem from a deficiency of EBV-specific cytotoxic T cells, we assessed the safety and efficacy of donor-derived polyclonal (CD4(+) and CD8(+)) T-cell lines as immunoprophylaxis and treatment for EBV-related lymphoma. Thirty-nine patients considered to be at high risk for EBV-induced lymphoma each received 2 to 4 intravenous infusions of donor-derived EBV-specific T lymphocytes, after they had received T-cell-depleted bone marrow from HLA-matched unrelated donors (n = 33) or mismatched family members (n = 6). The immunologic effects of this therapy were monitored during and after the infusions. Infused cells were identified by detection of the neo marker gene. EBV-specific T cells bearing the neo marker were identified in all but 1 of the patients. Serial analysis of DNA detected the marker gene for as long as 18 weeks in unmanipulated peripheral blood

mononuclear cells and for as long as 38 months in regenerated lines of EBV-specific cytotoxic T cells. Six patients (15.5%) had greatly increased amounts of EBV-DNA on study entry (>2, 000 genome copies/10(6) mononuclear cells), indicating uncontrolled EBV replication, a complication that has had a high correlation with subsequent development of overt lymphoma. All of these patients showed 2 to 4 log decreases in viral DNA levels within 2 to 3 weeks after infusion and none developed lymphoma, confirming the antiviral activity of the donor-derived cells. There were no toxic effects that could be attributed to prophylactic T-cell therapy. Two additional patients who did not receive prophylaxis and developed overt immunoblastic lymphoma responded fully to T-cell infusion. Polyclonal donor-derived T-cell lines specific for EBV proteins can thus be used safely to prevent EBV-related immunoblastic lymphoma after allogeneic marrow transplantation and may also be effective in the treatment of established disease.

11 [503]. Till, B. G., M. C. Jensen, et al. (2012). "CD20-specific adoptive immunotherapy for lymphoma using a chimeric antigen receptor with both CD28 and 4-1BB domains: pilot clinical trial results." Blood **119**(17): 3940-3950.

Cellular immune responses have the potential to elicit dramatic and sustained clinical remissions in lymphoma patients. Recent clinical trial data demonstrate that modification of T cells with chimeric antigen receptors (CARs) is a promising strategy. T cells containing CARs with costimulatory domains exhibit improved activity against tumors. We conducted a pilot clinical trial testing a "third-generation" CD20-specific CAR with CD28 and 4-1BB costimulatory domains in patients with relapsed indolent B-cell and mantle cell lymphomas. Four patients were enrolled, and 3 received T-cell infusions after cyclophosphamide lymphodepletion. Treatment was well tolerated, although one patient developed transient infusional symptoms. Two patients without evaluable disease remained progression-free for 12 and 24 months. The third patient had an objective partial remission and relapsed at 12 months after infusions. Modified T cells were detected by quantitative PCR at tumor sites and up to 1 year in peripheral blood, albeit at low levels. No evidence of host immune responses against infused cells was detected. In conclusion, adoptive immunotherapy with CD20-specific T cells was well tolerated and was associated with antitumor activity. We will pursue alternative gene transfer technologies and culture conditions in future studies to improve CAR expression and cell production efficiency.

12 [495]. Vitolo, U., A. Chiappella, et al. (2014). "Lenalidomide plus R-CHOP21 in elderly patients with untreated diffuse large B-cell lymphoma: results of the REAL07 open-label, multicentre, phase 2 trial." Lancet Oncol **15**(7): 730-737.

BACKGROUND: Up to 40% of elderly patients with untreated diffuse large B-cell lymphoma (DLBCL) given a regimen of rituximab, cyclophosphamide, doxorubicin, vincristine, and prednisolone every 21 days (R-CHOP21) relapse or develop refractory disease. Lenalidomide has high activity in relapsed or refractory aggressive B-cell lymphomas. In phase 2 of the REAL07 trial, we aimed to establish the safety and efficacy of the combination of lenalidomide and R-CHOP21 in elderly patients with untreated DLBCL. METHODS: REAL07 was an open-label, multicentre trial that was done in 13 centres in Italy and one in Germany. Eligible patients were aged 60-80 years; had newly diagnosed, untreated, CD20-positive, Ann Arbor stage II-IV DLBCL or grade 3b follicular lymphoma; had an Eastern Cooperative Oncology Group performance status of 0-2; had an International Prognostic Index (IPI) risk of low-intermediate, intermediate-high, or high; and were fit according to comprehensive geriatric assessment. Participants were to receive 15 mg oral lenalidomide on days 1-14 of six 21-day cycles, and standard doses of R-CHOP21 chemotherapy (375 mg/m(2) intravenous rituximab, 750 mg/m(2) intravenous cyclophosphamide, 50 mg/m(2) intravenous doxorubicin, and 1.4 mg/m(2) intravenous vincristine on day 1, and 40 mg/m(2) oral prednisone on days 1-5). The primary endpoint was frequency of overall response (complete response [CR] and partial response [PR]), which was assessed by (18)F-fluorodeoxyglucose ((18)F-FDG) PET at the end of the treatment. Analyses were by intention to treat. This trial is registered with ClinicalTrials.gov, number NCT00907348. FINDINGS: 49 patients were included in phase 2: nine had been enrolled into phase 1 between Oct 23, 2008, and June 4, 2009, and had received the maximum tolerated dose of

15 mg lenalidomide; and 40 were enrolled into phase 2 between April 28, 2010, and June 3, 2011. 45 patients (92%, 95% CI 81-97) achieved a response (42 [86%] CR; three [6%] PR). Three patients (6%) did not respond and one (2%) died for reasons unrelated to treatment or disease. 277 (94%) of 294 planned cycles of lenalidomide and R-CHOP21 were completed. Grade 3-4 neutropenia was reported in 87 cycles (31%), grade 3-4 leukopenia in 77 (28%), and grade 3-4 thrombocytopenia in 35 (13%). No grade 4 non-haematological adverse events were reported. No patients died during the study as a result of toxic effects. INTERPRETATION: Lenalidomide with R-CHOP21 is effective and safe in elderly patients with untreated DLBCL. FUNDING: Fondazione Italiana Linfomi and Celgene.

13 [494]. Wang, M., L. Fayad, et al. (2012). "Lenalidomide in combination with rituximab for patients with relapsed or refractory mantle-cell lymphoma: a phase 1/2 clinical trial." Lancet Oncol **13**(7): 716-723.

BACKGROUND: The combination of rituximab and lenalidomide has shown promise for the treatment of mantle-cell lymphoma (MCL) in preclinical studies. We aimed to identify the maximum tolerated dose (MTD) of lenalidomide when combined with rituximab in a phase 1 trial and to assess the efficacy and safety of this combination in a phase 2 trial in patients with relapsed or refractory MCL. METHODS: Patients with relapsed or refractory MCL who had received one to four previous lines of treatment were enrolled in this single-arm, open-label, phase 1/2 trial at MD Anderson Cancer Center. In phase 1, to identify the MTD of lenalidomide, four patient cohorts received escalating doses (10, 15, 20, and 25 mg) of daily oral lenalidomide on days 1-21 of each 28-day cycle. 375 mg/m(2) intravenous rituximab was also administered in four weekly doses during cycle 1 only. In phase 2, patients received rituximab plus the MTD of lenalidomide, following the same cycles as for phase 1. Treatment in both phases continued until disease progression, stem-cell transplantation, or

severe toxicity. The primary efficacy endpoint was overall response (complete or partial response). The secondary efficacy endpoint was survival. We used the Kaplan-Meier method to estimate response duration, progression-free survival, and overall survival. Analysis was by intention to treat. This study is registered with ClinicalTrials.gov, number NCT00294632. FINDINGS: 52 patients were enrolled between Feb 10, 2006 and July 30, 2009, 14 in phase 1 and 44 (including six patients who received the MTD of lenalidomide in the phase 1 portion) in phase 2. The MTD was 20 mg lenalidomide. One patient who was treated with 25 mg lenalidomide developed a grade 4 non-neutropenic infection and died. In the phase 2 portion of the study, grade 3-4 haematological toxicities included neutropenia (29 patients), lymphopenia (16 patients), leucopenia (13 patients), and thrombocytopenia (ten patients). There were only two episodes of febrile neutropenia. Among 44 patients in phase 2, 25 (57%) had an overall response: 16 (36%) had a complete response and nine (20%) had a partial response. The median response duration was 18.9 months (95% CI 17.0 months to not reached [NR]). The median progression-free survival was 11.1 months (95% CI 8.3 to 24.9 months), and the median overall survival was 24.3 months (19.8 months to NR). Five of 14 patients who had received bortezomib treatment before enrolment achieved an overall response. INTERPRETATION: Oral lenalidomide plus rituximab is well tolerated and effective for patients with relapsed or refractory MCL. FUNDING: Celgene.

14 [501]. Wiernik, P. H., I. S. Lossos, et al. (2008). "Lenalidomide monotherapy in relapsed or refractory aggressive non-Hodgkin's lymphoma." J Clin Oncol **26**(30): 4952-4957.

PURPOSE: The major cause of death in aggressive lymphoma is relapse or nonresponse to initial therapy. Lenalidomide has activity in a variety of hematologic malignancies, including non-Hodgkin's lymphoma (NHL). We report the results of a phase II, single-arm, multicenter trial evaluating the safety and efficacy of lenalidomide oral monotherapy in patients with relapsed or refractory aggressive NHL. PATIENTS AND METHODS: Patients were treated with oral lenalidomide 25 mg once daily on days 1 to 21, every 28 days, for 52 weeks, until disease progression or intolerance. The primary end point was response; secondary end points included duration of response, progression-free survival (PFS), and safety. RESULTS: Forty-nine patients with a median age of 65 years received lenalidomide in this study. The most common histology was diffuse large B-cell lymphoma (53%), and patients had received a median of four prior treatment regimens for NHL. An objective response rate of 35% was observed in 49 treated patients, including a 12% rate of complete response/unconfirmed complete response. Responses were observed in each aggressive histologic subtype tested (diffuse large B-cell, follicular center grade 3, mantle cell, and transformed lymphomas). Of patients with stable disease or partial response at first assessment, 25% improved with continued treatment. Estimated median duration of response was 6.2 months, and median PFS was 4.0 months. The most common grade 4 adverse events were neutropenia (8.2%) and thrombocytopenia (8.2%); the most common grade 3 adverse events were neutropenia (24.5%), leukopenia (14.3%), and thrombocytopenia (12.2%). CONCLUSION: Oral lenalidomide monotherapy is active in relapsed or refractory aggressive NHL, with manageable side effects.

15 [500]. Witzig, T. E., P. H. Wiernik, et al. (2009). "Lenalidomide oral monotherapy produces durable responses in relapsed or refractory indolent non-Hodgkin's Lymphoma." J Clin Oncol **27**(32): 5404-5409.

PURPOSE: Lenalidomide is a novel immunomodulatory agent with antiproliferative activities. Given its efficacy in a wide range of hematologic malignancies, we conducted a phase II trial (NHL-001) of single-agent lenalidomide in indolent non-Hodgkin's lymphoma (NHL). PATIENTS AND METHODS: Patients with relapsed/refractory indolent NHL were eligible, with no limit on the number of previous therapies. Oral lenalidomide 25 mg was self-administered once daily on days 1 to 21 of

every 28-day cycle for up to 52 weeks as tolerated, or until disease progression. The primary end point was objective response rate (ORR), with secondary end points of duration of response (DR), progression-free survival (PFS), and safety. RESULTS: Forty-three enrolled patients were assessable for response and safety. Patients received a median of three prior systemic therapies (range, 1 to 17) and half were refractory to last therapy. ORR was 23% (10 of 43), including a 7% complete response (CR) or unconfirmed CR rate. Twenty-seven percent (six of 22) of patients with follicular lymphoma grade 1 or 2, and 22% (four of 18) with small lymphocytic lymphoma responded to therapy. Median DR was not reached, but was longer than 16.5 months with seven of 10 responses ongoing at 15 to 28 months. Median PFS for the whole group was 4.4 months (95% CI, 2.5 to 10.4 months). Adverse events were predictable and manageable; the most common grade 3 or 4 adverse events were neutropenia (30% and 16%, respectively) and thrombocytopenia (14% and 5%, respectively). CONCLUSION: Oral lenalidomide monotherapy produces durable responses with manageable adverse events in patients with relapsed/refractory indolent NHL, warranting further investigation of treatment for indolent NHL.
