# Supplementary material for: The Society for Immunotherapy of Cancer consensus statement on immunotherapy for the treatment of hematologic malignancies: multiple myeloma, lymphoma, and acute leukemia
Source: J Immunother Cancer. 2016 Dec 20;4:90. doi: 10.1186/s40425-016-0188-z (PMC5168808; doi:10.1186/s40425-016-0188-z)
Supplement: Additional file 3: — Annotated Bibliography (Myeloma), Annotated Bibliography (Lymphoma), and Annotated Bibliography (Leukemia). (ZIP 1812 kb) [file 40425_2016_188_MOESM3_ESM.zip › Add/APPENDIX II-Supplementary Biobliography III Cancer Immunotherapy Guidelines Acute Leukemia.pdf]

Cancer Immunotherapy Guidelines (Leukemia)

An Annotated Bibliography of the Literature (in order of topic)

Society for Immunotherapy of Cancer

November 30, 2014

Table of Contents

TOPIC: AML + Epigenetic Therapy 8

TOPIC: AML + Hypomethylating Agents or Azacytidine or Decitabine 8

TOPIC: AML + Monoclonal Antibodies 28

TOPIC: ALL + Monoclonal Antibodies or Rituximab or Blinatumomab 38

TOPIC: ALL + Rituximab or Blinatumomab 38

TOPIC: AML + Checkpoint Blockade 42

TOPIC: AML + CAR or CART 43

TOPIC: ALL + CAR or CART 45

Note: The bibliography is organized in topic order and then in alphabetical order by author. Duplicates and selected references have been removed in this bibliography.

Note: The bibliography is organized in topic order and then in alphabetical order by author. Duplicates and selected references (by Dr. Boyiadzis) have been removed in this bibliography. The searches were conducted on 10/25/14. 11/5/14, and 11/7/14 in the sequence and with the limits as follows:

Leukemia Immunotherapy Literature Searches Conducted Oct 25, Nov 5, and Nov 7, 2014

Search Terms	Date Limits	Query Translation	Date Search Completed	File Name with Dupes/File Name without Dupes/File Name without Dupes plus Adds/File name with Dupes and Drops removed	EndNote record numbers	total records found	total # dupes	total # drops	Resulting # of records in bibliography
AML + Epigenetic Therapy	2004-2014	((Acute[All Fields] AND myeloid[All Fields] AND luekemia[All Fields]) OR ("acute myelogenous leukaemia"[All Fields] OR "leukemia, myeloid, acute"[MeSH Terms] OR ("leukemia"[All Fields] AND "myeloid"[All Fields] AND "acute"[All Fields]) OR "acute myeloid leukemia"[All Fields] OR ("acute"[All Fields] AND "myelogenous"[All Fields] AND "leukemia"[All Fields]) OR "acute myelogenous leukemia"[All Fields])) AND (("epigenomics"[MeSH Terms] OR "epigenomics"[All Fields] OR "epigenetic"[All Fields]) AND ("therapy"[Subheading] OR "therapy"[All Fields] OR "therapeutics"[MeSH Terms] OR "therapeutics"[All Fields])) AND ((Clinical Trial[ptyp] OR Controlled Clinical Trial[ptyp] OR Randomized Controlled Trial[ptyp] OR Meta-Analysis[ptyp] OR Practice Guideline[ptyp]) AND ("2004/01/01"[PDAT] : "2014/12/31"[PDAT]) AND "humans"[MeSH Terms])	10/25/2014	Leukemia 110724.enl/ Leukemia 11072014 Dupes removed.enl/ Leukemia 11302014 Dupes & Drops Removed.enl	1-11	11	0	10	1
AML + hypomethylating agents (OR) Azacytidine (OR) Decitabine	2004-2014	(((("leukemia, myeloid, acute"[MeSH Terms] OR ("leukemia"[All Fields] AND "myeloid"[All Fields] AND "acute"[All Fields]) OR "acute myeloid leukemia"[All Fields] OR ("acute"[All Fields] AND "myeloid"[All Fields] AND "leukemia"[All Fields])) OR ("acute myelogenous leukaemia"[All Fields] OR "leukemia, myeloid, acute"[MeSH Terms] OR ("leukemia"[All Fields] AND "myeloid"[All Fields] AND "acute"[All Fields]) OR "acute myeloid leukemia"[All Fields] OR ("acute"[All Fields] AND "myelogenous"[All Fields] AND "leukemia"[All Fields]) OR "acute myelogenous leukemia"[All Fields])) AND (hypomethylating[All Fields] AND agents[All Fields])) OR ("azacitidine"[MeSH Terms] OR "azacitidine"[All Fields] OR "azacytidine"[All Fields])) OR ("decitabine"[Supplementary Concept] OR "decitabine"[All Fields]) AND ((Clinical Trial[ptyp] OR Controlled Clinical Trial[ptyp] OR Randomized Controlled Trial[ptyp] OR Meta-Analysis[ptyp] OR Practice Guideline[ptyp]) AND ("2004/01/01"[PDAT] : "2014/12/31"[PDAT]) AND "humans"[MeSH Terms])	10/25/2014	Leukemia 110724.enl/ Leukemia 11072014 Dupes removed.enl/ Leukemia 11302014 Dupes & Drops Removed.enl	12-196	185	8	147	30
AML + monoclonal antibod* (y or ies)	2004-2014	"leukemia, myeloid, acute"[MeSH Major Topic] AND "antibodies, monoclonal"[MeSH Terms] AND ((Clinical Trial[ptyp] OR Controlled Clinical Trial[ptyp] OR Randomized Controlled Trial[ptyp] OR Meta-Analysis[ptyp] OR Practice Guideline[ptyp]) AND ("2004/01/01"[PDAT] : "2014/12/31"[PDAT]) AND "humans"[MeSH Terms])	11/5/2014	Leukemia 110724.enl/ Leukemia 11072014 Dupes removed.enl/ Leukemia 11302014 Dupes & Drops Removed.enl	197-259	63	5	45	13

**ALL + monoclonal antibod* (y or ies) (OR) Rituximab (OR) Blinatumomab**	**2004-2014**	**(("precursor cell lymphoblastic leukemia-lymphoma"[MeSH Major Topic] AND "antibodies, monoclonal"[MeSH Terms]) OR "rituximab"[Supplementary Concept]) OR "blinatumomab"[Supplementary Concept] AND ((Controlled Clinical Trial[ptyp] OR Randomized Controlled Trial[ptyp] OR Clinical Trial[ptyp] OR Meta-Analysis[ptyp] OR Practice Guideline[ptyp]) AND ("2004/01/01"[PDAT] : "2014/12/31"[PDAT]) AND "humans"[MeSH Terms])**	**11/7/2014**	**Leukemia 110724.enl/ Leukemia 11072014 Dupes removed.enl/ Leukemia 11302014 Dupes & Drops Removed.enl**	260-525	266	0	265	1

Search Terms	Date Limits	Query Translation	Date Search Completed	File Name with Dupes/File Name without Dupes/File Name without Dupes plus Adds/File name with Dupes and Drops removed	EndNote record numbers	total records found	total # dupes	total # drops	Resulting # of records in bibliography
**ALL + Rituximab (OR) Blinatumomab**	**2004-2014**	**("precursor cell lymphoblastic leukemia-lymphoma"[MeSH Major Topic] AND "rituximab"[Supplementary Concept]) OR "blinatumomab"[Supplementary Concept] AND ((Randomized Controlled Trial[ptyp] OR Practice Guideline[ptyp] OR Clinical Trial[ptyp] OR Controlled Clinical Trial[ptyp] OR Meta-Analysis[ptyp]) AND ("2004/01/01"[PDAT] : "2014/12/31"[PDAT]) AND "humans"[MeSH Terms])**	**11/5/2014**	**Leukemia 110724.enl/ Leukemia 11072014 Dupes removed.enl/ Leukemia 11302014 Dupes & Drops Removed.enl**	526-535	10	0	3	7
**AML + checkpoint blockade**	**2004-2014**	**(("leukemia, myeloid, acute"[MeSH Terms] OR ("leukemia"[All Fields] AND "myeloid"[All Fields] AND "acute"[All Fields]) OR "acute myeloid leukemia"[All Fields] OR ("acute"[All Fields] AND "myeloid"[All Fields] AND "leukemia"[All Fields])) OR ("acute myelogenous leukaemia"[All Fields] OR "leukemia, myeloid, acute"[MeSH Terms] OR ("leukemia"[All Fields] AND "myeloid"[All Fields] AND "acute"[All Fields]) OR "acute myeloid leukemia"[All Fields] OR ("acute"[All Fields] AND "myelogenous"[All Fields] AND "leukemia"[All Fields]) OR "acute myelogenous leukemia"[All Fields])) AND (("cell cycle checkpoints"[MeSH Terms] OR ("cell"[All Fields] AND "cycle"[All Fields] AND "checkpoints"[All Fields]) OR "cell cycle checkpoints"[All Fields] OR "checkpoint"[All Fields]) AND blockade[All Fields]) AND ((Clinical Trial[ptyp] OR Controlled Clinical Trial[ptyp] OR Meta-Analysis[ptyp] OR Practice Guideline[ptyp] OR Randomized Controlled Trial[ptyp]) AND ("2004/01/01"[PDAT] : "2014/12/31"[PDAT]) AND "humans"[MeSH Terms])**	**10/25/2014**	**Leukemia 110724.enl/ Leukemia 11072014 Dupes removed.enl/ Leukemia 11302014 Dupes & Drops Removed.enl**	0	0	0	0	0

AML + CAR (OR) CART	2004-2014	(((((("leukemia, myeloid, acute"[MeSH Terms] OR ("leukemia"[All Fields] AND "myeloid"[All Fields] AND "acute"[All Fields]) OR "acute myeloid leukemia"[All Fields] OR ("acute"[All Fields] AND "myeloid"[All Fields] AND "leukemia"[All Fields])) OR ("acute myelogenous leukaemia"[All Fields] OR "leukemia, myeloid, acute"[MeSH Terms] OR ("leukemia"[All Fields] AND "myeloid"[All Fields] AND "acute"[All Fields]) OR "acute myeloid leukemia"[All Fields] OR ("acute"[All Fields] AND "myelogenous"[All Fields] AND "leukemia"[All Fields]) OR "acute myelogenous leukemia"[All Fields])) AND (("chimera"[MeSH Terms] OR "chimera"[All Fields] OR "chimeric"[All Fields]) AND ("receptors, antigen"[MeSH Terms] OR ("receptors"[All Fields] AND "antigen"[All Fields]) OR "antigen receptors"[All Fields] OR ("antigen"[All Fields] AND "receptor"[All Fields]) OR "antigen receptor"[All Fields]))) OR (("chimera"[MeSH Terms] OR "chimera"[All Fields] OR "chimeric"[All Fields]) AND ("receptors, antigen"[MeSH Terms] OR ("receptors"[All Fields] AND "antigen"[All Fields]) OR "antigen receptors"[All Fields] OR ("antigen"[All Fields] AND "receptors"[All Fields])))) OR (("chimera"[MeSH Terms] OR "chimera"[All Fields] OR "chimeric"[All Fields]) AND ("receptors, antigen"[MeSH Terms] OR ("receptors"[All Fields] AND "antigen"[All Fields]) OR "antigen receptors"[All Fields] OR ("antigen"[All Fields] AND "receptor"[All Fields]) OR "antigen receptor"[All Fields]) AND ("t-lymphocytes"[MeSH Terms] OR "t-lymphocytes"[All Fields] OR "t cells"[All Fields]))) OR (("chimera"[MeSH Terms] OR "chimera"[All Fields] OR "chimeric"[All Fields]) AND ("receptors, antigen"[MeSH Terms] OR ("receptors"[All Fields] AND "antigen"[All Fields]) OR "antigen receptors"[All Fields] OR ("antigen"[All Fields] AND "receptor"[All Fields]) OR "antigen receptor"[All Fields]) AND ("t-lymphocytes"[MeSH Terms] OR "t-lymphocytes"[All Fields] OR "t cells"[All Fields])) AND ((Clinical Trial[ptyp] OR Controlled Clinical Trial[ptyp] OR Meta-Analysis[ptyp] OR Randomized Controlled Trial[ptyp] OR Practice Guideline[ptyp]) AND ("2004/01/01"[PDAT] : "2014/12/31"[PDAT]) AND "humans"[MeSH Terms])	10/25/2014	Leukemia 110724.enl/ Leukemia 11072014 Dupes removed.enl/ Leukemia 11302014 Dupes & Drops Removed.enl	536-564	29	0	26	3

Leukemia Immunotherapy Literature Searches Conducted Oct 25, Nov 5, and Nov 7, 2014

Search Terms	Date Limits	Query Translation	Date Search Completed	File Name with Dupes/File Name without Dupes/File Name without Dupes plus Adds/File name with Dupes and Drops removed	EndNote record numbers	total records found	total # dupes	total # drops	Resulting # of records in bibliography

**ALL + CAR (OR) CART**	**2004-2014**	**((((("precursor cell lymphoblastic leukemia-lymphoma"[MeSH Terms] OR ("precursor"[All Fields] AND "cell"[All Fields] AND "lymphoblastic"[All Fields] AND "leukemia-lymphoma"[All Fields]) OR "precursor cell lymphoblastic leukemia-lymphoma"[All Fields] OR ("acute"[All Fields] AND "lymphoid"[All Fields] AND "leukemia"[All Fields]) OR "acute lymphoid leukemia"[All Fields]) OR ("acute lymphoblastic leukaemia"[All Fields] OR "precursor cell lymphoblastic leukemia-lymphoma"[MeSH Terms] OR ("precursor"[All Fields] AND "cell"[All Fields] AND "lymphoblastic"[All Fields] AND "leukemia-lymphoma"[All Fields]) OR "precursor cell lymphoblastic leukemia-lymphoma"[All Fields] OR ("acute"[All Fields] AND "lymphoblastic"[All Fields] AND "leukemia"[All Fields]) OR "acute lymphoblastic leukemia"[All Fields])) AND (("chimera"[MeSH Terms] OR "chimera"[All Fields] OR "chimeric"[All Fields]) AND ("receptors, antigen"[MeSH Terms] OR ("receptors"[All Fields] AND "antigen"[All Fields]) OR "antigen receptors"[All Fields] OR ("antigen"[All Fields] AND "receptor"[All Fields]) OR "antigen receptor"[All Fields])) OR (("chimera"[MeSH Terms] OR "chimera"[All Fields] OR "chimeric"[All Fields]) AND ("receptors, antigen"[MeSH Terms] OR ("receptors"[All Fields] AND "antigen"[All Fields]) OR "antigen receptors"[All Fields] OR ("antigen"[All Fields] AND "receptors"[All Fields]))) OR (("chimera"[MeSH Terms] OR "chimera"[All Fields] OR "chimeric"[All Fields]) AND ("receptors, antigen"[MeSH Terms] OR ("receptors"[All Fields] AND "antigen"[All Fields]) OR "antigen receptors"[All Fields] OR ("antigen"[All Fields] AND "receptor"[All Fields]) OR "antigen receptor"[All Fields]) AND ("tlymphocytes"[MeSH Terms] OR "t-lymphocytes"[All Fields] OR "t cells"[All Fields])) AND ((Clinical Trial[ptyp] OR Randomized Controlled Trial[ptyp] OR Practice Guideline[ptyp] OR Controlled Clinical Trial[ptyp] OR Meta-Analysis[ptyp]) AND ("2004/01/01"[PDat] : "2014/12/31"[PDat]) AND Humans[Mesh])**	**11/7/2014**	**Leukemia 110724.enl/ Leukemia 11072014 Dupes removed.enl/ Leukemia 11302014 Dupes & Drops Removed.enl**	565-594	30	29	0	1
					**Totals**	**594**	**42**	**496**	**56**

This page intentionally left blank.

NOTE: IN THE BIBLIOGRAPHY, THE NUMBER IN BRACKETS IS THE RECORD NUMBER IN THE ENDNOTE DATABASE (e.g., 2 is the record number for the first item in this bibliography). This is the correct number to use for identifying references in the manuscript during the manuscript draft stages and for any other purposes.

TOPIC: AML + Epigenetic Therapy

1 [2]. Scandura, J. M., G. J. Roboz, et al. (2011). "Phase 1 study of epigenetic priming with decitabine prior to standard induction chemotherapy for patients with AML." Blood **118**(6): 1472-1480.

We conducted an open-label phase 1 study exploring the feasibility, safety, and biologic activity of epigenetic priming with decitabine before standard induction chemotherapy in patients with less-than-favorable risk of acute myelogenous leukemia (AML). We directly compared the clinical and DNA-hypomethylating activity of decitabine delivered at 20 mg/m(2) by either a 1-hour infusion (Arm A) or a continuous infusion (Arm B) for 3, 5, or 7 days before a single, standard induction with infusional cytarabine (100 mg/m(2) for 7 days) and daunorubicin (60 mg/m(2) x 3 doses). Toxicity was similar to that of standard induction chemotherapy alone. Although we did not identify a maximum tolerated dose, there was more gastro-intestinal toxicity with 7 days of decitabine priming. Decitabine induced DNA hypomethylation at all dose levels and there was a trend toward greater hypomethylation in CD34(+) bone marrow cells when decitabine was delivered by a short pulse (Arm A). Twenty-seven subjects (90%) responded to therapy: 17 with complete remission (57%) and 10 with partial remission (33%). Of the patients with partial remission to protocol treatment, 8 achieved remission to their next therapy, bringing the overall complete remission rate to 83%. We conclude that epigenetic priming of intensive chemotherapy can be safely delivered in an attempt to improve response rates. This trial was registered at www.clinicaltrials.gov as NCT00538876.

TOPIC: AML + Hypomethylating Agents or Azacytidine or Decitabine

1 [93]. Al-Ali, H. K., N. Jaekel, et al. (2012). "Azacitidine in patients with acute myeloid leukemia medically unfit for or resistant to chemotherapy: a multicenter phase I/II study." Leuk Lymphoma **53**(1): 110-117.

The safety and efficacy of azacitidine (5-day schedule) were assessed in a multicenter study in 40 patients (median age 72 years) with acute myeloid leukemia (AML) medically unfit for (n = 20) or resistant to chemotherapy (n

= 20) from April to October 2008. Median marrow blasts were 42%. After a median follow-up of 13 months, response (complete remission [CR]/partial remission [PR]/hematologic improvement [HI]) was 50% and 10% in newly diagnosed and relapsed/refractory patients, respectively (p = 0.008). Median time-to-response was 2.5 months with a median duration of 5.9 months. Median survival was not reached for responders versus 3.8 months for 15 (38%) patients with stable disease (p < 0.045). High-risk cytogenetics was associated with inferior survival (p = 0.05). Lower marrow blasts on day 15 of cycle 1, irrespective of pretreatment count, predicted subsequent response (p = 0.01). Azacitidine is active and well tolerated in elderly patients with newly diagnosed AML.

2 [130]. Blum, W., R. Garzon, et al. (2010). "Clinical response and miR-29b predictive significance in older AML patients treated with a 10-day schedule of decitabine." Proc Natl Acad Sci U S A **107**(16): 7473-7478.

A phase II clinical trial with single-agent decitabine was conducted in older patients (>or=60 years) with previously untreated acute myeloid leukemia (AML) who were not candidates for or who refused intensive chemotherapy. Subjects received low-dose decitabine at 20 mg/m(2) i.v. over 1 h on days 1 to 10. Fifty-three subjects enrolled with a median age of 74 years (range, 60-85). Nineteen (36%) had antecedent hematologic disorder or therapy-related AML; 16 had complex karyotypes (>or=3 abnormalities). The complete remission rate was 47% (n = 25), achieved after a median of three cycles of therapy. Nine additional subjects had no morphologic evidence of disease with incomplete count recovery, for an overall response rate of 64% (n = 34). Complete remission was achieved in 52% of subjects presenting with normal karyotype and in 50% of those with complex karyotypes. Median overall and disease-free survival durations were 55 and 46 weeks, respectively. Death within 30 days of initiation of treatment occurred in one subject (2%), death within 8 weeks in 15% of subjects. Given the DNA hypomethylating effect of decitabine, we examined the relationship of clinical response and pretreatment level of miR-29b, previously shown to target DNA methyltransferases. Higher levels of miR-29b were associated with clinical response (P = 0.02). In conclusion, this schedule of decitabine was highly active and well tolerated in this poor-risk cohort of older AML patients. Levels of miR-29b should be validated as a predictive factor for stratification of older AML patients to decitabine treatment.

3 [166]. Blum, W., R. B. Klisovic, et al. (2007). "Phase I study of decitabine alone or in combination with valproic acid in acute myeloid leukemia." J Clin Oncol **25**(25): 3884-3891.

PURPOSE: To determine an optimal biologic dose (OBD) of decitabine as a single agent and then the maximum-tolerated dose (MTD) of valproic acid (VA) combined with decitabine in acute myeloid leukemia (AML).

PATIENTS AND METHODS: Twenty-five patients (median age, 70 years) were enrolled; 12 were untreated and 13 had relapsed AML. To determine an OBD (based on a gene re-expression end point), 14 patients received decitabine alone for 10 days. To determine the MTD, 11 patients received decitabine (at OBD, days 1 through 10) plus dose-escalating VA (days 5 through 21). RESULTS: The OBD of decitabine was 20 mg/m(2)/d intravenously, with limited nonhematologic toxicity. In patients treated with decitabine plus VA, dose-limiting encephalopathy occurred in two of two patients at VA 25 mg/kg/d and one of six patients at VA 20 mg/kg/d. Drug-induced re-expression of estrogen receptor (ER) was associated with clinical response (P < or = .05). ER promoter demethylation, global DNA hypomethylation, depletion of DNA methyltransferase enzyme, and histone hyperacetylation were also observed. In an intent-to-treat analysis, the response rate was 44% (11 of 25). Of 21 assessable patients, 11 (52%) responded: four with morphologic and cytogenetic complete remission (CR; each had complex karyotype), four with incomplete CR, and three with partial remission. In untreated AML, four of nine assessable patients achieved CR. Clinical responses appeared similar for decitabine alone or with VA. CONCLUSION: Low-dose decitabine was safe and showed encouraging clinical and biologic activity in AML, but the addition of VA led to encephalopathy at relatively low doses. On the basis of these results, additional studies of decitabine (20 mg/m(2)/d for 10 days) alone or with an alternative deacetylating agent are warranted.

4 [78]. Blum, W., S. Schwind, et al. (2012). "Clinical and pharmacodynamic activity of bortezomib and decitabine in acute myeloid leukemia." Blood **119**(25): 6025-6031.

We recently reported promising clinical activity for a 10-day regimen of decitabine in older AML patients; high miR-29b expression associated with clinical response. Subsequent preclinical studies with bortezomib in AML cells have shown drug-induced miR-29b up-regulation, resulting in loss of transcriptional activation for several genes relevant to myeloid leukemogenesis, including DNA methyltransferases and receptor tyrosine kinases. Thus, a phase 1 trial of bortezomib and decitabine was developed. Nineteen poor-risk AML patients (median age 70 years; range, 32-84 years) enrolled. Induction with decitabine (20 mg/m(2) intravenously

on days 1-10) plus bortezomib (escalated up to the target 1.3 mg/m(2) on days 5, 8, 12, and 15) was tolerable, but bortezomib-related neuropathy developed after repetitive cycles. Of previously untreated patients (age >/= 65 years), 5 of 10 had CR (complete remission, n = 4) or incomplete CR (CRi, n = 1); 7 of 19 overall had CR/CRi. Pharmacodynamic analysis showed FLT3 down-regulation on day 26 of cycle 1 (P = .02). Additional mechanistic studies showed that FLT3 down-regulation was due to bortezomib-induced miR-29b up-regulation; this led to SP1 down-regulation and destruction of the SP1/NF-kappaB complex that transactivated FLT3. This study demonstrates the feasibility and preliminary clinical activity of decitabine plus bortezomib in AML and identifies FLT3 as a novel pharmacodynamic end point for future trials.

5 [161]. Borthakur, G., S. E. Ahdab, et al. (2008). "Activity of decitabine in patients with myelodysplastic syndrome previously treated with azacitidine." Leuk Lymphoma **49**(4): 690-695.

Azacitidine and decitabine are the two hypomethylating agents approved by the Food and Drug Administration for the treatment of patients with myelodysplastic syndrome (MDS). The efficacy of one agent post-failure of the other is unknown. Fourteen patients with MDS post-azacitidine failure/lack of response/intolerance were treated with decitabine. Overall three patients achieved a complete remission, and one patient had hematologic improvement, for an overall response rate of 28%. Of the responders, one stopped prior 5-azacitidine owing to disease progression, two for no response and one for severe skin toxicity. Grade 3-4 drug related side-effects were minimal. Global methylation studies in patient samples showed decrease of methylation after treatment with decitabine. As in our previous studies, there was no difference in hypomethylation between responders and nonresponders. We conclude that clinically significant responses with decitabine can be seen in patients post-azacitidine failure without significant toxicity.

6 [137]. Borthakur, G., X. Huang, et al. (2010). "Report of a phase 1/2 study of a combination of azacitidine and cytarabine in acute myelogenous leukemia and high-risk myelodysplastic syndromes." Leuk Lymphoma **51**(1): 73-78.

Cytarabine resistance characterizes relapsed and refractory acute myelogenous leukemia (AML). Restoration of cytarabine sensitivity can potentially improve treatment outcome in this setting. Acquired hypermethylation of gene promoters and associated silencing of gene expression has been implicated in chemo resistance, and drug-induced hypomethylation can improve sensitivity to cytarabine in vitro. We

conducted an adaptively randomized study of a combination of azacitidine, a hypomethylating agent, and cytarabine in 34 patients with AML. The combination administered in a concomitant fashion is safe at full doses of azacitidine and cytarabine, without unexpected toxicities. However, in this advanced AML population, it was difficult to deliver more than one cycle of therapy, and minimal anti-leukemia activity was seen in patients with relapsed/refractory disease. Complete remission was achieved in 2 of 6 minimally pre-treated patients. We conclude that the combination of azacitidine and cytarabine is feasible but has limited activity in relapsed/refractory AML.

7 [75]. Boumber, Y., H. Kantarjian, et al. (2012). "A randomized study of decitabine versus conventional care for maintenance therapy in patients with acute myeloid leukemia in complete remission." Leukemia **26**(11): 2428-2431.

8 [12]. Burke, M. J., J. K. Lamba, et al. (2014). "A therapeutic trial of decitabine and vorinostat in combination with chemotherapy for relapsed/refractory acute lymphoblastic leukemia." Am J Hematol **89**(9): 889-895.

DNA hypermethylation and histone deacetylation are pathways of leukemia resistance. We investigated the tolerability and efficacy of decitabine and vorinostat plus chemotherapy in relapse/refractory acute lymphoblastic leukemia (ALL). Decitabine (15 mg/m(2) iv) and vorinostat (230 mg/m(2) PO div BID) were given days 1-4 followed by vincristine, prednisone, PEG-asparaginase, and doxorubicin. Genome wide methylation profiles were performed in 8 matched patient bone marrow (BM) samples taken at day 0 and day 5 (postdecitabine). The median age was 16 (range, 3-54) years. All patients had a prior BM relapse, with five relapsing after allogeneic transplant. The most common nonhematological toxicities possibly related to decitabine or vorinostat were infection with neutropenia (grade 3; n = 4) and fever/neutropenia (grade 3, n = 4; grade 4, n = 1). Of the 13 eligible patients, four achieved complete remission without platelet recovery (CRp), two partial response (PR), one stable disease (SD), one progressive disease (PD), two deaths on study and three patients who did not have end of therapy disease evaluations for an overall response rate of 46.2% (CRp + PR). Following decitabine, significant genome-wide hypo-methylation was observed. Comparison of clinical responders with nonresponders identified methylation profiles of clinical and biological relevance. Decitabine and vorinostat followed by re-Induction chemotherapy was tolerable and demonstrated clinical benefit in relapsed patients with ALL. Methylation

differences were identified between responders and nonresponders indicating interpatient variation, which could impact clinical outcome. This study was registered at www.clinicaltrials.gov as NCT00882206.

9 [136]. Cashen, A. F., G. J. Schiller, et al. (2010). "Multicenter, phase II study of decitabine for the first-line treatment of older patients with acute myeloid leukemia." J Clin Oncol **28**(4): 556-561.

PURPOSE: Older patients with acute myeloid leukemia (AML) have limited treatment options because of the lack of effectiveness and the toxicity of available therapies. We investigated the efficacy and toxicity of the hypomethylating agent decitabine as initial therapy in older patients with AML. PATIENTS AND METHODS: In this multicenter, phase II study, patients older than 60 years who had AML (ie, > 20% bone marrow blasts) and no prior therapy for AML were treated with decitabine 20 mg/m(2) intravenously for 5 consecutive days of a 4-week cycle. Response was assessed by weekly CBC and bone marrow biopsy after cycle 2 and after each subsequent cycle. Patients continued to receive decitabine until disease progression or an unacceptable adverse event occurred. RESULTS: Fifty-five patients (mean age, 74 years) were enrolled and were treated with a median of three cycles (range, one to 25 cycles) of decitabine. The expert-reviewed overall response rate was 25% (complete response rate, 24%). The response rate was consistent across subgroups, including in patients with poor-risk cytogenetics and in those with a history of myelodysplastic syndrome. The overall median survival was 7.7 months, and the 30-day mortality rate was 7%. The most common toxicities were myelosuppression, febrile neutropenia, and fatigue. CONCLUSION: Decitabine given in a low-dose, 5-day regimen has activity as upfront therapy in older patients with AML, and it has acceptable toxicity and 30-day mortality.

10 [133]. Fenaux, P., N. Gattermann, et al. (2010). "Prolonged survival with improved tolerability in higher-risk myelodysplastic syndromes: azacitidine compared with low dose ara-C." Br J Haematol **149**(2): 244-249.

In the phase III AZA-001 trial, low-dose cytarabine (LDara-C), the most widely used low-dose chemotherapy in patients with higher-risk myelodysplastic syndrome (MDS) who are ineligible for intensive treatment, was found to be associated with poorer survival compared with azacitidine. This analysis further compared the efficacy and the toxicity of these two drug regimens. Before randomization, investigators preselected patients to receive a conventional care regimen, one of which was LDara-C. Of 94 patients preselected to LDara-C, 45 were

randomized to azacitidine and 49 to LDara-C. Azacitidine patients had significantly more and longer haematological responses and increased red blood cell transfusion independence. Azacitidine prolonged overall survival versus LDara-C in patients with poor cytogenetic risk, presence of -7/del(7q), and French-American-British subtypes refractory anaemia with excess blasts (RAEB) and RAEB in transformation. When analyzed per patient year of drug exposure, azacitidine treatment was associated with fewer grade 3-4 cytopenias and shorter hospitalisation time than LDara-C in these higher-risk MDS patients.

11 [153]. Fenaux, P., G. J. Mufti, et al. (2009). "Efficacy of azacitidine compared with that of conventional care regimens in the treatment of higher-risk myelodysplastic syndromes: a randomised, open-label, phase III study." Lancet Oncol **10**(3): 223-232.

BACKGROUND: Drug treatments for patients with high-risk myelodysplastic syndromes provide no survival advantage. In this trial, we aimed to assess the effect of azacitidine on overall survival compared with the three commonest conventional care regimens. METHODS: In a phase III, international, multicentre, controlled, parallel-group, open-label trial, patients with higher-risk myelodysplastic syndromes were randomly assigned one-to-one to receive azacitidine (75 mg/m(2) per day for 7 days every 28 days) or conventional care (best supportive care, low-dose cytarabine, or intensive chemotherapy as selected by investigators before randomisation). Patients were stratified by French-American-British and international prognostic scoring system classifications; randomisation was done with a block size of four. The primary endpoint was overall survival. Efficacy analyses were by intention to treat for all patients assigned to receive treatment. This study is registered with ClinicalTrials.gov, number NCT00071799. FINDINGS: Between Feb 13, 2004, and Aug 7, 2006, 358 patients were randomly assigned to receive azacitidine (n=179) or conventional care regimens (n=179). Four patients in the azacitidine and 14 in the conventional care groups received no study drugs but were included in the intention-to-treat efficacy analysis. After a median follow-up of 21.1 months (IQR 15.1-26.9), median overall survival was 24.5 months (9.9-not reached) for the azacitidine group versus 15.0 months (5.6-24.1) for the conventional care group (hazard ratio 0.58; 95% CI 0.43-0.77; stratified log-rank p=0.0001). At last follow-up, 82 patients in the azacitidine group had died compared with 113 in the conventional care group. At 2 years, on the basis of Kaplan-Meier estimates, 50.8% (95% CI 42.1-58.8) of patients in the azacitidine group were alive compared with 26.2% (18.7-34.3) in the conventional care group (p<0.0001).

Peripheral cytopenias were the most common grade 3-4 adverse events for all treatments. INTERPRETATION: Treatment with azacitidine increases overall survival in patients with higher-risk myelodysplastic syndromes relative to conventional care.

12 [135]. Fenaux, P., G. J. Mufti, et al. (2010). "Azacitidine prolongs overall survival compared with conventional care regimens in elderly patients with low bone marrow blast count acute myeloid leukemia." J Clin Oncol **28**(4): 562-569.

PURPOSE: In a phase III randomized trial, azacitidine significantly prolonged overall survival (OS) compared with conventional care regimens (CCRs) in patients with intermediate-2- and high-risk myelodysplastic syndromes. Approximately one third of these patients were classified as having acute myeloid leukemia (AML) under current WHO criteria. This analysis compared the effects of azacitidine versus CCR on OS in this subgroup. PATIENTS AND METHODS: Patients were randomly assigned to receive subcutaneous azacitidine 75 mg/m(2)/d or CCR (best supportive care [BSC] only, low-dose cytarabine (LDAC), or intensive chemotherapy [IC]). RESULTS: Of the 113 elderly patients (median age, 70 years) randomly assigned to receive azacitidine (n = 55) or CCR (n = 58; 47% BSC, 34% LDAC, 19% IC), 86% were considered unfit for IC. At a median follow-up of 20.1 months, median OS for azacitidine-treated patients was 24.5 months compared with 16.0 months for CCR-treated patients (hazard ratio = 0.47;
95% CI, 0.28 to 0.79; P = .005), and 2-year OS rates were 50% and 16%, respectively (P = .001). Two-year OS rates were higher with azacitidine versus CCR in patients considered unfit for IC (P = .0003). Azacitidine was associated with fewer total days in hospital (P < .0001) than CCR. CONCLUSION: In older adult patients with low marrow blast count (20% to 30%) WHO-defined AML, azacitidine significantly prolongs OS and significantly improves several patient morbidity measures compared with CCR.

13 [143]. Figueroa, M. E., L. Skrabanek, et al. (2009). "MDS and secondary AML display unique patterns and abundance of aberrant DNA methylation." Blood **114**(16): 3448-3458.

Increasing evidence shows aberrant hypermethylation of genes occurring in and potentially contributing to pathogenesis of myeloid malignancies. Several of these diseases, such as myelodysplastic syndromes (MDSs), are responsive to DNA methyltransferase inhibitors. To determine the extent of promoter hypermethylation in such tumors, we compared the distribution of DNA methylation of 14 000 promoters in MDS and secondary acute

myeloid leukemia (AML) patients enrolled in a phase 1 trial of 5-azacytidine and the histone deacetylase inhibitor entinostat against de novo AML patients and normal CD34(+) bone marrow cells. The MDS and secondary AML patients displayed more extensive aberrant DNA methylation involving thousands of genes than did the normal CD34(+) bone marrow cells or de novo AML blasts. Aberrant methylation in MDS and secondary AML tended to affect particular chromosomal regions, occurred more frequently in Alu-poor genes, and included prominent involvement of genes involved in the WNT and MAPK signaling pathways. DNA methylation was also measured at days 15 and 29 after the first treatment cycle. DNA methylation was reversed at day 15 in a uniform manner throughout the genome, and this effect persisted through day 29, even without continuous administration of the study drugs. This trial was registered at www.clinicaltrials.gov as J0443.

14 [103]. Garcia-Manero, G., S. D. Gore, et al. (2011). "Phase I study of oral azacitidine in myelodysplastic syndromes, chronic myelomonocytic leukemia, and acute myeloid leukemia." J Clin Oncol **29**(18): 2521-2527.

PURPOSE: To determine the maximum-tolerated dose (MTD), safety, pharmacokinetic and pharmacodynamic profiles, and clinical activity of an oral formulation of azacitidine in patients with myelodysplastic syndromes (MDSs), chronic myelomonocytic leukemia (CMML), or acute myeloid leukemia (AML). PATIENTS AND METHODS: Patients received 1 cycle of subcutaneous (SC) azacitidine (75 mg/m2) on the first 7 days of cycle 1, followed by oral azacitidine daily (120 to 600 mg) on the first 7 days of each additional 28-day cycle. Pharmacokinetic and pharmacodynamic profiles were evaluated during cycles 1 and 2. Adverse events and hematologic responses were recorded. Cross-over to SC azacitidine was permitted for nonresponders who received >/= 6 cycles of oral azacitidine. RESULTS: Overall, 41 patients received SC and oral azacitidine (MDSs, n = 29; CMML, n = 4; AML, n = 8). Dose-limiting toxicity (grade 3/4 diarrhea) occurred at the 600-mg dose and MTD was 480 mg. Most common grade 3/4 adverse events were diarrhea (12.2%), nausea (7.3%), vomiting (7.3%), febrile neutropenia (19.5%), and fatigue (9.8%). Azacitidine exposure increased with escalating oral doses. Mean relative oral bioavailability ranged from 6.3% to 20%. Oral and SC azacitidine decreased DNA methylation in blood, with maximum effect at day 15 of each cycle. Hematologic responses occurred in patients with MDSs and CMML. Overall response rate (i.e., complete remission, hematologic improvement, or RBC or platelet transfusion independence) was 35% in previously treated patients and 73% in previously untreated

patients. CONCLUSION: Oral azacitidine was bioavailable and demonstrated biologic and clinical activity in patients with MDSs and CMML.

15 [36]. Garcia-Manero, G., E. Jabbour, et al. (2013). "Randomized open-label phase II study of decitabine in patients with low- or intermediate-risk myelodysplastic syndromes." J Clin Oncol **31**(20): 2548-2553.

PURPOSE: This open-label, randomized phase II trial assessed efficacy and tolerability of two low-dose regimens of subcutaneous (SC) decitabine in patients with low- or intermediate-1-risk myelodysplastic syndrome (MDS). PATIENTS AND METHODS: Patients received decitabine 20 mg/m(2) SC per day for 3 consecutive days on days 1, 2, and 3 every 28 days (schedule A) or 20 mg/m(2) SC per day once every 7 days on days 1, 8, and 15 every 28 days (schedule B) for up to 1 year. Primary efficacy end point was overall improvement rate (OIR: complete remission [CR], partial remission [PR], marrow CR [mCR], or hematologic improvement [HI]). Secondary end points were HI, transfusion independence, cytogenetic response, overall survival (OS), and time to acute myeloid leukemia or death. RESULTS: Efficacy and safety populations were identical: schedule A, n = 43; schedule B, n = 22. Median time from MDS diagnosis to treatment was 3.6 months; 89% had de novo MDS. The trial was terminated early on achievement of protocol-defined OIR superiority of schedule A over schedule B; OIR was 23% for schedule A (seven CRs, three HIs) and 23% for schedule B (one mCR, one PR, three HIs). No differences were observed in secondary end points. Median OS was not reached; approximately 70% of patients were alive at 500 days. Patients in schedule A (67%) and schedule B (59%) were RBC/platelet independent on study. The most frequent drug-related adverse events overall were neutropenia (28% v 36%), anemia (23% v 18%), and thrombocytopenia (16% v 32%). CONCLUSION: In this phase II study, low-dose decitabine showed promising results in patients with low- or intermediate-1-risk MDS.

16 [159]. Garcia-Manero, G., M. L. Stoltz, et al. (2008). "A pilot pharmacokinetic study of oral azacitidine." Leukemia **22**(9): 1680-1684.

Azacitidine is a pyrimidine nucleoside analog of cytidine with hypomethylating and antileukemia activity. Azacitidine has been shown to have survival benefits in patients with high-risk myelodysplastic syndrome (MDS), and has activity in the treatment of acute myelogenous leukemia (AML). It is administered by subcutaneous (s.c.) or intravenous (i.v.) injection daily at a dose of 75 mg/m(2) for 7 days every 4 weeks. An oral formulation would facilitate dosing, reduce administration side effects

and potentially maximize azacitidine pharmacologic action. Previously, oral formulations of this class of agent have failed due to rapid catabolism by cytidine deaminase and hydrolysis in aqueous environments. Development of a film-coated formulation has circumvented this difficulty. In a formulation feasibility pilot study, four subjects with solid malignant tumors, AML or MDS received single oral doses of 60 or 80 mg azacitidine. Subjects demonstrated measurable plasma concentrations of azacitidine,
allowing bioavailability comparisons to be made to historical pharmacokinetic data for s.c. azacitidine. Subjects safely tolerated 80 mg, a dose for which the mean bioavailability was 17.4% of historic s.c. exposure. No severe drug-related toxicities were observed. These data suggest that oral azacitidine is bioavailable in humans and should be studied in formal phase 1 trials.

17 [126]. Grovdal, M., M. Karimi, et al. (2010). "Maintenance treatment with azacytidine for patients with high-risk myelodysplastic syndromes (MDS) or acute myeloid leukaemia following MDS in complete remission after induction chemotherapy." Br J Haematol **150**(3): 293-302.

This prospective Phase II study is the first to assess the feasibility and efficacy of maintenance 5-azacytidine for older patients with high-risk myelodysplastic syndrome (MDS), chronic myelomonocytic leukaemia and MDS-acute myeloid leukaemia syndromes in complete remission (CR) after induction chemotherapy. Sixty patients were enrolled and treated by standard induction chemotherapy. Patients that reached CR started maintenance therapy with subcutaneous azacytidine, 5/28 d until relapse. Promoter-methylation status of CDKN2B (P15 ink4b), CDH1 and HIC1 was examined pre-induction, in CR and 6, 12 and 24 months post CR. Twenty-four (40%) patients achieved CR after induction chemotherapy and 23 started maintenance treatment with azacytidine. Median CR duration was 13.5 months, >24 months in 17% of the patients, and 18-30.5 months in the four patients with trisomy 8. CR duration was not associated with CDKN2B methylation status or karyotype. Median overall survival was 20 months. Hypermethylation of CDH1 was significantly associated with low CR rate, early relapse, and short overall survival (P = 0.003). 5-azacytidine treatment, at a dose of 60 mg/m(2) was well tolerated. Grade III-IV thrombocytopenia and neutropenia occurred after 9.5 and 30% of the cycles, respectively, while haemoglobin levels increased during treatment. 5-azacytidine treatment is safe, feasible and may be of benefit in a subset of patients.

18 [184]. Kantarjian, H., J. P. Issa, et al. (2006). "Decitabine improves patient outcomes in myelodysplastic syndromes: results of a phase III randomized study." Cancer **106**(8): 1794-1803.

BACKGROUND: Aberrant DNA methylation, which results in leukemogenesis, is frequent in patients with myelodysplastic syndromes (MDS) and is a potential target for pharmacologic therapy. Decitabine indirectly depletes methylcytosine and causes hypomethylation of target gene promoters. METHODS: A total of 170 patients with MDS were randomized to receive either decitabine at a dose of 15 mg/m2 given intravenously over 3 hours every 8 hours for 3 days (at a dose of 135 mg/m2 per course) and repeated every 6 weeks, or best supportive care. Response was assessed using the International Working Group criteria and required that response criteria be met for at least 8 weeks. RESULTS: Patients who were treated with decitabine achieved a significantly higher overall response rate (17%), including 9% complete responses, compared with supportive care (0%) (P < .001). An additional 12 patients who were treated with decitabine (13%) achieved hematologic improvement. Responses were durable (median, 10.3 mos) and were associated with transfusion independence. Patients treated with decitabine had a trend toward a longer median time to acute myelogenous leukemia (AML) progression or death compared with patients who received supportive care alone (all patients, 12.1 mos vs. 7.8 mos [P = 0.16]; those with International Prognostic Scoring System intermediate-2/high-risk disease, 12.0 mos vs. 6.8 mos [P = 0.03]; those with de novo disease, 12.6 mos vs. 9.4 mos [P = 0.04]; and treatment-naive patients, 12.3 mos vs. 7.3 mos [P = 0.08]). CONCLUSIONS: Decitabine was found to be clinically effective in the treatment of patients with MDS, provided durable responses, and improved time to AML transformation or death. The duration of decitabine therapy may improve these results further.

19 [182]. Kantarjian, H., Y. Oki, et al. (2007). "Results of a randomized study of 3 schedules of low-dose decitabine in higher-risk myelodysplastic syndrome and chronic myelomonocytic leukemia." Blood **109**(1): 52-57.

Epigenetic therapy with hypomethylating drugs is now the standard of care in myelodysplastic syndrome (MDS). Response rates remain low, and mechanism-based dose optimization has not been reported. We investigated the clinical and pharmacodynamic results of different dose schedules of decitabine. Adults with advanced MDS or chronic myelomonocytic leukemia (CMML) were randomized to 1 of 3 decitabine schedules: (1) 20 mg/m2 intravenously daily for 5 days; (2) 20 mg/m2 subcutaneously daily for 5 days; and (3) 10 mg/m2 intravenously daily for

10 days. Randomization followed a Bayesian adaptive design. Ninety-five patients were treated (77 with MDS, and 18 with CMML). Overall, 32 patients (34%) achieved a complete response (CR), and 69 (73%) had an objective response by the new modified International Working Group criteria. The 5-day intravenous schedule, which had the highest dose-intensity, was selected as optimal; the CR rate in that arm was 39%, compared with 21% in the 5-day subcutaneous arm and 24% in the 10-day intravenous arm (P < .05). The high dose-intensity arm was also superior at inducing hypomethylation at day 5 and at activating P15 expression at days 12 or 28 after therapy. We conclude that a low-dose, dose-intensity schedule of decitabine optimizes epigenetic modulation and clinical responses in MDS.

20 [172]. Kantarjian, H. M., S. O'Brien, et al. (2007). "Survival advantage with decitabine versus intensive chemotherapy in patients with higher risk myelodysplastic syndrome: comparison with historical experience." Cancer **109**(6): 1133-1137.

BACKGROUND: Decitabine, a hypomethylating agent, is active and has been approved for the treatment of myelodysplastic syndrome (MDS) and chronic myelomonocytic leukemia. Intensive chemotherapy is an accepted form of therapy for patients with higher risk MDS. The comparative efficacy of these 2 forms of treatment in MDS is unknown. The objective of the current study was to compare the efficacy and toxicity profiles of decitabine and intensive chemotherapy in MDS. METHODS: The authors compared lower intensity decitabine therapy (n = 115 patients) with intensive chemotherapy (as it is used in acute myeloid leukemia [AML]) in patients with higher risk MDS. Two comparisons were made with a cohort of 376 historic patients (from 1995 to 2005): The first comparison included a subcohort of 115 patients (Group A) who matched the 115 decitabine study patients according to age, International Prognostic Scoring System, and cytogenetics; and the second comparison included the whole cohort of 376 patients without matching (Group B). A multivariate analysis was performed for outcome. RESULTS: The complete remission (CR) rate according to AML criteria was 43% with decitabine, 46% with intensive chemotherapy in Group A, and 52% with intensive chemotherapy in Group B. Compared with Group A, mortality at 6 weeks was 3% with decitabine versus 13% with intensive chemotherapy (P = .006) and, at 3 months, 7% with decitabine versus 23% with intensive chemotherapy (P = .001). Survival was better with decitabine versus intensive chemotherapy in Group A (median survival: 22 months vs 12

months; P < .001). A multivariate analysis of survival in all 491 patients who received decitabine or intensive chemotherapy (Group B) selected decitabine as an independent, favorable prognostic factor for survival (P = .006; hazard ratio, 0.74) after accounting for the independent prognostic effect of pretreatment factors. CONCLUSIONS: In this analysis, decitabine was associated with a survival advantage compared with intensive chemotherapy in patients with higher risk MDS. Future studies should evaluate prospectively the results of decitabine versus intensive chemotherapy in this setting.

21 [74]. Kantarjian, H. M., X. G. Thomas, et al. (2012). "Multicenter, randomized, open-label, phase III trial of decitabine versus patient choice, with physician advice, of either supportive care or low-dose cytarabine for the treatment of older patients with newly diagnosed acute myeloid leukemia." J Clin Oncol **30**(21): 2670-2677.

PURPOSE: This multicenter, randomized, open-label, phase III trial compared the efficacy and safety of decitabine with treatment choice (TC) in older patients with newly diagnosed acute myeloid leukemia (AML) and poor- or intermediate-risk cytogenetics. PATIENTS AND METHODS: Patients (N = 485) age >/= 65 years were randomly assigned 1:1 to receive decitabine 20 mg/m(2) per day as a 1-hour intravenous infusion for five consecutive days every 4 weeks or TC (supportive care or cytarabine 20 mg/m(2) per day as a subcutaneous injection for 10 consecutive days every 4 weeks). The primary end point was overall survival (OS); the secondary end point was the complete remission (CR) rate plus the CR rate without platelet recovery (CRp). Adverse events (AEs) were recorded. RESULTS: The primary analysis with 396 deaths (81.6%) showed a nonsignificant increase in median OS with decitabine (7.7 months; 95% CI, 6.2 to 9.2) versus TC (5.0 months; 95% CI, 4.3 to 6.3; P = .108; hazard ratio [HR], 0.85; 95% CI, 0.69 to 1.04). An unplanned analysis with 446 deaths (92%) indicated the same median OS (HR, 0.82; 95% CI, 0.68 to 0.99; nominal P = .037). The CR rate plus CRp was 17.8% with decitabine versus 7.8% with TC (odds ratio, 2.5; 95% CI, 1.4 to 4.8; P = .001). AEs were similar for decitabine and cytarabine, although patients received a median of four cycles of decitabine versus two cycles of TC. The most common drug-related AEs with decitabine were thrombocytopenia (27%) and neutropenia (24%). CONCLUSION: In older patients with AML, decitabine improved response rates compared with standard therapies without major differences in safety. An unplanned survival analysis showed a benefit for decitabine, which was not observed at the time of the primary analysis.

22 [55]. Krug, U., A. Koschmieder, et al. (2012). "Feasibility of azacitidine added to standard chemotherapy in older patients with acute myeloid leukemia--a randomised SAL pilot study." PLoS One **7**(12): e52695.

INTRODUCTION: Older patients with acute myeloid leukemia (AML) experience short survival despite intensive chemotherapy. Azacitidine has promising activity in patients with low proliferating AML. The aim of this dose-finding part of this trial was to evaluate feasibility and safety of azacitidine combined with a cytarabine- and daunorubicin-based chemotherapy in older patients with AML. TRIAL DESIGN: Prospective, randomised, open, phase II trial with parallel group design and fixed sample size. PATIENTS AND METHODS: Patients aged 61 years or older, with untreated acute myeloid leukemia with a leukocyte count of <20,000/microl at the time of study entry and adequate organ function were eligible. Patients were randomised to receive azacitidine either 37.5 (dose level 1) or 75 mg/sqm (dose level 2) for five days before each cycle of induction (7+3 cytarabine plus daunorubicine) and consolidation (intermediate-dose cytarabine) therapy. Dose-limiting toxicity was the primary endpoint. RESULTS: Six patients each were randomised into each dose level and evaluable for analysis. No dose-limiting toxicity occurred in either dose level. Nine serious adverse events occurred in five patients (three in the 37.5 mg, two in the 75 mg arm) with two fatal outcomes. Two patients at the 37.5 mg/sqm dose level and four patients at the 75 mg/sqm level achieved a complete remission after induction therapy. Median overall survival was 266 days and median event-free survival 215 days after a median follow up of 616 days. CONCLUSIONS: The combination of azacitidine 75 mg/sqm with standard induction therapy is feasible in older patients with AML and was selected as an investigational arm in the randomised controlled part of this phase-II study, which is currently halted due to an increased cardiac toxicity observed in the experimental arm.

TRIAL REGISTRATION: This trial is registered at clinical trials.gov (identifier: NCT00915252).

23 [86]. Lubbert, M., B. H. Ruter, et al. (2012). "A multicenter phase II trial of decitabine as first-line treatment for older patients with acute myeloid leukemia judged unfit for induction chemotherapy." Haematologica **97**(3): 393-401.

BACKGROUND: The treatment of acute myeloid leukemia of older, medically non-fit patients still poses a highly unmet clinical need, and only few large, prospective studies have been performed in this setting. Given the established activity of hypomethylating agents such as

5-aza-2'-deoxycytidine (decitabine) in myelodysplastic syndromes and acute myeloid leukemia with 20-30% bone marrow blasts, we investigated whether this drug is also active in patients with more than 30% blasts. DESIGN AND METHODS: To evaluate the efficacy and toxicity of decitabine in patients over 60 years old with untreated acute myeloid leukemia ineligible for induction chemotherapy, 227 patients (median age, 72 years), many with comorbidities, adverse cytogenetics and/or preceding myelodysplastic syndrome were treated with this hypomethylating agent.
During the initial decitabine treatment (135 mg/m(2) total dose infused intravenously over 72 hours every 6 weeks), a median of two cycles was administered (range, 1-4). All-trans retinoic acid was administered to 100 patients during course 2. Fifty-two patients who completed four cycles of treatment subsequently received a median of five maintenance courses (range, 1-19) with a lower dose of decitabine (20 mg/m(2)) infused over 1 hour on 3 consecutive days every 4-6 weeks. RESULTS: The complete and partial remission rate was 26%, 95% CI (20%, 32%), and an antileukemic effect was noted in 26% of patients. Response rates did not differ between patients with or without adverse cytogenetics; patients with monosomal karyotypes also responded. The median overall survival from the start of decitabine treatment was 5.5 months (range, 0-57.5+) and the 1-year survival rate was 28%, 95%CI (22%,34%). Toxicities were predominantly hematologic. CONCLUSIONS: Decitabine is well tolerated by older, medically non-fit patients with acute myeloid leukemia; myelosuppression is the major toxicity. The response rate and overall survival were not adversely influenced by poor-risk cytogenetics or myelodysplastic syndrome. Because of these encouraging results, randomized studies evaluating single-agent decitabine versus conventional treatment are warranted. The study is registered with the German Clinical Trials Registry, number DRKS00000069.

24 [105]. Lubbert, M., S. Suciu, et al. (2011). "Low-dose decitabine versus best supportive care in elderly patients with intermediate- or high-risk myelodysplastic syndrome (MDS) ineligible for intensive chemotherapy: final results of the randomized phase III study of the European Organisation for Research and Treatment of Cancer Leukemia Group and the German MDS Study Group." J Clin Oncol **29**(15): 1987-1996.

PURPOSE: To compare low-dose decitabine to best supportive care (BSC) in higher-risk patients with myelodysplastic syndrome (MDS) age 60 years or older and ineligible for intensive chemotherapy. PATIENTS AND METHODS: Two-hundred thirty-three patients (median age, 70 years;

range, 60 to 90 years) were enrolled; 53% had poor-risk cytogenetics, and the median MDS duration at random assignment was 3 months. Primary end point was overall survival (OS). Decitabine (15 mg/m(2)) was given intravenously over 4 hours three times a day for 3 days in 6-week cycles. RESULTS: OS prolongation with decitabine versus BSC was not statistically significant (median OS, 10.1 v 8.5 months, respectively; hazard ratio [HR], 0.88; 95% CI, 0.66 to 1.17; two-sided, log-rank P = .38). Progression-free survival (PFS), but not acute myeloid leukemia (AML) -free survival (AMLFS), was significantly prolonged with decitabine versus BSC (median PFS, 6.6 v 3.0 months, respectively; HR, 0.68; 95% CI, 0.52 to 0.88; P = .004; median AMLFS, 8.8 v 6.1 months, respectively; HR, 0.85; 95% CI, 0.64 to 1.12; P = .24). AML transformation was significantly (P = .036) reduced at 1 year (from 33% with
BSC to 22% with decitabine). Multivariate analyses indicated that patients with short MDS duration had worse outcomes. Best responses with decitabine versus BSC, respectively, were as follows: complete response (13% v 0%), partial response (6% v 0%), hematologic improvement (15% v 2%), stable disease (14% v 22%), progressive disease (29% v 68%), hypoplasia (14% v 0%), and inevaluable (8% v 8%). Grade 3 to 4 febrile neutropenia occurred in 25% of patients on decitabine versus 7% of patients on BSC; grade 3 to 4 infections occurred in 57% and 52% of patients on decitabine and BSC, respectively. Decitabine treatment was associated with improvements in patient-reported quality-of-life (QOL) parameters. CONCLUSION: Decitabine administered in 6-week cycles is active in older patients with higher-risk MDS, resulting in improvements of OS and AMLFS (nonsignificant), of PFS and AML transformation (significant), and of QOL. Short MDS duration was an independent adverse prognosticator.

25 [152]. Lyons, R. M., T. M. Cosgriff, et al. (2009). "Hematologic response to three alternative dosing schedules of azacitidine in patients with myelodysplastic syndromes." J Clin Oncol **27**(11): 1850-1856.

PURPOSE: Azacitidine (AZA) is effective treatment for myelodysplastic syndromes (MDS) at a dosing schedule of 75 mg/m(2)/d subcutaneously for 7 days every 4 weeks. The initial phase of this ongoing multicenter, community-based, open-label study evaluated three alternative AZA dosing schedules without weekend dosing. PATIENTS AND METHODS: MDS patients were randomly assigned to one of three regimens every 4 weeks for six cycles: AZA 5-2-2 (75 mg/m(2)/d subcutaneously for 5 days, followed by 2 days no treatment, then 75 mg/m(2)/d for 2 days); AZA 5-2-5 (50 mg/m(2)/d subcutaneously for 5 days, followed by 2 days no treatment,

then 50 mg/m(2)/d for 5 days); or AZA 5 (75 mg/m(2)/d subcutaneously for 5 days). RESULTS: Of patients randomly assigned to AZA 5-2-2 (n = 50), AZA 5-2-5 (n = 51), or AZA 5 (n = 50), most were French-American-British (FAB) lower risk (refractory anemia [RA]/RA with ringed sideroblasts/chronic myelomonocytic leukemia with < 5% bone marrow blasts, 63%) or RA with excess blasts (30%), and 79 (52%) completed > or = six treatment cycles. Hematologic improvement (HI) was achieved by 44% (22 of 50), 45% (23 of 51), and 56% (28 of 50) of AZA 5-2-2, AZA 5-2-5, and AZA 5 arms, respectively. Proportions of RBC transfusion-dependent patients who achieved transfusion independence were 50% (12 of 24), 55% (12 of 22), and 64% (16 of 25), and of FAB lower-risk transfusion-dependent patients were 53% (nine of 17), 50% (six of 12), and 61% (11 of 18), respectively. In the AZA 5-2-2, AZA 5-2-5, and AZA 5 groups, 84%, 77%, and 58%, respectively, experienced > or = 1 grade 3 to 4 adverse events. CONCLUSION: All three alternative dosing regimens produced HI, RBC transfusion independence, and safety responses consistent with the currently approved AZA regimen.
These results support AZA benefits in transfusion-dependent lower-risk MDS patients.

26 [27]. Nand, S., M. Othus, et al. (2013). "A phase 2 trial of azacitidine and gemtuzumab ozogamicin therapy in older patients with acute myeloid leukemia." Blood **122**(20): 3432-3439.

This trial tested the safety and efficacy of a regimen consisting of hydroxyurea followed by azacitidine, 75 mg/m(2) for 7 days, and gemtuzumab ozogamicin, 3 mg/m(2) on day 8, in older patients with newly diagnosed acute myeloid leukemia. Those achieving a complete remission received 1 consolidation treatment followed by 4 cycles of azacitidine. The patients were stratified into good-risk (age 60-69 years or performance status 0-1) and poor-risk (age >/=70 years and performance status 2 or 3) groups. Specific efficacy and safety goals were defined as being supportive of further study of the regimen. Eighty-three patients were registered in the good-risk cohort and 59 in poor-risk cohort, with median age of 71 and 75 years, respectively. In the good-risk group, 35 patients (44%) achieved a complete remission. Median relapse-free and overall survivals were 8 and 11 months, respectively. Six patients (8%) died within 30 days of registration. In the poor-risk group, 19 (35%) achieved a complete remission. Median relapse-free and overall survivals were 7 and 11 months, respectively. Seven patients (14%) died early. The results of this trial met predefined goals for efficacy and safety for the poor-risk cohort but not the good-risk group. .

27 [42]. Passweg, J. R., T. Pabst, et al. (2014). "Azacytidine for acute myeloid leukemia in elderly or frail patients: a phase II trial (SAKK 30/07)." Leuk Lymphoma **55**(1): 87-91.

This phase II trial treated elderly or frail patients with acute myeloid leukemia (AML) with single-agent subcutaneous azacytidine at 100 mg/m(2), on 5 of 28 days for up to six cycles. Treatment was stopped for lack of response, or continued to progression in responders. The primary endpoint was response within 6 months. A response rate >/= 34% was considered a positive trial outcome. From September 2008 to April 2010, 45 patients from 10 centers (median age 74 [55-86] years) were accrued. Patients received four (1-21) cycles. Best response was complete response/complete response with incomplete recovery of neutrophils and/or platelets (CR/CRi) in eight (18%; 95% confidence interval [CI]: 8-32%.), 0 (0%) partial response (PR), seven (16%) hematologic improvement, 17 (38%) stable disease. Three non-responding patients stopped treatment after six cycles, 31 patients stopped early and 11 patients continued treatment for 8-21 cycles. Adverse events (grade >/= III) were infections (n = 13), febrile neutropenia (n = 8), thrombocytopenia (n = 7), dyspnea (p = 6), bleeding (n = 5) and anemia (n = 4). Median overall survival was 6 months. Peripheral blood blast counts, grouped at 30%, had a borderline significant association with response (p = 0.07). This modified azacytidine schedule is feasible for elderly or frail patients with AML in an outpatient setting with moderate, mainly hematologic, toxicity and response in a proportion of patients, although the primary objective was not reached.

28 [17]. Prebet, T., Z. Sun, et al. (2014). "Prolonged administration of azacitidine with or without entinostat for myelodysplastic syndrome and acute myeloid leukemia with myelodysplasia-related changes: results of the US Leukemia Intergroup trial E1905." J Clin Oncol **32**(12): 1242-1248.

PURPOSE: Although azacitidine (AZA) improves survival in patients with high-risk myelodysplastic syndrome, the overall response remains approximately 50%. Entinostat is a histone deacetylase inhibitor that has been combined with AZA with significant clinical activity in a previous phase I dose finding study. DESIGN: Open label phase II randomized trial comparing AZA 50 mg/m(2)/d given for 10 days +/- entinostat 4 mg/m(2)/d day 3 and day 10. All subtypes of myelodysplasia, chronic myelomonocytic leukemia, and acute myeloid leukemia with myelodysplasia-related changes were eligible for the study. The primary objective was the rate of hematologic normalization (HN; complete

remission + partial remission + trilineage hematological improvement). RESULTS: One hundred forty-nine patients were analyzed, including 97 patients with myelodysplastic syndrome and 52 patients with acute myeloid leukemia. In the AZA group, 32% (95% CI, 22% to 44%) experienced HN and 27% (95% CI, 17% to 39%) in the AZA + entinostat group. Both arms exceeded the HN rate of historical control (Cancer and Leukemia Group B 9221 trial), but only the AZA group fulfilled the primary objective of the study. Rates of overall hematologic response were 46% and 44%, respectively. Median overall survivals were 18 months for the AZA group and 13 months for the AZA + entinostat group. The combination arm led to less demethylation compared with the monotherapy arm, suggesting pharmacodynamic antagonism. CONCLUSION: Addition of entinostat to AZA did not increase clinical response as defined by the protocol and was associated with pharmacodynamic antagonism. However, the prolonged administration of AZA by itself seems to increase HN rate compared with standard dosing and warrants additional investigation.

29 [13]. Roboz, G. J., T. Rosenblat, et al. (2014). "International randomized phase III study of elacytarabine versus investigator choice in patients with relapsed/refractory acute myeloid leukemia." J Clin Oncol **32**(18): 1919-1926.

PURPOSE: Most patients with acute myeloid leukemia (AML) eventually experience relapse. Relapsed/refractory AML has a dismal prognosis and currently available treatment options are generally ineffective. The objective of this large, international, randomized clinical trial was to investigate the efficacy of elacytarabine, a novel elaidic acid ester of cytarabine, versus the investigator's choice of one of seven commonly used AML salvage regimens, including high-dose cytarabine, multiagent chemotherapy, hypomethylating agents, hydroxyurea, and supportive care. PATIENTS AND METHODS: A total of 381 patients with relapsed/refractory AML were treated in North America, Europe, and Australia. Investigators selected a control treatment for individual patients before random assignment. The primary end point was overall survival (OS). RESULTS: There were no significant differences in OS (3.5 v 3.3 months), response rate (23% v 21%), or relapse-free survival (5.1 v 3.7 months) between the elacytarabine and control arms, respectively. There was no significant difference in OS among any of the investigator's choice regimens. Prolonged survival was only achieved in a few patients in both study arms whose disease responded and who underwent allogeneic stem-cell transplantation. CONCLUSION: Neither elacytarabine nor any of the seven alternative treatment regimens provided clinically meaningful

benefit to these patients. OS in both study arms and for all treatments was extremely poor. Novel agents, novel clinical trial designs, and novel strategies of drug development are all desperately needed for this patient population.

30 [22]. Thepot, S., R. Itzykson, et al. (2014). "Azacitidine in untreated acute myeloid leukemia: a report on 149 patients." Am J Hematol **89**(4): 410-416.

Limited data are available on azacitidine (AZA) treatment and its prognostic factors in acute myeloid leukemia (AML). One hundred and forty-nine previously untreated AML patients considered ineligible for intensive chemotherapy received AZA in a compassionate patient-named program. AML diagnosis was de novo, post-myelodysplastic syndromes (MDS), post-MPN, and therapy-related AML in 51, 55, 13, and 30 patients, respectively. Median age was 74 years, median white blood cell count (WBC) was 3.2 x 10(9) /L and 58% of the patients had >/= 30% marrow blasts. Cytogenetics was adverse in 60 patients. Patients received AZA for a median of five cycles (range 1-31). Response rate (including complete remission/CR with incomplete recovery/partial remission) was 27.5% after a median of three cycles (initial response), and 33% at any time (best response). Only adverse cytogenetics predicted poorer response. Median overall survival (OS) was 9.4 months. Two-year OS was 51% in responders and 10% in non-responders (P<0.0001). Adverse cytogenetics, WBC >15 x 10(9) /L and ECOG-PS >/= 2 predicted poorer OS, while age and marrow blast percentage had no impact. Using MDS IWG 2006 response criteria, among patients with stable disease, those with hematological improvement had no significant survival benefit in a 7 months landmark analysis. Outcomes observed in this high-risk AML population treated with

AZA deserve comparison with those of patients treated intensively in prospective studies.

TOPIC: AML + Monoclonal Antibodies

1 [233]. Amadori, S., S. Suciu, et al. (2010). "Randomized trial of two schedules of low-dose gemtuzumab ozogamicin as induction monotherapy for newly diagnosed acute myeloid leukaemia in older patients not considered candidates for intensive chemotherapy. A phase II study of the EORTC and GIMEMA leukaemia groups (AML-19)." Br J Haematol **149**(3): 376-382.

This study compared two schedules of low-dose gemtuzumab ozogamicin (GO) as induction monotherapy for untreated acute myeloid leukaemia in older patients unfit for intensive chemotherapy, to identify the more

promising regimen for further study. Patients were randomized to receive either best supportive care or a course of GO according to one of two schedules: 3 mg/m(2) on days 1, 3 and 5 (arm A), or GO 6 mg/m(2) on day 1 and 3 mg/m(2) on day 8 (arm B). Primary endpoint was the rate of disease non-progression (DnP), defined as the proportion of patients either achieving a response or maintaining a stable disease following GO induction in each arm. Fifty-six patients were randomized in the two GO arms (A, n = 29; B, n = 27). The rate of DnP was 38% [90% confidence interval (CI), 23-55] in arm A, and 63% (90% CI, 45-78) in arm B. Peripheral cytopenias were the most common adverse events for both regimens. The all-cause early mortality rate was 14% in arm A and 11% in arm B. The day 1 + 8 schedule, which was associated with the highest rate of DnP, met the statistical criteria to be selected as the preferred regimen for phase III comparison with best supportive care.

2 [221]. Brunnberg, U., M. Mohr, et al. (2012). "Induction therapy of AML with ara-C plus daunorubicin versus ara-C plus gemtuzumab ozogamicin: a randomized phase II trial in elderly patients." Ann Oncol **23**(4): 990-996.

BACKGROUND: Chemotherapy for elderly patients with acute myeloid leukemia (AML) results in a median overall survival (OS) of </= 1 year. Elderly patients often present with cardiac comorbidity. Gemtuzumab ozogamicin (GO) is active in elderly (>/= 60 years) patients with relapsed AML with low cardiac toxicity. PATIENTS AND METHODS: This randomized phase II study compared a standard combination of ara-C and daunorubicin (DNR; 7+3) versus ara-C plus gemtuzumab ozogamicin (7+GO) as the first course of induction therapy. Primary objectives were comparison of blast clearance on day 16, event-free survival (EFS), and remission duration. OS, complete remission (CR), and tolerability were secondary objectives. RESULTS: One hundred and nineteen patients with de novo AML, treatment-related AML, AML with a history of myelodysplastic syndrome (MDS), or high-risk MDS entered the study. Median age of 115 patients (intent-to-treat population) was 69 years. Protocol outlined a second course 7+3 for patients without blast clearance and two courses of high-dose ara-C consolidation upon CR. Both treatments were equally effective in blast clearance, CR, EFS, remission duration, or OS (median: 7+3, 9 months; 7+GO, 10 months). Induction death rate was higher in the GO group due to veno-occlusive disease. CONCLUSION: The study did not show significant superiority of 7+GO over standard 7+3.

3 [208]. Burnett, A. K., R. K. Hills, et al. (2013). "The addition of gemtuzumab ozogamicin to low-dose Ara-C improves remission rate but does not significantly prolong survival in older patients with acute myeloid leukaemia: results from the LRF AML14 and NCRI AML16 pick-a-winner comparison." Leukemia **27**(1): 75-81.

The treatment of older patients with acute myeloid leukaemia, who are not considered suitable for conventional intensive therapy, is unsatisfactory. Low-dose Ara-C(LDAC) has been established as superior to best supportive care, but only benefits the few patients who enter complete remission. Alternative or additional treatments are required to improve the situation. This randomised trial compared the addition of the immunoconjugate, gemtuzumab ozogamicin (GO), at a dose of 5 mg on day 1 of each course of LDAC, with the intention of improving the remission rate and consequently survival. Between June 2004 and June 2010, 495 patients entered the randomisation. The addition of GO significantly improved the remission rate (30% vs 17%; odds ratio(OR) 0.48 (0.32-0.73); P=0.006), but not the 12 month overall survival (25% vs 27%). The reason for the induction benefit failing to improve OS was two-fold: survival of patients in the LDAC arm who did not enter remission and survival after relapse were both superior in the LDAC arm. Although the addition of GO to LDAC doubled the remission rate it did not improve overall survival. Maintaining remission in older patients remains elusive.

4 [226]. Burnett, A. K., R. K. Hills, et al. (2011). "Identification of patients with acute myeloblastic leukemia who benefit from the addition of gemtuzumab ozogamicin: results of the MRC AML15 trial." J Clin Oncol **29**(4): 369-377.

PURPOSE: Antibody-directed chemotherapy for acute myeloid leukemia (AML) may permit more treatment to be administered without escalating toxicity. Gemtuzumab ozogamicin (GO) is an immunoconjugate between CD33 and calicheamicin that is internalized when binding to the epitope. We previously established that it is feasible to combine GO with conventional chemotherapy. We now report a large randomized trial testing the addition of GO to induction and/or consolidation chemotherapy in untreated younger patients. PATIENTS AND METHODS: In this open-label trial, 1,113 patients, predominantly younger than age 60 years, were randomly assigned to receive a single dose of GO (3 mg/m(2)) on day 1 of induction course 1 with one of the following three induction schedules: daunorubicin and cytarabine; cytarabine, daunorubicin, and etoposide; or fludarabine, cytarabine, granulocyte colony-stimulating factor, and idarubicin. In remission, 948 patients were randomly assigned to GO in course 3 in combination with amsacrine, cytarabine, and etoposide or high-dose cytarabine. The primary end

points were response rate and survival. RESULTS: The addition of GO was well tolerated with no significant increase in toxicity. There was no overall difference in response or survival in either induction of consolidation. However, a predefined analysis by cytogenetics showed highly significant interaction with induction GO (P = .001), with significant survival benefit for patients with favorable cytogenetics, no benefit for patients with poor-risk disease, and a trend for benefit in intermediate-risk patients. An internally validated prognostic index identified approximately 70% of patients with a predicted benefit of 10% in 5-year survival. CONCLUSION: A substantial proportion of younger patients with AML have improved survival with the addition of GO to induction chemotherapy with little additional toxicity.

5 [211]. Burnett, A. K., N. H. Russell, et al. (2012). "Addition of gemtuzumab ozogamicin to induction chemotherapy improves survival in older patients with acute myeloid leukemia." J Clin Oncol **30**(32): 3924-3931.

PURPOSE: There has been little survival improvement in older patients with acute myeloid leukemia (AML) in the last two decades. Improving induction treatment may improve the rate and quality of remission and consequently survival. In our previous trial, in younger patients, we showed improved survival for the majority of patients when adding gemtuzumab ozogamicin (GO) to induction chemotherapy. PATIENTS AND METHODS: Untreated patients with AML or high-risk myelodysplastic syndrome (median age, 67 years; range, 51 to 84 years) were randomly assigned to receive induction chemotherapy with either daunorubicin/ara-C or daunorubicin/clofarabine, with (n = 559) or without (n = 556) GO 3 mg/m(2) on day 1 of course one of therapy. The primary end point was overall survival (OS). RESULTS: The overall response rate was 69% (complete remission [CR], 60%; CR with incomplete recovery [CRi], 9%), with no difference between GO (70%) and no GO (68%) arms. There was no difference in 30- or 60-day mortality and no major increase in toxicity with GO. With median follow-up of 30 months (range, 5.5 to 54.6 months), 3-year cumulative incidence of relapse was significantly lower with GO (68% v 76%; hazard ratio [HR], 0.78; 95% CI, 0.66 to 0.93; P = .007), and 3-year survival was significantly better (25% v 20%; HR, 0.87; 95% CI, 0.76 to 1.00; P = .05). The benefit was apparent across subgroups. There was no interaction with other treatment interventions. A meta-analysis of 2,228 patients in two United Kingdom National Cancer Research Institute trials showed significant improvements in relapse (HR, 0.82; 95% CI, 0.72 to 0.93; P = .002) and OS (HR, 0.88; 95% CI, 0.79 to 0.98; P = .02). CONCLUSION: Adding GO (3 mg/m(2)) to induction chemotherapy reduces relapse risk and improves survival with little increase in toxicity.

6 [215]. Castaigne, S., C. Pautas, et al. (2012). "Effect of gemtuzumab ozogamicin on survival of adult patients with de-novo acute myeloid leukaemia (ALFA-0701): a randomised, open-label, phase 3 study." Lancet **379**(9825): 1508-1516.

BACKGROUND: The results of the addition of gemtuzumab ozogamicin, an anti-CD33 antibody conjugate, to the standard treatment for patients with acute myeloid leukaemia in phase 3 trials were contradictory. We investigated whether the addition of low fractionated-dose gemtuzumab ozogamicin to standard front-line chemotherapy would improve the outcome of patients with this leukaemia without causing excessive toxicity. METHODS: In a phase 3, open-label study, undertaken in 26 haematology centres in France, patients aged 50-70 years with previously untreated de novo acute myeloid leukaemia were randomly assigned with a computer-generated sequence in a 1:1 ratio with block sizes of four to standard treatment (control group) with or without five doses of intravenous gemtuzumab ozogamicin (3 mg/m(2) on days 1, 4, and 7 during induction and day 1 of each of the two consolidation chemotherapy courses). The primary endpoint was event-free survival (EFS). Secondary endpoints were relapse-free (RFS), overall survival (OS), and safety. Analysis was by intention to treat. This study is registered with EudraCT, number 2007-002933-36. FINDINGS: 280 patients were randomly assigned to the control (n=140) and gemtuzumab ozogamicin groups (n=140), and 139 patients were analysed in each group. Complete response with or without incomplete platelet recovery to induction was 104 (75%) in the control group and 113 (81%) in the gemtuzumab ozogamicin group (odds ratio 1.46, 95% CI 0.20-2.59; p=0.25). At 2 years, EFS was estimated as 17.1% (10.8-27.1) in the control group versus 40.8% (32.8-50.8) in the gemtuzumab ozogamicin group (hazard ratio 0.58, 0.43-0.78; p=0.0003), OS 41.9% (33.1-53.1) versus 53.2% (44.6-63.5), respectively (0.69, 0.49-0.98; p=0.0368), and RFS 22.7% (14.5-35.7) versus 50.3% (41.0-61.6), respectively (0.52, 0.36-0.75; p=0.0003). Haematological toxicity, particularly persistent thrombocytopenia, was more common in the gemtuzumab ozogamicin group than in the control group (22 [16%] vs 4 [3%]; p<0.0001), without an increase in the risk of death from toxicity. INTERPRETATION: The use of fractionated lower doses of gemtuzumab ozogamicin allows the safe delivery of higher cumulative doses and substantially improves outcomes in patients with acute myeloid leukaemia. The findings warrant reassessment of gemtuzumab ozogamicin as front-line therapy for acute myeloid leukaemia. FUNDING: Wyeth (Pfizer).

7 [240]. Chevallier, P., J. Delaunay, et al. (2008). "Long-term disease-free survival after gemtuzumab, intermediate-dose cytarabine, and mitoxantrone in patients with CD33(+) primary resistant or relapsed acute myeloid leukemia." J Clin Oncol **26**(32): 5192-5197.

PURPOSE: To determine the antitumor activity and safety of a combination of gemtuzumab ozogamicin (GO), intermediate-dose cytarabine, and mitoxantrone (MIDAM) in patients with refractory or relapsed CD33(+) acute myeloid leukemia (AML). PATIENTS AND METHODS: We treated 62 patients with refractory (n = 18) or relapsed (n = 44) CD33(+) AML. Median age was 55.5 years. Salvage regimen consisted of GO 9 mg/m(2) on day 4, cytarabine 1 g/m(2) every 12 hours on days 1 through 5, and mitoxantrone 12 mg/m(2)/d on days 1 through 3. Median follow-up time was 26.5 months. RESULTS: Thirty-one patients (50%) achieved complete remission (CR), and eight patients (13%) had CR with delayed platelet recovery (CRp); the overall response (OR; CR + CRp) rate was 63%. A significantly higher OR rate was achieved in patients who had relapsed versus refractory AML (73% v 39%, respectively; P = .007) and patients with CD33 expression more than 98% of the blast population versus less than 98% (79% v 52.3%, respectively; P = .03). The overall, event-free, and disease-free survival rates were 41%, 33%, and 53% at 2 years, respectively. Leukocytosis more than 20,000/microL at MIDAM therapy, high-risk cytogenetics, and absence of postremission therapy were adverse prognostic factors. Age, disease status, and/or CD33 expression did not influence survival parameters. Four early toxic deaths occurred; a grade 3 to 4 hyperbilirubinemia rate of 16% was observed, and two patients had veno-occlusive disease (3%). CONCLUSION: The MIDAM regimen seems to be an effective salvage regimen for refractory/relapsed CD33(+) AML patients. These encouraging results support the need for a randomized phase III trial before considering this combination of GO and chemotherapy as superior or the standard of care treatment for refractory/relapsed CD33(+) AML patients.

8 [246]. Eom, K. S., H. J. Kim, et al. (2007). "Gemtuzumab ozogamicin in combination with attenuated doses of standard induction chemotherapy can successfully induce complete remission without increasing toxicity in patients with acute myeloid leukemia aged 55 or older." Eur J Haematol **79**(5): 398-404.

BACKGROUND: In this study, the effectiveness and safety of combining gemtuzumab ozogamicin (GO) with an abbreviated schedule of standard induction chemotherapy were assessed in 37 patients (aged > or =55) yr with previously untreated acute myeloid leukemia (AML). METHODS: GO

was administered at a dose of 6 mg/m(2) as a single 2-h intravenous infusion on day 1. Following GO, an abbreviated schedule of induction chemotherapy consisting of idarubicin (12 mg/m(2)/d, days 2-4), and N4-behenoyl-1-beta-arabinofuranosyl cytosine (300 mg/m(2)/d, days 2-6) was given. RESULTS: Thirty-seven patients were treated with GO in combination with chemotherapy. Complete remission (CR) and CR with incomplete platelet recovery were achieved in 28 patients (75.7%) and one patient (2.7%) respectively. Two patients (5.4%) died during induction and two patients (5.4%) with grade 4 treatment emergent adverse effects during chemotherapy did not complete induction chemotherapy. The majority of toxicities were mild and manageable. Severe myelosuppression was universal with significantly prolonged thrombocytopenic period. In total, 25 patients who received consolidation treatment, 19 patients remain alive at the time of analysis. Thirteen patients had undergone hematopoietic stem cell transplantation, three are preparing for transplantation and seven are receiving their consolidation chemotherapy course. CONCLUSION: Although only a relatively small number of cases were included in this preliminary study and the follow-up duration was short, frontline GO in combination with attenuated conventional chemotherapy was found to be effective and feasible in elderly patients with AML.

9 [254]. Feldman, E. J., J. Brandwein, et al. (2005). "Phase III randomized multicenter study of a humanized anti-CD33 monoclonal antibody, lintuzumab, in combination with chemotherapy, versus chemotherapy alone in patients with refractory or first-relapsed acute myeloid leukemia." J Clin Oncol **23**(18): 4110-4116.

PURPOSE: Lintuzumab (HuM195) is an unconjugated humanized murine monoclonal antibody directed against the cell surface myelomonocytic differentiation antigen CD33. In this study, the efficacy of lintuzumab in combination with induction chemotherapy was compared with chemotherapy alone in adults with first relapsed or primary refractory acute myeloid leukemia (AML). PATIENTS AND METHODS: Patients with relapsed or primary resistant AML (duration of first response, zero to 12 months) were randomly assigned to receive either mitoxantrone 8 mg/m(2), etoposide 80 mg/m(2), and cytarabine 1 g/m(2) daily for 6 days (MEC) in combination with lintuzumab 12 mg/m(2), or MEC alone. Overall response, defined as the rate of complete remission (CR) and CR with incomplete platelet recovery (CRp), was the primary end point of the study, with additional analyses of survival time and toxicity. RESULTS: A total of 191 patients were randomly assigned from November 1999 to April

2001. The percent CR plus CRp with MEC plus lintuzumab was 36% v 28% in patients treated with MEC alone (P = .28). The overall median survival was 156 days and was not different in the two arms of the study. Apart from mild antibody infusion-related toxicities (fever, chills, and hypotension), no differences in chemotherapy-related adverse effects, including hepatic and cardiac dysfunction, were observed with the addition of lintuzumab to induction chemotherapy. CONCLUSION: The addition of lintuzumab to salvage induction chemotherapy was safe, but did not result in a statistically significant improvement in response rate or survival in patients with refractory/relapsed AML.

10 [225]. Fernandez, H. F., Z. Sun, et al. (2011). "Autologous transplantation gives encouraging results for young adults with favorable-risk acute myeloid leukemia, but is not improved with gemtuzumab ozogamicin." Blood **117**(20): 5306-5313.

We report the results of a prospective, randomized phase 3 trial evaluating the use of gemtuzumab ozogamicin (GO) in an intensive consolidation approach in 657 patients 17-60 years of age. Patients in first complete remission (CR1) after cytarabine and standard- or high-dose daunorubicin induction received 2 cycles of consolidation with high-dose cytarabine followed by peripheral blood progenitor cell collection. The 352 patients who entered consolidation were randomized to receive GO (n = 132) or not (n = 138) and then proceeded to autologous hematopoietic cell transplantation (HCT). GO was given to 67 patients. Median follow-up was 50.9 months. Results of the intention-to-treat analysis demonstrated a 4-year disease-free survival (DFS) of 33.6% versus 35.9% (P = .54) and an overall survival (OS) of 41.3% versus 41.9% (P = .52) for those randomized to receive GO versus no GO, respectively. Patients with favorable- and intermediate-risk acute myeloid leukemia (AML) treated with high-dose daunorubicin and autologous HCT had 4-year DFS rates of 60% and 40% and OS rates of 80% and 49.3%, respectively. For younger AML patients in CR1, autologous HCT should be considered in favorable- and intermediate-cytogenetic risk patients who do not have an allogeneic donor. The addition of a single dose of GO in this setting did not improve outcomes.

11 [253]. Larson, R. A., E. L. Sievers, et al. (2005). "Final report of the efficacy and safety of gemtuzumab ozogamicin (Mylotarg) in patients with CD33-positive acute myeloid leukemia in first recurrence." Cancer **104**(7): 1442-1452.

BACKGROUND: In this study, the authors analyzed the efficacy and safety of gemtuzumab ozogamicin (GO) (Mylotarg), an antibody-targeted chemotherapy for CD33-positive acute myeloid leukemia (AML). METHODS: Patients with CD33-positive AML in first recurrence were entered in 3 open-label, single-arm, Phase II studies. Patients received monotherapy with GO 9 mg/m(2) as a 2-hour intravenous infusion in 2 doses separated by 2 weeks. Patients were evaluated for remission, survival, and treatment-emergent adverse events. RESULTS: Two hundred seventy-seven patients (median age, 61 yrs) were treated with GO, and 71 patients (26%) achieved remission, which was defined as < or = 5% blasts in the bone marrow without leukemic blasts in the peripheral blood, neutrophil recovery to > or = 1500/microL, hemoglobin > or = 9 g/dL, and independence from red blood cell and platelet transfusions. Complete remission (CR) with platelet recovery (> or = 100,000/microL) or without full platelet recovery (< 100,000/microL) (CRp) was observed in 35 patients (13%) and 36 patients (13%), respectively. The median recurrence-free survival was 6.4 months for patients who achieved CR and 4.5 months for patients who achieved CRp. Although expected incidences of Grade 3 or 4 neutropenia (98%) and thrombocytopenia (99%) were observed, the incidence of Grade 3 or 4 sepsis (17%) and pneumonia (8%) was relatively low. Grade 3 or 4 hyperbilirubinemia and hepatic aspartate aminotransferase and alanine aminotransferase elevations were reported in 29%, 18%, and 9% of patients, respectively; 0.9% of patients who did not undergo prior or subsequent hematopoietic stem cell transplantation developed hepatic venoocclusive disease after GO treatment. CONCLUSIONS: When it was administered to patients with CD33-positive AML in first recurrence, single-agent GO induced a 26% remission rate with a generally acceptable safety profile.

12 [206]. Petersdorf, S. H., K. J. Kopecky, et al. (2013). "A phase 3 study of gemtuzumab ozogamicin during induction and postconsolidation therapy in younger patients with acute myeloid leukemia." Blood **121**(24): 4854-4860.

This randomized phase 3 clinical trial evaluated the potential benefit of the addition of gemtuzumab ozogamicin (GO) to standard induction and postconsolidation therapy in patients with acute myeloid leukemia. Patients were randomly assigned to receive daunorubicin (45 mg/m(2) per day on days 1, 2, and 3), cytarabine (100 mg/m(2) per day by continuous infusion on days 1-7), and GO (6 mg/m(2) on day 4; DA+GO) vs standard induction therapy with daunorubicin (60 mg/m(2) per day on days 1, 2, and 3) and cytarabine alone (DA). Patients who achieved complete remission (CR) received 3 courses of high-dose cytarabine.

Those remaining in CR after consolidation were randomly assigned to receive either no additional therapy or 3 doses of GO (5 mg/m(2) every 28 days). From August 2004 until August 2009, 637 patients were registered for induction. The CR rate was 69% for DA+GO and 70% for DA (P = .59). Among those who achieved a CR, the 5-year relapse-free survival rate was 43% in the DA+GO group and 42% in the DA group (P = .40). The 5-year overall survival rate was 46% in the DA+GO group and 50% in the DA group (P = .85). One hundred seventy-four patients in CR after consolidation underwent the postconsolidation randomization. Disease-free survival was not improved with postconsolidation GO (HR, 1.48; P = .97). In this study, the addition of GO to induction or postconsolidation therapy failed to show improvement in CR rate, disease-free survival, or overall survival.

13 [228]. Rosenblat, T. L., M. R. McDevitt, et al. (2010). "Sequential cytarabine and alpha-particle immunotherapy with bismuth-213-lintuzumab (HuM195) for acute myeloid leukemia." Clin Cancer Res **16**(21): 5303-5311.

PURPOSE: Lintuzumab (HuM195), a humanized anti-CD33 antibody, targets myeloid leukemia cells and has modest single-agent activity against acute myeloid leukemia (AML). To increase the potency of the antibody without the nonspecific cytotoxicity associated with beta-emitters, the alpha-particle-emitting radionuclide bismuth-213 ((213)Bi) was conjugated to lintuzumab. This phase I/II trial was conducted to determine the maximum tolerated dose (MTD) and antileukemic effects of (213)Bi-lintuzumab, the first targeted alpha-emitter, after partially cytoreductive chemotherapy. EXPERIMENTAL DESIGN: Thirty-one patients with newly diagnosed (n = 13) or relapsed/refractory (n = 18) AML (median age, 67 years; range, 37-80) were treated with cytarabine (200 mg/m(2)/d) for 5 days followed by (213)Bi-lintuzumab (18.5-46.25 MBq/kg). RESULTS: The MTD of (213)Bi-lintuzumab was 37 MB/kg; myelosuppression lasting >35 days was dose limiting. Extramedullary toxicities were primarily limited to grade </=2 events, including infusion-related reactions. Transient grade 3/4 liver function abnormalities were seen in five patients (16%). Treatment-related deaths occurred in 2 of 21 (10%) patients who received the MTD. Significant reductions in marrow blasts were seen at all dose levels. The median response duration was 6 months (range, 2-12). Biodistribution and pharmacokinetic studies suggested that saturation of available CD33 sites by (213)Bi-lintuzumab was achieved after partial cytoreduction with cytarabine. CONCLUSIONS: Sequential administration of cytarabine and (213)Bi-lintuzumab is tolerable and can produce remissions in patients with AML.

TOPIC: ALL + Monoclonal Antibodies or Rituximab or Blinatumomab

1 [337]. Kantarjian, H., D. Thomas, et al. (2012). "Inotuzumab ozogamicin, an anti-CD22-calecheamicin conjugate, for refractory and relapsed acute lymphocytic leukaemia: a phase 2 study." Lancet Oncol **13**(4): 403-411.

BACKGROUND: The outlook for patients with refractory and relapsed acute lymphocytic leukaemia (ALL) is poor. CD22 is highly expressed in patients with ALL. Inotuzumab ozogamicin is a CD22 monoclonal antibody conjugated to the toxin calecheamicin. We did a phase 2 study to assess the efficacy of this antibody. METHODS: We recruited patients at the MD Anderson Cancer Center, Houston, TX, USA, between June, 2010, and March, 2011. Adults and children with refractory and relapsed ALL were eligible. Ten adults were treated before enrolment of children started. Patients were given 1.8 mg/m(2) inotuzumab ozogamicin intravenously over 1 h every 3-4 weeks (the first three adults and three children received 1.3 mg/m(2) in the first course). The primary endpoint was overall response (complete response or marrow complete response with no recovery of platelet count or incomplete recovery of neutrophil and platelet counts). Analysis was done by intention to treat. This study is registered, number NCT01134575. FINDINGS: 49 patients were enrolled and treated. Median age was 36 years (range 6-80). CD22 was expressed in more than 50% of blasts in all patients. The median number of courses was two (range one to five) and the median time between courses was 3 weeks (range 3-6). Nine (18%) patients had complete response, 19 (39%) had marrow complete response, 19 (39%) had resistant disease, and two (4%) died within 4 weeks of starting treatment. The overall response rate was 57% (95% CI 42-71). The most frequent adverse events during course one of treatment were fever (grade 1-2 in 20 patients, grade 3-4 in nine), hypotension (grade 1-2 in 12 patients, grade 3 in one), and liver-related toxic effects (bilirubin: grade 1-2 in 12 patients, grade 3 in two; raised aminotransferase concentration: grade 1-2 in 27 patients, grade 3 in one). INTERPRETATION: Inotuzumab ozogamicin shows promise as a treatment for refractory and relapsed ALL. FUNDING: Pfizer.

TOPIC: ALL + Rituximab or Blinatumomab

1 [533]. Bargou, R., E. Leo, et al. (2008). "Tumor regression in cancer patients by very low doses of a T cell-engaging antibody." Science **321**(5891): 974-977.

Previous attempts have shown the potential of T cells in immunotherapy of cancer. Here, we report on the clinical activity of a bispecific antibody construct called blinatumomab, which has the potential to engage all cytotoxic T cells in patients for lysis of cancer cells. Doses as low as 0.005

milligrams per square meter per day in non-Hodgkin's lymphoma patients led to an elimination of target cells in blood. Partial and complete tumor regressions were first observed at a dose level of 0.015 milligrams, and all seven patients treated at a dose level of 0.06 milligrams experienced a tumor regression. Blinatumomab also led to clearance of tumor cells from bone marrow and liver. T cell-engaging antibodies appear to have therapeutic potential for the treatment of malignant diseases.

2 [528]. Chevallier, P., A. Pigneux, et al. (2012). "Rituximab for the treatment of adult relapsed/refractory CD20 positive B-ALL patients: a pilot series." Leuk Res **36**(3): 311-315.

No series have reported the results of the use of the therapeutic anti-CD20 monoclonal antibody (Rituximab) in the setting of adult refractory/relapsed B-ALL. We report here the outcomes of such nine patients treated at two french institutions by a combination of Rituximab+chemotherapy. We showed that four patients could achieve complete response while four other patients were documented with blast clearance superior to 50% from the baseline in bone marrow. We conclude that our results suggested some efficacy for the use of Rituximab in combination with chemotherapy in the setting of refractory/relapsed adult B-ALL. Larger series within prospective trials are needed to confirm these results.

3 [527]. Klinger, M., C. Brandl, et al. (2012). "Immunopharmacologic response of patients with B-lineage acute lymphoblastic leukemia to continuous infusion of T cell-engaging CD19/CD3-bispecific BiTE antibody blinatumomab." Blood **119**(26): 6226-6233.

T cell-engaging CD19/CD3-bispecific BiTE Ab blinatumomab has shown an 80% complete molecular response rate and prolonged leukemia-free survival in patients with minimal residual B-lineage acute lymphoblastic leukemia (MRD(+) B-ALL). Here, we report that lymphocytes in all patients of a phase 2 study responded to continuous infusion of blinatumomab in a strikingly similar fashion. After start of infusion, B-cell counts dropped to < 1 B cell/muL within an average of 2 days and remained essentially undetectable for the entire treatment period. By contrast, T-cell counts in all patients declined to a nadir within < 1 day and recovered to baseline within a few days. T cells then expanded and on average more than doubled over baseline within 2-3 weeks under continued infusion of blinatumomab. A significant percentage of reappearing CD8(+) and CD4(+) T cells newly expressed activation marker CD69. Shortly after start of infusion, a transient release of cytokines dominated by IL-10, IL-6, and

IFN-gamma was observed, which no longer occurred on start of a second treatment cycle. The response of lymphocytes in leukemic patients to continuous infusion of blinatumomab helps to better understand the mode of action of this and other globally T cell-engaging Abs. The trial is registered with www.clinicaltrials.gov identifier NCT00560794.

4 [534]. Thomas, D. A., S. Faderl, et al. (2006). "Chemoimmunotherapy with hyper-CVAD plus rituximab for the treatment of adult Burkitt and Burkitt-type lymphoma or acute lymphoblastic leukemia." Cancer **106**(7): 1569-1580.

BACKGROUND: Adult Burkitt-type lymphoma (BL) and acute lymphoblastic leukemia (B-ALL) are rare entities composing 1% to 5% of non-Hodgkin lymphomas NHL) or ALL. Prognosis of BL and B-ALL has been poor with conventional NHL or ALL regimens, but has improved with dose-intensive regimens. METHODS: To evaluate the addition of rituximab, a CD20 monoclonal antibody, to intensive chemotherapy in adults with BL or B-ALL,

31 patients with newly diagnosed BL or B-ALL received the hyper-fractionated cyclophosphamide, vincristine, doxorubicin, and dexamethasone (hyper-CVAD) regimen with rituximab. Their median age was 46 years; 29% were 60 years or older. Rituximab 375 mg/m(2) was given on Days 1 and 11 of hyper-CVAD courses and on Days 1 and 8 of methotrexate and cytarabine courses. RESULTS: Complete remission (complete response [CR]) was achieved in 24 of 28 (86%) evaluable patients; 3 had a partial response, and 1 had resistant disease. There were no induction deaths. The 3-year overall survival (OS), event-free survival, and disease-free survival rates were 89%, 80%, and 88%, respectively. Nine elderly patients achieved CR with all of them in continuous CR (except 1 death in CR from infection), with a 3-year OS rate of 89%. Multivariate analysis of current and historical (those treated with hyper-CVAD alone) groups identified age and treatment with rituximab as favorable factors. CONCLUSIONS: The addition of rituximab to hyper-CVAD may improve outcome in adult BL or B-ALL, particularly in elderly patients.

5 [530]. Thomas, D. A., S. O'Brien, et al. (2010). "Chemoimmunotherapy with a modified hyper-CVAD and rituximab regimen improves outcome in de novo Philadelphia chromosome-negative precursor B-lineage acute lymphoblastic leukemia." J Clin Oncol **28**(24): 3880-3889.

PURPOSE: The adverse prognosis of CD20 expression in adults with de novo precursor B-lineage acute lymphoblastic leukemia (ALL) prompted incorporation of monoclonal antibody therapy with rituximab into the intensive chemotherapy regimen hyper-CVAD (fractionated cyclophosphamide, vincristine, doxorubicin, dexamethasone). Other

modifications (irrespective of CD20 expression) included early anthracycline intensification, alterations in number of risk-adapted intrathecal chemotherapy treatments for CNS prophylaxis, additional early and late intensifications, and extension of maintenance phase chemotherapy by 6 months. PATIENTS AND METHODS: Two hundred eighty-two adolescents and adults with de novo Philadelphia chromosome (Ph)-negative precursor B-lineage ALL were treated with standard or modified hyper-CVAD regimens. The latter incorporated standard-dose rituximab if CD20 expression > or = 20%. RESULTS: The complete remission (CR) rate was 95% with 3-year rates of CR duration (CRD) and survival (OS) of 60% and 50%, respectively. In the younger (age < 60 years) CD20-positive subset, rates of CRD and OS were superior with the modified hyper-CVAD and rituximab regimens compared with standard hyper-CVAD (70% v 38%; P < .001% and 75% v 47%, P = .003). In contrast, rates of CRD and OS for CD20-negative counterparts treated with modified versus standard hyper-CVAD regimens were similar (72% v 68%, P = not significant [NS] and 64% v 65%, P = NS, respectively). Older patients with CD20-positive ALL did not benefit from rituximab-based chemoimmunotherapy (rates of CRD 45% v 50%, P = NS and OS 28% v 32%, P = NS, respectively), related in part to deaths in CR. CONCLUSION: The incorporation of rituximab into the hyper-CVAD regimen appears to improve outcome for younger patients with CD20-positive Ph-negative precursor B-lineage ALL.

6 [526]. Topp, M. S., N. Gokbuget, et al. (2012). "Long-term follow-up of hematologic relapse-free survival in a phase 2 study of blinatumomab in patients with MRD in B-lineage ALL." Blood **120**(26): 5185-5187.

Persistence or recurrence of minimal residual disease (MRD) after chemotherapy results in clinical relapse in patients with acute lymphoblastic leukemia (ALL). In a phase 2 trial of B-lineage ALL patients with persistent or relapsed MRD, a T cell-engaging bispecific Ab construct induced an 80% MRD response rate. In the present study, we show that after a median follow-up of 33 months, the hematologic relapse-free survival of the entire evaluable study cohort of 20 patients was 61% (Kaplan-Meier estimate). The hema-tologic relapse-free survival rate of a subgroup of 9 patients who received allogeneic hematopoietic stem cell transplantation after blinatumomab treatment was 65% (Kaplan-Meier estimate). Of the subgroup of 6 Philadelphia chromosome-negative MRD responders with no further therapy after blinatumomab, 4 are in ongoing hematologic and molecular remission. We conclude that blinatumomab can induce long-lasting complete remission in B-lineage ALL patients with

persistent or recurrent MRD. The original study and this follow-up study are registered at www.clinicaltrials.gov as NCT00198991 and NCT00198978, respectively.

7 [529]. Topp, M. S., P. Kufer, et al. (2011). "Targeted therapy with the T-cell-engaging antibody blinatumomab of chemotherapy-refractory minimal residual disease in B-lineage acute lymphoblastic leukemia patients results in high response rate and prolonged leukemia-free survival." J Clin Oncol **29**(18): 2493-2498.

PURPOSE: Blinatumomab, a bispecific single-chain antibody targeting the CD19 antigen, is a member of a novel class of antibodies that redirect T cells for selective lysis of tumor cells. In acute lymphoblastic leukemia (ALL), persistence or relapse of minimal residual disease (MRD) after chemotherapy indicates resistance to chemotherapy and results in hematologic relapse. A phase II clinical study was conducted to determine the efficacy of blinatumomab in MRD-positive B-lineage ALL. PATIENTS AND METHODS: Patients with MRD persistence or relapse after induction and consolidation therapy were included. MRD was assessed by quantitative reverse transcriptase polymerase chain reaction for either rearrangements of immunoglobulin or T-cell receptor genes, or specific genetic aberrations. Blinatumomab was administered as a 4-week continuous intravenous infusion at a dose of 15 mug/m2/24 hours. RESULTS: Twenty-one patients were treated, of whom 16 patients became MRD negative. One patient was not evaluable due to a grade 3 adverse event leading to treatment discontinuation. Among the 16 responders, 12 patients had been molecularly refractory to previous chemotherapy. Probability for relapse-free survival is 78% at a median follow-up of 405 days. The most frequent grade 3 and 4 adverse event was lymphopenia, which was completely reversible like most other adverse events. CONCLUSION: Blinatumomab is an efficacious and well-tolerated treatment in patients with MRD-positive B-lineage ALL after intensive chemotherapy. T cells engaged by blinatumomab seem capable of eradicating chemotherapy-resistant tumor cells that otherwise cause clinical relapse.

TOPIC: AML + Checkpoint Blockade

No references found.

TOPIC: AML + CAR or CART

1 [542]. Brentjens, R. J., M. L. Davila, et al. (2013). "CD19-targeted T cells rapidly induce molecular remissions in adults with chemotherapy-refractory acute lymphoblastic leukemia." Sci Transl Med **5**(177): 177ra138.

Adults with relapsed B cell acute lymphoblastic leukemia (B-ALL) have a dismal prognosis. Only those patients able to achieve a second remission with no minimal residual disease (MRD) have a hope for long-term survival in the context of a subsequent allogeneic hematopoietic stem cell transplantation (allo-HSCT). We have treated five relapsed B-ALL subjects with autologous T cells expressing a CD19-specific CD28/CD3zeta second-generation dual-signaling chimeric antigen receptor (CAR) termed 19-28z. All patients with persistent morphological disease or MRD(+) disease upon T cell infusion demonstrated rapid tumor eradication and achieved MRD(-) complete remissions as assessed by deep sequencing polymerase chain reaction. Therapy was well tolerated, although significant cytokine elevations, specifically observed in those patients with morphologic evidence of disease at the time of treatment, required lymphotoxic steroid therapy to ameliorate cytokine-mediated toxicities. Indeed, cytokine elevations directly correlated to tumor burden at the time of CAR-modified T cell infusions. Tumor cells from one patient with relapsed disease after CAR-modified T cell therapy, who was ineligible for additional allo-HSCT or T cell therapy, exhibited persistent expression of CD19 and sensitivity to autologous 19-28z T cell-mediated cytotoxicity, which suggests potential clinical benefit of additional CAR-modified T cell infusions. These results demonstrate the marked antitumor efficacy of 19-28z CAR-modified T cells in patients with relapsed/refractory B-ALL and the reliability of this therapy to induce profound molecular remissions, forming a highly effective bridge to potentially curative therapy with subsequent allo-HSCT.

2 [555]. Kalos, M., B. L. Levine, et al. (2011). "T cells with chimeric antigen receptors have potent antitumor effects and can establish memory in patients with advanced leukemia." Sci Transl Med **3**(95): 95ra73.

Tumor immunotherapy with T lymphocytes, which can recognize and destroy malignant cells, has been limited by the ability to isolate and expand T cells restricted to tumor-associated antigens. Chimeric antigen receptors (CARs) composed of antibody binding domains connected to domains that activate T cells could overcome tolerance by allowing T

cells to respond to cell surface antigens; however, to date, lymphocytes engineered to express CARs have demonstrated minimal in vivo expansion and antitumor effects in clinical trials. We report that CAR T cells that target CD19 and contain a costimulatory domain from CD137 and the T cell receptor zeta chain have potent non-cross-resistant clinical activity after infusion in three of three patients treated with advanced chronic lymphocytic leukemia (CLL). The engineered T cells expanded >1000-fold in vivo, trafficked to bone marrow, and continued to express functional CARs at high levels for at least 6 months. Evidence for on-target toxicity included B cell aplasia as well as decreased numbers of plasma cells and hypogammaglobulinemia. On average, each infused CAR-expressing T cell was calculated to eradicate at least 1000 CLL cells. Furthermore, a CD19-specific immune response was demonstrated in the blood and bone marrow, accompanied by complete remission, in two of three patients. Moreover, a portion of these cells persisted as memory CAR(+) T cells and retained anti-CD19 effector functionality, indicating the potential of this major histocompatibility complex-independent approach for the effective treatment of B cell malignancies.

3 [547]. Xu, X. J., H. Z. Zhao, et al. (2013). "Efficacy and safety of adoptive immunotherapy using anti-CD19 chimeric antigen receptor transduced T-cells: a systematic review of phase I clinical trials." <u>Leuk Lymphoma</u> **54**(2): 255-260.

There remain some key questions regarding the adoptive infusion of chimeric antigen receptor (CAR) transduced T-cells in the clinical setting. This article systematically reviews the phase I clinical trials using CARs targeting CD19 in B-lineage malignancies. Twenty-nine patients were enrolled and the 6-month progression free survival for this cohort was 50.0 +/- 9.9%. Univariate analysis showed that patients benefited from lymphodepletion before CAR+T-cell infusion and the administration of interleukin-2 (IL-2). Longer-term persistence (>/= 4 weeks) and stronger expansion of CAR+ T-cells in the blood and higher peak serum interferon-gamma (IFN-gamma) level (>/= 200 pg/mL) were also related to superior outcome. Regarding treatment-related adverse events, the most prominent toxicities were fever, rigors, chills, acute renal failure, hypotension and capillary leak syndrome. In conclusion, anti-CD19 CAR+ T-cells have shown some benefits in patients with B-lineage malignancies and are well tolerated in most patients. Preconditioning and cytokine supplement are required to improve the clinical outcome.

TOPIC: ALL + CAR or CART

1 [594]. Maude, S. L., N. Frey, et al. (2014). "Chimeric antigen receptor T cells for sustained remissions in leukemia." N Engl J Med **371**(16): 1507-1517.

BACKGROUND: Relapsed acute lymphoblastic leukemia (ALL) is difficult to treat despite the availability of aggressive therapies. Chimeric antigen receptor-modified T cells targeting CD19 may overcome many limitations of conventional therapies and induce remission in patients with refractory disease. METHODS: We infused autologous T cells transduced with a CD19-directed chimeric antigen receptor (CTL019) lentiviral vector in patients with relapsed or refractory ALL at doses of 0.76x10(6) to 20.6x10(6) CTL019 cells per kilogram of body weight. Patients were monitored for a response, toxic effects, and the expansion and persistence of circulating CTL019 T cells. RESULTS: A total of 30 children and adults received CTL019. Complete remission was achieved in 27 patients (90%), including 2 patients with blinatumomab-refractory disease and 15 who had undergone stem-cell transplantation. CTL019 cells proliferated in vivo and were detectable in the blood, bone marrow, and cerebrospinal fluid of patients who had a response. Sustained remission was achieved with a 6-month event-free survival rate of 67% (95% confidence interval [CI], 51 to 88) and an overall survival rate of 78% (95% CI, 65 to 95). At 6 months, the probability that a patient would have persistence of CTL019 was 68% (95% CI, 50 to 92) and the probability that a patient would have relapse-free B-cell aplasia was 73% (95% CI, 57 to 94). All the patients had the cytokine-release syndrome. Severe cytokine-release syndrome, which developed in 27% of the patients, was associated with a higher disease burden before infusion and was effectively treated with the anti-interleukin-6 receptor antibody tocilizumab. CONCLUSIONS: Chimeric antigen receptor-modified T-cell therapy against CD19 was effective in treating relapsed and refractory ALL. CTL019 was associated with a high remission rate, even among patients for whom stem-cell transplantation had failed, and durable remissions up to 24 months were observed. (Funded by Novartis and others; CART19 ClinicalTrials.gov numbers, NCT01626495 and NCT01029366.).
